# Phase II Clinical Trial and Preclinical Evaluation of a Novel CD47 Blockade Combination in Refractory Microsatellite-Stable Metastatic Colorectal Cancer

**DOI:** 10.1158/2767-9764.CRC-25-0332

**Published:** 2025-11-20

**Authors:** Robert W. Lentz, Julie Lang, Todd M. Pitts, Patrick Blatchford, Junxiao Hu, Kimberly R. Jordan, Adrie Van Bokhoven, Stacey M. Bagby, Adrian T.A. Dominguez, Cameron A. Binns, Hannah R. Robinson, Nicole Balmaceda, Emily Baiyee, Alexis D. Leal, Sunnie S. Kim, S. Lindsey Davis, Christopher H. Lieu, Raymond C. Wadlow, Kristen Spencer, Aaron J. Scott, Patrick M. Boland, Howard S. Hochster, Wells A. Messersmith

**Affiliations:** 1 Division of Medical Oncology, Department of Medicine, University of Colorado School of Medicine, Aurora, Colorado.; 2 Department of Immunology and Microbiology, University of Colorado School of Medicine, Aurora, Colorado.; 3 Department of Biostatistics and Informatics, Colorado School of Public Health, University of Colorado Denver, Aurora, Colorado.; 4 Department of Pathology, University of Colorado School of Medicine, Aurora, Colorado.; 5 Inova Schar Cancer Institute, Fairfax, Virginia.; 6 Perlmutter Cancer Center of NYU Langone Health and NYU Grossman School of Medicine, New York, New York.; 7 Division of Hematology and Oncology, Department of Medicine, College of Medicine, The University of Arizona Cancer Center, Tucson, Arizona.; 8 Division of Medical Oncology, Department of Medicine, Robert Wood Johnson Medical School, Rutgers Cancer Institute of New Jersey, New Brunswick, New Jersey.

## Abstract

**Purpose:**

In this preclinical human immune system patient-derived xenograft (HIS-PDX) model and phase II clinical trial, we assessed evorpacept (anti-CD47 engineered fusion protein with inactive Fc), cetuximab, and pembrolizumab (triple therapy) in microsatellite-stable (MSS) colorectal cancer.

**Patients and Methods:**

HIS BALB/c-Rag2^null^Il2rγ^null^Sirpα^NOD^ mice with PDXs were treated with triple therapy or its components. Patients with refractory MSS colorectal cancer were treated with triple therapy in a safety run-in (stage 1) followed by expansion (stage 2, planned *N* = 42). The co-primary objectives were to determine the recommended dose of evorpacept and objective response rate (vs. historic control).

**Results:**

In HIS-PDX mice, triple therapy decreased the growth of MSS colorectal cancer tumors and increased tumor-infiltrating CD8^+^ T cells. Sixteen patients were treated on the clinical trial across two evorpacept dose levels: *N* = 12 in stage 1 and *N* = 4 in stage 2. Trial enrollment was terminated early because of safety concerns (one treatment-related grade 5 event each of hemophagocytic lymphohistiocytosis and cytokine release syndrome). Otherwise, the adverse event profile was as expected. Among all patients, the objective response rate was 6.3%; formal hypothesis testing was not performed. The disease control rate was 12.5%, the median progression-free survival was 2.3 months, and the median overall survival was 10.9 months. Blood- and tumor-based clinical trial correlative analyses identified innate and adaptive immune system activation.

**Conclusions:**

Whereas triple therapy demonstrated evidence of efficacy in refractory MSS colorectal cancer, safety concerns halted enrollment. Further investigation is necessary to determine the optimal use of CD47-targeted therapies in MSS colorectal cancer.

**Significance:**

Evorpacept, cetuximab, and pembrolizumab demonstrated antitumor activity in a preclinical HIS-PDX model and clinical trial in refractory MSS colorectal cancer; however, immune-related adverse events prompted early termination of study enrollment. Evidence of innate and adaptive antitumor immune activation was identified. The role of SIRPα/CD47 blockade in the treatment of MSS colorectal cancer needs to be further elucidated in future trials.

## Introduction

Colorectal cancer is the third most common cancer in both men and women (1). Advances in colorectal cancer treatment have improved survival, but in the metastatic setting, 5-year overall survival (OS) remains only 13% (1). Standard-of-care treatment for proficient mismatch repair/microsatellite-stable (pMMR/MSS) metastatic colorectal cancer (mCRC) consists of chemotherapy, biologic agents, and targeted therapies for a minority (2). In the refractory setting, available treatments are trifluridine/tipiracil with or without bevacizumab, fruquintinib, and regorafenib; however, objective response rates (ORR) are 1% to 6% with median OS less than 1 year (3–5).

In contrast, for the 5% of patients with deficient MMR/microsatellite instability–high mCRC, immune checkpoint inhibitors targeting the inhibitory adaptive immune system checkpoints PD-1 and CTLA-4, which are designed to generate an antitumor T-cell response, have revolutionized treatment over the last decade (6, 7). Unfortunately, MSS tumors are resistant to these checkpoint inhibitors, with the ORR of close to 0% in most trials (8–10). MSS colorectal cancer tumors are immunologically cold, especially in the presence of liver metastases, with an immunosuppressive tumor microenvironment (TME) including macrophages (11–15).

Innate immune checkpoint inhibitors are being evaluated in multiple settings and are intended to disrupt inhibitory “do not eat me” interactions between tumor and both phagocytes and NK cells (16, 17). The signal regulatory protein α (SIRPα)/CD47 axis is an inhibitory phagocytosis immune checkpoint expressed on phagocytes and colorectal cancer cells, respectively, blocking phagocytosis of tumor cells (16, 18, 19). This process is regulated by these antiphagocytic receptor–ligand interactions and pro-phagocytic signals, such as tumor antigen–bound opsonizing antibodies (16). CD47 is widely expressed in normal human cells; however, SIRPα/CD47 blockade primarily results in tumor cell phagocytosis, as normal human cells lack pro-phagocytic signals (16, 17). However, red blood cells are an exception due to gain of prophagocytic signals with aging, and on-target anemia has frequently been observed with certain anti-CD47 agents (16, 20).

Evorpacept (ALX148) is an engineered fusion protein with two high-affinity CD47-binding domains of SIRPα linked to an inactive Fc region of human immunoglobulin (21). The inactive Fc domain prevents antibody-dependent cellular phagocytosis of CD47-expressing hematopoietic cells, minimizing anemia (16, 17, 21). In a syngeneic mouse model using CT26 colon carcinoma cells, ALX148 reduced myeloid immunosuppression, increased dendritic cell and CD8^+^ T-cell activation, and decreased tumor growth in combination with anti–PD-1 antibody (21, 22). It has been shown that PD-1/PD-L1 blockade is necessary to maximize T-cell cytotoxicity in combination with CD47/SIRPα blockade (23–26). *In vitro*, ALX148 enhanced the antibody-dependent cellular phagocytosis activity of the anti-EGFR antibody cetuximab in tumor cell lines (21). Evorpacept was previously evaluated clinically alone and in combination with anticancer antibodies, and pembrolizumab in solid and hematologic malignancies, and minimal anemia was observed (27). Phase 2 trials of evorpacept in combination with pembrolizumab with or without chemotherapy in advanced head/neck squamous cell carcinoma did not meet primary endpoints, whereas promising activity of evorpacept in combination with trastuzumab, ramucirumab, and paclitaxel was observed in advanced HER2+ gastric cancer (28, 29).

Based on these data, we hypothesized that blocking the SIRPα/CD47 inhibitory innate immune checkpoint with evorpacept and providing a pro-phagocytic (“eat me”) opsonizing signal with pembrolizumab would overcome resistance to single-agent anti–PD-1 antibody in patients with refractory MSS colorectal cancer by enhancing macrophage activation, generating synergistic innate and adaptive antitumor immunity, and reversing the immunosuppressive TME. Importantly, cetuximab was not intended to inhibit signaling pathways downstream of EGFR, rationalizing its use regardless of RAS/BRAF mutational status and prior exposure to EGFR inhibitor.

To test this, we conducted a human immune system (HIS) patient-derived xenograft (PDX) murine experiment and phase II clinical trial. In our preclinical study, BALB/c-Rag2^null^Il2rγ^null^Sirpα^NOD^ (BRGS) mice are injected with human hematopoietic stem cells, followed by subcutaneous implantation of MSS colorectal cancer PDX models. These mice develop human T and B lymphocytes, and to a lesser degree myeloid cells (30, 31). The resultant TME maintains differential human immune cell infiltration into “hot” versus “cold” tumors and allows immunotherapy regimens to be tested on human tumors (30, 32). We additionally hypothesized that the addition of liposomal clodronate (LC), which upon intracellular delivery results in apoptosis of mononuclear phagocytes, would abolish the anti-tumor activity of CD47 blockade (33–35).

## Patients and Methods

### Preclinical HIS murine experiment

Complete preclinical methods are available in the Supplementary Materials (30–32, 36–40). Animal work was approved by the University of Colorado Anschutz Medical Campus Institutional Animal Care and Use Committee. In brief, HIS-BRGS mice, following confirmation of engraftment, were implanted with PDX model CRC307P (MSS colorectal cancer; *KRAS* G12S; tumor derived from primary colon mass in a patient also with liver metastases, previously exposed to EGFR inhibitor) into the bilateral flanks (Supplementary Fig. S1A). Fourteen mice received LC 100 μL weekly intraperitoneal starting 1 week prior to other treatments. Mice were randomized into one of six treatment groups based on equivalent human chimerism: vehicle, vehicle + LC, ALX90 (preclinical surrogate for evorpacept with a similar modified/inactive Fc domain and binding properties, with mouse and human cross-reactivity), cetuximab + pembrolizumab, ALX90 + cetuximab + pembrolizumab (triple therapy), or triple therapy + LC (Supplementary Fig. S1B). The doses used were as follows: ALX90 30 mg/kg twice weekly intraperitoneally, pembrolizumab 15 mg/kg weekly intraperitoneally, and cetuximab 0.004 mg/mouse twice weekly intraperitoneally. Tumor size, weight, and health were assessed twice weekly. Treatment began 25 days following tumor implantation when the tumor sizes reached approximately 50 to 300 mm^3^. Mice were harvested 11 to 25 days following the start of treatment based on health and tumor size. At harvest, lymph nodes (LN), spleen (SP), and tumors were collected and processed into single-cell suspensions as previously described for flow cytometric analysis of immune subsets (Supplementary Table S1). Tumor volume was estimated using the following formula: (length × width^2^) × 0.52. Tumor growth was assessed using specific growth rate (SGR), which was calculated as folloes: $SGR\;=\;\ln\left(\frac{V2}{V1}\right)/\left(t2\;-\;t1\right)$, in which volume = V and time = t. This assesses percent growth by volume per day; a negative SGR corresponds to a regressing tumor, whereas a positive SGR corresponds to a growing tumor.

### Patients

Patients were eligible for this study if they were 18 years of age or older, had an Eastern Cooperative Oncology Group performance status of 0 to 1, and had pMMR/MSS mCRC which had progressed on at least two prior lines of therapy in the metastatic setting (a prior single line of therapy for metastatic disease, including fluoropyrimidine, oxaliplatin, and irinotecan, was allowed; Supplementary Table S2). For patients with left-sided, RAS/BRAF wild-type (WT) colorectal cancer, a prior EGFR inhibitor was required. For all patients, prior EGFR inhibitor was allowed, and enrollment was not restricted based on primary tumor location. Additionally, patients were required to have measurable tumor burden as assessed by RECIST v1.1 and acceptable laboratory results. Key exclusion criteria were prior immune therapy–based treatment (anti–PD-1, anti–PD-L1, agents targeting other stimulatory or co-inhibitory T-cell receptors, anti-CD47, and/or anti-SIRPα) and autoimmune disease. See Supplementary Protocol for additional information.

### Clinical trial design

This was a multi-institution, open-label, single-arm, two-stage, and investigator-initiated phase II clinical trial (with safety run-in) conducted through the Academic Gastrointestinal Cancer Consortium (NCT05167409). The trial was performed in two stages. In stage 1 (safety run-in), six patients were planned to enroll and be treated with evorpacept at dose level 1 (15 mg/kg weekly), cetuximab (400 mg/m^2^ then 250 mg/m^2^ weekly), and pembrolizumab (200 mg every 3 weeks). If these doses were tolerated [assessed by a first-cycle dose-limiting toxicity (DLT) rate of less than 33%; see Supplementary Protocol], then the trial would proceed to stage 2 at the evorpacept recommended dose (RD) of 15 mg/kg weekly. If the doses were not tolerated, then additional patients would be enrolled and treated with evorpacept at dose level −1 (10 mg/kg weekly) and standard doses of cetuximab and pembrolizumab. The cetuximab dose could be reduced only if DLT(s) were incontrovertibly attributable to cetuximab, and pembrolizumab dose reductions were not allowed. In stage 2 (expansion), 42 additional patients would be enrolled and treated with evorpacept at the RD and standard doses of cetuximab and pembrolizumab. Among the first *N* = 6 patients treated in stage 1 at evorpacept dose level 1, cetuximab, and pembrolizumab, one grade 5 DLT occurred [hemophagocytic lymphohistiocytosis (HLH)]. The protocol was amended to allow additional evaluation in stage 1 at evorpacept dose levels 1 and −1 (see Supplementary Protocol).

In both stages 1 and 2, patients received evorpacept, cetuximab, and pembrolizumab in 21-day cycles. For all patients, peripheral blood was collected on day 1 of each cycle for pharmacodynamic analyses. A subset of patients in stage 2 were to undergo paired pretreatment and on-treatment biopsies, and archival tissue would be obtained if available for all patients in stage 2. Imaging was obtained every 9 weeks for evaluation of response using RECIST v1.1 and iRECIST (41, 42). Treatment was continued until disease progression by RECIST v1.1, unacceptable toxicity, or death. The maximum duration of pembrolizumab was 35 cycles, whereas there was no maximum duration of evorpacept or cetuximab. Under certain circumstances in which patients had clinical stability or better despite disease progression by RECIST v1.1, patients could consent to continue study treatments beyond progression. If the subsequent scan showed progression (by iRECIST), the patient would exit the study. Adverse events (AE) were classified according to Common Terminology Criteria for Adverse Events v5.0.

This study was conducted after approval by a local Human Investigations Committee and in accord with an assurance filed with and approved by the Department of Health and Human Services, data were anonymized to protect the identities of patients involved in the research, and investigators obtained informed consent from each participant. The study followed the CONSORT statement guidelines.

### Clinical trial objectives and statistical methods

The co-primary objectives of the study were to determine the RD of evorpacept in combination with cetuximab and pembrolizumab and determine the ORR [defined as partial response (PR) or complete response] by RECIST v1.1. Additional efficacy endpoints included disease control rate (DCR; defined as stable disease, PR, or complete response), duration of response, progression-free survival (PFS), and OS. Safety and tolerability were evaluated as secondary objectives based on first-cycle DLTs in stage 1 and Common Terminology Criteria for Adverse Events v5.0 for all study participants. The exploratory objectives were to evaluate pharmacodynamic effects in blood and tumor tissue.

The primary hypothesis was that evorpacept, cetuximab, and pembrolizumab would demonstrate an improved ORR compared with the historic control rate. The one-sided proportion test would be conducted for ORRs against the null hypothesis that the ORR is ≤3% (based on historic results with regorafenib and trifluridine/tipiracil in the refractory setting; refs. 4, 43, 44). The cumulative frequency, estimated proportion, and exact 95% confidence intervals (CI) would be reported. A sample size of 48 patients (including patients treated in both stages 1 and 2 at the evorpacept RD) was chosen, which would provide 87% power if the ORR in this trial is 15% with a target significance level of 0.05. The one-sided proportion test would be conducted in a similar manner for the secondary endpoint DCR. The time-to-event endpoints of PFS and OS would be summarized by the Kaplan–Meier method to estimate the median time-to-event and corresponding 95% CI. Due to termination of study enrollment prior to completing the planned accrual, formal hypothesis testing was not performed. All analyses were conducted in the safety-evaluable population, defined as all patients who received any amount of study drugs.

### Clinical trial exploratory correlative analyses

Complete clinical trial correlative analysis methods are available in the Supplementary Materials (45, 46). In brief, multiplex IHC was performed to quantity levels of immune infiltrate, EGFR, CD47, and PD-L1 using formalin-fixed paraffin-embedded tissue sections. For mass cytometry, peripheral blood mononuclear cells were stained with a panel of antibodies conjugated to heavy metal ions (Supplementary Table S3). For T-cell receptor (TCR) sequencing, RNA was isolated from peripheral blood mononuclear cell samples, followed by V(D)J sequencing of TCR β chain libraries. The frequency of unique TCR clonotypes was calculated, and the D50 values, representing the number of unique clonotypes that account for 50% of all sequencing reads, and the Shannon entropy values, a diversity index representing a measure of randomness, are reported.

## Results

### ALX90, cetuximab, and pembrolizumab triple treatment increases cytotoxic T cells and inhibits tumor growth in a humanized mouse MSS colorectal cancer PDX model

**Figure 1.**
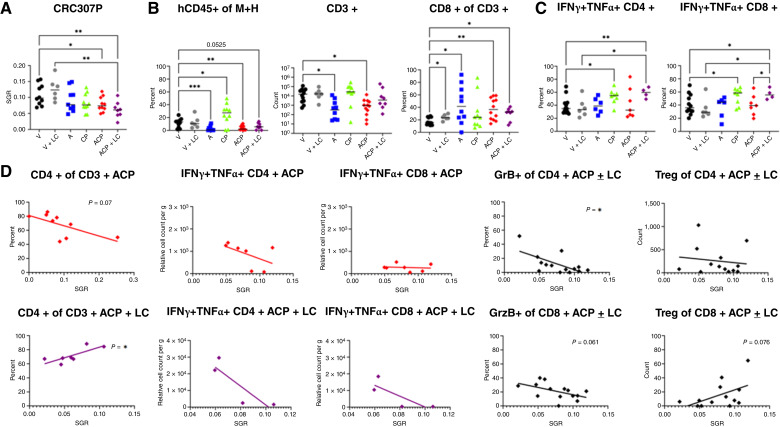
Triple therapy (ALX90, cetuximab, and pembrolizumab) activates human T cells and slows tumor growth of CRC307P colorectal cancer MSS PDX in HIS-BRGS mice. **A,** SGRs of CRC307P in HIS-BRGS mice among treatment groups: vehicle (V), ALX90 (A), cetuximab (C) + pembrolizumab (CP), ACP (ALX90 + cetuximab + pembrolizumab), LC, and ACP + LC. **B,** Human immune infiltration in CRC307P tumors in HIS-BRGS mice. Left, frequency of human immune (human CD45^+^ of mouse + human CD45^+^). Middle, number of human T cells. Right, frequency of CD8^+^ T cells among CD3^+^ cells. **C,** IFNγ+ and TNFα+ cytotoxic CD4^+^ and CD8^+^ tumor-infiltrating T cells. **D,** Immunophenotype correlation with tumor growth (SGR) among triple therapy (ACP, red), triple therapy following LC treatments (ACP + LC, purple), and all mice treated with triple therapy with or without LC (ACP ± LC, black). Statistics of linear correlation are provided; *, *P* < 0.05; **, *P* < 0.001. GrzB, granzyme B.

In HIS-BRGS mice with MSS colorectal cancer tumors, six experimental cohorts were evaluated (vehicle, vehicle + LC, ALX90 alone, cetuximab + pembrolizumab, and triple therapy with or without LC; Supplementary Fig. S1). Consistent with product warnings, 56% of mice treated with LC were euthanized because of poor health prior to harvest (47). No additional toxicity was observed in the treated groups compared with the vehicle control. Triple therapy significantly inhibited tumor growth, and surprisingly more so with the addition of LC (Fig. 1A). Analysis of both the peripheral immune system (LN and SP) and the tumor were performed to identify treatment-related differences in immune parameters as a result of treatment. ALX90, alone or in triple combination, resulted in significant loss of human immune cells in the tumors and less so in the SPs (Fig. 1B; Supplementary Fig. S2A). The minimal effect in the LNs, which contain very few mCD45^+^ cells, and the partial rescue of human T cells in both the SP and tumor with prior LC treatment, suggest the mouse myeloid cells are phagocytosing the human T cells following blockage of the SIRPα–CD47 macrophage tolerance pathway (Supplementary Fig. S2A and S2B).

The cetuximab + pembrolizumab combination increased the ratio of human:mouse immune infiltration into the tumor, with no significant changes in the peripheral lymph organs, whereas ALX90, alone or in triple combination, increased proportions of the human CD8^+^ T cells only in the tumors (Fig. 1B; Supplementary Fig. S2A). Consistent with the slower tumor growth in the triple therapy + LC cohort, we measured increased cytotoxic CD4^+^GrB+, CD4^+^, and CD8^+^ IFNγ+TNFα+ and a trend to more memory T cells and fewer CD8^+^ regulatory T cells (Tregs) in the tumors (Fig. 1C; Supplementary Fig. S3A). These same effects were not observed in the peripheral lymph organs in these mice with the exception of increased CD4 memory T cells in the LNs (Supplementary Fig. S4A and S4B). However, in mice treated with triple therapy without LC, the increased memory and cytotoxic T cells were significantly higher in the peripheral lymph organs but not in the tumor. ALX90 alone or in triple combination showed more activated CD8^+^ HLA-DR+ T cells in the LNs. CD4+ Tregs were increased in the LNs of the triple therapy + LC cohort and in the tumor of the triple therapy mice. We observed few changes among treatment cohorts in tumor properties, with the tumor maintaining an HLA class I (high), class II (low), poliovirus receptor (PVR; high), and PD-L1 (low) profile, although the PVR inhibitory receptor was significantly downregulated on tumor cells in the triple therapy cohort (Supplementary Fig. S4C).

The interplay of cytotoxic and regulatory immune cells likely contributes to overall tumor growth in individual mice. The highest numbers and frequencies of cytotoxic T cells and fewest CD8^+^ Tregs were observed in the triple therapy + LC cohort, which had the most significant reduction in tumor growth (Fig. 1C; Supplementary Fig. S3A). To assess contributions of distinct phenotypes on tumor growth, we correlated tumor SGR with immunotypes (Fig. 1D; Supplementary Fig. S3B). In this analysis, we found that cytotoxic CD4^+^ T cells largely correlated with reduced tumor growth in the triple therapy group, with the smallest tumors having the highest frequencies of CD4^+^ T cells with higher IFNγ and TNFα expression (Fig. 1D; Supplementary Fig. S3B). In the triple therapy + LC group, the CD8^+^ T cells had a more pronounced effect. Analysis of all mice treated with triple therapy, with or without LC, indicated a role for granzyme B+ and effector memory CD4^+^ and CD8^+^ T cells in the antitumor response (Fig. 1D; Supplementary Fig. S3B). In this model, the CD8^+^ Tregs showed a higher antitumor correlation than the CD4^+^ Tregs.

### LC-sensitive mouse macrophages interfere with ALX90, cetuximab, and pembrolizumab treatment

To address mechanism of action in our HIS mouse model, we treated two cohorts of mice with LC alone or followed by the triple therapy regimen. Contrary to our initial hypothesis, we found that the addition of LC to triple therapy unexpectedly resulted in a significant decrease in tumor growth, accompanied by the increased activated human T cells described above (Fig. 1).

**Figure 2.**
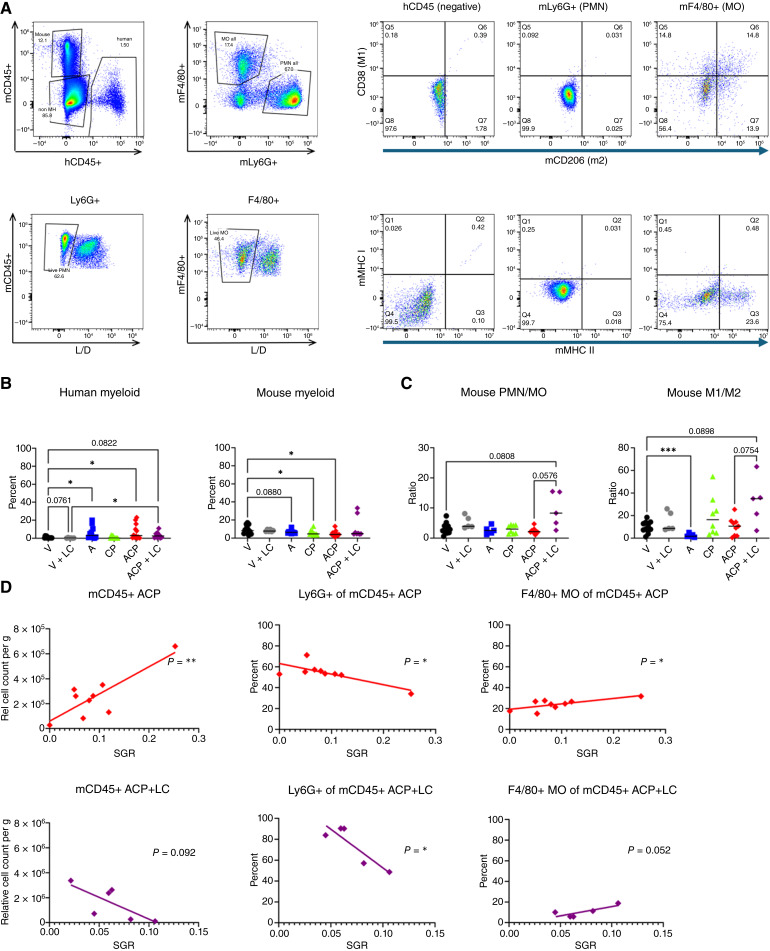
Role of mouse myeloid cells in CRC307P-bearing HIS-BRGS mice treated with combinations of ALX90, cetuximab, pembrolizumab, and LC. **A,** Flow cytometric gating strategy for quantification of mouse (mCD45^+^) myeloid populations: PMNs (Ly6G+), M1 (CD38^+^) and M2 (CD206^+^) macrophages (MO: F4/80+), and expression of mouse MHC class I and class II. **B,** Quantification of human (left, CD11b^+^ CD14^+^, CD33^+^, or CD11c^+^) and mouse (right, mCD45^+^) myeloid populations in tumors of HIS-CRC307P-BRGS mice among treatment groups: vehicle (V), ALX90 (A), cetuximab (C) + pembrolizumab (CP), ACP (ALX90 + cetuximab + pembrolizumab), LC, and ACP + LC. **C,** Ratios of PMN/MO and M1/M2 of F4/80 macrophages among mouse myeloid cells. **D,** Correlation of mouse myeloid populations with tumor growth (SGR) among triple therapy (ACP, top) and triple therapy following LC treatments (ACP + LC, bottom). Statistics of linear correlation are provided; *, *P* < 0.05; **, *P* < 0.001; ***, *P* < 0.0001.

LC has previously been reported to generate systemic T-cell activity by depleting immunosuppressive myeloid populations including myeloid-derived suppressor cells (monocytic greater than neutrophilic) and tumor-associated macrophages (33, 34, 47–49). In HIS-BRGS mice, most of the myeloid cells are of mouse origin, as mCD45^+^ cells are void of the T, B, and NK lineage due to genetic manipulations of recipient strain, and human myeloid cells are underrepresented in HIS-BRGS mice due lack of ability to compete with mouse myeloid cells. We confirmed decreased but not absent mCD45^+^ cells in the SPs of the surviving LC-treated cohorts (Supplementary Fig. S2B). Treatment with ALX90 alone or in triple combination increased the frequency of human and decreased the frequency of murine myeloid cells in the tumors (Fig. 2A and B). ALX90 alone resulted in a higher MO population, with more immunosuppressive M2 compared with antitumor M1 and less polymorphonuclear neutrophils (PMN; Fig. 2C). However, this trend was reversed with triple therapy following LC, with higher PMN:MO and M1:M2 ratios among the mouse CD45^+^ tumor infiltrating leukocytes. Notably, in our assessment of the influence of murine immune populations on tumor growth, we found that the presence of mCD45^+^ cells in the tumor correlated with greatly increased tumor size in the triple therapy cohort, but this was reversed when the mouse MO were targeted with LC, suggesting their contribution to an immunosuppressive TME (Fig. 2D). Ly6G+ PMNs correlated with antitumor effect, whereas the F4/80+ MOs correlated with pro-tumor effect in mice treated with triple therapy, with or without LC. These data suggest that in this model, LC causes incomplete and selective myeloid depletion, with more depletion of M2 than M1 and more depletion of macrophages than neutrophils, resulting in a less immunosuppressive TME and improved antitumor T-cell effect. The data also suggest that PMNs are important in the SIRPα phagocytic pathway in this model.

### Patient characteristics

Between July 2022 and August 2023, 25 patients were screened, 19 patients were enrolled, and 16 patients received at least one dose of the study drug (*CONSORT* in Supplementary Fig. S5). In stage 1, *N* = 12 patients were treated. Initially, *N* = 6 patients were treated at evorpacept dose level 1. Following a DLT (grade 5 HLH), the protocol was amended, and *N* = 3 patients each were subsequently treated at evorpacept dose levels −1 and 1. No additional DLTs were observed, and the study proceeded to stage 2, in which *N* = 4 patients were treated at evorpacept dose level 1. Accrual was terminated following the second treatment-related grade 5 event [cytokine release syndrome (CRS)]. All patients on study received cetuximab and pembrolizumab in combination with evorpacept.

The data cutoff was October 30, 2024. All patients were pMMR/MSS, most patients were male with Eastern Cooperative Oncology Group performance status zero, and the primary tumor site was variable (Table 1). Mutations in *KRAS*, *NRAS*, and *BRAF* were present in 6 (38%), 1 (6%), and 0 patients, respectively, with status unknown for some patients. As expected in this setting, liver metastases were common; 13 (81%) of patients had active liver metastasis at study entry, 1 (6%; subject ID 36-002) patient had prior liver metastasis treated with ablation and no active liver metastasis at study entry, and 2 (13%) patients had no history of liver metastasis. The median number of prior therapy lines was 3, including prior EGFR inhibitor in 8 (50%) patients.

**Table 1.** Baseline characteristics.

Characteristic	Number of patients (*N* = 16) *N* (%)
Age (years; median, minimum–maximum)	53 (36–81)
Sex	​
Female	5 (31)
Male	11 (69)
Ethnicity	​
Hispanic or Latino	1 (6)
Not Hispanic or Latino	15 (94)
Race	​
Caucasian	13 (81)
African American	1 (6)
Asian	1 (6)
Other	1 (6)
Eastern Cooperative Oncology Group performance status	​
0	11 (69)
1	5 (31)
Primary tumor site	​
Right	5 (31)
Left	5 (31)
Transverse	1 (6)
Unknown	5 (31)
*KRAS*	​
WT	9 (56)
Mutant	6 (38)
Unknown	1 (6)
*NRAS*	​
WT	13 (81)
Mutant	1 (6)
Unknown	2 (13)
*BRAF*	​
WT	11 (69)
Mutant	0
Unknown	5 (31)
Tumor mutation burden (Mut/Mb)	​
<10	9 (56)
≥10	0
Unknown	7 (44)
Liver metastases	​
Present at study entry	13 (81)
Prior, none at study entry	1 (6)
Never	2 (13)
Prior lines of therapy (median, minimum–maximum)	3 (2–5)
Prior fluoropyrimidine	​
Yes	16 (100)
No	0
Prior oxaliplatin	​
Yes	16 (100)
No	0
Prior irinotecan	​
Yes	16 (100)
No	0
Prior bevacizumab	​
Yes	14 (88)
No	2 (13)
Prior EGFR inhibitor	​
Yes	8 (50)
No	8 (50)
Prior regorafenib	​
Yes	1 (6)
No	15 (94)
Prior trifluridine/tipiracil	​
Yes	4 (25)
No	12 (75)

Abbreviations: dMMR, deficient MMR; MSI-H, microsatellite instability–high.

### Safety and tolerability

Two evorpacept dose levels were evaluated (15 and 10 mg/kg weekly); one patient had a subsequent dose reduction. No patients required cetuximab dose reduction. All patients experienced at least one treatment-emergent AE. AEs were grade 1 to 2 in 5 (31%), grade 3 to 4 in 8 (50%), and grade 5 in 3 (19%) patients. One DLT occurred in stage 1 in the initial cohort of patients treated at evorpacept dose level 1 (*N* = 6; 15 mg/kg weekly; grade 5 HLH). There were no DLTs at evorpacept dose level −1 (*N* = 3; 10 mg/kg weekly) and in the second cohort of patients treated at evorpacept dose level 1 (*N* = 3). The study proceeded to stage 2 with the evorpacept RD at dose level 1 (*N* = 4; 15 mg/kg weekly), meeting the co-primary objective.

Table 2 summarizes the most common treatment-emergent AEs per patient. Headache, acneiform rash, and fatigue were the most common low-grade AEs. Most anemia events were low-grade, except for one grade 4 event which co-occurred with HLH in patient 30-003. Among the grade 3 to 5 treatment-emergent AEs, the most common were dyspnea, hypoxia, and hypotension. Treatment-related AEs are presented in Supplementary Table S4, and are overall similar to treatment-emergent AEs.

**Table 2.** Treatment-emergent adverse events by Common Terminology Criteria for Adverse Events v5.0, grade 1–4 occurring in at least 10% of patients and all grade 5, at the patient level.

Adverse event term	Grade 1/2 (%)	Grade 3/4 (%)	Grade 5 (%)	Total (%)
Headache	5 (31)	1 (6)	0	6 (38)
Acneiform rash	5 (31)	0	0	5 (31)
Fatigue	5 (31)	0	0	5 (31)
Anemia	3 (19)	1 (6)	0	4 (25)
Diarrhea	4 (25)	0	0	4 (25)
Dyspnea	1 (6)	2 (13)	0	3 (19)
Abdominal pain	2 (13)	1 (6)	0	3 (19)
Anorexia	3 (19)	0	0	3 (19)
Nausea	3 (19)	0	0	3 (19)
Vomiting	3 (19)	0	0	3 (19)
Hypomagnesemia	3 (19)	0	0	3 (19)
Hypoxia	0	2 (13)	0	2 (13)
Hypotension	0	2 (13)	0	2 (13)
Lymphocyte count decreased	1 (6)	1 (6)	0	2 (13)
Rash maculopapular	1 (6)	1 (6)	0	2 (13)
Fever	2 (13)	0	0	2 (13)
Hypokalemia	2 (13)	0	0	2 (13)
Infusion-related reaction	2 (13)	0	0	2 (13)
Blood bilirubin increased	2 (13)	0	0	2 (13)
Cough	2 (13)	0	0	2 (13)
Edema limbs	2 (13)	0	0	2 (13)
Hyperhidrosis	2 (13)	0	0	2 (13)
Oral mucositis	2 (13)	0	0	2 (13)
Proteinuria	2 (13)	0	0	2 (13)
Rash	2 (13)	0	0	2 (13)
Constipation	2 (13)	0	0	2 (13)
Dysuria	2 (13)	0	0	2 (13)
Hemophagocytic lymphohistiocytosis	0	0	1	1 (6)
CRS	0	0	1	1 (6)
Respiratory failure	0	0	1	1 (6)

The grade 5 events were HLH, CRS, and respiratory failure. Patient 30-003 experienced grade 5 HLH following cycle 1 day 8 treatment, including the HLH diagnostic criteria of splenomegaly, cytopenias, hemophagocytosis (bone marrow), elevated ferritin (>37,500 ng/mL; normal 24–336), and elevated soluble CD25 (soluble IL2 receptor α; 27,735 pg/mL; ref. 50), as well as hepatic and respiratory failure. Treatments included high-dose steroids, tocilizumab, and mycophenolate mofetil and were unfortunately unsuccessful. HLH was assessed as probably related to all three study drugs and possibly related to progressive cancer, as very high tumor burden, including in the liver and lungs, was a contributing factor as assessed by autopsy. Patient 36-004 experienced CRS following cycle 1 day 8 treatment, including fever, hypotension, and hypoxia. The ferritin (3,870 ng/mL; normal 24–336) and cytokine profiles [IL2 receptor α 6,266.1 pg/mL (normal 175.3–858.2), IL5 <2.1 pg/mL, IL6 59.7 pg/mL (normal ≤2.0), IL10 353.3 pg/mL (normal ≤2.8) and IFNγ 18.5 pg/mL (normal ≤4.2)] were moderately elevated. A confirmed infectious source was not identified. However, the patient opted to transition to comfort-focused care and did not receive anti-IL6 therapy or intensive care unit support. CRS was assessed as probably related to all three study drugs. Patient 34-005 experienced grade 5 respiratory failure after exiting the study, related to disease progression. Other than the two treatment-related grade 5 events, no patients exited the study because of treatment-related toxicity. The Data Safety Monitoring Committee reviewed the totality of the study data and recommended to terminate study accrual. At this time, one patient (36-002) elected to continue study after signing a revised informed consent form.

### Efficacy

**Figure 3.**
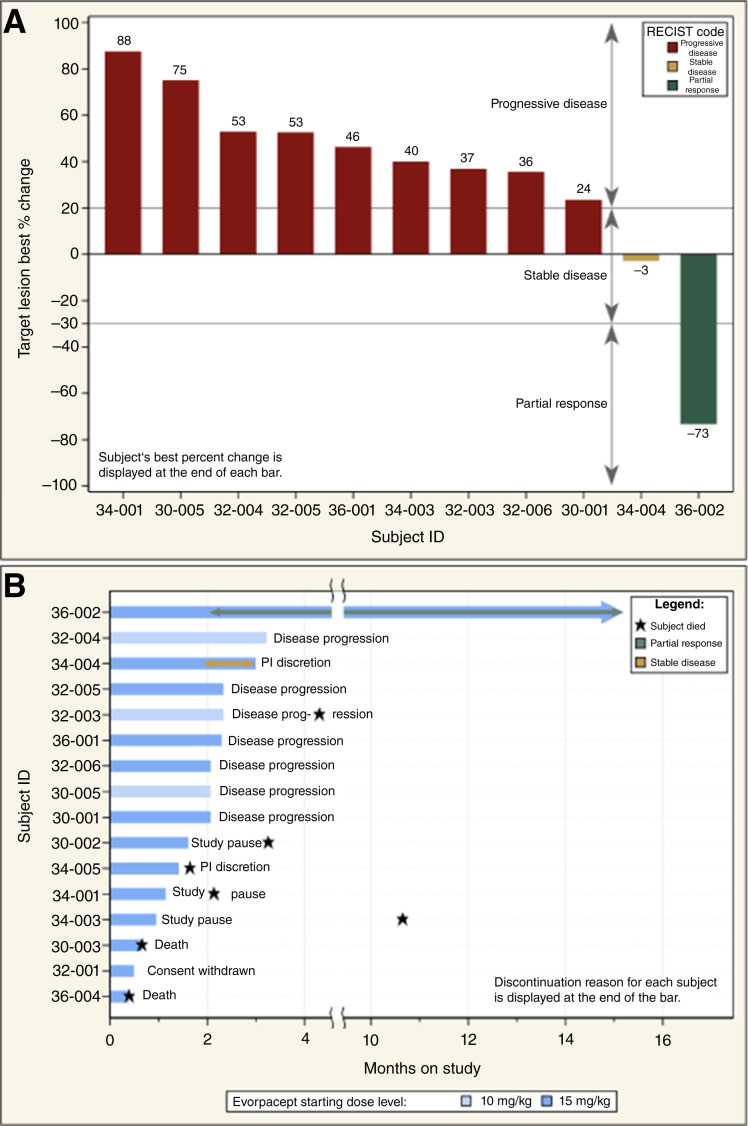
Depth and duration of response by subject. **A,** Waterfall plot of best percent change in aggregate size of target lesions. **B,** Swimmer plot of duration of response. As of the data cutoff, subject 36-002 remained on study. In (**A**), subject 30-002 is not included due to a 35% decrease in target lesions but unequivocal progression in nontarget lesions. PI, principal investigator.

The co-primary efficacy endpoint of this trial was ORR per RECIST v1.1. One patient (36-002) had a PR; the ORR was 6.3% (1/16; 95% CI, 0.2%–30.2%). Depth and duration of response are demonstrated in Fig. 3A and B. Formal hypothesis testing was not performed, as study enrollment was halted prior to full accrual. One additional patient had stable disease; the DCR was 12.5% (2/16; 95% CI, 1.6%–38.3%). The observed median PFS was 2.3 months (95% CI, 1.9–2.7 months; Supplementary Fig. S6A) and median OS was 10.9 months (95% CI, 3.3 months–infinity; Supplementary Fig. S6B). One patient continued treatment beyond disease progression by RECIST v1.1 and experienced further disease progression on the next scan by iRECIST. After this trial, *N* = 7 patients received additional systemic therapy, and the median number of subsequent lines of therapy was 1 (min–max 1–3). At the time of data pull, survival follow-up was ongoing.

The patient with PR (36-002) presented with sigmoid adenocarcinoma metastatic to a single site in the liver and retroperitoneal lymphadenopathy at diagnosis. The tumor was *KRAS*, *NRAS*, and *BRAF* WT, and the tumor mutational burden was 6 Mut/Mb. The patient previously underwent ablation of the single liver metastasis (approximately 1.5 years prior to study entry) and progressed on a cetuximab-containing regimen immediately prior to trial enrollment. At study entry, the only site of disease was the lungs. As of the data pull, patient 36-002 remained on study with a duration of response of 13.2 months, and the carcinoembryonic antigen level declined from 22.6 ng/mL at baseline to 5.8 ng/mL. The patient with stable disease (34-004) presented with sigmoid adenocarcinoma metastatic to liver at diagnosis. The tumor was *KRAS*, *NRAS*, and *BRAF* WT, and tumor mutational burden was 4 Mut/Mb. The patient progressed on a cetuximab-containing regimen immediately prior to trial enrollment. At study entry, sites of disease were the liver, lungs, and LNs, and the carcinoembryonic antigen level was normal. The patient exited the study with ongoing stable disease to pursue palliative radiation. Among the 11 patients with complete results available for *KRAS*, *NRAS*, and *BRAF* (Table 1), the ORR and DCR were 14.3% and 28.6% for patients with *RAS*/*BRAF* WT, respectively, and 0% and 0% for patients with *RAS* or *BRAF* mutant, respectively. In patients with liver metastases at study entry (*N* = 13), the ORR was 0% and the DCR was 7.7% (one patient with SD). Among the three patients without liver metastases at study entry, the ORR and DCR were both 33.3% (one patient with PR).

### Correlative analyses

**Figure 4.**
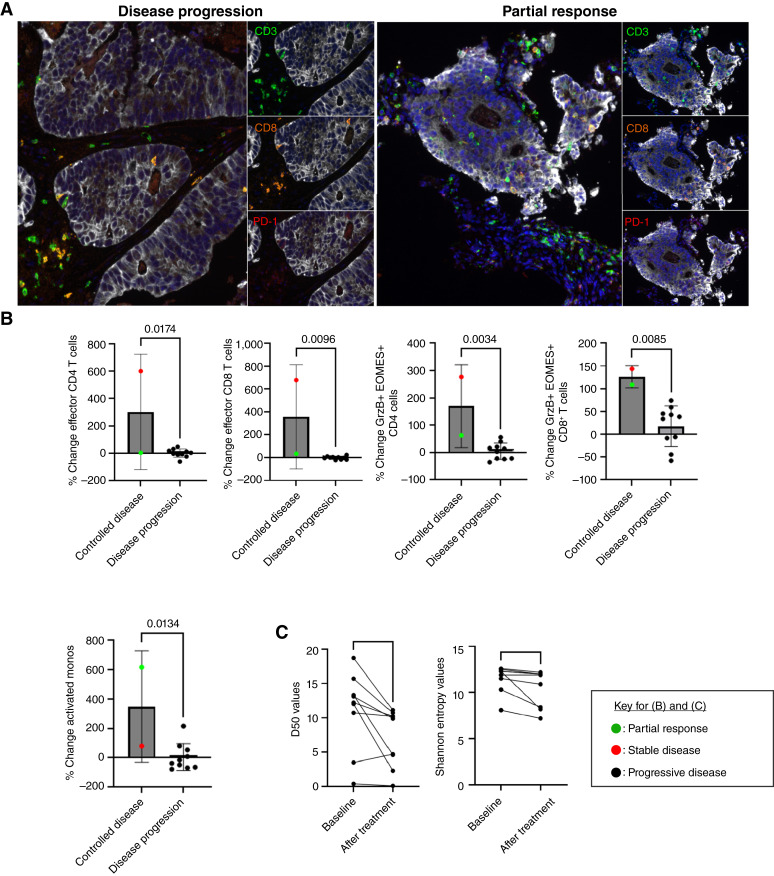
Pharmacodynamic analyses. **A,** Multiplex IHC of archival tumor tissue from one patient with disease progression and one patient with PR; **B,** Percent change (cycle 3 day 1 compared with baseline) in peripheral blood effector CD4^+^ (CD4^+^CD3^+^CD45RA^−^CD45RO^−^) and CD8^+^ (CD8^+^CD3^+^CD45RA^−^CD45RO^−^) T cells, granzyme B+ EOMES+ CD4^+^ (CD4^+^CD3^+^GrzB+EOMES+) and CD8^+^ (CD8^+^CD3^+^GrzB+EOMES+) T cells, and activated monocytes (CD11c^+^HLADR+CD16^+^) in patients with controlled disease (PR or stable disease) as best response compared with disease progression; (**C**) TCR diversity at baseline and after treatment (cycle 3 day 1 or cycle 4 day 1). GrzB, granzyme B. Statistics of linear correlation are provided; *, *P* < 0.05.

Due to early termination of study enrollment, no patients had paired (before and after treatment) tumor biopsies available for analysis. Two patients had usable archival tissue available (32-006 with disease progression as best response, tumor tissue obtained approximately 1 year prior to study entry; 36-002 with ongoing PR, tumor tissue obtained approximately 2 months prior to study entry). Descriptively, in the tumor with disease progression, CD3^+^ and CD8^+^ T cells were present in the surrounding stroma but were excluded from the tumor. On the contrary, there were numerous CD3^+^ and CD8^+^ T cells in the tumor with PR (Fig. 4A). This was further supported by the qualitative finding that in the tumor with disease progression, there was an increased distance between tumor cells and CD4^+^ or CD8^+^ T cells, compared with the tumor with PR. PD1 expression was low in both tumors (Supplementary Fig. S7). Due to limited tumor tissue availability, baseline EGFR, CD47, and PD-L1 expression were uninformative.

Immune cells in the peripheral blood from all study participants with available samples (*N* = 15) were analyzed at baseline and cycle 3 day 1 by mass cytometry (Supplementary Fig. S8). Patients with controlled disease (stable disease or PR) as best response, compared with those with disease progression, demonstrated an increase in effector CD4^+^ and CD8^+^ T cells, cytotoxic (granzyme B+ EOMES+) CD4^+^ and CD8^+^ T cells, and activated (CD16^+^) monocytes (Fig. 4B; Supplementary Figs. S9 and S10). In all patients with available samples (*N* = 9, all with disease progression as best response), there was a decrease in TCR diversity before versus after treatment, as evidenced by the decreased D50 and Shannon entropy values (Fig. 4C). This suggests that T-cell clonal expansion occurred following triple therapy, although the antigen specificity is unknown.

## Discussion

Immune checkpoint inhibitors targeting PD-(L)1 and CTLA-4 have revolutionized the treatment of deficient MMR/microsatellite instability–high colorectal cancer. However, in refractory MSS colorectal cancer, numerous trials using these agents alone and in combination with tyrosine kinase inhibitors and antiangiogenic therapy have unfortunately failed to produce practice-changing results, many of which we recently summarized (51). We aimed to evaluate a novel treatment regimen for this population by blocking the CD47/SIRP “do not eat me” signal between tumor cells and phagocytes with evorpacept, providing a tumor-directed pro-phagocytic opsonizing signal with cetuximab and synergistically activating the adaptive immune system with pembrolizumab. We hypothesized that reducing myeloid immunosuppression associated with refractory MSS colorectal cancer with synergistic innate and adaptive antitumor immunity could be effective (14, 21).

Among the 16 patients treated in our trial, the ORR was 6.3%, DCR 12.5%, median PFS 2.3 months, and median OS 10.9 months. These results must be interpreted with caution in the context of limited sample size due to early termination of study enrollment. Despite this limitation, signals of efficacy were observed, and our HIS-PDX likewise showed decreased tumor growth following treatment with triple therapy. One patient experienced deep (73% tumor shrinkage by RECIST v1.1) and durable PR (ongoing for 13.2 months at data pull). This patient did not have active liver metastases at study entry, consistent with improved efficacy of other immunotherapy regimens in non–liver-metastatic MSS colorectal cancer (52–54). Another patient experienced stable disease as best response. Both of these patients were *RAS*/*BRAF* WT and had disease progression on a cetuximab-containing regimen immediately prior to study entry, reducing the possibility that disease control in this trial was a result of cetuximab alone (55). Furthermore, the duration of PR is much longer than would typically be observed with EGFR inhibitor rechallenge (56), supporting a synergistic mechanism of evorpacept, cetuximab, and pembrolizumab. One prior study evaluated the anti-CD47 humanized IgG4 antibody magrolimab with cetuximab in previously treated colorectal cancer, and the ORR of 2.6% was similar to our trial (57). Immune checkpoint inhibition (PD-1 ± CTLA-4) with anti-EGFR demonstrated activity in previously treated colorectal cancer in two prior studies, with ORR 8% to 35% and median PFS 3.6 to 5.7 months (58, 59). Whether these results are better than what could be achieved by EGFR inhibitor rechallenge alone are unknown (56).

Treatment was tolerated as expected, except for two treatment-related grade 5 events. Headache and acneiform rash were common, as expected with cetuximab, as was fatigue, as expected in a refractory colorectal cancer population (60). Anemia, which has been frequently observed with other CD47 blockers, only occurred in 25% of patients in this trial and was low-grade in all cases except one (co-occurring with HLH), consistent with evorpacept’s inactive Fc domain and prior data (16, 61). Grade 5 HLH and CRS prompted early termination of study enrollment. HLH is a hyperinflammatory syndrome that is believed to be related to activated macrophages and lymphocytes with absence of downregulation, leading to cytokine secretion, tissue damage, and multi-organ failure (62). HLH has rarely been reported to occur following treatment with immune checkpoint inhibitors targeting PD-(L)1 and CTLA-4, is listed in the pembrolizumab FDA label, and in case reports, most patients recovered following immunosuppressive treatment (50, 63–65). HLH has not previously been reported with either cetuximab or evorpacept, but fatal HLH has been reported with magrolimab (66). Prior studies have suggested that the SIRPα/CD47 “do not eat me” axis must be blocked and a proinflammatory cytokine state be present in order for macrophages to phagocytose normal human cells and secondary HLH to develop (67, 68). This is a plausible explanation for patient 36-004 in this study. We assessed that HLH was probably related to all three drugs given the hypothesized mechanism of action, but the specific contribution of drug components and progressive cancer with high tumor burden is unknown.

Cytokine and ferritin levels generally correlate with the severity of CRS in patients receiving chimeric antigen receptor T-cell therapy, with severe CRS often associated with ferritin >100,000 ng/mL and elevations of certain cytokines ≥75-fold (69, 70). The ferritin and cytokine values for the patient who experienced grade 5 CRS in this study were only moderately elevated, which may reflect an initially less severe AE that became fatal in the context of the patient’s decision to transition to comfort care rather than pursue further CRS-directed treatments. CRS has rarely been reported with evorpacept (*N* = 1 patient with reversible grade 3 CRS) and pembrolizumab but not with cetuximab, and increased cytokine secretion has been observed in preclinical models of combined CD47 and PD-L1 inhibition (26, 65, 71–73). Similar to the case of HLH, contribution of each treatment is unknown, and the event was ultimately assessed as possibly related to all three drugs.

Our preclinical and clinical correlative studies provide additional insight regarding the mechanism of action of evorpacept/ALX90. In our HIS-PDX model, we found that ALX90 alone or in triple combination increased tumor-infiltrating CD8^+^ T cells but not T-cell cytotoxicity. This may be explained by our observation that ALX90 led to an increase in immunosuppressive M2 macrophages and loss of human T cells, presumably by phagocytosis, following blockade of the SIRPα–CD47 macrophage tolerance pathway. When we added LC to the triple therapy combination, we expected to observe abolished antitumor activity due to depletion of mononuclear phagocytes (i.e., the target of ALX90). However, we unexpectedly observed selective and incomplete myeloid depletion, with an increased M1:M2 macrophage ratio, increased neutrophil to macrophage ratio, and prevention of T-cell phagocytosis. In this more favorable TME, an antitumor T-cell response was unleashed, including increased tumor-infiltrating cytotoxic T cells and memory T cells and decreased tumor-infiltrating CD8^+^ Tregs. The results of our triple therapy with LC arm are consistent with prior data using ALX148 in syngeneic colorectal cancer models (21). Importantly, LC alone did not decrease SGR, demonstrating potential synergy of TME modulation with immunotherapy regimens. With or without LC, increased tumor-infiltrating neutrophils correlated with smaller tumors, whereas the opposite occurred for the overall macrophage population. This is consistent with known SIRPα expression on neutrophils and their ability to kill tumor cells following CD47/SIRPα blockade (74).

Blood- and tumor-based correlatives from our clinical trial are consistent with this preclinical data, demonstrating that triple therapy successfully activates both the innate (monocytes) and adaptive (effector and cytotoxic T cells) immune systems. We show evidence of clonal T-cell expansion, as measured by a decrease in TCR diversity following treatment, even in patients with disease progression as best response. Consistent with prior reports, more inflamed MSS colorectal cancer tumors at baseline may have improved response to immunotherapy regimens, which held true for the single responding patient in this study (75).

The mechanistic findings from our studies are overall consistent with prior evorpacept data (21). However, our preclinical finding that ALX90 increases immunosuppressive M2 macrophages and decreases human T cells, presumably by phagocytosis with CD47 blockade, was unexpected. In our preclinical experiment, T-cell depletion was much more prominent in tumors versus peripheral lymph organs. We hypothesize that SIRPα/CD47 blockade may cause phagocytosis of highly activated T cells dependent on the myeloid milieu, as would be expected to occur in the TME rather than peripheral lymph organs or peripheral blood. In fact, anti-CD47 can be used as a safety switch to eliminate chimeric antigen receptor T cells, supporting our notion (65). It is unknown whether this phenomenon occurs in humans; posttreatment tumor tissue was not available for more definitive assessment. Alternatively, this could be an artifact of the HIS-PDX model, which contains mouse myeloid cells and human T cells, perhaps increasing the impact of SIRPα–CD47 macrophage tolerance pathway loss or increased pro-phagocytosis signals between the xenogeneic interaction. In the evorpacept first-in-human trial, limited posttreatment biopsies showed an increase in macrophages (without further subtyping) and no change in CD8^+^ T cells (27). We hypothesize that combining CD47 blockade with therapies that further reduce myeloid immunosuppression, a key mediator of immunotherapy resistance in MSS colorectal cancer (14, 15), such as KRAS inhibitor, STING agonist, TLR9 agonist, GM-CSF, or inflammasome modulation, could be effective (76–80).

Limitations of our studies include the small number of patients treated in the clinical trial, restricting conclusions regarding efficacy, including the impact of liver metastases. Lack of sufficient tumor tissue from the clinical trial precluded correlation of baseline PD-L1, EGFR, and CD47 expression with response, and precluded posttreatment assessments. Determination of the contribution of components on PR and fatal adverse events is also not possible. Limitations of the HIS-PDX model have been extensively described (30). Most relevant to our experiment is the mixed mouse and human myeloid compartment, which could have contributed to our finding that ALX90 caused phagocytosis of human T cells (i.e., loss of the CD47 tolerance pathway between allogeneic mouse macrophages and human T cells). It is thus unclear whether this finding is restricted to this model or is biologically sound and is recapitulated in human tissues with highly activated T cells.

### Conclusion

The triple combination of evorpacept (ALX90 preclinically), cetuximab, and pembrolizumab demonstrated tumor response in a phase II clinical and HIS-PDX experiment in refractory MSS colorectal cancer, including a patient with a deep and durable PR. However, immune-related toxicity concerns, HLH and CRS, arose in the clinical trial, prompting early termination of study enrollment. This elucidates the importance of achieving immune activation within a therapeutic window when evaluating myeloid-targeted therapies. Biomarker analyses showed that triple therapy activated both innate and adaptive antitumor immunity, consistent with existing data. A potential mechanism of resistance to this regimen is an increase in immunosuppressive M2 macrophages. Although triple therapy may be limited by toxicity, evorpacept in double combination, potentially with another agent known to modulate immunosuppressive macrophages, could be worth evaluating in MSS colorectal cancer.

## Supplementary Material

Supplementary Methods Supplementary Methods

Supplementary Table 1 Supplementary Table 1. Flow cytometric Abs and Reagents

Supplementary Table 2 Supplementary Table 2. Representativeness of Study Participants

Supplementary Table 3 Supplementary Table 3. Mass Cytometry Antibodies and Metals

Supplementary Table 4 Supplementary Table 4. Treatment-related adverse events by CTCAE v5.0, grade 1-4 occurring in at least 10% of patients and all grade 5, at the patient level.

Supplementary Figure S1 Supplementary Figure S1. HIS-BRGS-PDX experiment schema and chimerism.

Supplementary Figure S2 Supplementary Figure S2. Human and murine immune system in peripheral lymphatic organs and tumor of HIS-BRGS mice bearing CRC307P PDX.

Supplementary Figure S3 Supplementary Figure S3. Triple therapy (ALX90, cetuximab, and pembrolizumab) activates human T cells and slows tumor growth of CRC307P CRC MSS PDX in HIS-BRGS mice with liposomal clodronate treatment (LC).

Supplementary Figure S4 Supplementary Figure S4. T cell subsets in peripheral lymphatic organs and tumor properties of HIS-BRGS mice bearing CRC307P PDX.

Supplementary Figure S5 Supplementary Figure S5. CONSORT

Supplementary Figure S6 Supplementary Figure S6. Survival outcomes of the trial.

Supplementary Figure S7 Supplementary Figure S7. Distance between tumor cells and CD4+ and CD8+ T cells in archival tumor tissue by immunohistochemistry.

Supplementary Figure S8 Supplementary Figure S8. Percent change in major peripheral blood cell populations by mass cytometry at Cycle 3 Day 1 versus baseline.

Supplementary Figure S9 Supplementary Figure S9. Pie chart of peripheral blood mass cytometry.

Supplementary Figure S10 Supplementary Figure S10. Serial peripheral blood mass cytometry by patient.

Protocol Protocol

## Data Availability

The data are available upon request from the corresponding author and will be available on ClinicalTrials.gov (NCT05167409).
