# Supplementary material for: Phase II Clinical Trial and Preclinical Evaluation of a Novel CD47 Blockade Combination in Refractory Microsatellite-Stable Metastatic Colorectal Cancer
Source: Cancer Res Commun. 2025 Nov 20;5(11):2039–52. doi: 10.1158/2767-9764.CRC-25-0332 (PMC12631056; doi:10.1158/2767-9764.CRC-25-0332)
Supplement: Supplementary Methods [file crc-25-0332_supplementary_methods_suppsm.docx]

**Preclinical Human Immune System Murine Experiment***PDX Generation*

Animal work was approved by the University of Colorado Anschutz Medical Campus Institutional Animal Care and Use Committee (#00021). Tumor samples from patients were previously obtained from patients at the University of Colorado Cancer Center under an approved protocol by the Colorado Multiple Institutional Review Board (COMIRB #08-0439). PDX models were generated as previously described. Athymic nude mice (females ages 6-8 weeks) were obtained from Envigo (Indianapolis, IN); tumors approximately 3 mm^3^ were implanted subcutaneously on the bilateral hind flanks. Following a period of growth, tumors were harvested at approximately 1500-2000 mm^3^ and used to generate HIS-BRGS-PDX mice.

*HIS-BRGS Mouse Studies*

HIS-BRGS mice were generated as shown in **Supplementary Figure 1A** and as previously described (Lang et al, 2011; Marin-Jimenez et al, 2021). In brief, CD34+ hematopoietic stem cells (HSC) were isolated from a single cord blood (CB) unit deemed unfit for CB banking from Clinimmune under an approved protocol (COMIRB #16-0541). Following 300 rad irradiation, neonatal BRGS mice were injected with CD34+ HSCs in the facial vein and liver. Human chimerism was measured 10 and 14 weeks later using flow cytometry to confirm engraftment. MSS CRC PDX tumors (CRC307P; KRAS G12S; tumor derived from primary colon mass in a patient also with liver metastases, previously exposed to EGFR inhibitor) were implanted into both flanks of 37 mixed male and female HIS-BRGS mice between 19-28 weeks of age. Fourteen mice received LC 100 μl weekly intraperitoneal starting one week prior to other treatments. Mice were randomized into one of six treatment groups based on chimerism: vehicle, vehicle + LC, ALX90 (preclinical surrogate for evorpacept with a similar modified/inactive Fc domain and binding properties, with mouse and human cross-reactivity), cetuximab + pembrolizumab, ALX90 + cetuximab + pembrolizumab (triple therapy), or triple therapy + LC (**Supplementary Figure 1B**). Previous studies with anti-PD-1 agents alone in this PDX model have shown no efficacy. Twice weekly, tumors were measured, weights were recorded, and health assessment was performed; supplemental food was given daily. Treatment began 25 days following tumor implantation when the tumor sizes reached approximately 50-300 mm^3^. Mice were harvested 11-25 days following start of treatment based on health and tumor size. At harvest, lymph nodes (LN), spleen (SP), and tumors were collected and processed into single-cell suspensions as previously described for flow cytometric analysis (Marin-Jimenez et al, 2021). Additional details regarding the HIS-BRGS mouse model and experimental procedures are described in detail in prior publications from our group (Marin-Jimenez et al, 2021; Capasso et al, 2019; Lang et al, 2013; Lang et al, 2022). The dose of ALX90 was 30 mg/kg twice weekly intraperitoneal, the dose of pembrolizumab was 15 mg/kg weekly intraperitoneal, and the dose of cetuximab was 0.004 mg/mouse twice weekly intraperitoneal. Diluents were sterile saline for cetuximab, pembrolizumab, and liposomal clodronate, and PBS for ALX90.

*Flow Cytometry and Analysis*

Single-cell suspensions of LNs, spleens, and tumors were stained with fluorescently-labeled antibodies (Abs) to evaluate mouse and human immune cell subsets as previously described and as listed in **Supplementary Table 1** (Capasso et al, 2019; Marin-Jimenez et al, 2021). Four panels were used to analyze the immune subsets and run on a Biorad Yeti 5-laser (3) or a Cytek Aurora (1) for a set time and rate to enumerate cell counts. We evaluated 1) overall human and mouse immune subsets, 2) activated (HLA-DR+), memory (CD45RA-), immunosuppressed (TIGIT+ and PD-1+ inhibitory receptors), regulatory (CD25+FoxP3+), and cytotoxic (Granzyme B+, IFNγ+ and TNFα+) human T cells, 3) mouse subsets including polymorphonuclear (PMN) cells (Ly6G+), macrophages (MO, F4/80+) including M1 (CD38+) and M2 (CD206+), and MHC class I and II expression on the PMN and MO populations. We analyzed LN, spleens, and tumors to assess systemic immune and tumor-specific changes. For detection of intracellular IFNγ and TNFα positive T cells, cell suspensions were stimulated overnight with Invitrogen Cell stim cocktail and Golgi plug added for the last 4 hours followed by staining and fixation with 1% paraformaldehyde solutions and permeabilization with saponin (1X PBS, 0.5% BSA and 0.5% Saponin) solution. Detection of Tregs was determined by extracellular staining of CD25 followed by staining for intracellular FoxP3 (and Granzyme B) using the eBioscience Transcription Factor intracellular staining kit. Data were analyzed using FlowJo 10.9 software (BD Biosciences, RRID: SCR_008520) and frequencies were exported for further statistical analyses.

*Tumor Growth Measurement*

Tumor volume was estimated using the formula: (length x width^2^) x 0.52. Tumor growth was assessed using specific growth rate (SGR), which was calculated as: $SGR=\ln\left( \frac{V2}{V1} \right)/(t2-t1)$, where volume = V and time = t. This assesses percent growth by volume per day. A negative SGR corresponds to a regressing tumor whereas a positive SGR corresponds to a growing tumor. Compared to tumor volume doubling time, SGR has been shown to be more accurate, and is appropriate for statistical testing (Mehrara et al, 2007; Mehrara et al, 2009).

*Statistics*

SGR and flow cytometry data was analyzed using GraphPad Prism version 10.4.0 (GraphPad Software, San Diego, California USA). For comparisons of means among three or more groups, Welch’s ANOVA test was used. For comparisons between two independent groups, unpaired t-test with Welch’s correction were conducted.

**Clinical Trial Exploratory Correlative Analyses**

*Multiplex Immunohistochemistry*

Multispectral imaging using the PhenoImager HT instrument (Akoya Biosciences) was performed as previously described (de Jong et al, 2023). To quantify levels of immune infiltrate, formalin-fixed paraffin-embedded tissue sections were stained consecutively with specific primary antibodies for EGFR, PDL1, Granzyme B, CD47, CD3, PD-1, CD8, and CK, horseradish peroxidase (HRP)-conjugated secondary antibody polymer, and HRP-reactive OPAL fluorescent reagents. The slides were stripped between each stain with heat treatment in antigen retrieval buffer. Whole slide scans were collected with PhenoImager HT v2.0.0 software using the 20x objective with a 0.5 micron resolution. Regions of interest were selected and rescanned using the 20x objective and multispectral imaging. Spectral references and unstained control images were measured and inForm software v3.0 was used to create a multispectral library reference. The multispectral images (.im3 files) were spectrally unmixed and analyzed with tissue segmentation, cell segmentation, and phenotyping using inForm software v3.0 (Akoya Biosciences) and data were compiled and summarized using PhenoptrReports (Akoya Biosciences).

*Mass cytometry*

Peripheral blood mononuclear cells were stained with a panel of antibodies (**Supplementary Table 3**) conjugated to heavy metal ions as previously described (Waugh et al, 2019). Briefly, samples were individually barcoded with palladium isotopes using the Cell-ID 20-Plex Pd Barcoding kit (Standard Biotools) and combined prior to staining with surface antibodies. Intracellular targets were stained after permeabilization using Transcription Factor Phospho Buffer Set (BD Pharmingen). Cells were incubated overnight with Cell-ID Intercalator-Ir (Standard Biotools) and collected on a CyTOF XT instrument (Standard Biotools). CyTOF Software v8.0 was used for debarcoding and data normalization with EQTM Six Element Calibration beads (Standard Biotools). Cell populations were identified with hierarchical gating using FlowJo v10.9 software (BD Pharmingen).

*TCR sequencing*

RNA was isolated from PBMC samples using RNeasy Kits (Qiagen). TCR beta chain libraries for V(D)J sequencing were prepared using iR-Complete Dual Index Primer Kits (iRepertoire). Libraries were sequenced using an Illumina MiSeq. Sequences were analyzed using the iR-Web platform (iRepertoire). In these analyses, the frequency of unique TCR clonotypes were calculated and the D50 values, representing the number of unique clonotypes that account for 50% of all sequencing reads, and the Shannon entropy values, a diversity index representing a measure of randomness, are reported.
