# Supplementary material for: Phase II Clinical Trial and Preclinical Evaluation of a Novel CD47 Blockade Combination in Refractory Microsatellite-Stable Metastatic Colorectal Cancer
Source: Cancer Res Commun. 2025 Nov 20;5(11):2039–52. doi: 10.1158/2767-9764.CRC-25-0332 (PMC12631056; doi:10.1158/2767-9764.CRC-25-0332)
Supplement: Supplementary Table 2 — Representativeness of Study Participants [file crc-25-0332_supplementary_table_2_suppst2.docx]

**Supplementary Table 2. Representativeness of Study Participants**

| Cancer type: Colorectal cancer (CRC) | |
| --- | --- |
| Considerations related to: | |
| Sex | The incidence and mortality of CRC is higher in men than women. The incidence of CRC is approximately 44 per 100,000 males and 34 per 100,000 females in the United States. The mortality of CRC is approximately 17 per 100,000 males and 12 per 100,000 females. |
| Age | The median age at CRC diagnosis is 66 years old in men and 69 years old in women. CRC patients are increasingly younger. The risk of CRC increases with age. For each 5-year age group, the incidence rate approximately doubles until the age of 50. |
| Race/ethnicity | CRC incidence and mortality are highest in non-Hispanic blacks, followed by American Indians and Alaska Natives, and lowest in Asians/Pacific Islanders. CRC incidence rates are approximately 20% higher in blacks than non-Hispanic whites. Larger disparities exist in CRC mortality, where the mortality rate for blocks is about 40% higher than in non-Hispanic whites. |
| Geography | The incidence of CRC in the Unites States is approximately 153,000 cases per year, while in Colorado it is approximately 2,100 cases per year. |
| Other considerations | Approximately 95% of patients with metastatic CRC have microsatellite stable/proficient mismatch repair tumors, which is the population included in the present study. |
| Overall representativeness of this study | The trial included more men (69%) than women (31%), limiting potential generalizability of results by sex.  The median age of patients in this trial (53 years) is lower than the median age at diagnosis, likely due to trial requirements to enroll fit patients (ECOG performance status 0-1). The protocol allowed patients 18 years of age or older to enroll.  This was a multi-site trial that enrolled patients from five diverse sites across the United States, improving generalizability. |

Source: Colorectal Cancer Facts & Figures 2020-2022, American Cancer Society, accessed 12/31/2024. <https://www.cancer.org/content/dam/cancer-org/research/cancer-facts-and-statistics/colorectal-cancer-facts-and-figures/colorectal-cancer-facts-and-figures-2020-2022.pdf>
