# Supplementary material for: Phase II Clinical Trial and Preclinical Evaluation of a Novel CD47 Blockade Combination in Refractory Microsatellite-Stable Metastatic Colorectal Cancer
Source: Cancer Res Commun. 2025 Nov 20;5(11):2039–52. doi: 10.1158/2767-9764.CRC-25-0332 (PMC12631056; doi:10.1158/2767-9764.CRC-25-0332)
Supplement: Supplementary Table 4 — Treatment-related adverse events by CTCAE v5.0, grade 1-4 occurring in at least 10% of patients and all grade 5, at the patient level. [file crc-25-0332_supplementary_table_4_suppst4.docx]

| **Adverse Event Term** | **Grade 1/2 (%)** | **Grade 3/4 (%)** | **Grade 5 (%)** | **Total (%)** |
| --- | --- | --- | --- | --- |
| Headache | 4 (25) | 1 (6) | 0 | 5 (31) |
| Acneiform Rash | 4 (25) | 0 | 0 | 4 (25) |
| Fatigue | 4 (25) | 0 | 0 | 4 (25) |
| Anemia | 2 (13) | 1 (6) | 0 | 3 (19) |
| Diarrhea | 3 (19) | 0 | 0 | 3 (19) |
| Hypomagnesemia | 3 (19) | 0 | 0 | 3 (19) |
| Nausea | 3 (19) | 0 | 0 | 3 (19) |
| Hypoxia | 0 | 2 (13) | 0 | 2 (13) |
| Rash Maculopapular | 1 (6) | 1 (6) | 0 | 2 (13) |
| Blood Bilirubin Increased | 2 (13) | 0 | 0 | 2 (13) |
| Fever | 2 (13) | 0 | 0 | 2 (13) |
| Hypokalemia | 2 (13) | 0 | 0 | 2 (13) |
| Infusion Related Reaction | 2 (13) | 0 | 0 | 2 (13) |
| Oral Mucositis | 2 (13) | 0 | 0 | 2 (13) |
| Rash | 2 (13) | 0 | 0 | 2 (13) |
| Vomiting | 2 (13) | 0 | 0 | 2 (13) |
| Hemophagocytic Lymphohistiocytosis | 0 | 0 | 1 | 1 (6) |
| Cytokine Release Syndrome | 0 | 0 | 1 | 1 (6) |

**Supplementary Table 4: Treatment-related adverse events by CTCAE v5.0, grade 1-4 occurring in at least 10% of patients and all grade 5, at the patient level.**
