# Supplementary material for: Phase II Clinical Trial and Preclinical Evaluation of a Novel CD47 Blockade Combination in Refractory Microsatellite-Stable Metastatic Colorectal Cancer
Source: Cancer Res Commun. 2025 Nov 20;5(11):2039–52. doi: 10.1158/2767-9764.CRC-25-0332 (PMC12631056; doi:10.1158/2767-9764.CRC-25-0332)
Supplement: Supplementary Figure S1 — HIS-BRGS-PDX experiment schema and chimerism. [file crc-25-0332_supplementary_figure_s1_suppsf1.docx]

**
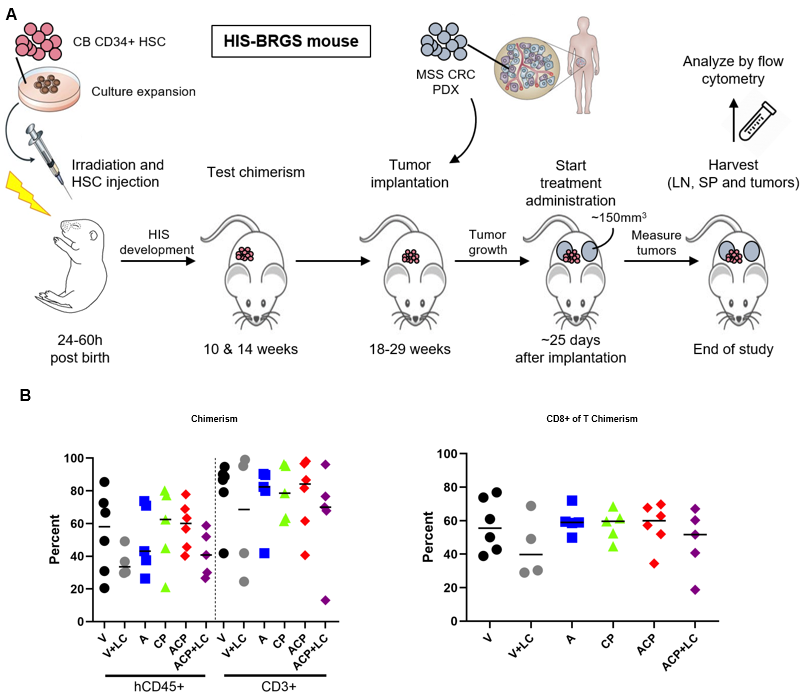
**

**S1**

**Supplementary Figure 1**. **HIS-BRGS-PDX experiment schema and chimerism.** (A) Schematic of HIS-BRGS-PDX generation, treatment, and organ harvest; modified with permission from Marin et al. (B) Human chimerism in blood of HIS-BRGS mice prior to tumor implantation. The percentage human (hCD45+) of mouse + human CD45+, human T cells (CD3+ of hCD45+), and human CD8+ T cells (hCD8+ of human T cells) were measured in PBMCs of mice at 15-17 weeks post-engraftment by flow cytometry. HIS-BRGS mice were allocated into experimental groups based on equivalent chimerism. Abbreviations: BRGS, BALB/c-Rag2^null^Il2rγ^null^Sirpα^NOD^; CB, cord blood; HIS, human immune system; HSC, hematopoietic stem cell; LN, lymph node; PDX, patient-derived xenograft; SP, spleen.
