# Supplementary material for: Phase II Clinical Trial and Preclinical Evaluation of a Novel CD47 Blockade Combination in Refractory Microsatellite-Stable Metastatic Colorectal Cancer
Source: Cancer Res Commun. 2025 Nov 20;5(11):2039–52. doi: 10.1158/2767-9764.CRC-25-0332 (PMC12631056; doi:10.1158/2767-9764.CRC-25-0332)
Supplement: Supplementary Figure S2 — Human and murine immune system in peripheral lymphatic organs and tumor of HIS-BRGS mice bearing CRC307P PDX. [file crc-25-0332_supplementary_figure_s2_suppsf2.docx]

**
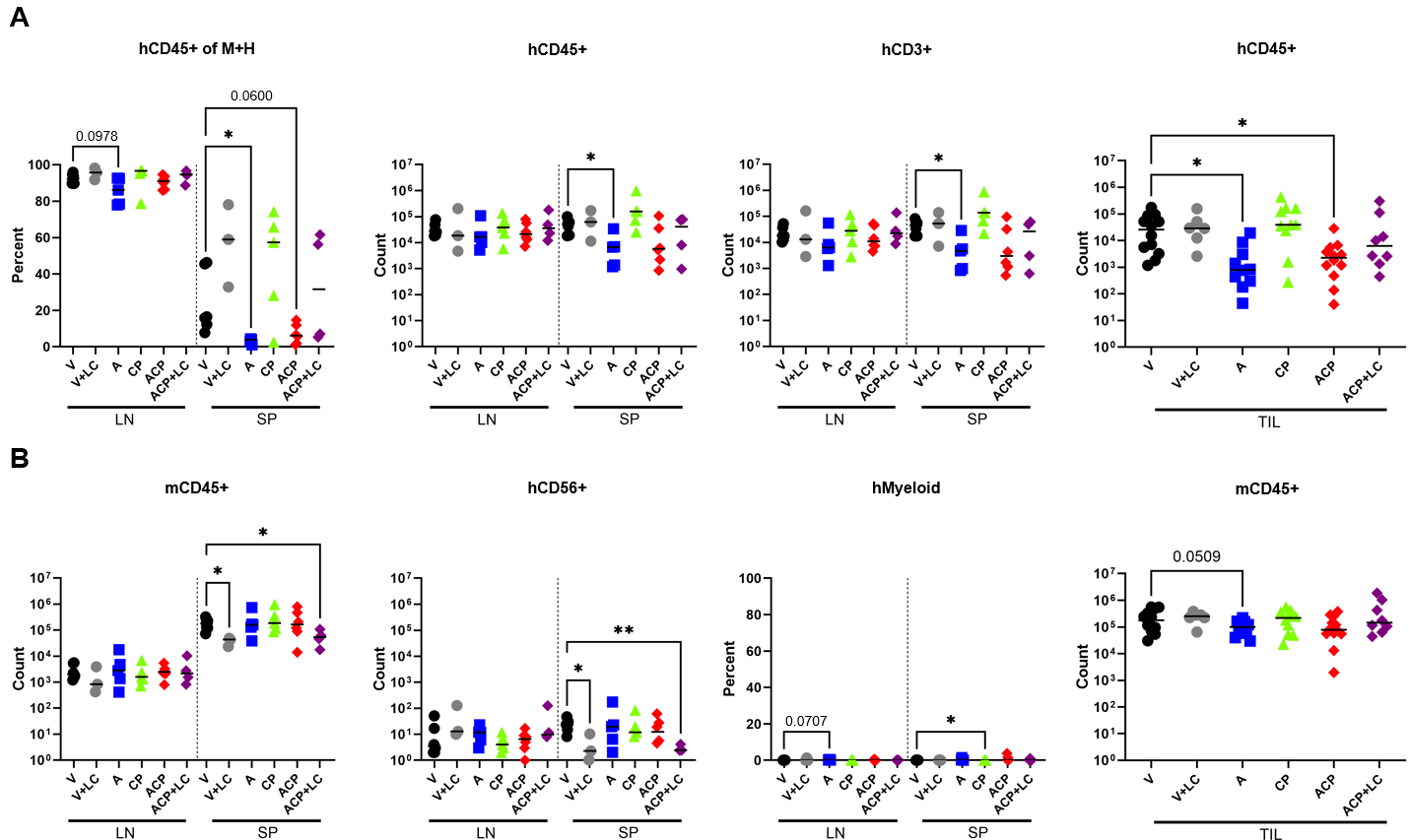
**

**S2**

**Supplementary Figure 2. Human and murine immune system in peripheral lymphatic organs and tumor of HIS-BRGS mice bearing CRC307P PDX.** (A) Loss of human immune cells in spleens and tumor of HIS-mice treated with ALX90 alone or in combination with cetuximab and pembrolizumab as measured by flow cytometry. (B) Liposomal clodronate treatment reduces the number of mouse myeloid (mCD45+) cells in spleens, but not TILs, and human NK (CD56+) cells in spleens of HIS-BRGS mice. hCD3+, CD56+, and myeloid (hCD11b, hCD14, hCD33 or hCD11c+) are a percentage of the overall hCD45+ gate.
