# Supplementary material for: Phase II Clinical Trial and Preclinical Evaluation of a Novel CD47 Blockade Combination in Refractory Microsatellite-Stable Metastatic Colorectal Cancer
Source: Cancer Res Commun. 2025 Nov 20;5(11):2039–52. doi: 10.1158/2767-9764.CRC-25-0332 (PMC12631056; doi:10.1158/2767-9764.CRC-25-0332)
Supplement: Supplementary Figure S3 — Triple therapy (ALX90, cetuximab, and pembrolizumab) activates human T cells and slows tumor growth of CRC307P CRC MSS PDX in HIS-BRGS mice with liposomal clodronate treatment (LC). [file crc-25-0332_supplementary_figure_s3_suppsf3.docx]

**
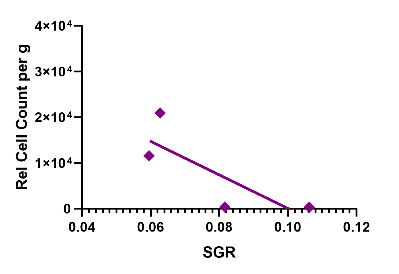

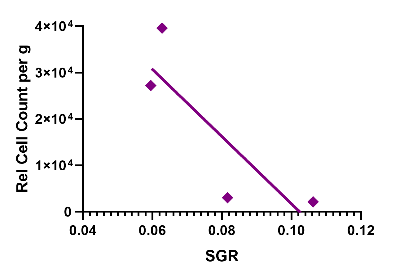

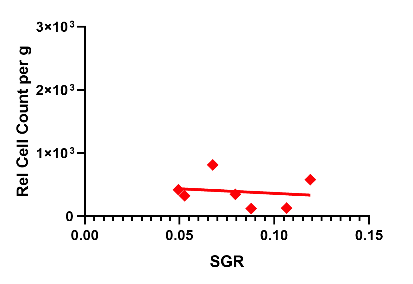

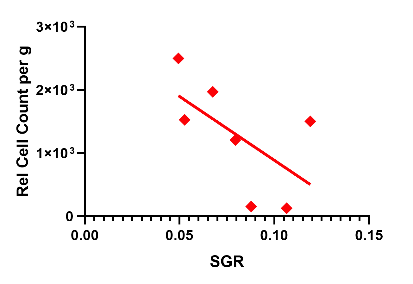

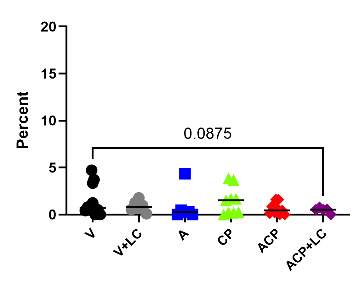

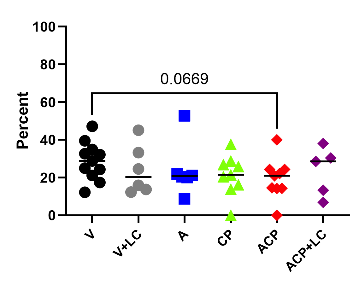

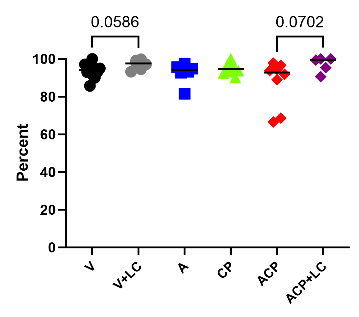

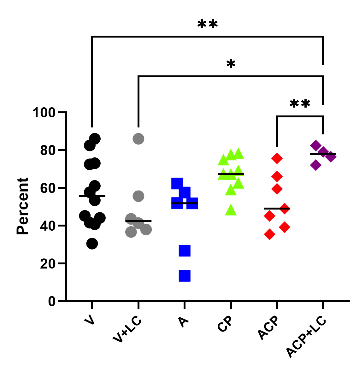

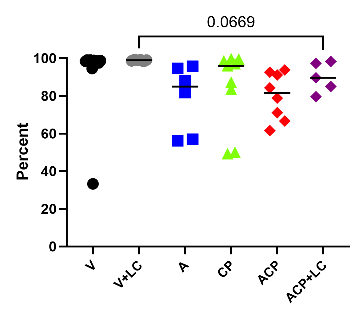

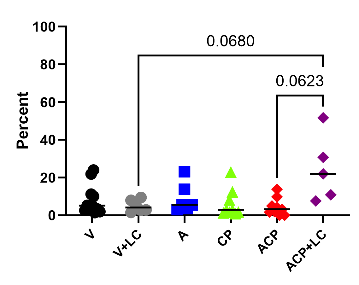
**

**S3**


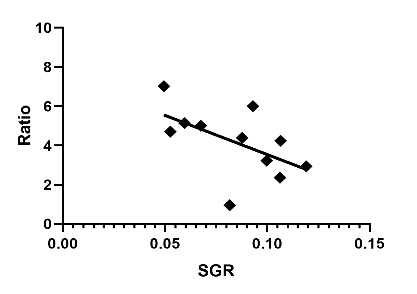


p= 0.078

**Tem/Tcm CD4+ ACP**$\boldsymbol{\pm}$**LC**


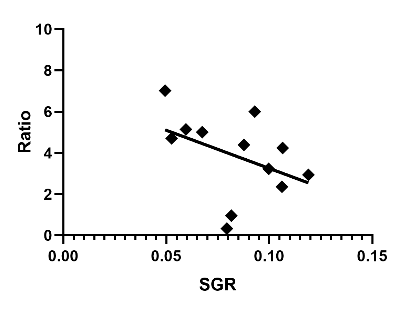


**Tem/Tcm CD8+ ACP**$\boldsymbol{\pm}$**LC**

**IFNγ+ CD8+ ACP+LC**

**TNFα+ CD4+ ACP+LC**

**IFNγ+ CD8+ ACP**

**TNFα+ CD4+ ACP**

**Treg CD8+**

**TNFα+ CD8+**

**GrB+ CD8+**

**Tmem CD8+**


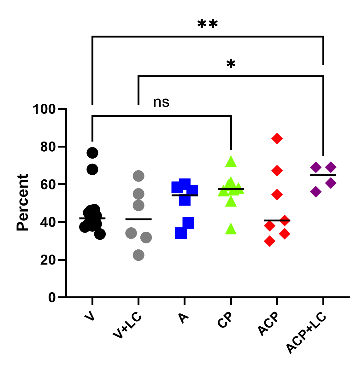


0.0572


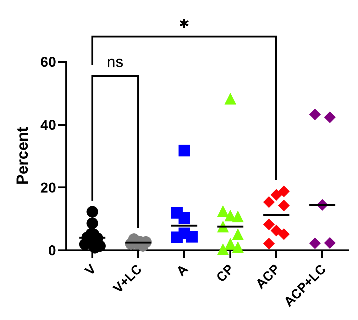


0.0606

**GrB+ CD4+**

**Tmem CD4+**

**Treg CD4+**

**IFNγ+ CD4+**

**A**

**Supplementary Figure 3. Triple therapy (ALX90, cetuximab, and pembrolizumab) activates human T cells and slows tumor growth of CRC307P CRC MSS PDX in HIS-BRGS mice with liposomal clodronate treatment (LC).** A) Phenotypes of CD4+ (top) and CD8+ (bottom) tumor infiltrating T cells as determined by flow cytometry: Memory (CD45RA-), Granzyme B, IFNγ, and TNFα cytotoxic T cells and Tregs (CD25+, FoxP3+). B) Immonophenotype correlation with tumor growth (SGR) among triple therapy (ACP, red) and triple therapy following LC treatments (ACP + LC, purple), and all mice treated with triple therapy with or without LC (ACP ± LC, black). Statistics of linear correlation are provided; *p<0.05, **p<0.001.
