# Supplementary material for: Phase II Clinical Trial and Preclinical Evaluation of a Novel CD47 Blockade Combination in Refractory Microsatellite-Stable Metastatic Colorectal Cancer
Source: Cancer Res Commun. 2025 Nov 20;5(11):2039–52. doi: 10.1158/2767-9764.CRC-25-0332 (PMC12631056; doi:10.1158/2767-9764.CRC-25-0332)
Supplement: Supplementary Figure S4 — T cell subsets in peripheral lymphatic organs and tumor properties of HIS-BRGS mice bearing CRC307P PDX. [file crc-25-0332_supplementary_figure_s4_suppsf4.docx]

**
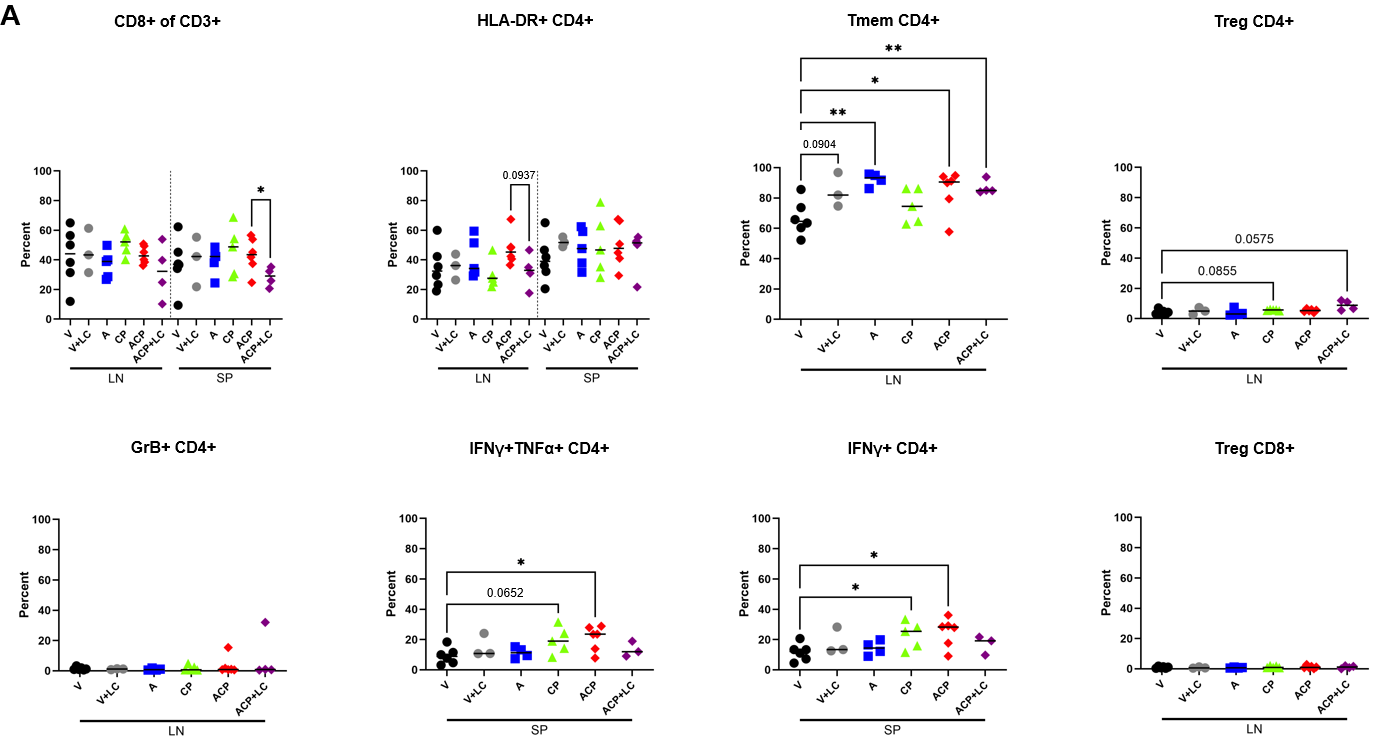
**

**S4**

**S4**

**
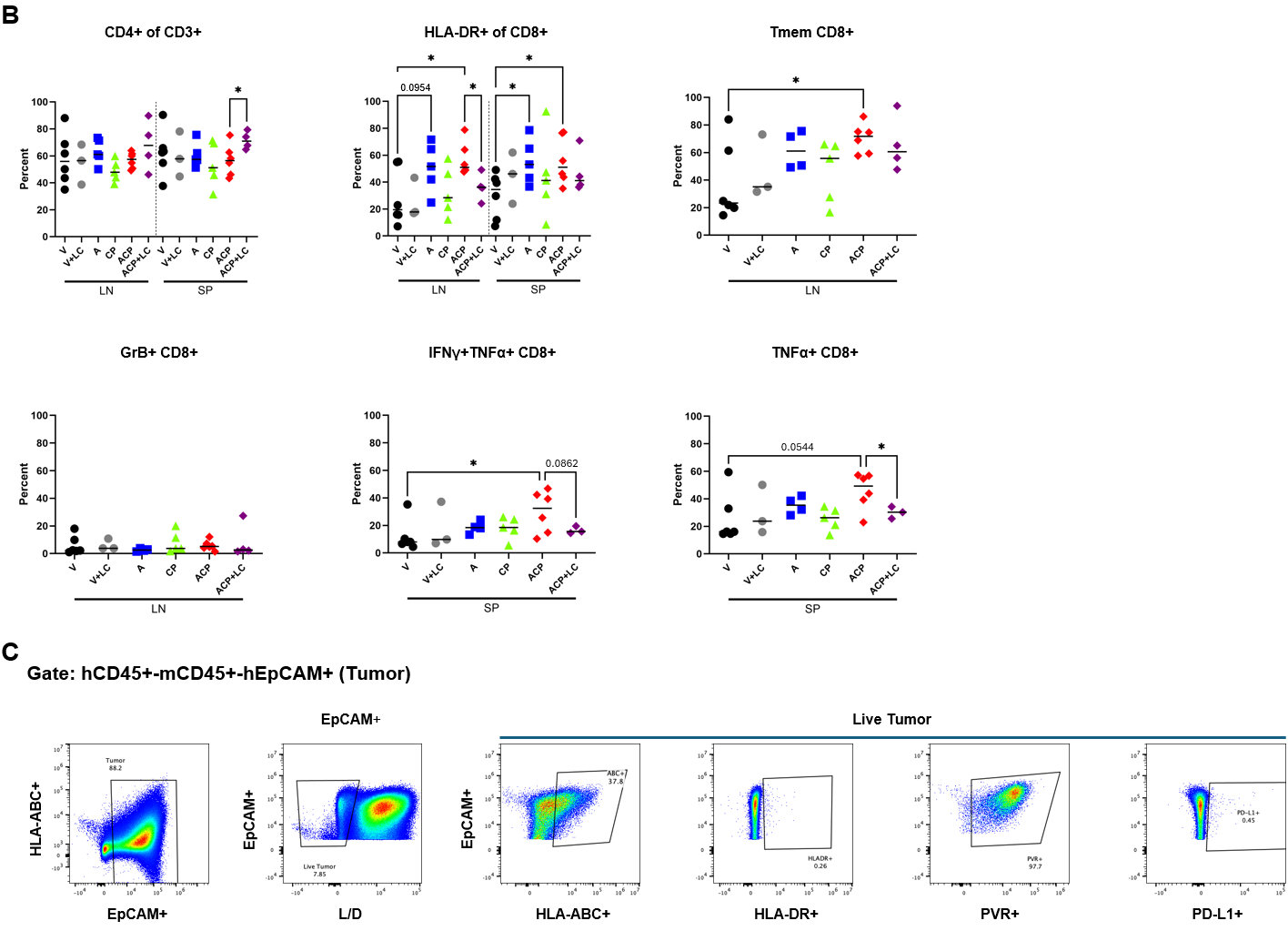
**

**S4**

**Supplementary Figure 4. T cell subsets in peripheral lymphatic organs and tumor properties of HIS-BRGS mice bearing CRC307P PDX.** Activated and cytotoxic CD4+ (A) and CD8+ (B) T cells in peripheral lymph nodes and spleen of HIS-BRGS mice. Frequencies of CD4+ and CD8+ T cells, as well as activation (HLA-DR+), memory (CD45RA-), cytotoxic (Granzyme B+, IFNγ+ and TNFα+) and Treg (CD25+, FoxP3+) markers, were measured by flow cytometry. C) The CRC307P PDX has high HLA-ABC class I and PVR expression but low PD-L1 and HLA-class II as determined by flow cytometry, gating on EpCAM+ human tumor cells.
