# Supplementary material for: Phase II Clinical Trial and Preclinical Evaluation of a Novel CD47 Blockade Combination in Refractory Microsatellite-Stable Metastatic Colorectal Cancer
Source: Cancer Res Commun. 2025 Nov 20;5(11):2039–52. doi: 10.1158/2767-9764.CRC-25-0332 (PMC12631056; doi:10.1158/2767-9764.CRC-25-0332)
Supplement: Supplementary Figure S5 — CONSORT [file crc-25-0332_supplementary_figure_s5_suppsf5.docx]

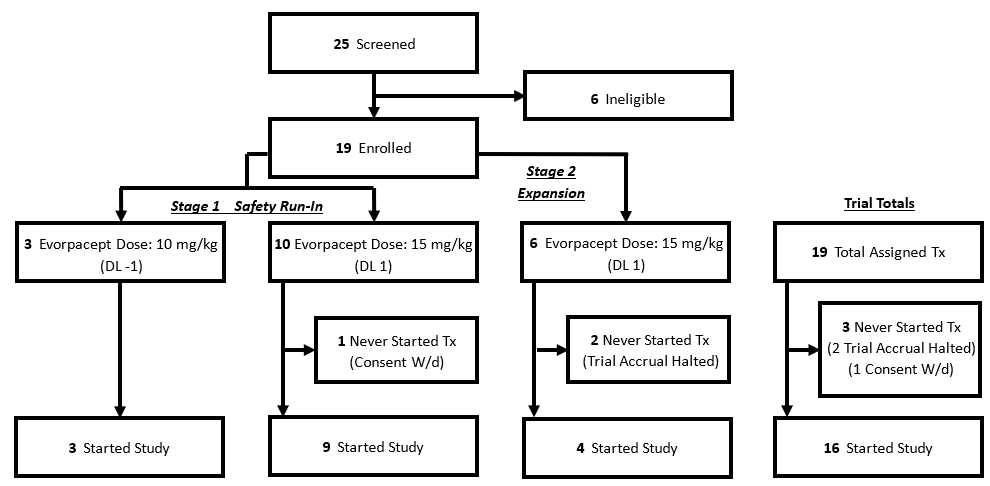


**S5**

**Supplementary Figure 5: CONSORT**Note: All patients received cetuximab and pembrolizumab in combination with evorpacept. Abbreviations: DL, dose level.
