# Supplementary material for: Phase II Clinical Trial and Preclinical Evaluation of a Novel CD47 Blockade Combination in Refractory Microsatellite-Stable Metastatic Colorectal Cancer
Source: Cancer Res Commun. 2025 Nov 20;5(11):2039–52. doi: 10.1158/2767-9764.CRC-25-0332 (PMC12631056; doi:10.1158/2767-9764.CRC-25-0332)
Supplement: Supplementary Figure S6 — Survival outcomes of the trial. [file crc-25-0332_supplementary_figure_s6_suppsf6.docx]

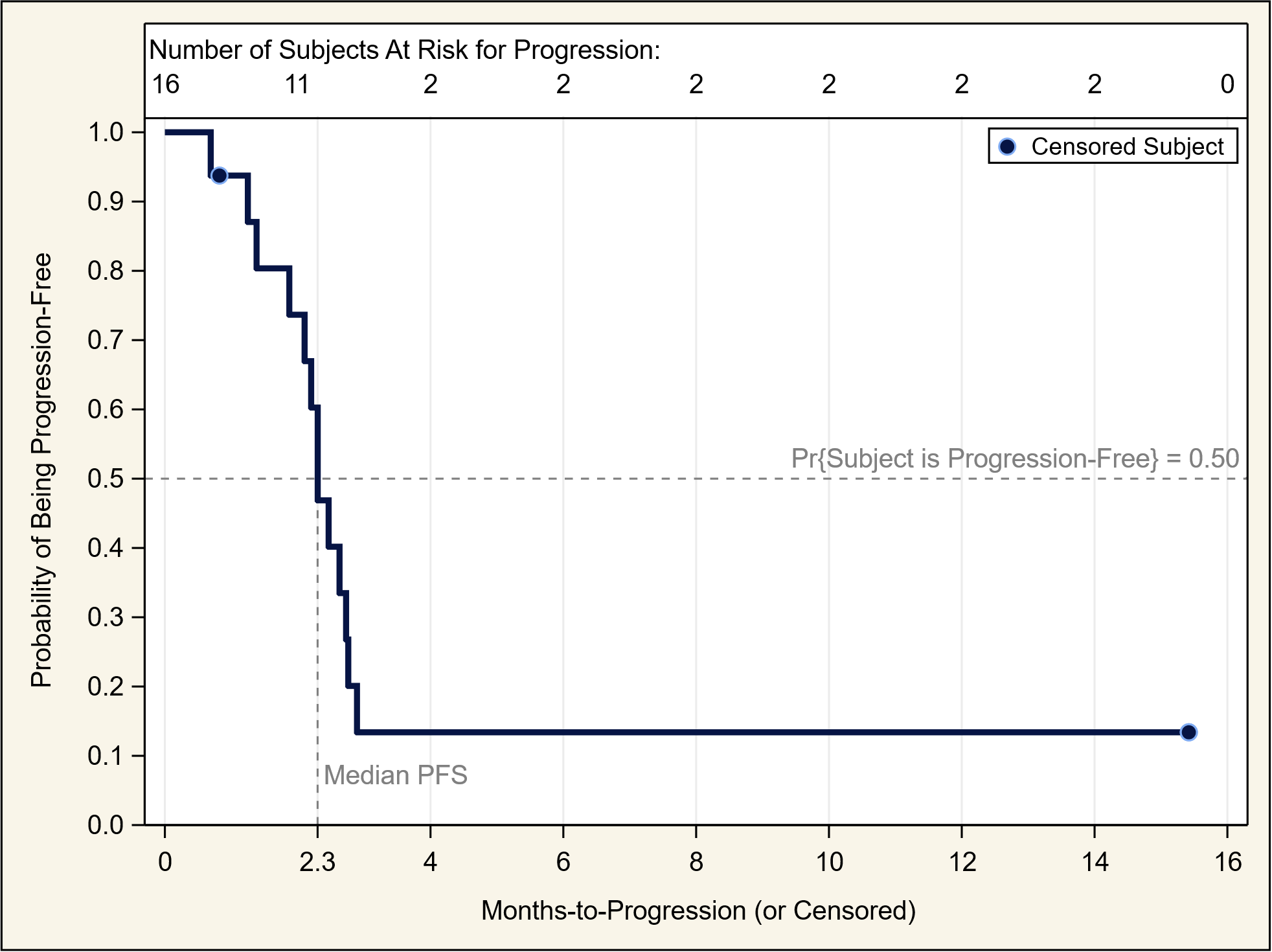


**S6**

**B**

**A**


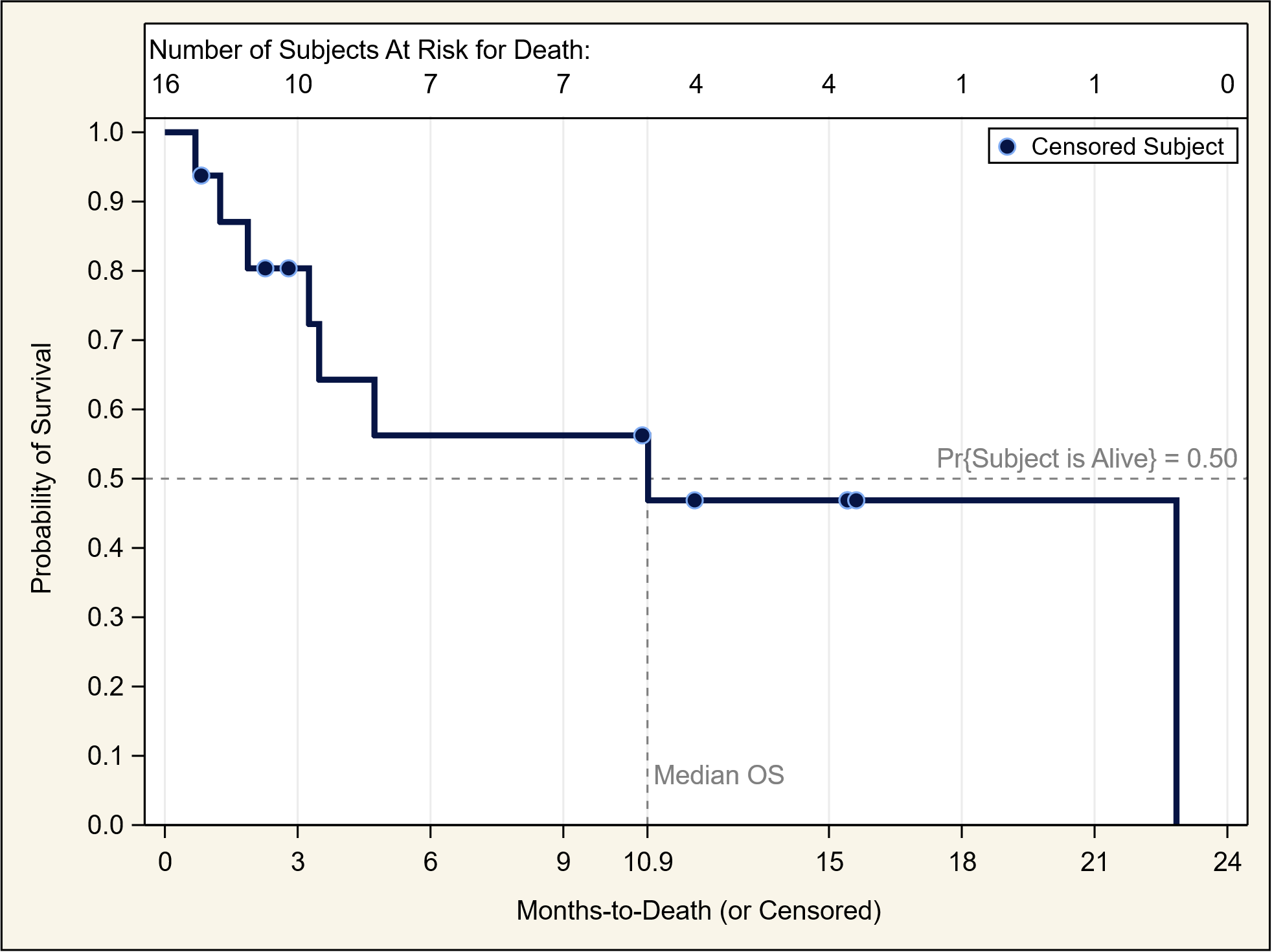


**Supplementary Figure 6: Survival outcomes of the trial.** (A) Progression-free survival and (B) overall survival Kaplan-Meier curves.
