# Supplementary material for: Phase II Clinical Trial and Preclinical Evaluation of a Novel CD47 Blockade Combination in Refractory Microsatellite-Stable Metastatic Colorectal Cancer
Source: Cancer Res Commun. 2025 Nov 20;5(11):2039–52. doi: 10.1158/2767-9764.CRC-25-0332 (PMC12631056; doi:10.1158/2767-9764.CRC-25-0332)
Supplement: Supplementary Figure S7 — Distance between tumor cells and CD4+ and CD8+ T cells in archival tumor tissue by immunohistochemistry. [file crc-25-0332_supplementary_figure_s7_suppsf7.docx]

**
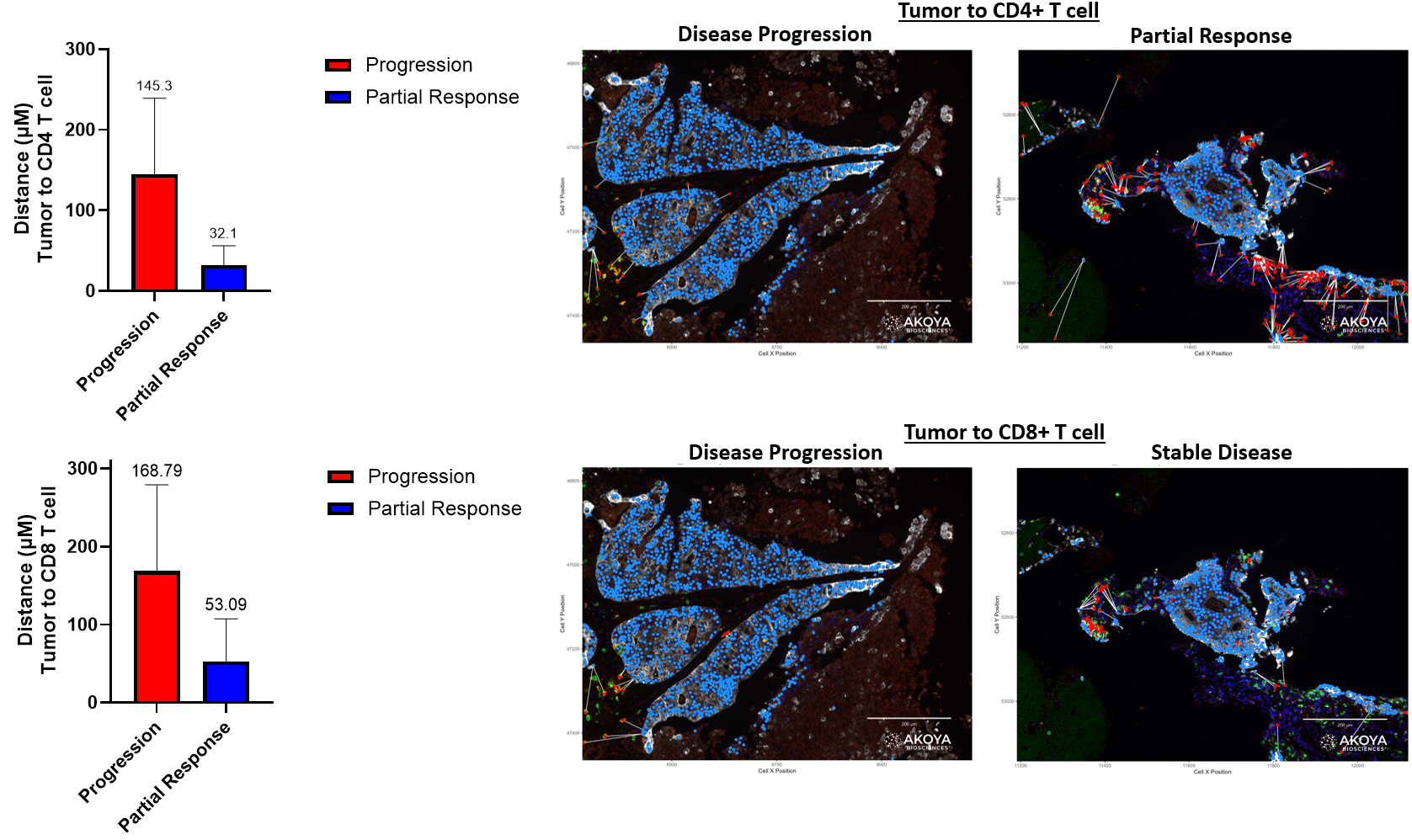
**

**S7**

**Supplementary Figure 7: Distance between tumor cells and CD4+ and CD8+ T cells in archival tumor tissue by immunohistochemistry.**
