# Supplementary material for: Phase II Clinical Trial and Preclinical Evaluation of a Novel CD47 Blockade Combination in Refractory Microsatellite-Stable Metastatic Colorectal Cancer
Source: Cancer Res Commun. 2025 Nov 20;5(11):2039–52. doi: 10.1158/2767-9764.CRC-25-0332 (PMC12631056; doi:10.1158/2767-9764.CRC-25-0332)
Supplement: Supplementary Figure S8 — Percent change in major peripheral blood cell populations by mass cytometry at Cycle 3 Day 1 versus baseline. [file crc-25-0332_supplementary_figure_s8_suppsf8.docx]

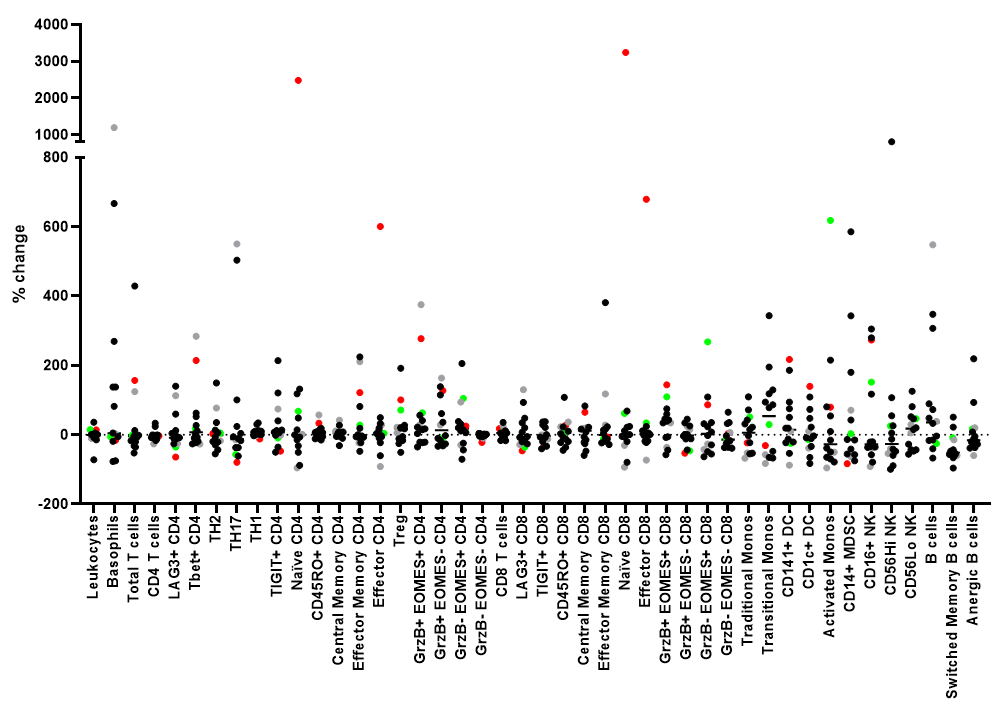


**S8**

●: Partial Response
●: Stable Disease
●: Progressive Disease
●: Not Response Evaluable

**Supplementary Figure 8: Percent change in major peripheral blood cell populations by mass cytometry at Cycle 3 Day 1 versus baseline.**
