# Supplementary material for: Phase II Clinical Trial and Preclinical Evaluation of a Novel CD47 Blockade Combination in Refractory Microsatellite-Stable Metastatic Colorectal Cancer
Source: Cancer Res Commun. 2025 Nov 20;5(11):2039–52. doi: 10.1158/2767-9764.CRC-25-0332 (PMC12631056; doi:10.1158/2767-9764.CRC-25-0332)
Supplement: Supplementary Figure S9 — Pie chart of peripheral blood mass cytometry. [file crc-25-0332_supplementary_figure_s9_suppsf9.docx]

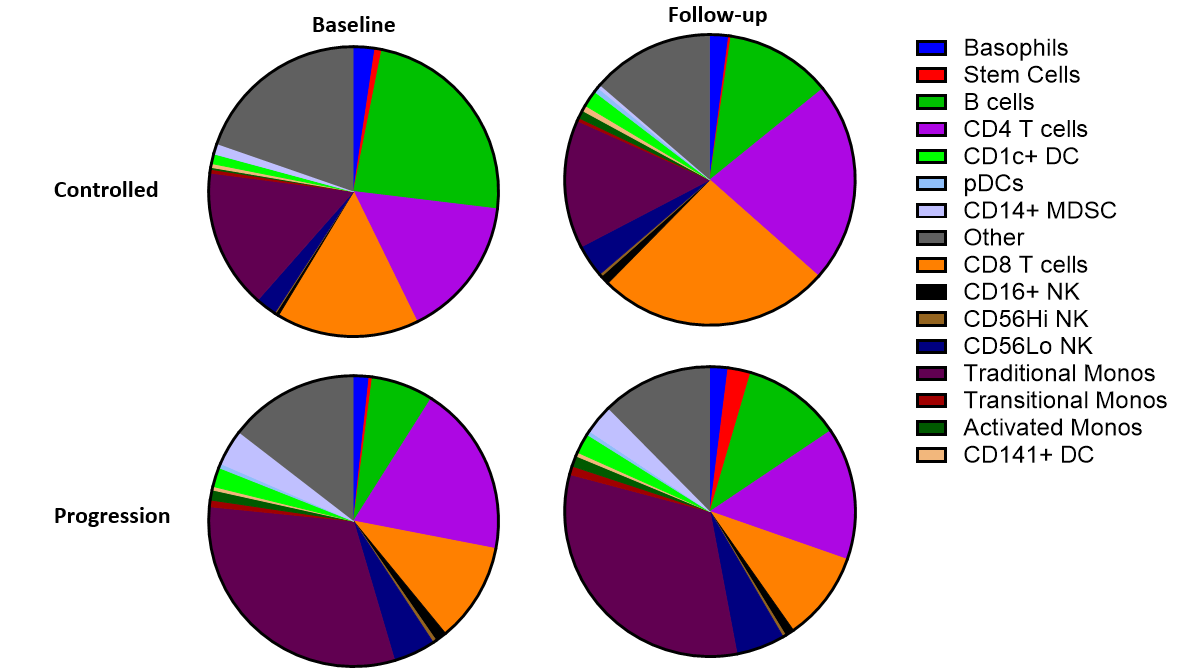


**S9**

**Supplementary Figure 9: Pie chart of peripheral blood mass cytometry.** In patients with controlled disease (stable disease or partial response) versus disease progression as best response, an increase in CD4+ and CD8+ T cells was observed at follow-up (Cycle 3 Day 1) compared to baseline.
