# Supplementary material for: Phase II Clinical Trial and Preclinical Evaluation of a Novel CD47 Blockade Combination in Refractory Microsatellite-Stable Metastatic Colorectal Cancer
Source: Cancer Res Commun. 2025 Nov 20;5(11):2039–52. doi: 10.1158/2767-9764.CRC-25-0332 (PMC12631056; doi:10.1158/2767-9764.CRC-25-0332)
Supplement: Supplementary Figure S10 — Serial peripheral blood mass cytometry by patient. [file crc-25-0332_supplementary_figure_s10_suppsf10.docx]

| 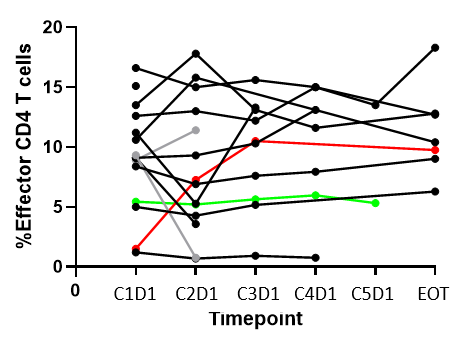 | 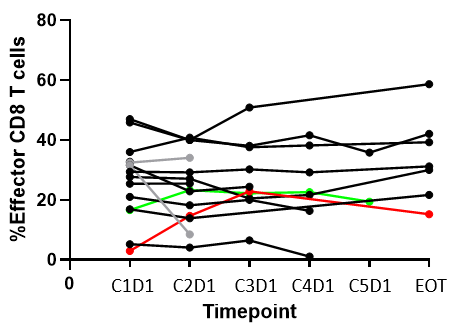 |
| --- | --- |
| 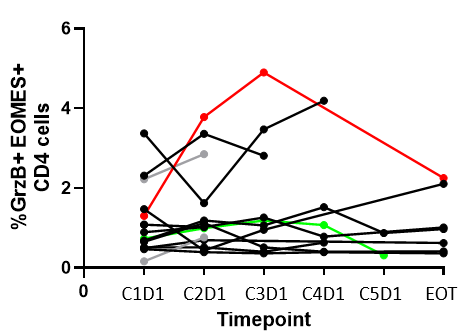 | 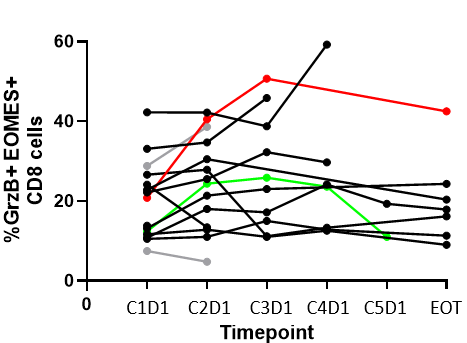 |
| 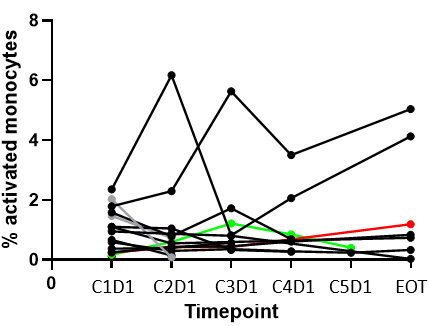 |   ●: Partial Response ●: Stable Disease ●: Progressive Disease ●: Not Response Evaluable |

**S10**

**Supplementary Figure 10: Serial peripheral blood mass cytometry by patient.**
