# Supplementary material for: Phase II Clinical Trial and Preclinical Evaluation of a Novel CD47 Blockade Combination in Refractory Microsatellite-Stable Metastatic Colorectal Cancer
Source: Cancer Res Commun. 2025 Nov 20;5(11):2039–52. doi: 10.1158/2767-9764.CRC-25-0332 (PMC12631056; doi:10.1158/2767-9764.CRC-25-0332)
Supplement: Protocol [file crc-25-0332_protocol_suppsp1.pdf]

A phase II study (with safety run-in) of evorpacept (ALX148) in combination with cetuximab and pembrolizumab in patients with refractory microsatellite stable metastatic colorectal cancer

**Protocol Number:**<br>**Protocol Version Date:**<br>**National Clinical Trial (NCT) Number**	AGICC-ALX148 21CRC01<br>December 28, 2022<br>NCT05167409
**Lead Principal Investigator:**	Robert Lentz, MD<br>Assistant Professor, Division of Medical Oncology<br>University of Colorado Cancer Center & School of Medicine<br>12801 E. 17th Avenue, Room L18-8124, Mail Stop 8117<br>Aurora, Colorado 80045
**Lead Site**	University of Colorado Cancer Center
**Study and Regulatory Sponsor:**	Academic Gastrointestinal Cancer Consortium (AGICC)
**Funded by:**	ALX Oncology Ltd, Merck & Co, Inc., & Eli Lilly USA, LLC

TABLE OF CONTENTS

STATEMENT OF COMPLIANCE 5
LIST OF ABBREVIATIONS 6
PARTICIPATING SITES 10
1 PROTOCOL SUMMARY 10
1.1 Synopsis 10
1.2 Study Schema 13
1.3 Schedule of Events 15
2 INTRODUCTION 20
2.1 Study Rationale 20
2.2 Background 20
2.2.1 PD-1/PD-L1 Checkpoint 21
2.2.2 Epidermal Growth Factor Receptor 22
2.2.3 SIRP Alpha/CD47 Checkpoint 23
2.2.4 Summary 25
2.3 Risk/Benefit Assessment 26
2.3.1 Known Potential Risks 26
2.3.2 Known Potential Benefits 28
2.3.3 Assessment of Potential Benefits 30
2.4 Known Pharmacokinetics 30
2.4.1 Pembrolizumab Pharmacokinetics 30
2.4.2 Cetuximab Pharmacokinetics 31
2.4.3 Evorpacept (ALX148) Pharmacokinetics 32
3 OBECTIVES AND ENDPOINTS 32
4 STUDY DESIGN 34
4.1 Overall Design 34
4.1.1 Study Overview 34
4.1.2 Criteria for Continuation of Treatment beyond RECIST v1.1-defined Progression 35
4.1.3 Stage 1: Safety Run-In 37
4.1.4 Stage 2: Dose Expansion 41
4.1.5 Dose-Limiting Toxicities 42
4.2 Scientific Rationale for Study Design 43
4.3 Justification for Dose 44
4.4 End of Study Definition 44
5 STUDY POPULATION 45
5.1 Inclusion Criteria 45
5.2 Exclusion Criteria 47
5.3 Lifestyle Considerations 50
5.4 Screen Failures 50
5.5 Strategies for Recruitment and Retention 50
6 STUDY INTERVENTION 51
6.1 Study Intervention(s) Administration 51
6.1.1 Study Intervention Description 51
6.1.2 Dosing and Administration 51
6.2 Study Drug Preparation/Handling/Storage/Accountability 54

6.2.1 Acquisition and Accountability 54
6.2.2 Formulation, Appearance, Packaging, and Labeling 54
6.2.3 Product Storage and Stability 55
6.2.4 Preparation 55
6.3 Measures to Minimize Bias: Randomization and Blinding 56
6.4 Study Intervention Compliance 56
6.5 Prohibited Therapy 56
6.6 Concomitant Therapy 58
6.6.1 Rescue Medicine 58
7 STUDY INTERVENTION DISCONTINUATION AND PARTICIPANT DISCONTINUATION/WITHDRAWAL 59
7.1 Discontinuation of Study Intervention (Stopping Rules) 59
7.2 Participant Discontinuation/Withdrawal from Study 60
7.3 Lost to Follow-Up 60
8 STUDY ASSESSMENTS AND PROCEDURES 61
8.1 Efficacy Assessments 61
8.2 Safety and Other Assessments 62
8.2.1 Screening 62
8.2.2 Treatment Period 66
8.2.3 Unscheduled Visit 68
8.2.4 Final study/Treatment Discontinuation Visit 69
8.2.5 After Treatment / Survival Follow-Up 69
8.2.6 Research Evaluations (and Specimen Preparation, Handling, Storage, and Shipment) 69
8.3 Adverse Events and Serious Adverse Events 73
8.3.1 Definition of Adverse Events (AE) 73
8.3.2 Definition of Serious Adverse Events (SAE) 73
8.3.3 Classification of an Adverse Event 74
8.3.4 Time Period and Frequency for Event Assessment and Follow-Up 76
8.3.5 Adverse Event Reporting 76
8.3.6 Serious Adverse Event Reporting 80
8.3.7 Reporting of Pregnancy 82
8.4 Unanticipated Problems 83
8.4.1 Definition of Unanticipated Problems (UAP) 83
8.4.2 Reporting of Unanticipated Problems 84
8.5 Events of Clinical Interest 85
9 STATISTICAL CONSIDERATIONS 85
9.1 Statistical Hypotheses 86
9.2 Sample Size Determination 87
9.3 Population for Analyses 87
9.4 Statistical Analyses 87
9.4.1 General Approach 87
9.4.2 Analysis of the Primary Efficacy Hypothesis & Stopping Criteria 88
9.4.3 Analysis of the Secondary Efficacy Hypotheses 88
9.4.4 Safety Analyses 89
9.4.5 Baseline Descriptive Statistics 89
9.4.6 Planned Interim Analyses 89

9.4.7 Tabulation of Individual Participant Data ... 90
9.4.8 Exploratory Analyses ... 90
10 SUPPORTING DOCUMENTATION AND OPERATIONAL CONSIDERATIONS ... 90
10.1 Regulatory, Ethical, and Study Oversight Considerations ... 90
10.1.1 Informed Consent Process ... 90
10.1.2 Study Discontinuation and Closure ... 92
10.1.3 Confidentiality and Privacy ... 92
10.1.4 Future Use of Stored Specimens or Data ... 94
10.1.5 Safety Oversight ... 94
10.1.6 Clinical Monitoring ... 95
10.1.7 Quality Assurance and Quality Control ... 95
10.1.8 Data Handling and Record Keeping ... 96
10.1.9 Protocol Deviations ... 97
10.1.10 Publication and Data Sharing Policy ... 97
10.1.11 Conflict of Interest Policy ... 98
11 REFERENCES ... 99
12 APPENDICES ... 105
12.1 Appendix 1: Guidelines for toxicity management, including dose modifications, dose interruptions, dose delays, & Discontinuation ... 105
12.1.1 General Guidance ... 105
12.1.2 Dose Levels ... 108
12.1.3 Management of Hematologic Toxicity ... 108
12.1.4 Management of Dermatologic Toxicity (Acneiform Rash) ... 111
12.1.5 Management of Dermatologic Toxicity (Other Than Acneiform Rash) ... 112
12.1.6 Management of Hepatotoxicity ... 113
12.1.7 Management of Pneumonitis ... 115
12.1.8 Management of Diarrhea & Colitis ... 116
12.1.9 Management of Infusion Reaction ... 120
12.1.10 Management of Endocrine Toxicity ... 121
12.1.11 Management of Renal Toxicity ... 122
12.1.12 Management of Hemophagocytic Lymphohistiocytosis ... 123
12.2 Appendix 2: Protocol criteria for measurement of study efficacy endpoints (RECIST v1.1 and iRECIST) ... 125
12.3 Appendix 3: ECOG Performance Status ... 133
12.4 Appendix 4: Efficacy of mCRC treatment options, including third line drugs, EGFR inhibitors, and a PD-1 inhibitor ... 134

STATEMENT OF COMPLIANCE

This is an investigator-initiated study. The lead principal investigator (PI), **Robert Lentz, MD**, and the Academic Gastrointestinal Cancer Consortium (AGICC), as the sponsor, are conducting the study. The legal/ethical obligations of a PI and those of a sponsor will be followed.

The trial will be carried out in accordance with Good Clinical Practice (GCP) as required by applicable United States (US) laws and applications, including but not limited to United States (US) Code of Federal Regulations (CFR) applicable to clinical studies (45 CFR Part 46, 21 CFR Part 50, 21 CFR Part 56, 21 CFR Part 312, and/or 21 CFR Part 812), as applicable.

The PI will assure that no changes to the protocol will take place without documented approval from the Institutional Review Board (IRB). All personnel involved in the conduct of this study have completed Human Subjects Protection Training.

I agree to ensure that all staff members involved in the conduct of this study are informed about their obligations in meeting the above commitments.

Lead Principal Investigator: ____

Print/Type Name

Signature: ______________________________ **Date:** ____________

Site Principal Investigator: ____

Print/Type Name

Signature: ________________________________ **Date:** ___________

LIST OF ABBREVIATIONS

ACRONYM	DESCRIPTION
ADA	Anti-drug antibody
ADCP	Antibody-dependent cellular phagocytosis
AE	Adverse event
AGICC	Academic Gastrointestinal Cancer Consortium
AIDS	Acquired immunodeficiency syndrome
ALP	Alkaline phosphatase
ALT	Alanine transaminase
ANC	Absolute neutrophil count
AST	Aspartate transaminase
AUC	Area under the concentration-time curve
BID	Bis in die or Twice a day
BP	Blood pressure
BUN	Blood urea nitrogen
C1D1	Cycle 1 Day 1
CBC	Complete blood count
CEA	Carcinoembryonic antigen
Cl	Clearance
Cmax	Maximum concentration
Cmin	Minimum concentration
CNS	Central nervous system
CR	Complete response
CRC	Colorectal cancer
CRF	Case report form
CT	Computed tomography
ctDNA	Circulating tumor DNA
Ctrough	Target trough concentration
Cyp	Cytochrome P 450
DC	Dendritic cells
DLT	Dose limiting toxicity
dMMR	Deficient mismatch repair
DNA	Deoxyribonucleic acid
DOR	Duration of response
EC	Ethics committee
ECG	Electrocardiogram
ECOG	Eastern cooperative oncology group
eCRF	Electronic case report form
EDC	Electronic data capture
EGFR	Epidermal growth factor receptor

EOS	End of study
FDA	Food and Drug Administration
FFPE	Formalin-fixed, paraffin-embedded
FIP	First-in-patient
HBcAb	Hepatitis B core antibody
HbsAg	Hepatitis B surface antigen
HBV	Hepatitis B virus
HCV	Hepatitis C virus
HER2	Human epidermal growth factor receptor 2
HIMSR	Human Immune Monitoring Shared Resource
HIPAA	Health insurance portability and Accountability Act
HIV	Human immunodeficiency virus
HLH	Hemophagocytic lymphohistiocytosis
HNSCC	Head/neck squamous cell carcinoma
HR	Hazard Ratio
IARC	International Agency for Research on Cancer
ICH	International Conference on Harmonization
IHC	Immunohistochemistry
IIT	Investigator-Initiated Trial
IMP	Investigational medicinal product
IND	Investigational new drug
INR	International normalized ratio
IRB	Institutional Review Board
iRECIST	Immune Response Evaluation Criteria in Solid Tumors
ITT	Intention to treat population
IUD	Intrauterine device
IV	Intravenous
LDH	Lactate dehydrogenase
LFT	Liver function test
mAb	Monoclonal antibody
mCRC	Metastatic colorectal cancer
mg	Milligram
ml	Milliliter
MMR	Mismatch repair
MP	Monitoring plan
MRI	Magnetic resonance imaging
mRNA	Messenger RNA
Msec	Millisecond
MSI	Microsatellite
MSI-H	Microsatellite unstable
MTD	Maximum-tolerated dose

NCI CTCAE	National Cancer Institute Common Terminology Criteria for Adverse Events
NGS	Next generation Sequencing
NK	Natural killer cell
NOEL	No observed effect level
NSCLC	Non-small cell lung cancer
OHRP	Office for Human Research Protections
ORR	Overall response rate
OS	Overall survival
OTC	Over the counter
PCR	Polymerase chain reaction
PD	Progressive disease
PD	Pharmacodynamic
PD-1	Programmed death-1
PD-L1	Programmed death ligand-1
PE	Physical exam
PET	Positron emission tomography
PFS	Progression free survival
PK	Pharmacokinetic
PO	Per Os
PR	Pulse rate
PR	Partial response
PS	Performance status
PT	Prothrombin time
PTT	Partial thromboplastin time
Q3w	Every 3 weeks
QD	Once per day
QoW	Every other week
QW	Weekly
RBC	Red blood cell
RECIST	Response Evaluation Criteria in Solid Tumors
RNA	Ribonucleic acid
SAE	Serious adverse event
SD	Stable disease
SD	Stable disease
SGOT	Serum glutamic oxaloacetic transaminase
SGPT	Serum glutamic pyruvic transaminase
SIRPα	Signal regulatory protein alpha
SOP	Standard operating procedures
T ½	Terminal elimination half-life
TAM	Tumor-associated macrophages
TIL	Tumor-infiltrating lymphocyte

TK	Toxicokinetic
TMB	Tumor mutational burden
TME	Tumor microenvironment
TNF-α	Tumor necrosis factor-α
TSH	Thyroid-stimulating hormone
UA	Urinalysis
UAP	Unanticipated Problems
ULN	Upper limit of normal
ULN	Upper limit of normal
VEGF	Vascular endothelial growth factor
Vss	Volume of distribution at steady-state
WBC	White blood cell

PARTICIPATING SITES

The University of Colorado and members of the Academic GI Gastrointestinal Cancer Consortium (AGICC) are the participating sites in this study (all within the United States). A complete and current listing of investigators, research personnel, research facilities and other study centers (if applicable) participating in this study will be maintained throughout the duration of this study on applicable study required forms such as an *FDA Form 1572*, the *COMIRB Research Personnel Form*, and/or a *UCCC Protocol Contact List*, incorporated herein by reference.

1 PROTOCOL SUMMARY

1.1 SYNOPSIS

Protocol Title: *A phase II study (with safety run-in) of evorpacept (ALX148) in combination with cetuximab and pembrolizumab in patients with refractory microsatellite stable metastatic colorectal cancer*

Objectives:

- ***Primary Objectives:***
  1. *To determine the recommended dose (RD) of evorpacept (ALX148) in combination with cetuximab and pembrolizumab*
  2. *To determine the objective response rate (ORR), defined as partial response or complete response, with evorpacept (ALX148), cetuximab, and pembrolizumab using RECIST v1.1 in patients with microsatellite stable (MSS) metastatic colorectal cancer (mCRC) who have progressed on at least two lines of standard therapy*

- ***Secondary Objectives:***
  1. *To determine the disease-control rate (DCR), defined as stable disease, partial response, or complete response with evorpacept (ALX148), cetuximab, and pembrolizumab using RECIST v1.1*
  2. *To determine the duration of response (DOR) with evorpacept (ALX148), cetuximab, and pembrolizumab, defined as the time from response (partial or complete) to progression using RECIST v1.1 or death from any cause*
  3. *To determine the progression-free survival (PFS) with evorpacept (ALX148), cetuximab, and pembrolizumab, defined as the time from enrollment to the first observation of progression using RECIST v1.1 or death from any cause*

4. *To determine the overall survival (OS) with evorpacept (ALX148), cetuximab, and pembrolizumab, defined as the time from enrollment to death from any cause*
5. *To determine the first cycle dose-limiting toxicities (DLT) of evorpacept (ALX148), cetuximab, and pembrolizumab in Stage 1*
6. *To evaluate the safety and tolerability of evorpacept (ALX148), cetuximab, and pembrolizumab, defined and graded according to the NCI CTCAE v5.0*

- ***Exploratory Objectives:***
  1. *To compare the objective response rate (ORR), disease-control rate (DCR), duration of response (DOR), and progression-free survival (PFS) determined according to RECIST v1.1 to that identified by iRECIST*
  2. *To identify immune modulation (myeloid and lymphoid) in the peripheral blood and tissue biopsies pre- and post-treatment with evorpacept (ALX148), cetuximab, and pembrolizumab*
  3. *To correlate immune modulation in the peripheral blood and tissue biopsies pre- and post-treatment with evorpacept (ALX148), cetuximab, and pembrolizumab with response*
  4. *To evaluate the relationship between PD-L1, EGFR, and CD47 tumor expression and efficacy of evorpacept (ALX148), cetuximab, and pembrolizumab*
  5. *To evaluate the relationship between tumor mutational burden (TMB) and efficacy of evorpacept (ALX148), cetuximab, and pembrolizumab*

Endpoints:

- ***Primary Endpoints:***
  1. *Recommended dose (RD) of evorpacept (ALX148) in combination with cetuximab and pembrolizumab*
  2. *Objective response rate (ORR per RECIST v1.1)*

- ***Secondary Endpoints:***
  1. *Disease control rate (DCR, per RECIST v1.1)*
  2. *Duration of response (DOR, per RECIST v1.1)*
  3. *Progression-free survival (PFS, per RECIST v1.1)*
  4. *Overall survival (OS)*
  5. *First-cycle dose-limiting toxicities (DLTs) in stage 1*
  6. *Safety and tolerability defined and graded according to the NCI CTCAE v5.0*

- ***Exploratory Endpoints:***

1. *Objective response rate (ORR, per iRECIST), disease-control rate (DCR, per iRECIST), duration of response (DOR), and progression-free survival (PFS, per iRECIST)*
2. *Change in pre- and post-treatment levels and activation of immune cells in the peripheral blood and tumor microenvironment*
3. *Change in pre- and post-treatment mRNA expression in a panel of immune-related genes in tumor tissues*
4. *Objective response rate (by RECIST v1.1 and iRECIST) according to change in pre- and post-treatment levels and activation of immune cells in the peripheral blood and tumor microenvironment*
5. *Objective response rate (by RECIST v1.1 and iRECIST) according to pre-treatment PD-L1, EGFR, and CD47 tumor expression by immunohistochemistry*
6. *Objective response rate (by RECIST v1.1 and iRECIST) according to tumor mutational burden (TMB)*

Population:

- ***Gender*** *Male and Female*
- ***Age*** *≥ 18 years*
- ***Demographic group*** *refractory microsatellite stable metastatic colorectal cancer*
- ***General health status*** *ECOG performance status 0-1*
- ***Sample size***
  - *Maximum number of participants that can be enrolled is 80 (allowing for screen failures)*
  - *Minimum number of participants to be enrolled is 48 (number of participants needed to answer scientific question/aims)*

Phase: *II (with safety run-in)*

Participating Sites: *University of Colorado*
Members of the Academic GI Cancer Consortium (AGICC)

Description of Study Intervention: *Evorpacept (ALX148)+ cetuximab + pembrolizumab*

Study Duration: *Approximately 24 months (enrollment/treatment) and 48 months (including survival follow-up)*

Participant Duration: *Approximately 12 months (enrollment/treatment) and 36 months (including survival follow-up)*

1.2 STUDY SCHEMA

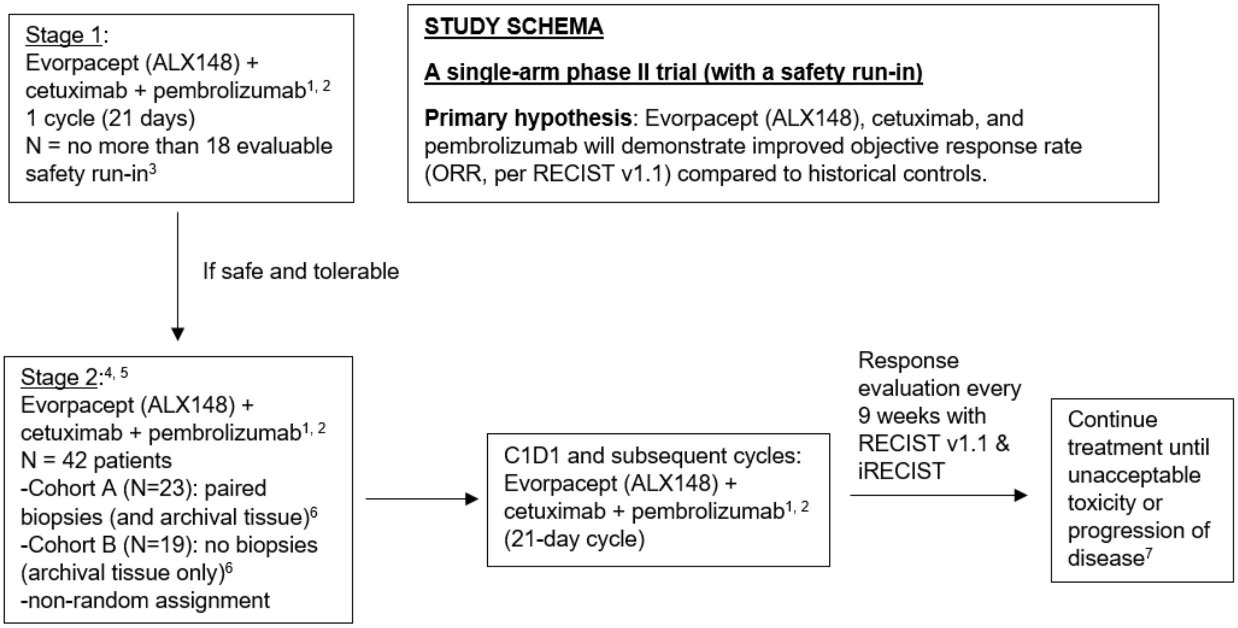

1. Dose Levels:
   - Evorpacept (ALX148) dose level (DL) 1: 15 mg/kg weekly; DL -1: 10 mg/kg weekly; DL -2: 5 mg/kg weekly
   - Cetuximab DL 1: 250 mg/m^2 weekly; DL -1: 200 mg/m^2 weekly; DL -2: 150 mg/m^2 weekly
   - Pembrolizumab DL 1: 200 mg every 3 weeks (no additional dose levels)
2. The desired mechanism of action of cetuximab is to augment phagocytosis via antibody-dependent cellular phagocytosis (ADCP) rather than to modulate the EGFR signaling pathway.
3. No more than 18 evaluable patients will be accrued in Stage 1. A single patient will be treated with evorpacept (ALX148), cetuximab, and pembrolizumab, followed by a 48-hour safety observation period prior to treating the next patient. A 48-hour safety observation period will be required between the start of treatment for patients in Stage 1. Additional patients may be enrolled at lower dose levels if at least 33% of patients experience a first-cycle dose limiting toxicity.
4. If a significant portion of patients in Stage 2 experience AEs meeting dose-limiting toxicity (DLT) criteria as defined in Section 4.1.5, then dose reduction(s) of evorpacept (ALX148) and/or cetuximab for subsequent patients can be considered in discussion with the Investigators and Medical Monitor.
5. A Simon-like 2-stage design will be used. If there are no responses (partial response or complete response) among the first 24 evaluable patients (including patients in both Stage 1 and Stage 2), study enrollment will be stopped for futility. Otherwise, full study enrollment will continue as planned.
6. Patients in Cohort A will provide a fresh pretreatment biopsy sample (before C1D1 but after screening) and an on-treatment biopsy sample at C3D1 (±3 days). While the acceptable window for the C3D1 biopsy is ±3 days, it is preferred for the biopsy to occur following all treatments on C3D1. The date and time of the biopsies will be documented. In Cohort A, archival tissue may not be used in place of a fresh pre-treatment biopsy. Patients in Cohort B will not undergo any study-mandated biopsies. When available, archival tissue will also be obtained for analysis for patients in both Cohort A and Cohort B. For a subject in Cohort A, if the Investigator judges the risk for the pre- or on-treatment biopsy to be unacceptable in a research setting eg, based on complications during a previous procedure, use of blood thinning medications, or inaccessible tumor, or the patient refuses, a subject should not undergo the pre- or on-treatment biopsy and the patient will be allowed to enroll in or remain on the study. If a patient initially assigned to Cohort A is unable to undergo both the pretreatment and on-treatment biopsies due to medical reasons or patient refusal, an additional patient may be enrolled in Cohort A (to achieve N=23 patients who underwent pretreatment and on-treatment biopsies). Other than biopsies, there are no differences between Cohort A and Cohort B (both receive the same treatment and study events). Details for handling of these samples including processing, storage, and shipment will be provided in the Laboratory Manual.

7. The maximum duration of pembrolizumab is 35 cycles. There is no maximum duration of evorpacept (ALX148) or cetuximab.

1.3 SCHEDULE OF EVENTS

Table 1. Schedule of events (21 day cycle)

		CYCLE 1 (Days -21)			CYCLE 2 (Days 1-21)			CYCLE 3 & LATER CYCLES (Days 1-21)			Final Study / Treatment Discontinuation Visit[24]	Survival follow-up
Protocol activity	Screening (≤28 days)[1]	D1	D8 (±1)	D15 (±1)	D1 (±1)	D8 (±1)	D15 (±1)	D1 (±1)	D8 (±1)	D15 (±1)	(28-35 days after treatment discontinuation)	
Informed consent[2]	X											
Tumor history[3]	X											
Demographic/medical history[4]	X											
Physical examination[5] (C=complete, B=brief)	X^{C}	X^{B}	X^{B}	X^{B}	X^{B}	X^{B}		X^{B}			X^{C}	
Baseline signs and symptoms[6]	X	X										
Weight[7]	X	X			X			X			X	
Vital signs (BP/HR/T)[8]	X	X	X	X	X	X		X			X	
ECOG performance status[9]	X	X			X			X			X	
12 lead ECG[10]	X	X	X					X			X	
Hematology[11]	X	X	X	X	X	X		X			X	
Blood chemistry[12]	X	X	X	X	X	X		X			X	
Coagulation[13]	X	X			X						X	
Urinalysis[14]	X	X			X						X	
Pregnancy test[15]	X	X									X	
CEA		X			X			X			X	
TSH, free T4, and cortisol	X				X			X			X	
HBV, HCV, and HIV[16]	X											
Registration[17]	X											
Evorpacept (ALX148) dosing (weekly)[18]		X	X	X	X	X	X	X	X	X		
Cetuximab dosing (weekly)		X	X	X	X	X	X	X	X	X		
Pembrolizumab dosing (every 3 weeks)[19]		X			X			X				
Tumor assessment[20]	X									X	X	
Adverse events[21]	X	X	X	X	X	X	X	X	X	X	X	
Concomitant treatments[22]	X	X	X	X	X	X	X	X	X	X	X	
Tumor tissue samples[23]	X							X				
Survival Follow-up[25]												X
Blood sample for PK[26]		X			X			X			X	
Blood sample for PD/biomarker analysis[27]		X			X			X			X	
Blood sample for ADA[28]		X			X			X			X	

ADA=anti-drug antibodies; CEA=carcinoembryonic antigen; ECOG=Eastern Cooperative Oncology Group; HBV=hepatitis B virus; HCV=hepatitis C virus; HIV=human immunodeficiency virus; PD=pharmacodynamic; PK=pharmacokinetic; TSH=thyroid stimulating hormone

Footnotes
1. Screening: to be obtained within 28 days prior to study entry (ie: prior to C1D1).

2. Informed Consent: must be obtained prior to undergoing any study specific procedures.

3. Tumor History: History of CRC should include stage, date of first diagnosis, site of primary disease, previous biopsies particularly date and site of biopsy, location of metastases, previous treatments administered including all surgeries and radiation therapy, previous clinical trials especially details of any previous use of immunotherapies, ECOG performance status, MMR/MSI status, tumor mutational burden (TMB), and mutational testing particularly KRAS, NRAS, and BRAF.

4. Demographic/Medical History: Includes age, sex, self-reported ethnicity, detailed description of history of non-CRC cancer, past medical and surgical history, list of medications including prescription, OTC, herbal, homeopathic, nutritional drugs, performance status, social history including alcohol, tobacco and recreational drug use, family history especially of malignancy and reproductive history.

5. Physical Exam: A complete (C) physical exam (including height at screening only) will be conducted at screening and at the EOT visit. A brief (B) physical exam will be conducted at the other time points.

6. Baseline Signs & Symptoms: patients will be asked about any signs and symptoms experienced within the 14 days prior to study entry. Baseline signs and symptoms will be recorded on the electronic case report form (eCRF).

7. Weight: Patient's body weight will be measured at the beginning of each cycle and drug doses for that cycle will be based on the patient's body weight.

8. Vital signs: blood pressure (BP), heart rate (HR), and temperature to be recorded in the sitting position.

9. Performance status: use Eastern Cooperative Oncology Group (ECOG) performance status criteria (Appendix 3)

10. 12-lead ECG: A single ECG will be collected on at least a 10 second strip at screening and after infusion of evorpacept (ALX148) on C1D1, C1D8, and C3D1. No further study-mandated ECGs are needed after C3D1, except an ECG will be done at the Final study / treatment discontinuation visit. If the QTcF is prolonged (value of >500 msec), the ECG should be re-evaluated by a qualified person at the institution for confirmation. Additional ECGs should be performed as clinically indicated.

11. Hematology: Complete blood count (CBC) to include hemoglobin, platelets, WBC, absolute neutrophils, lymphocytes, monocytes, eosinophils, and basophils. No need to repeat on Cycle 1 Day 1 (C1D1) if screening assessment performed within 72 hours prior to that date. Additional testing as clinically needed. Blood type and a RBC antibody screen (in addition, baseline genotyping or extended phenotypoing of minor red cell antigens is recommended, if testing is available) also known as a 'Type and Screen' are to be collected at screening. See Section 8.2.1 for additional information regarding transfusion laboratory testing.

12. Blood Chemistry: Should include sodium, potassium, chloride, bicarbonate or carbon dioxide, BUN (or urea), creatinine, non-fasting glucose, calcium, **magnesium**, **phosphorus**, albumin, total protein, AST/SGOT, ALT/SGPT, alkaline phosphatase, total bilirubin, **lipase**, and **amylase**. No need to repeat

on Cycle 1 Day 1 (C1D1) if screening assessment performed within 72 hours prior to that date. Additional testing as clinically needed.

13. Coagulation: Should include International Normalized Ratio (INR) and Partial Thromboplastin Time (PTT). No need to repeat on Cycle 1 Day 1 (C1D1) if screening assessment performed within 72 hours prior to that date.

14. Urinalysis: Dipstick is acceptable. Microscopic analyses if dipstick abnormal. No need to repeat on C1D1 if screening assessment performed within 72 hours prior to that date. If ≥2+ protein on urine dipstick, then collect spot urine sample to calculate urine protein to creatinine ratio (UPCR).

15. Serum Pregnancy Test: For female patients of childbearing potential, a serum pregnancy test, with sensitivity of at least 25 mIU/mL, and assayed in a certified laboratory, will be performed on two occasions prior to starting study therapy - once at screening and once at the C1D1 visit, immediately before investigational product administration. The test must be repeated at the Final Study/Treatment Discontinuation Visit. Additional pregnancy tests may also be undertaken if requested by institutional review board/ethics committee (IRb/ECs) or if required by local regulations.

16. HBV, HCV and HIV: HBV serology: Hepatitis B surface antigen, hepatitis B surface antibody, and total hepatitis B core antibody. HBV DNA should be obtained prior to enrollment if patient has a negative serology for HbsAg and a positive serology for anti-HBcAb (see Section 5.2). HCV serology: HCV antibody (anti-HCV). HCV RNA should be obtained prior to enrollment if patient tests positive for anti-HCV. HIV: antigen/antibody combination test.

17. Registration: Site numbers are entered into the Electronic Data Capture (EDC) system when the EDC is created. Patient numbers (registration) are assigned by AGICC when the patient signs the Informed Consent Form (ICF) and the patient is entered into the EDC. Screening failures are captured in the EDC. Enrollment occurs when the Medical Monitor approves the screening documents with his/her signature.

18. Patients will be observed in the clinic for at least 2 hours after infusion of evorpacept (ALX148) on C1D1 and as clinically indicated, thereafter.

19. Vital signs at the first pembrolizumab infusion will be collected within 60 min prior to the infusion, every 15 (± 10) min during the pembrolizumab infusion and 30 (± 10) min after the infusion. For subsequent infusions, vital signs will be collected within 60 min prior to the infusion and should be collected during the infusion if clinically indicated or if symptoms occurred in the prior infusion(s).

20. Tumor Assessments for solid tumors: Baseline tumor assessments should be performed ≤ 28 days before Cycle 1 Day 1 and assessed according to RECIST v1.1 and iRECIST (see Appendix 2). The same procedure used to assess disease sites at baseline should be used throughout the study (e.g., the same contrast protocol for CT scans or MRI scans). CT or MRI scans should include chest, abdomen, and pelvic scans; and other areas as clinically indicated. At the investigator's discretion, imaging may be repeated at any time if progressive disease is suspected. Evaluation of tumor response conforming to RECIST v1.1 and iRECIST must be documented every 9 weeks ± 5 days (no matter where the patient is in the treatment cycle). Patients in both the safety run-in and dose expansion stages will continue to receive study therapy until disease progression according to RECIST v1.1 (Appendix 2), unacceptable toxicity, death, patient or physician decision to withdraw, or pregnancy, whichever occurs first. In all cohorts, at the time of RECIST v1.1-defined progression (i.e. iUPD), treatment is permitted to continue until the next assessment (at least 4 weeks but no longer than 8 weeks later) if the following criteria are

met

- Patient is clinically stable (An assignment of clinical stability requires that no worsening of performance status has occurred, that no clinically relevant increases in disease-related symptoms such as pain or dyspnea occur that are thought to be associated with disease progression (these symptoms are generally understood to mean a requirement for increased palliative intervention), and that no requirement for intensified management of disease-related symptoms exists, including increased analgesia, radiotherapy, or other palliative care.)
- Absence of tumor progression at critical anatomical sites (e.g., leptomeningeal disease) that cannot be managed by protocol-allowed medical interventions
- Patient and investigator agree to continue treatment
- Patient signs the Treatment Beyond Progression Informed Consent Form
- If the subsequent scan continues to show progression, all therapy will be discontinued and the patient will be taken off study.

If iUPD is noted, a follow-up scan should be obtained at least 4 weeks but no longer than 8 weeks later. Schedule of tumor assessments are independent of any changes to the study treatment administration schedule (e.g., dose delay) and may occur mid-cycle depending on length of cycle. If a tumor assessment must be performed early or late, subsequent assessments should be conducted according to the original schedule based on the date of first study drug administration (Cycle 1, Day 1). Confirmation of response (PR or complete response [CR]) will be done no earlier than 28 days from study entry. In the case of SD, measurements must have met the SD criteria at least once after study entry at a minimum interval not less than 6 weeks. Patients who discontinue study treatment for any reason other than disease progression will continue to undergo tumor response evaluations (approximately every 9 weeks) until progressive disease. Rising tumor markers (e.g., CEA) in the absence of radiological evidence of progression is not considered progressive disease.

21. Adverse Event (AE) Assessments: Adverse events should be documented and recorded at each visit using the National Cancer Institute Common Terminology Criteria for Adverse Events (NCI CTCAE) version 5.0. Patients must be followed for non-serious AEs from C1D1 up to 28 days after the last study treatment administration or until all drug-related toxicities have resolved, whichever is later; or earlier than 28 days should the patient commence another anticancer therapy in the meantime. For serious adverse events (SAEs), the active reporting period to AGICC or its designated representative begins from the time that the patient provides informed consent, which is obtained prior to the patient's participation in the study, ie, prior to undergoing any study-related procedure and/or receiving investigational product, through and including 28 calendar days after the last administration of the investigational product. SAEs experienced by a patient after the active reporting period has ended should be reported to the AGICC if the Investigator becomes aware of them, unless the SAE is attributed by the Investigator to complications of either the underlying malignancy or any subsequent anti-cancer therapy or to the patient's participation in a subsequent clinical study; at a minimum, all SAEs that the Investigator believes have at least a reasonable possibility of being related to investigational product are to be reported to the AGICC. To collect late immune related adverse event information, all patients will be contacted by phone 90 days after EOT. AEs will be collected continuously if there are changes between study visits.

22. Concomitant Treatments: all concomitant medications and Non-Drug Supportive Interventions should be recorded in the eCRF within 28 days prior to study entry (i.e., prior to C1D1). All concomitant medications taken during study participation will continue to be recorded continuously on the eCRF starting at the screening visit and ending 30 days after treatment discontinuation (See Section 6.6).

23. Tumor Tissue Samples: Patients in Cohort A will provide a fresh pretreatment biopsy sample and an

on-treatment biopsy sample at C3D1 (±3 days). Prior to the pre-treatment biopsy, patients must sign the Informed Consent Form and all other described screening evaluations (Section 8.2.1 – "Screening Assessments to Determine Eligibility") must be completed and reviewed by the treating clinician to confirm that all eligibility criteria are met. While the acceptable window for the C3D1 biopsy is ±3 days, it is preferred for the biopsy to occur following all treatments on C3D1. The date and time of the biopsies will be documented. In Cohort A, archival tissue may not be used in place of a fresh pre-treatment biopsy. Patients in Cohort B will not undergo any study-related biopsies. When available, archival tissue will also be obtained for analysis for patients in both Cohort A and Cohort B. In rare circumstances, if there are no available spots in Cohort B and if a patient is motivated to participate and the Investigator judges that the risk associated with the biopsy is not appropriate for a research setting or the patient refuses biopsy, after consultation with the AGICC's medical monitor the patient may be allowed on study without a fresh biopsy. Similarly, for a subject in Cohort A, if the Investigator judges the risk for the pre- or on-treatment biopsy to be unacceptable in a research setting eg, based on complications during a previous procedure, use of blood thinning medications, or inaccessible tumor, or the patient refuses, a subject should not undergo the pre- or on-treatment biopsy and the patient will be allowed to enroll in or remain on the study. If a patient initially assigned to Cohort A is unable to undergo both the pretreatment and on-treatment biopsies due to medical reasons or patient refusal, an additional patient may be enrolled in Cohort A (to achieve N=23 patients who underwent pretreatment and on-treatment biopsies). Other than biopsies, there are no differences between Cohort A and Cohort B (both receive the same treatment and study events). Details for handling of these samples including processing, storage, and shipment will be provided in the Laboratory Manual.

24. Final study / treatment discontinuation visit: At least 28 days and no more than 35 days after discontinuation of treatment, patients will return to undergo the listed activities (unless already completed in the last week, or last 6 weeks for response assessment). Patients continuing to experience toxicity at this point following discontinuation of treatment will continue to be followed at least every 4 weeks until resolution or determination, in the clinical judgment of the Investigator, that no further improvement is expected. If the patient has any concern and contacts the clinic, the patient will be seen in the clinic within 5 calendar days of the patient's contact with the clinic (assessments will be the same as the assessments performed at the Final Study/Treatment Discontinuation Visit).

25. Overall survival information will be collected via telephone calls and/or clinic visits every 3 months ±14 days (first call and/or clinic visit to occur 3 months following the Final Study/Treatment Discontinuation Visit) until death, withdrawal of consent, the patient is lost to follow-up, study termination by AGICC, or a maximum of 2 years from the Final Study/Treatment Discontinuation Visit.

26. Blood will be drawn for pharmacokinetic (PK) analysis prior to the start of evorpacept (ALX148) infusion and immediately following completion of evorpacept (ALX148) infusion (within 6 minutes after end of infusion) on Day 1 of Cycles 1-5. Starting with Cycle 6, the frequency of the pre- and post-evorpacept (ALX148) infusion PK draws will be decreased to every 3 cycles (i.e. C6D1, C9D1, C12D1, etc). A final blood draw for PK analysis will be performed at the Final Study Visit / Treatment Discontinuation Visit. In addition to samples collected at the scheduled times, an additional blood sample may be requested from patients experiencing unexpected and/or serious AE's with the date and time documented in the eCRF. Additional details can be found in the Laboratory Manual and Section 8.2.6.

27. Blood will be drawn for pharmacodynamic (PD)/biomarker analyses prior to the start of evorpacept (ALX148) infusion at Day 1 of each cycle and at the Final Study Visit/Treatment Discontinuation Visit. Additional details can be found in the Laboratory Manual and Section 8.2.6.

28. Blood will be drawn for anti-drug antibody (ADA) analysis prior to the start of evorpacept (ALX148) infusion Day 1 of Cycles 1-5. Starting with Cycle 6, the frequency of the pre-evorpacept (ALX148) infusion ADA draws will be decreased to every 3 cycles (i.e. C6D1, C9D1, C12D1, etc). A final blood draw for ADA analysis will be performed at the Final Study Visit / Treatment Discontinuation Visit. In addition to samples collected at the scheduled times, an additional blood sample may be requested from patients experiencing unexpected and/or serious AE's with the date and time documented in the eCRF. Additional details can be found in the Laboratory Manual and Section 8.2.6.

2 INTRODUCTION

2.1 STUDY RATIONALE

Colorectal cancer is common, deadly, and there is an unmet need for treatment options in patients with refractory metastatic disease. The current mainstay of treatment in metastatic colorectal cancer (mCRC) is cytotoxic chemotherapy with or without inhibitors of epidermal growth factor receptor (EGFR) and vascular endothelial growth factor (VEGF). However, many patients do not respond, duration of response is often limited, and treatment-related adverse events impair quality of life. Further, single-agent anti-PD-1 therapy is not effective for microsatellite stable (MSS) mCRC.

This study proposes combination therapy with three agents designed to synergistically generate an anti-tumor immune response by activation of both the innate and adaptive immune systems. Clinical and non-clinical data suggests that inhibition of the SIRPα/CD47 checkpoint (evorpacept [ALX148]), inhibition of the PD-1/PD-L1 checkpoint (pembrolizumab), and use of an anti-EGFR antibody (cetuximab) should result in additive anti-tumor activity.

2.2 BACKGROUND

Colorectal cancer (CRC) is a common and deadly condition. Worldwide, there are approximately 1.8 million new cases per year and 881,000 deaths per year, making it the third most prevalent and second most lethal cancer.[1] In the Unites states alone there are approximately 150,000 new cases per year and 53,000 deaths per year.[2] It is estimated that by 2035 there may be 2.5 million new cases of CRC per year worldwide.[3] While improved screening and treatments have improved prognosis, the 5-year survival probability of metastatic CRC (mCRC) is only about 12%.[2] There are many systemic treatment options for CRC. In patients with microsatellite stable (MSS) mCRC, first- and second-line treatments are typically cytotoxic chemotherapy (combinations of oxaliplatin, irinotecan, fluorouracil, and capecitabine) with or without inhibitors of the epidermal growth factor receptor (EGFR, cetuximab and panitumumab) and vascular endothelial growth factor (VEGF, bevacizumab).

However, only a subset of patients benefit from EGFR inhibitor use, as response to EGFR inhibitors in patients with RAS/BRAF mutant or right-sided mCRC is poor.[4-9] The currently available third-line treatment options for MSS mCRC are regorafenib and trifluridine-tipiracil. However, objective response rate (ORR) is <5% and improvements in progression-free survival (PFS) and overall survival (OS) are minimal, if any (Appendix 4).[10-13] Additional treatment strategies for MSS mCRC are desperately needed.

Pembrolizumab and nivolumab, anti-programmed death-1 (PD-1) monoclonal antibodies, are effective in many patients with microsatellite instability (MSI-H) mCRC, with ORR 30-50%.[14,15] However, MSI-H is present in only about 4% of patients with mCRC.[14,15] Patients with MSS do not respond to single-agent anti-PD-1 agents alone, likely due to immune tolerance. This may be explained by low tumor mutational burden in MSS (resulting in a reduced number of mutation-associated neoantigens) and anti-PD-1 agents may enhance the immunosuppressive function of regulatory T cells [Tregs]).[14,16-18] However, in resected CRC specimens, tumor infiltration by cytotoxic memory T lymphocytes correlates with improved disease-free and overall-survival at all stages, suggesting that immune-based therapeutics may be effective.[16] Novel immunotherapy-based treatment options offer a promising opportunity in MSS mCRC.

2.2.1 PD-1/PD-L1 CHECKPOINT

Immune checkpoint inhibitors have been successful in the treatment of many solid tumors, beginning with the approval of ipilimumab for metastatic melanoma in 2011.[19] Tumor cells can be recognized and destroyed by the immune system, but only after a highly coordinated cascade occurs. To do this, naïve T cells must bind to antigen-bound major histocompatibility complex (MHC) molecules on antigen presenting cells (APCs, such as dendritic cells) and then become activated through (stimulatory and inhibitory) co-interactions.[20,21] Coinhibitory receptors are necessary to allow immune tolerance and avoid autoimmune disease. Activated T cells can then exert effector anti-tumor responses.

There are multiple mechanisms by which tumors can evade the immune system, including secretion of immunosuppressive factors, downregulation of MHC, recruitment of immunosuppressive cells, activation of coinhibitory receptors on T cells, or activation of coinhibitory ligands on tumor.[20] PD-1 is an inhibitory receptor expressed on T cells; binding to its ligand, PD-L1 (also called B7-H1 and CD274), which is expressed in nonlymphoid tissue including tumor cells, inhibits downstream T cell receptor (TCR) signaling and inhibits T cell activation (including migration, proliferation, and function) (Figure 1).[19,22,23] The currently available immune checkpoint inhibitors target cytotoxic T-lymphocyte antigen-4 (CTLA-4), such as ipilimumab, programmed death-1 (PD-1), such as pembrolizumab, nivolumab, and cemiplimab, and its ligand (PD-L1), such as atezolizumab, avelumab, and durvalumab.

Pembrolizumab (Keytruda, MK-3475) is an anti-PD-1 humanized IgG4/kappa monoclonal antibody designed to directly block the interaction between PD-1 and its ligands.[24] Pembrolizumab is currently approved for many advanced solid tumors.[24]

Figure 1. PD-1-PD-L1 checkpoint[25]

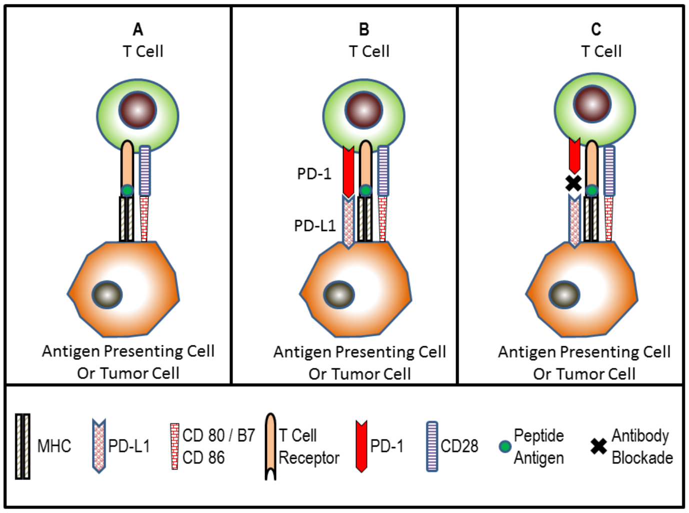

PD-1 mediated T-cell activation (A), exhaustion (B) and blockade (C). (A) APC or tumor cell mediated T- cell activation results in PD-1 expression. (B) PD-L1 binds to PD-1 and "turns off" the T-cell. (C) PD-L1-PD-1 blockade results in persistent activation of the T-cell which then continues to maintain its effector functions.
APC, Antigen presenting cell; MHC, major histocompatibility complex; PD-1, Programmed death-1; PD-L1, programmed death ligand 1; TCR, T-cell receptor.[25]

2.2.2 EPIDERMAL GROWTH FACTOR RECEPTOR

EGFR is a member of the erythroblastosis oncogene b (ErbB)/human epidermal growth factor receptor (HER) family (EGFR is also called ErbB1 and HER1).[20] EGFR is expressed on the surface of cells of epithelial, neuronal, and mesenchymal origin.[26] Activation of EGFR (such as by epidermal growth factor or transforming growth factor beta) results in downstream signaling pathways, including RAS/RAF/MEK/ERK, PI3K/AKT/mTOR, and JAK/STAT3, the end result is cell growth, survival, and migration.[20,26,27]

Cetuximab is an anti-EGFR IgG1 chimeric monoclonal antibody that binds to EGFR and prevents stimulatory downstream signaling.[20,28] It was first approved for mCRC in 2004[29] and has been shown to improve outcomes (variably ORR, DCR, PFS, and OS) in patients with left-sided RAS (including both KRAS and NRAS)/BRAF wild-type mCRC as monotherapy[30] and in combination with chemotherapy.[31-34] However, activating mutations downstream of EGFR, i.e. in RAS (most commonly KRAS, which is present in approximately 40% of mCRC, or NRAS) or BRAF (mutually exclusive with KRAS mutations, present in approximately 5-12% of mCRC, and most of which are V600E) result in constitutive activation of the downstream signaling

pathway, conferring resistance to EGFR inhibitors.[4-9,35-37] Likewise, right-sided tumors respond poorly to EGFR inhibitors, but the biologic reasons for lack of response in right-sided tumors are largely unknown.[38]

In addition to direct effects on tumor cells (such as blocking growth factors, inhibiting downstream signaling, and inducing apoptosis), the IgG1 Fc region of cetuximab strongly interacts with phagocyte Fcγ receptors.[39] Human macrophages indeed express activating Fcγ receptors. This results in cell death via antibody-dependent cellular phagocytosis (ADCP), a promising mechanism in cancer treatment.[39,40] In CRC cell lines, immune-mediated effects of cetuximab have been shown to be independent on KRAS, BRAF, and PIK3CA mutational status, and may correlate with EGFR expression.[41]

EGFR can be qualitatively measured using immunohistochemistry (IHC). While fast and cheap, it is an inconsistent method (inherent subjectivity of interpreting results and results are dependent on the antibody used).[26] EGFR is expressed in up to 80% of patients with CRC.[26,42] However, multiple studies have failed to demonstrate a correlation between EGFR expression by IHC and anti-EGFR efficacy (targeting EGFR signaling pathways), including some patients who respond to anti-EGFR therapy with undetectable EGFR by IHC.[26,43,44] Therefore EGFR IHC (or by other methods) is not used or recommended in clinical practice to select patients for anti-EGFR therapy (targeting EGFR signaling pathways) or to predict response.[26,45] In this present study, EGFR expression will be evaluated as an exploratory endpoint.

2.2.3 SIRP ALPHA/CD47 CHECKPOINT

While anti-PD-1 agents primarily target the adaptive immune system, the innate immune system is a promising novel target. The CD47/signal regulatory protein alpha (SIRPα) axis serves as a myeloid immune checkpoint.[46] SIRPα is an inhibitory receptor expressed on myeloid phagocytic cells (dendritic cells (DCs), macrophages, and neutrophils). SIRPα contains an extracellular immunoglobulin domain for ligand binding and intracellular immunoreceptor tyrosine-based inhibition motifs (ITIM).[47,48] CD47 (also called integrin-associated protein), the ligand for SIRPα, is widely expressed on cancer cells, including CRC.[49] The SIRPα-CD47 interaction results in phosphorylation of the ITIMs, binding and activation of SHP-1 and SHP-2, and disruption of the cytoskeleton, resulting in inhibition of phagocytosis.[49] Blockade of the SIRPα/CD47 axis increases phagocytosis, resulting in cell death and antigen presentation (with secondary adaptive immune system activation). Tumor killing by phagocytes can be further stimulated if an active Fc (fragment crystallizable) region of immunoglobulin is supplied (via antibody-dependent cellular phagocytosis, ADCP), either on the anti-CD47 agent or on a separate anti-cancer antibody.[47]

While CD47 is widely expressed among normal human cells, including hematopoietic cells, pre-clinical models show that blockade of the SIRPα/CD47 axis preferentially results in

phagocytosis of tumor, as normal human cells typically lack stimulatory "eat me" signals.[48,49] A notable exception is red blood cells (RBC), which are subject to dose-dependent phagocytosis in the setting of SIRPα/CD47 blockade.[50] Anemia has been a severely limiting toxicity of other anti-CD47 agents.[48] Strategies to minimize dose-limiting anemia including administering a priming dose of the SIRPα/CD47 blocker (resulting in RBC phagocytosis followed by reticulocytosis; reticulocytes express higher levels of CD47 and are thus resistant to phagocytosis) or selecting the Fc domain of the anti-CD47 monoclonal antibody to minimize interactions with the phagocyte Fcγ receptor (either by using an inactive Fc domain or using an Fc domain of IgG2 or IgG4, which more weakly interact with Fcγ receptors than do IgG1 or IgG3).[39,51]

Evorpacept (ALX148), an anti-CD47 agent, is an engineered fusion protein containing two high affinity CD47 binding domains of SIRPα linked to an inactive Fc region of human immunoglobulin (IgG1; inactivity achieved via amino acid substitutions within IgG1 to eliminate binding to Fcγ receptors and C1q) (Figure 2).[52] It is a 78-kDa disulfide-linked homodimer that has no glycosylation sites.[52] Evorpacept (ALX148) is specifically designed with an inactive Fc domain, to prevent CD47-targeted ADCP of hematopoietic cells (thereby minimizing cytopenias frequently seen with other anti-CD47 agents).[52] In preclinical models, evorpacept (ALX148) inhibits CD47/SIRPα interaction, enhances phagocytosis of tumor cells (via Fc-dependent ADCP, augmented by a second therapeutic agent with an active Fc domain including the EGFR inhibitor cetuximab to provide an "eat me" signal), increases the ratio of inflammatory to suppressive macrophages, and activates the adaptive immune system via antigen presentation by dendritic cells (Figure 3).[52] Increased antigen presentation allows evorpacept (ALX148) to augment the response to anti-PD-1 agents.[52,53] Further, there is evidence that the PD-1/PD-L1 axis serves as an innate immune checkpoint.[48] Thus, blockade of the PD-1/PD-L1 axis may induce not only a T cell mediated response but also a phagocyte response.[48]

Figure 2. Evorpacept (ALX148) structure

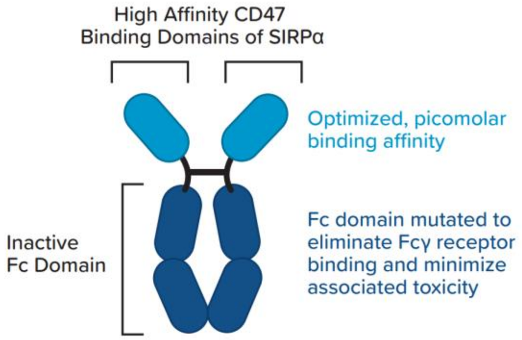

Evorpacept (ALX148) potently and selectively binds CD47 to block SIRPα interaction. It is an engineered fusion protein containing two high affinity CD47 binding domains of SIRPα linked to an inactive Fc region of human immunoglobulin.[54]

Figure 3. Evorpacept (ALX148) mechanisms of action

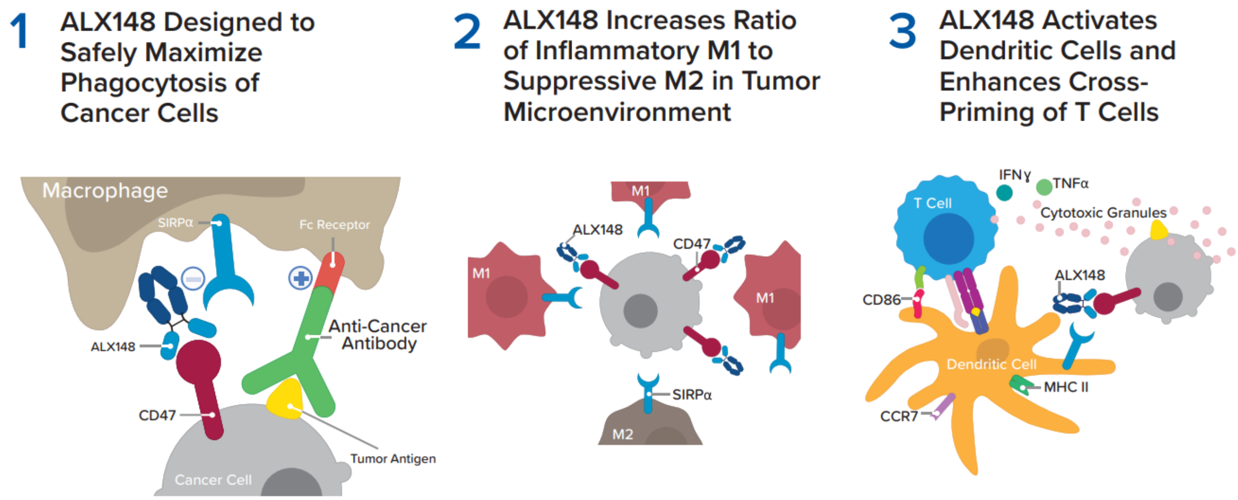

1) Evorpacept (ALX148) binds to CD47, interrupting the inhibitory ("don't eat me") CD47/SIRPα interaction between phagocyte and tumor. An anti-cancer antibody binds to its receptor, providing a stimulatory ("eat me") interaction between phagocyte and tumor. 2) Evorpacept (ALX148) increases the ratio of inflammatory macrophages (M1) to suppressive macrophages (M2) in the tumor microenvironment. 3) Evorpacept (ALX148) activates dendritic cells (as shown in pane 1), resulting in antigen presentation and activation of the adaptive immune system.[52,54]

2.2.4 SUMMARY

We hypothesize that the combination of evorpacept (ALX148), cetuximab, and pembrolizumab will demonstrate promising efficacy via synergistic anti-tumor activation of the innate and adaptive immune systems (i.e., overcome resistance to an immunologically "cold" tumor) and will be safe/tolerable in patients with refractory MSS mCRC. The anti-EGFR monoclonal antibody cetuximab provides the "eat me" signal and promotes ADCP, augmenting the innate immune response to evorpacept (ALX148), resulting in tumor cell death by phagocytosis (macrophages and neutrophils). Dendritic cell activation similarly occurs, resulting in antigen presentation and activation of the adaptive immune system, further augmented by the anti-PD-1 monoclonal antibody pembrolizumab. Here, the desired mechanism of action of cetuximab is to augment phagocytosis via ADCP rather than to modulate the EGFR signaling pathway. As noted, immune-mediated effects of cetuximab have been shown to be independent of tumor mutational status, allowing patients to be enrolled independent of RAS/BRAF status. Immune-mediated effects of cetuximab may correlate with EGFR expression and this will be explored. It is expected that patients anticipated to respond to EGFR signaling pathway inhibition will have already received directed treatment (i.e. left-sided and RAS/BRAF WT) and other patients are expected to respond poorly to EGFR signaling pathway inhibition.

2.3 RISK/BENEFIT ASSESSMENT

2.3.1 KNOWN POTENTIAL RISKS

Every drug has its own set of side-effects, and the risk in combining multiple agents is an increase in the number and intensity of adverse events. The potential risks and benefits of this combination therapy are listed below. See the Investigator's Brochure for the most current risk profile for evorpacept (ALX148), cetuximab, and pembrolizumab.

Evorpacept (ALX148)

Evorpacept (ALX148) has been shown to be generally well-tolerated in phase I studies in other cancer types, as monotherapy and in combination with pembrolizumab, anti-cancer antibodies, and cytotoxic chemotherapy.

Evorpacept (ALX148) monotherapy

In a phase I study presented at ASCO 2018, 28 patients with advanced solid tumors and non-Hodgkin lymphoma were treated with single agent evorpacept (ALX148).[55] The most common treatment-related adverse events (TRAEs) were headache (17%), fatigue (14%), thrombocytopenia (11%), increased AST (7%), increased ALT (7%), dizziness (7%), pruritus (7%), and rash (7%). There were 6 episodes of grade ≥ 3 TRAEs: grade 3 thrombocytopenia (2), grade 3 pancreatitis (1), grade 5 death (1, unknown etiology), grade 3 infection (1), and grade 4 neutropenia (1)).

Evorpacept (ALX148) combination therapy

In a phase I study presented at ASCO 2019 and updated at ASCO 2020, patients with advanced refractory solid tumors were treated with evorpacept (ALX148) in combination with pembrolizumab (non-small cell lung cancer (NSCLC) and head/neck squamous cell carcinoma (HNSCC), N=52) or evorpacept (ALX148) in combination with trastuzumab ($HER2^+$, primarily gastric/gastroesophageal junction carcinoma, N=30).[54,56] Among patients treated with evorpacept (ALX148) and pembrolizumab, any grade TRAEs included AST increased (17%), ALT increased (14%), fatigue (12%), anemia (10%), pruritus (10%), rash (10%), infusion reaction (8%), arthralgia (6%), pyrexia (6%), leukopenia (6%), decreased appetite (4%), myalgia (4%), nausea (4%), and neutropenia (4%). Grade ≥3 TRAEs included thrombocytopenia (4%) and 2% each ALT increased, anemia, and neutropenia. Among patients treated with evorpacept (ALX148) and trastuzumab, any grade TRAEs included fatigue (30%), thrombocytopenia (17%), decreased appetite (10%), pruritus (10%), pyrexia (10%), anemia (7%), neutropenia (7%), and nausea (7%). Grade ≥3 TRAEs included thrombocytopenia (7%) and neutropenia (6%).

Any grade TRAEs included fatigue (11%), AST increase (9%), ALT increase (8%), anemia (8%), and thrombocytopenia (6%).

Pembrolizumab

Pembrolizumab has been extensively evaluated in many cancer types, both as monotherapy and in combination. Safety data is summarized in the pembrolizumab Investigator Brochure and Package Insert.[24,57] The Reference Safety Database (RSD, N=2799) is a locked and verified dataset with pooled data from clinical studies of pembrolizumab monotherapy in participants with melanoma and NSCLC, as part of the Sponsor's product development of pembrolizumab. The RSD comprises safety data from Studies KN001, KN002, KN006, and KN010 locked databases. The Cumulative Running Safety Dataset (CRSD, N=8093) is a larger, locked aggregate safety dataset that is a cumulative, pooled safety data summary tabulation of AEs from clinical studies of pembrolizumab monotherapy that have been used to support regulatory applications.

Pembrolizumab monotherapy is well tolerated in the approved indications, as evidenced by a low rate of grade ≥ 3 drug-related AEs (13.8%), discontinuations due to AEs (11.9%), and deaths due to drug-related AEs (0.4%) in the RSD. Furthermore, the frequency of immune-mediated AEs is low, and these events are usually readily managed in the clinical setting.

The majority of participants in the RSD (n=2727; [97.4%]) experienced 1 or more AEs, 2062 (73.7%) participants experienced 1 or more AEs reported as drug-related by the investigator, 1042 (37.2%) participants experienced 1 or more serious AE (SAE); 282 (10.1%) participants experienced a SAE reported as drug-related by the investigator. The most frequently reported AEs from the RSD were fatigue (37.3%), nausea (24.5%), decreased appetite (22.5%), diarrhea (22.3%), and cough (22%), and 334 (11.9%) participants discontinued due to an AE. The 5 most frequently reported SAEs in the RSD were pneumonia (3.0%), pleural effusion (1.7%), pneumonitis (1.6%), dyspnea (1.6%), and pulmonary embolism (1.5%). The most frequently reported AEs in the RSD considered drug-related were fatigue (24.2%), pruritus (17.3%), rash (14.1%), diarrhea (12.3%), and nausea (10.9%). These were also the most frequently reported AEs (in addition to hypothyroidism) considered drug-related in the CRSD. Pneumonitis was the most common event leading to discontinuation of pembrolizumab in both the RSD (1.2%) and CRSD (1.5%). Drug-related events leading to death are very uncommon (0.4% in RSD and 0.8% in CSRD).

The Pembrolizumab Investigator's Brochure (Edition 23, Release Date 26-Oct-2022) and U.S. Food and Drug Administration Pembrolizumab Package Insert list additional adverse events of special interest, including those identified in the postmarketing setting, including: hypothyroidism, hyperthyroidism, pneumonitis, infusion reaction, colitis, severe skin reaction, adrenal insufficiency, hepatitis, hypophysitis, thyroiditis, uveitis, myositis, nephritis, pancreatitis,

type 1 diabetes mellitus, Guillain-Barre syndrome, myasthenic syndrome, sarcoidosis, vasculitis, encephalitis, hypoparathyroidism, myelitis, optic neuritis, arthritis, Vogt-Koyanagi-Harada syndrome, hemolysis, and hemophagocytic lymphohistiocytosis.

Cetuximab

In a phase II study of cetuximab monotherapy in patients with mCRC, 57 patients were treated.[58] The most common grade 3-4 adverse events (regardless of attribution) were acne-like rash (86% any grade, 18% grade 3, 0% grade 4), a composite of asthenia, fatigue, malaise, or lethargy (56% any grade, 9% grade 3), and allergic reaction (5% grade 3).

In a phase III study comparing cetuximab monotherapy to cetuximab + irinotecan, 111 patients received cetuximab monotherapy.[29] In the cetuximab monotherapy group, grade 3-4 TRAEs occurred in 44% of patients and most commonly were dyspnea (13%), asthenia (10%), abdominal pain (6%), acne-like rash (6%), nausea/vomiting (5%), hypersensitivity reaction (4%), and anemia (3%)

In a phase III study comparing cetuximab + best supportive care (BSC) versus BSC alone, 287 patients received cetuximab.[30] There were no statistically significant differences between the cetuximab and BSC alone groups in grade ≥ 3 AEs, other than rash (12% in cetuximab group), infection (13%), confusion (6%), and pain (15%). Of note, there was no increase in hematologic AEs in the cetuximab group. The most common AEs of any grade in the cetuximab group were rash (89%), hypomagnesemia (53%), and infusion reaction (21%).

Tumor biopsies

Patients in Cohort A will undergo a tumor biopsy pre- and post- treatment. Archival tissue may not replace the pre-treatment biopsy. Risks of biopsies include but are not limited to pain, bleeding, infection, and damage to nearby structures. Care will be taken to minimize risk to each patient by identifying the safest biopsy target. See Section 4.1.4 for additional information regarding tumor biopsies.

2.3.2 KNOWN POTENTIAL BENEFITS

Evorpacept (ALX148), cetuximab, and pembrolizumab

This study regimen combines three agents with the goal of synergistically generating anti-tumor innate and adaptive immune responses.

Evorpacept (ALX148) non-clinical data is summarized in a 2018 publication.[52] In cell line experiments, evorpacept (ALX148) was shown to bind human and mouse CD47 with high affinity and prevent its interaction with SIRPα; further, evorpacept (ALX148) did not bind to

either Fcγ receptor or C1q. In mice, hematologic parameters (white blood cell, hemoglobin, and platelets) were not affected when treated with evorpacept (ALX148).

In cell lines, evorpacept (ALX148) enhanced macrophage-mediated ADCP when given with various anti-cancer antibodies (including cetuximab and also trastuzumab, daratumumab, and obinutuzumab). Patient-derived tumor xenograft mice models showed that evorpacept (ALX148) enhanced antitumor activity and extended survival when given with anticancer antibodies (obinutuzumab, trastuzumab, or rituximab; note that these mice lacked adaptive immune systems, limiting full assessment of evorpacept [ALX148] efficacy).

To address this limitation, immunocompetent syngeneic mice were treated with evorpacept (ALX148) and anti-PD-1; evorpacept (ALX148) enhanced anti-tumor efficacy compared with anti-PD-1 alone, suggesting augmentation of the anti-tumor adaptive immune response. evorpacept (ALX148) cell line experiments showed an increase (approximately 3-fold) in the ratio of M1 (inflammatory) / M2 (suppressive) macrophages in the tumor microenvironment. Mouse experiments showed evorpacept (ALX148) induced DC activation and expanded/activated splenic CD4+ and CD8+ T cell populations (the latter either alone or in combination with anti-PD-1). In summary, this data suggests that evorpacept (ALX148), cetuximab, and pembrolizumab will be able to overcome resistance to anti-PD-1 monotherapy in patients with MSS mCRC by 1) direct cell death by the innate immune system; and 2) increased antigen presentation and subsequent activation of the adaptive immune system, resulting in cell death (i.e. tipping the balance to a more stimulatory than inhibitory anti-tumor immune state).

Clinical data also supports synergy between evorpacept (ALX148) and pembrolizumab. Fifty-two patients treated with evorpacept (ALX148) and pembrolizumab (HNSCC and NSCLC) have been reported to date, including patients with partial response.[54] Paired tumor biopsies (pre- and post-treatment) demonstrated increased tumor infiltration of both innate and adaptive immune cells.[59]

Cetuximab, alone or in combination with chemotherapy, has been shown to improve outcomes in patients with left-sided RAS/BRAF WT mCRC. Pembrolizumab has been shown to improve outcomes in patients with MSI-H mCRC and in many other cancer types.

Tumor biopsies

Patients in Cohort A will undergo a tumor biopsy pre- and post- treatment. Since the advent of the targeted therapy era it has become clear that a better understanding of the intimate biological mechanisms that tailor the cancer cell phenotype would be translated into a better chance of disease control. However, this has been a difficult task due to the complex interaction between the tumor cell, tumor stroma, and immune response. The animal models do not appropriately represent these intricate interactions, especially for the immune system.[60] Therefore, tumor

biopsies during the clinical study at different time-points present the best opportunity to increase understanding of the anti−tumor immune response as well as to identify biomarkers predictive of response and resistance. Study subjects will not be discontinued from the study if a biopsy cannot be safely obtained or the patient refuses. See Section 4.1.4 for additional information regarding tumor biopsies.

2.3.3 ASSESSMENT OF POTENTIAL BENEFITS

Based on the potential benefits of the study regimen, well-defined and tolerable safety profile of each agent, and availability of standard of care therapy if disease progresses, the Sponsor-Investigator believes that in this study the potential benefits outweigh its risks.

The risks to participants are reasonable in relation to the anticipated benefits to participants and/or society, and in relation to the importance of the knowledge that may reasonably be expected to result, thereby falling in favor of performing the study:

- To Participant: the benefit of possible short term and sustained disease response resulting in improvement in both quantity and quality of life.
- To Society: the knowledge gained regarding toxicity and efficacy will guide treatment for other patients with mCRC.
- Justify the importance of the knowledge gained: the knowledge gained regarding toxicity and efficacy will help determine future directions of research and treatment in the area of mCRC.

2.4 KNOWN PHARMACOKINETICS

2.4.1 PEMBROLIZUMAB PHARMACOKINETICS

The pharmacokinetics of pembrolizumab are described in the package insert.[57]

The pharmacokinetics (PK) of pembrolizumab were characterized using a population PK analysis with concentration data collected from 2993 patients with various cancers who received pembrolizumab doses of 1 to 10 mg/kg every 2 weeks, 2 to 10 mg/kg every 3 weeks, or 200 mg every 3 weeks.

Steady-state concentrations of pembrolizumab were reached by 16 weeks of repeated dosing with an every 3-week regimen and the systemic accumulation was 2.1-fold. The peak concentration (Cmax), trough concentration (Cmin), and area under the plasma concentration versus time curve at steady state (AUCss) of pembrolizumab increased dose proportionally in the dose range of 2 to 10 mg/kg every 3 weeks.

<u>Distribution</u>

The geometric mean value (CV%) for volume of distribution at steady state is 6.0 L (20%).

Elimination

Pembrolizumab clearance (CV%) is approximately 23% lower [geometric mean, 195 mL/day (40%)] at steady state than that after the first dose [252 mL/day (37%)]; this decrease in clearance with time is not considered clinically important. The terminal half-life (t1/2) is 22 days (32%).

Specific Populations

The following factors had no clinically important effect on the CL of pembrolizumab: age (range: 15 to 94 years), sex, race (89% White), renal impairment (eGFR ≥ 15 mL/min/1.73 m2), mild hepatic impairment (total bilirubin ≤ upper limit of normal (ULN) and AST > ULN or total bilirubin between 1 and 1.5 times ULN and any AST), or tumor burden. The impact of moderate or severe hepatic impairment on the pharmacokinetics of pembrolizumab is unknown.

2.4.2 CETUXIMAB PHARMACOKINETICS

The pharmacokinetics of cetuximab are described in the package insert.[61]

Cetuximab administered as monotherapy or in combination with concomitant chemotherapy or radiation therapy exhibits nonlinear pharmacokinetics. The area under the concentration time curve (AUC) increased in a greater than dose proportional manner while clearance of cetuximab decreased from 0.08 L/h/m2 to 0.02 L/h/m2 as the dose increased from 20 mg/m^2 to 200 mg/m^2 and plateaued at doses >200 mg/m^2.

The systemic exposure of cetuximab after ERBITUX administration was 22% (90% CI: 6%, 38%) higher than that of another cetuximab product used in EXTREME and CRYSTAL.

Distribution

The volume of the distribution for cetuximab appeared to be independent of dose and approximated the vascular space of 2–3 L/m^2.

Elimination

Following the recommended dosage (400 mg/m^2 initial dose; 250 mg/m^2 weekly dose), concentrations of cetuximab reached steady-state levels by the third weekly infusion with mean peak and trough concentrations across studies ranging from 168 µg/mL to 235 µg/mL and 41 µg/mL to 85 µg/mL, respectively. The mean half-life of cetuximab was approximately 112 hours (63 to 230 hours).

Specific Population

Age, sex, race, hepatic, and renal function had no clinically significant effect on the pharmacokinetics of cetuximab. Clearance of cetuximab increased 1.8-fold as body surface area

increased from 1.3 m^2 to 2.3 m^2, which is consistent with the recommended dosing of cetuximab on mg/m^2 basis.

Drug Interaction Studies

No pharmacokinetic interaction was observed between cetuximab and irinotecan, cetuximab and cisplatin, and cetuximab and carboplatin.

2.4.3 EVORPACEPT (ALX148) PHARMACOKINETICS

The pharmacokinetics of evorpacept (ALX148) are described in the Investigator's Brochure.[62]

3 OBJECTIVES AND ENDPOINTS

Primary Objective:

1. To determine the recommended dose (RD) of evorpacept (ALX148) in combination with cetuximab and pembrolizumab
2. To determine the objective response rate (ORR), defined as partial response or complete response, with evorpacept (ALX148), cetuximab, and pembrolizumab using RECIST v1.1 in patients with microsatellite stable (MSS) metastatic colorectal cancer (mCRC) who have progressed on at least two lines of standard therapy

Secondary Objectives:

1. To determine the disease-control rate (DCR), defined as stable disease, partial response, or complete response with evorpacept (ALX148), cetuximab, and pembrolizumab using RECIST v1.1
2. To determine the duration of response (DOR) with evorpacept (ALX148), cetuximab, and pembrolizumab, defined as the time from response (partial or complete) to progression using RECIST v1.1 or death from any cause
3. To determine the progression-free survival (PFS) with evorpacept (ALX148), cetuximab, and pembrolizumab, defined as the time from enrollment to the first observation of progression using RECIST v1.1 or death from any cause
4. To determine the overall survival (OS) with evorpacept (ALX148), cetuximab, and pembrolizumab, defined as the time from enrollment to death from any cause
5. To determine the first cycle dose-limiting toxicities (DLT) of evorpacept (ALX148), cetuximab, and pembrolizumab in stage 1
6. To evaluate the safety and tolerability of evorpacept (ALX148), cetuximab, and pembrolizumab, defined and graded according to the NCI CTCAE v5.0

Exploratory Objective:

1. To compare the objective response rate (ORR), disease-control rate (DCR), duration of response (DOR), and progression-free survival (PFS) determined according to RECIST v1.1 to that identified by iRECIST
2. To identify immune modulation (myeloid and lymphoid) in the peripheral blood and tissue biopsies pre- and post-treatment with evorpacept (ALX148), cetuximab, and pembrolizumab
3. To correlate immune modulation in the peripheral blood and tissue biopsies pre- and post-treatment with evorpacept (ALX148), cetuximab, and pembrolizumab with response
4. To evaluate the relationship between PD-L1, EGFR, and CD47 tumor expression and efficacy of evorpacept (ALX148), cetuximab, and pembrolizumab
5. To evaluate the relationship between tumor mutational burden (TMB) and efficacy of evorpacept (ALX148), cetuximab, and pembrolizumab

Primary Endpoint:

1. Recommended dose (RD) of evorpacept (ALX148) in combination with cetuximab and pembrolizumab
2. Objective response rate (ORR, per RECIST v1.1)

Secondary Endpoints:

1. Disease control rate (DCR, per RECIST v1.1)
2. Duration of response (DOR, per RECIST v1.1)
3. Progression-free survival (PFS, per RECIST v1.1)
4. Overall survival (OS)
5. First-cycle dose-limiting toxicities (DLTs) in stage 1
6. Safety and tolerability defined and graded according to the NCI CTCAE v5.0

Exploratory endpoints:

1. Objective response rate (ORR, per iRECIST), disease-control rate (DCR, per iRECIST), duration of response (DOR, per iRECIST), and progression-free survival (PFS, per iRECIST)
2. Change in pre- and post-treatment levels and activation of immune cells in the peripheral blood and tumor microenvironment
3. Change in pre- and post-treatment mRNA expression in a panel of immune-related genes in tumor tissues
4. Objective response rate (by RECIST v1.1 and iREICST) according to change in pre- and post-treatment levels and activation of immune cell in the peripheral blood and tumor microenvironment
5. Objective response rate (by RECIST v1.1 and iRECIST) according to pre-treatment PD-L1, EGFR, and CD47 tumor expression by immunohistochemistry

6. Objective response rate (by RECIST v1.1 and iRECIST) according to tumor mutational burden (TMB)

4 STUDY DESIGN

4.1 OVERALL DESIGN

4.1.1 STUDY OVERVIEW

This is an open-label, multi-center, single-arm phase II clinical trial (with safety run-in) evaluating the combination of evorpacept (ALX148), cetuximab, and pembrolizumab in patients with metastatic microsatellite stable colorectal cancer who have progressed on at least 2 lines of systemic therapy. A patient who progressed on a single line of therapy including a fluoropyrimidine, oxaliplatin, and irinotecan for unresectable metastatic colorectal adenocarcinoma (e.g., FOLFIRINOX or FOLFOXIRI) is eligible. Patients will be eligible regardless of tumor sidedness or RAS/BRAF status, as the desired mechanism of action of cetuximab is to generate ADCP in synergy with evorpacept (ALX148). However, there is one exception. Patients with left-sided (at or distal to the splenic flexure) RAS/BRAF WT mCRC who are EGFR inhibitor naïve will be excluded. EGFR inhibitor (cetuximab or panitumumab) monotherapy[30,63] and in combination with chemotherapy[31-34,64,65] has been shown to improve outcomes in patients with left-sided RAS/BRAF WT mCRC (variably ORR, DCR, PFS, and OS). However, patients with RAS/BRAF mutations[4,5] and/or right-sided tumors[8,38,66] respond poorly to EGFR inhibitors. Therefore, patients with left-sided, RAS/BRAF WT mCRC who are EGFR-inhibitor naïve would be expected to benefit from cetuximab alone or in combination with chemotherapy (due to EGFR signaling pathway inhibition) and will be excluded from this study. Baseline tumor EGFR expression will be evaluated as an exploratory endpoint.

There are 2 stages to this study:

1. Stage 1: safety run-in (N = no more than 18 evaluable)
2. Stage 2: dose expansion (N = 42), with two cohorts – Cohort A (N=23, will undergo paired pre- and post-treatment biopsies) and Cohort B (N=19, will not undergo paired biopsies)

Protocol amendment version 2 (dated December 28, 2022): The study DSMC will meet after N=12 patients in Stage 2 have been treated for at least one cycle each to review the totality of available safety data; study enrollments and treatments will continue around this review, unless otherwise communicated by the DSMC.

Using a Simon-like 2-stage design, ORR will be determined based on the first 24 evaluable patients (including patients in both Stage 1 and Stage 2). If there are no responses (partial response or complete response as defined in the protocol) in the first 24 evaluable patients, then study enrollment will be stopped for futility. If there is at least one response (partial response or complete response as defined in the protocol) in the first 24 evaluable patients then full study enrollment will proceed as planned. If there are no responses (partial response or complete response as defined in the protocol) in the first 23 evaluable patients then enrollment will be temporarily paused to allow response evaluation of the 24th evaluable patient after up to 15 total weeks (ie 5 total cycles) of study therapy. If the 24th patient has response (partial response or complete response as defined in the protocol) then then full study enrollment will proceed as planned. If the 24th patient does not have a response (partial response or complete response as defined in the protocol) then the study will be stopped for futility. See Section 9.4.2 for statistical considerations.

A subset of patients (Cohort A) will undergo paired pre- and post- (around C3D1) treatment biopsies. See Section 4.1.4 for additional information regarding tumor biopsies.

There will be a 4-week window during which screening can occur. Patients in both the safety run-in and dose expansion stages will continue to receive study therapy until disease progression according to RECIST v1.1 (Appendix 2), unacceptable toxicity, death, patient or physician decision to withdraw, or pregnancy, whichever occurs first. The maximum duration of pembrolizumab is 35 cycles. There is no maximum duration of evorpacept (ALX148) or cetuximab.

4.1.2 CRITERIA FOR CONTINUATION OF TREATMENT BEYOND RECIST V1.1-DEFINED PROGRESSION

In all cohorts, at the time of RECIST v1.1-defined progression (i.e., iUPD), treatment is permitted to continue until the next assessment (at least 4 weeks but no longer than 8 weeks later) if the following criteria are met:

- Patient is clinically stable (An assignment of clinical stability requires that no worsening of performance status has occurred, that no clinically relevant increases in disease-related symptoms such as pain or dyspnea occur that are thought to be associated with disease progression (these symptoms are generally understood to mean a requirement for increased palliative intervention), and that no requirement for intensified management of disease-related symptoms exists, including increased analgesia, radiotherapy, or other palliative care.)
- Absence of tumor progression at critical anatomical sites (e.g., leptomeningeal disease) that cannot be managed by protocol-allowed medical interventions
- Patient and investigator agree to continue treatment

- Patient signs the Treatment Beyond Progression Informed Consent Form
- If the subsequent scan continues to show progression, all therapy will be discontinued and the patient will be taken off study.

At least 28 days and no more than 35 days after discontinuation of treatment, patients will return to undergo the listed activities in Schedule of Events (section 1.3, unless already completed in the last week, or last 6 weeks for response assessment). Patients continuing to experience toxicity at this point following discontinuation of treatment will continue to be followed at least every 4 weeks until resolution or determination, in the clinical judgment of the Investigator, that no further improvement is expected. If the patient has any concern and contacts the clinic, the patient will be seen in the clinic within 5 calendar days of the patient's contact with the clinic (assessments will be the same as the assessments performed at the Final Study/Treatment Discontinuation Visit). Overall survival information will be collected via telephone calls and/or clinic visits every 3 months ±14 days (first call and/or clinic visit to occur 3 months following the Final Study/Treatment Discontinuation Visit) until death, withdrawal of consent, the patient is lost to follow-up, study termination by AGICC, or a maximum of 2 years from the Final Study/Treatment Discontinuation Visit.

Study Schema

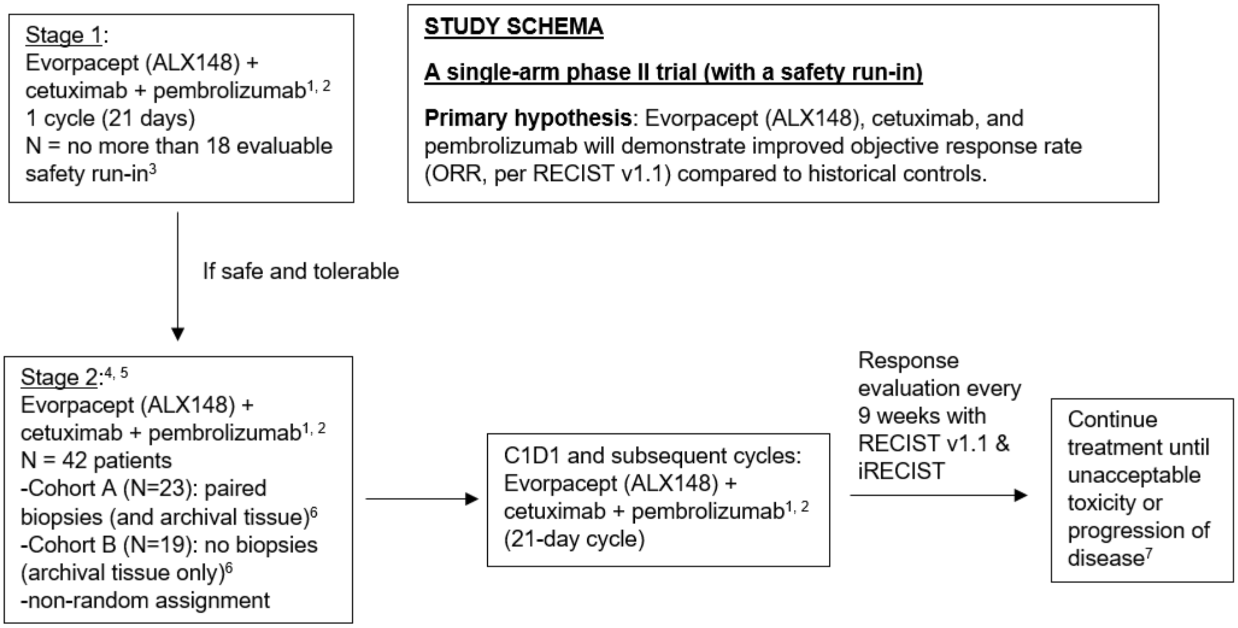

1. Dose Levels:
   - Evorpacept (ALX148) dose level (DL) 1: 15 mg/kg weekly; DL -1: 10 mg/kg weekly; DL -2: 5 mg/kg weekly
   - Cetuximab DL 1: 250 mg/m^2 weekly; DL -1: 200 mg/m^2 weekly; DL -2: 150 mg/m^2 weekly
   - Pembrolizumab DL 1: 200 mg every 3 weeks (no additional dose levels)
2. The desired mechanism of action of cetuximab is to augment phagocytosis via antibody-dependent cellular phagocytosis (ADCP) rather than to modulate the EGFR signaling pathway.

3. No more than 18 evaluable patients will be accrued in Stage 1. A single patient will be treated with evorpacept (ALX148), cetuximab, and pembrolizumab, followed by a 48-hour safety observation period prior to treating the next patient. A 48-hour safety observation period will be required between the start of treatment for patients in Stage 1. Additional patients may be enrolled at lower dose levels if at least 33% of patients experience a first-cycle dose limiting toxicity.
4. If a significant portion of patients in Stage 2 experience AEs meeting dose-limiting toxicity (DLT) criteria as defined in Section 4.1.5, then dose reduction(s) of evorpacept (ALX148) and/or cetuximab for subsequent patients can be considered in discussion with the Investigators and Medical Monitor.
5. A Simon-like 2-stage design will be used. If there are no responses (partial response or complete response) among the first 24 evaluable patients (including patients in both Stage 1 and Stage 2), study enrollment will be stopped for futility. Otherwise, full study enrollment will continue as planned.
6. Patients in Cohort A will provide a fresh pretreatment biopsy sample (before C1D1 but after screening) and an on-treatment biopsy sample at C3D1 (±3 days). While the acceptable window for the C3D1 biopsy is ±3 days, it is preferred for the biopsy to occur following all treatments on C3D1. The date and time of the biopsies will be documented. In Cohort A, archival tissue may not be used in place of a fresh pre-treatment biopsy. Patients in Cohort B will not undergo any study-mandated biopsies. When available, archival tissue will also be obtained for analysis for patients in both Cohort A and Cohort B. For a subject in Cohort A, if the Investigator judges the risk for the pre- or on-treatment biopsy to be unacceptable in a research setting eg, based on complications during a previous procedure, use of blood thinning medications, or inaccessible tumor, or the patient refuses, a subject should not undergo the pre- or on-treatment biopsy and the patient will be allowed to enroll in or remain on the study. If a patient initially assigned to Cohort A is unable to undergo both the pretreatment and on-treatment biopsies due to medical reasons or patient refusal, an additional patient may be enrolled in Cohort A (to achieve N=23 patients who underwent pretreatment and on-treatment biopsies). Other than biopsies, there are no differences between Cohort A and Cohort B (both receive the same treatment and study events). Details for handling of these samples including processing, storage, and shipment will be provided in the Laboratory Manual.
7. The maximum duration of pembrolizumab is 35 cycles. There is no maximum duration of evorpacept (ALX148) or cetuximab.

4.1.3 STAGE 1: SAFETY RUN-IN

The original protocol (version 1, dated 12/22/21) specified that approximately six patients (no more than 18, accounting for the possible need for additional dose levels) will be accrued in Stage 1. A single patient will be treated with evorpacept (ALX148)15 mg/kg weekly, cetuximab (400 mg/m^2 cycle 1 day 1 followed by 250 mg/m^2 weekly), and pembrolizumab (200 mg every 3 weeks), followed by a 48-hour safety observation period prior to treating the next patient. A 48-hour safety observation period will be required between the start of treatment for patients in Stage 1. One cycle will be 21 days. Evorpacept (ALX148) dosing is based on prior phase I data in which no maximum tolerated dose (MTD) was reached alone or in combination (maximum administered dose 15 mg/kg weekly with pembrolizumab and chemotherapy and 15 mg/kg weekly with rituximab and trastuzumab) and treatment was well tolerated.[53-55,67] Cetuximab and pembrolizumab dosing is based on standard dosing. Once 6 patients have been enrolled into the study, further accrual to the study will be temporarily halted while the study team reviews the totality of the first-cycle clinical data to determine safety and tolerability of the regimen. The safety review should contain data from patients who have been receiving the regimen for a minimum of one cycle of treatment.

Further enrollment and study treatments will be halted if either of the following occur:

- Any patient experiences death due to an adverse event that is assessed as related to study treatment (by investigator and/or AGICC)
- At least 33% of patients experience a DLT

If these doses are not tolerable (i.e. at least 33% of patients experience a first-cycle DLT, see below), then 3 additional patients will be enrolled in dose level -1 which will comprise the same doses of cetuximab and pembrolizumab with a lower dose of evorpacept (ALX148) (10 mg/kg weekly). The cetuximab dose may be reduced (to 200 mg/m^2 weekly) if DLT(s) are incontrovertibly attributable to cetuximab, after review by AGICC and the Investigators. If no DLTs are observed, dose level -1 will be the recommended Stage 2 dose. If 1 DLT is observed, then additional patients will be enrolled at dose level -1. If, after enrolling additional patients at dose level -1, at least 33% of patients experience a DLT at dose level -1, then 3 patients will be enrolled in dose level -2 which will comprise the same doses of cetuximab and pembrolizumab with a lower dose of evorpacept (ALX148) (5mg/kg weekly). The cetuximab dose may be reduced (to either 200 mg/m^2 weekly or 150 mg/m^2 weekly, depending on the prior cetuximab dose level) if DLT(s) are incontrovertibly attributable to cetuximab, after review by AGICC and the Investigators. In the safety run-in, cetuximab dose adjustments are not allowed unless the DLT(s) is incontrovertibly attributable to cetuximab. If no DLTs are observed, dose level -2 will be the recommended Stage 2 dose. If 1 DLT is observed, then additional patients will be enrolled at dose level -1. If, after enrolling additional patients at dose level -2, at least 33% of patients experience a DLT at dose level -2, the study will end.

Protocol amendment version 2 (dated December 28, 2022)

During Stage 1 of this study, six patients were administered evorpacept (ALX148) 15 mg/kg, weekly in combination with pembrolizumab and cetuximab. One patient experienced a DLT of grade 5 HLH (see Section 2.3.1) that was assessed as possibly related to ALX148, pembrolizumab and cetuximab and the study was temporarily paused per protocol guidance. Three additional patients had completed the full DLT assessment period (all cycle 1 study treatments) and two additional patients did not complete all cycle 1 doses without reporting a DLT. Protocol amendments included in version 2 (dated December 28, 2022) dictate modification to Stage 1 when the study re-opens to enrollment in response to this event. Stage 1 will evaluate an additional patient cohort (N=3) administered evorpacept (ALX148) dose level -1 (10 mg/kg weekly) and dose level 1 of both cetuximab (400 mg/m2 cycle 1 day 1 followed by 250 mg/m2 weekly) and pembrolizumab (200 mg every 3 weeks). If there are no DLTs among these 3 patients, then N=3 additional patients will be enrolled at evorpacept dose level 1 (15 mg/kg weekly) with the same doses of cetuximab and pembrolizumab. If there is one DLT among the initial 3 patients enrolled at evorpacept (ALX148) dose level -1 (evorpacept 10 mg/kg), a further 3 patients will be enrolled at that dose level. Escalation to dose level 1 (evorpacept 15 mg/kg) should only be permitted if either 0 of 3 or 1 of 6 patients experience a DLT at evorpacept (ALX148) dose level -1. This follows a standard '3+3' study design at

ALX148 dose level -1 (evorpacept 10 mg/kg) upon re-initiation of the study. Consistent with protocol version 1, the study will proceed to Stage 2 at doses where less than 33% of patients experience a first-cycle DLT. See Figure 6 for additional details on the management of Stage 1 under protocol version 2. Note that in the event of a further DLT in 1 of 3 patients at evorpacept (ALX148) dose level 1 (evorpacept 15 mg/kg) i.e., 2 of 7 patients treated at dose level 1 (including N=4 patients treated under protocol version 1 dated 12/22/21), a complete safety evaluation including assessment of late onset toxicities should be reviewed by the data safety monitoring committee (DSMC) prior to proceeding to Stage 2. This DSMC review would be in addition to the DSMC review after N=12 patients in Stage 2 have been treated for at least one cycle as noted in Section 4.1.1.

Figure 6. Modification to Stage 1 with Protocol Version 2 (dated December 28, 2022)

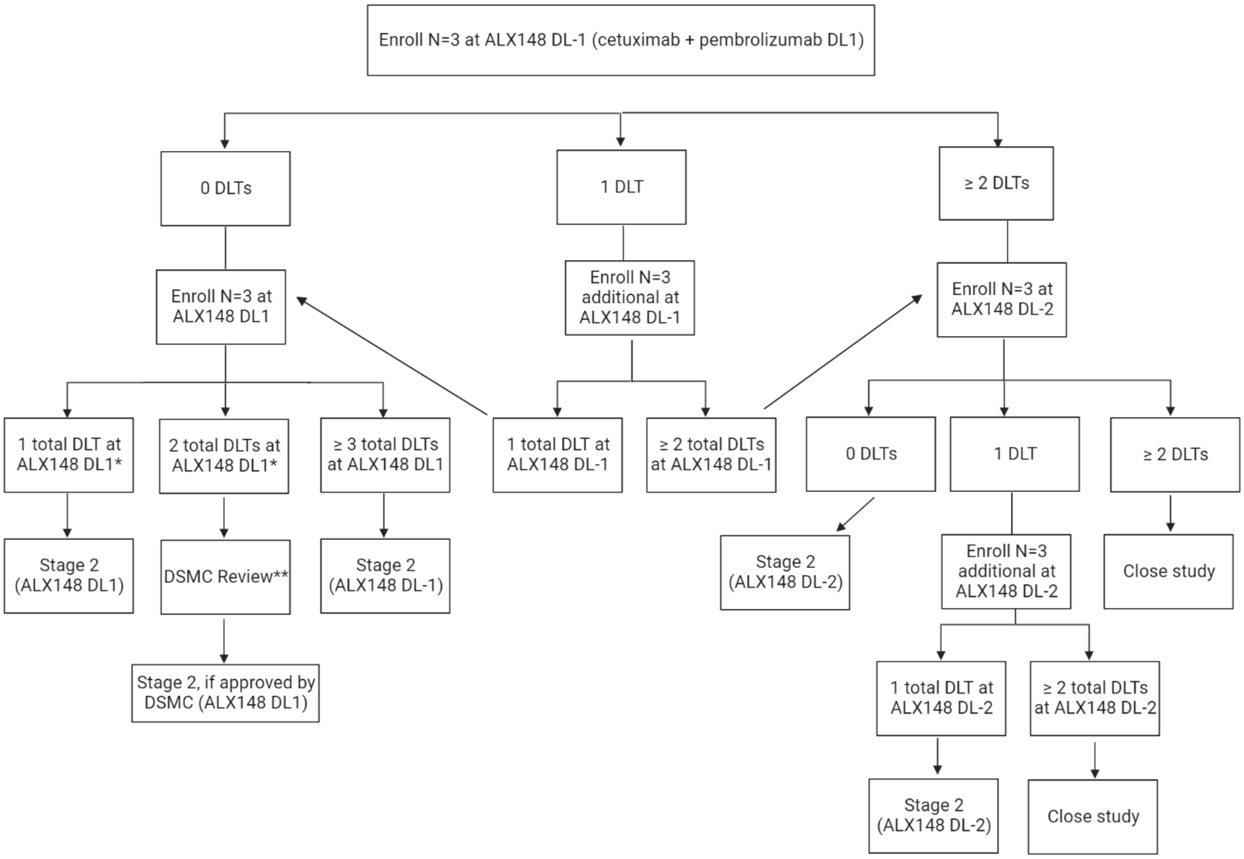

Abbreviations: DL, dose level; DLT, dose-limiting toxicity; DSMC, data safety monitoring committee.
*Includes N=4 patients treated with evorpacept (ALX148) dose level 1 under protocol version 1 (dated 12/22/21) that are DLT evaluable (including 1 DLT [HLH].
** In the event of a further DLT in 1 of 3 patients at evorpacept (ALX148) dose level 1 (evorpacept 15 mg/kg) i.e., 2 of 7 patients treated at dose level 1 (including N=4 patients treated under protocol version 1 dated 12/22/21), a complete safety evaluation including assessment of late onset toxicities should be reviewed by the data safety monitoring committee (DSMC) prior to proceeding to Stage 2. This DSMC review would be in addition to the DSMC review after N=12 patients in Stage 2 have been treated for at least one cycle as noted in Section 4.1.1.

A maximum of 18 patients will be enrolled in Stage 1. Upon determination of the safety and tolerability of the treatment regimen, including determination of the evorpacept (ALX148) RD (and cetuximab RD if needed), the study will proceed to Stage 2. Only after the regimen has been determined to be safe and tolerable can the dose expansion phase be initiated (Stage 2).

Given the potential for late-onset toxicities, adverse event data will be collected for all patients in Stage 1 for 90 days after Cycle 1 Day 1. If additional DLT(s) are encountered during this extended monitoring period after the study has proceeded to Stage 2, then further enrollment in Stage 2 will be halted until discussion with the Investigators and Medical Monitor. If this occurs, the doses used in Stage 2 may be modified after discussion with the Investigators and Medical Monitor.

4.1.4 STAGE 2: DOSE EXPANSION

Forty-two patients will be enrolled and treated with the evorpacept (ALX148) RD, cetuximab (400 mg/m^2 day 1 of week 1 followed by 250 mg/m^2 weekly, unless a different RD is determined), and pembrolizumab (200 mg every 3 weeks). One cycle will be 21 days. There will be two cohorts – Cohort A and Cohort B. Enrollment will not occur by randomization and instead will occur based on Investigator and patient preference and slot availability.

If a significant portion of patients in Stage 2 experience AEs meeting DLT criteria as defined in Section 4.1.5, then dose reduction(s) of evorpacept (ALX148) and/or cetuximab for subsequent patients can be considered in discussion with the Investigators and Medical Monitor.

Patients in Cohort A will provide a fresh pretreatment biopsy sample and an on-treatment biopsy sample at C3D1 (±3 days). Prior to the pre-treatment biopsy, patients must sign the Informed Consent Form and all other described screening evaluations (Section 8.2.1 – "Screening Assessments to Determine Eligibility") must be completed and reviewed by the treating clinician to confirm that all eligibility criteria are met. While the acceptable window for the C3D1 biopsy is ±3 days, it is preferred for the biopsy to occur following all treatments on C3D1. The date and time of the biopsies will be documented. In Cohort A, archival tissue may not be used in place of a fresh pre-treatment biopsy. Patients in Cohort B will not undergo any study-related biopsies. When available, archival tissue will also be obtained for analysis for patients in both Cohort A and Cohort B. Other than biopsies, there are no differences between Cohort A and Cohort B (both receive the same treatment and study events). Details for handling of these samples including processing, storage, and shipment will be provided in the Laboratory Manual.

In rare circumstances, if there are no available spots in Cohort B and if a patient is motivated to participate and the Investigator judges that the risk associated with the biopsy is not appropriate for a research setting or the patient refuses biopsy, after consultation with the AGICC's medical monitor the patient may be allowed on study without a fresh biopsy. Similarly, for a subject in Cohort A, if the Investigator judges the risk for the pre- or on-treatment biopsy to be unacceptable in a research setting eg, based on complications during a previous procedure, use of blood thinning medications, or inaccessible tumor, or the patient refuses, a subject should not undergo the pre- or on-treatment biopsy and the patient will be allowed to enroll on or remain

on the study. If a patient initially assigned to Cohort A is unable to undergo both the pretreatment and on-treatment biopsies due to medical reasons or patient refusal, an additional patient may be enrolled in Cohort A (to achieve N=23 patients who underwent pretreatment and on-treatment biopsies).

Patients will undergo imaging of the chest, abdomen, and pelvis every 9 weeks for evaluation of response using RECIST v1.1 and iRECIST. Patients in both the safety run-in and dose expansion stages will continue to receive study therapy until disease progression according to RECIST v1.1 (Appendix 2), unacceptable toxicity, death, patient or physician decision to withdraw, or pregnancy, whichever occurs first. The maximum duration of pembrolizumab is 35 cycles. There is no maximum duration of evorpacept (ALX148) or cetuximab. Criteria for continuation of treatment beyond RECIST v1.1-defined progression is listed above in Section 4.1.2. The time from first patient enrollment until end of follow-up is expected to take approximately 48 months (24 months for enrollment/treatment and an additional 24 months for survival follow-up).

4.1.5 DOSE-LIMITING TOXICITIES

Adverse events (AEs), serious adverse events (SAEs), and clinically significant laboratory values that occur during the study and considered related to any of the study drugs will be regularly evaluated to determine whether continued dosing would potentially compromise the safety of future subjects. The severity of adverse events will be graded according to CTCAE version 5.0. The following constitute the definition of DLT in this study:

- Hematologic:
    - Grade 4 neutropenia lasting >7 days
    - Febrile neutropenia (defined as neutropenia ≥ Grade 3 and a single body temperature >38.3°C or a sustained temperature of ≥38°C for more than one hour)
    - Grade ≥3 neutropenia with infection
    - Grade 3 thrombocytopenia associated with clinically significant bleeding
    - Grade 4 thrombocytopenia
    - Grade ≥3 hemophagocytic lymphohistiocytosis
- Non-hematologic:
    - AST or ALT > 3x upper limit of normal (ULN) AND total bilirubin >2 x ULN, unless clear evidence of biliary obstruction exists
    - For patients with hepatic metastases, AST or ALT >8 x ULN or AST or ALT >5 x ULN for ≥14 days
    - Any Grade 2 pneumonitis that does not resolve to Grade ≤ 1 within 3 days of the initiation of maximal supportive care
    - Grade ≥3 toxicities, with the exception of the following:

        - Grade 3 nausea/vomiting or diarrhea <72 hours with adequate antiemetic and other supportive care
        - Grade 3 pain, rash, or constipation if duration is < 7 days
        - Grade 3 fatigue if duration is < 7 days
        - Grade ≥ 3 electrolyte abnormality that lasts < 72 hours, is not clinically complicated, and resolves spontaneously or responds to conventional medical interventions
        - Grade ≥ 3 amylase or lipase that is not associated with symptoms or clinical manifestations of pancreatitis
        - Grade ≥ 3 hypertension that recovers to Grade 2 within 5 days
    - Delay by more than 2 weeks in receiving the next scheduled dose due to persisting toxicities attributable to evorpacept (ALX148)
- In addition, clinically significant or persistent Grade 2 toxicities may be considered a DLT following review by AGICC and the Investigators.
    - A clinically significant laboratory value is one that indicates a new disease process, an exacerbation or worsening of an existing condition, or requires further action(s) to be taken

4.2 SCIENTIFIC RATIONALE FOR STUDY DESIGN

Stage 1: safety run-in

Cetuximab and pembrolizumab have been extensively assessed in prior clinical trials both as monotherapy and in combination with chemotherapy and/or other targeted agents. Avelumab (anti-PD-L1) and cetuximab are being evaluated in an ongoing phase II study in patients with previously treated mCRC and preliminary results suggest the combination is effective and well-tolerated.[68] Evorpacept (ALX148) has been previously evaluated in phase I clinical trials in other cancer types, both as monotherapy and in combination (including with pembrolizumab, where it has been found to be well-tolerated). We are unaware of any studies that have previously combined evorpacept (ALX148) (or any SIRPα/CD47 inhibitor), cetuximab (or any EGFR inhibitor), and pembrolizumab (or any anti-PD-(L)1 drug).

Stage 2: dose expansion

Additional patients will be enrolled and treated with evorpacept (ALX148), cetuximab, and pembrolizumab to better assess the efficacy and safety of this regimen. Additionally, a subset of patients (Cohort A) will undergo paired pre-treatment (after screening but before C1D1) and on-treatment (around C3D1) biopsies. This will allow for exploration of changes in tumor infiltrating immune cells (both innate and adaptive) and if this may be predictive or prognostic. The two cohorts (Cohort A and Cohort B) otherwise are identical and receive the same treatment.

Patients will not be randomized between the two cohorts and rather can enroll based on slot availability and Investigator/patient preference.

4.3 JUSTIFICATION FOR DOSE

Evorpacept (ALX148) dosing is based on prior phase I data in which no MTD was reached alone or in combination (maximum administered dose 15 mg/kg weekly with pembrolizumab and chemotherapy and 15 mg/kg weekly with rituximab and trastuzumab [53-55,67] Cetuximab and pembrolizumab dosing is based on standard dosing.

Specifically for pembrolizumab, the planned dose of pembrolizumab for this study is 200 mg every 3 weeks (Q3W). Based on the totality of data generated in the Keytruda development program, 200 mg Q3W is the appropriate dose of pembrolizumab for adults across all indications and regardless of tumor type. As outlined below, this dose is justified by:

- Clinical data from 8 randomized studies in melanoma and NSCLC indications demonstrating flat dose- and exposure-efficacy relationships from 2 mg/kg Q3W to 10 mg/kg Q2W, representing an approximate 5- to 7.5-fold exposure range (refer to IB, Section 5.2.2)
- Population PK analysis showing that both fixed dosing and weight-based dosing provides similar control of PK variability with considerable overlap in the distributions of exposures, supporting suitability of 200 mg Q3W
- Clinical data showing meaningful improvement in benefit-risk including overall survival at 200 mg Q3W across multiple indications, and
- Pharmacology data showing full target saturation in both systemic circulation (inferred from pharmacokinetic [PK] data) and tumor (inferred from physiologically-based PK [PBPK] analysis) at 200 mg Q3W

4.4 END OF STUDY DEFINITION

Patients in both the safety run-in and dose expansion stages will continue to receive study therapy until disease progression according to RECIST v1.1, unacceptable toxicity, death, patient or physician decision to withdraw, or pregnancy, whichever occurs first. Criteria in which patients in all cohorts may continue treatment after radiographic progression are detailed in Section 4.1.2. The end of study will be considered to have occurred when the 90-day phone call following the Final Study/Treatment Discontinuation Visit is complete. Overall survival information will be collected via telephone calls and/or clinic visits every 3 months ±14 days (first call and/or clinic visit to occur 3 months following the Final Study/Treatment Discontinuation Visit) until death, withdrawal of consent, the patient is lost to follow-up, study termination by AGICC, or a maximum of 2 years from the Final Study/Treatment Discontinuation Visit.

5 STUDY POPULATION

5.1 INCLUSION CRITERIA

To be eligible to participate in this study, an individual must meet all of the following criteria at screening (any assessments included in the Schedule of Events [Section 1.3] on Cycle 1 Day 1 must also continue to be met for the patient to remain eligible):

1. Provision to sign and date the consent form.
2. Able to comply with all study procedures and be available for the duration of the study in the Investigator's judgment.
3. Age ≥ 18 years on the day of signing informed consent
4. If in Cohort A, the patient must state willingness to undergo pre- and post-treatment biopsies. According to the Investigator's judgement, the planned biopsies should not expose the patient to substantially increased risk of complications.
5. Eastern Cooperative Oncology Group (ECOG) performance status of 0 or 1.
6. Histologically confirmed unresectable metastatic colorectal adenocarcinoma.
    - All primary tumor locations are allowed
    - Measurement of EGFR expression by immunohistochemistry is not required
7. Progression on at least two prior lines of therapy for unresectable metastatic colorectal adenocarcinoma.
    - A patient who progressed on a single line of therapy including a fluoropyrimidine, oxaliplatin, and irinotecan for unresectable metastatic colorectal adenocarcinoma (e.g., FOLFIRINOX or FOLFOXIRI) is eligible.
    - Previous administration of anti-EGFR drugs does not impact eligibility, except as listed in Exclusion Criterion #15.
8. Microsatellite stable or proficient mismatch repair status documented (only one of these criteria is needed, however if one criterion is met and one is not met then the patient is excluded)
9. Measurable disease, according to RECIST v1.1. Previously irradiated lesions are not considered measurable unless progression has been documented in the lesion. Note that lesions intended to be biopsied should not be target lesions.
10. Adequate hematologic and end organ function, defined by the following laboratory results:
    - ANC $\geq 1.5 \times 10^9$/L
    - Platelet count $\geq 100 \times 10^9$/L
    - Hemoglobin ≥ 9 g/dL without transfusion in the previous week
    - Serum bilirubin ≤ 1.5 x the upper limit of normal (ULN); patients with known Gilbert's disease may have a bilirubin ≤ 3.0 ×ULN

- AST, ALT, and alkaline phosphatase (ALP) ≤ 3 × ULN with the following exceptions:
    - Patients with documented liver metastases: AST and/or ALT ≤ 5 ×ULN
    - Patients with documented liver or bone metastases: ALP ≤ 5×ULN
- Creatinine clearance ≥ 50 mL/min as calculated using the Cockcroft-Gault formula or measured using a 24-hour urine collection
- International normalized ratio (INR) OR prothrombin time (PT) ≤1.5 × ULN unless participant is receiving anticoagulant therapy as long as INR or PT is within expected or therapeutic range of intended use of anticoagulants
- Activated partial thromboplastin time (aPTT) ≤1.5 × ULN unless participant is receiving anticoagulant therapy as long as aPTT is within expected or therapeutic range of intended use of anticoagulants

11. QTcF interval of ≤480 msec (Based upon value from the screening ECG).
12. Serum pregnancy test (for females of childbearing potential) negative at screening and at C1D1. A woman is considered fertile (woman of childbearing potential, "WOCBP") following menarche and until becoming post-menopausal unless permanently sterile. Women in the following categories are not considered WOCBP:
    - Premenarchal
    - Premenopauseal female with one of the following: documented hysterectomy, documented bilateral salpingectomy, documented bilateral oophorectomy (note: documentation can come from the site personnel's review of the participant's medical records, medical examination, or medical history interview)
    - Postmenopausal female. A postmenopausal state is defined as no menses for 12 months without an alternative medical cause. A high follicle stimulating hormone (FSH) level in the postmenopausal range may be used to confirm a postmenopausal state in women not using hormonal contraception or hormonal replacement therapy (HRT). However, in the absence of 12 months of amenorrhea, confirmation with two FSH measurements in the postmenopausal range is required. Females on HRT and whose menopausal status is in doubt will be required to use one of the non-hormonal highly effective contraception methods if they wish to continue their HRT during the study. Otherwise, they must discontinue HRT to allow confirmation of postmenopausal status before study enrollment.

    Female participants of childbearing potential are eligible to participate if they agree to correctly use one of the following forms of highly effective method of contraception with a failure rate of <1% per year when used consistently and correctly during the treatment period and for at least 180 days after the last study treatment. Periodic abstinence (e.g., calendar, ovulation, symptothermal, or postovulation methods) and

withdrawal are not acceptable methods of contraception. Use should be consistent with local regulations regarding the use of contraceptive methods for participants of clinical studies. If locally required, in accordance with Clinical Trial Facilitation Group (CTFG) guidelines, acceptable hormonal contraceptives are limited to those which inhibit ovulation.

- Progestogen- only contraceptive implant
- Intrauterine hormone-releasing system
- Intrauterine device (IUD)
- Bilateral tubal occlusion
- Vasectomized partner. A vasectomized partner is a highly effective contraception method provided that the partner is the sole male sexual partner of the WOCBP and the absence of sperm has been confirmed. If not, an additional highly effective method of contraception should be used.
- Sexual abstinence. Sexual abstinence is considered a highly effective method only if defined as refraining from heterosexual intercourse during the entire period of risk associated with the study treatment. The reliability of sexual abstinence needs to be evaluated in relation to the duration of the study and the preferred and usual lifestyle of the participant.
- Combined (estrogen- and progestogen- containing) hormonal contraception, including oral, intravaginal, transdermal, or injectable
- Progestogen-only hormonal contraception, including oral or injectable

13. For men: Male participants with female partners of childbearing potential are eligible to participate if they agree to one of the following during the treatment period and for at least 180 days after the last dose of study treatment as defined below. Periodic abstinence (e.g., calendar, ovulation, symptothermal, or postovulation methods) and withdrawal are not acceptable methods of contraception. Men must also agree to refrain from donating sperm following during the treatment period and for at least 180 days after the last dose of study treatment.
    - Be abstinent from penile-vaginal intercourse as their usual and preferred lifestyle (abstinent on a long term and persistent basis) and agree to remain abstinent
    - Use a male condom plus partner use of a contraceptive method with a failure rate of <1% per year as described in Inclusion Criteria #12 when having penile-vaginal intercourse with a woman of childbearing potential who is not currently pregnant.
        - Note: Men with a pregnant or breastfeeding partner must agree to remain abstinent from penile-vaginal intercourse or use a male condom during each episode of penile penetration.

5.2 EXCLUSION CRITERIA

An individual who meets any of the following criteria will be excluded from participation in this study:

Cancer-related exclusion criteria:

1. Patients with known MSI-high status or known mismatch repair deficiency (dMMR)
2. Patients in whom both mismatch repair and microsatellite stability status are unknown
3. Has known active CNS metastases and/or carcinomatous meningitis. Participants with previously treated brain metastases may participate provided they are radiologically stable, i.e. without evidence of progression for at least 4 weeks by repeat imaging (note that the repeat imaging should be performed during study screening), clinically stable and without requirement of steroid treatment for at least 14 days prior to Cycle 1 Day 1.
4. Systemic anti-cancer therapy within 4 weeks of starting study treatment (6 weeks for mitomycin C or nitrosureas). If systemic anti-cancer therapy was given within 4 weeks, patient may be included if 5 times the elimination half-life of the drug has passed.
5. Malignancies other than CRC within 3 years prior to Cycle 1 Day 1 with the exception of those with a negligible risk of metastasis or death (e.g., expected 5-year overall survival > 90%) treated with expected curative outcome (such as adequately treated carcinoma in situ of the cervix, basal or squamous cell skin cancer, localized prostate cancer treated surgically with curative intent, and ductal carcinoma in situ treated surgically with curative intent).
6. Prior radiation therapy within 14 days prior to study Cycle 1 Day 1 and/or persistence of radiation-related adverse effects. Participants must have recovered from all radiation-related toxicities, not require corticosteroids, and not have had radiation pneumonitis. A 1-week washout is permitted for palliative radiation (≤2 weeks of radiotherapy) to non-CNS disease. However, palliative radiation therapy (as long as it does not involve target lesions) is permitted on the study.
7. Prior allogeneic bone marrow transplantation or solid organ transplant for another malignancy in the past.
8. Spinal cord compression not definitively treated with surgery and/or radiation.
9. Uncontrolled pleural effusion, pericardial effusion, or ascites requiring recurrent drainage procedures.
10. Uncontrolled tumor related pain. Patients who require narcotic pain medication during screening should be on a stable dose regimen for seven days prior to Cycle 1 Day 1.

Exclusion criteria related to study medication:

11. History of severe allergic, anaphylactic, or other hypersensitivity reactions to any of the study medications or their classes
12. History of red meat allergy or history of tick bite (these may increase the risk of a cetuximab infusion reaction).

13. Prior therapy with an anti-PD-1, anti-PD-L1, or anti-PD-L2 agent or with an agent directed to another stimulatory or co-inhibitory T-cell receptor (eg, CTLA-4, OX-40, CD137).
14. Prior treatment with any anti-CD47 or anti-SIRPα drugs
15. Left-sided (at or distal to the splenic flexure) RAS/BRAF WT mCRC who are EGFR inhibitor naïve.
16. Has a diagnosis of immunodeficiency or is receiving systemic steroid therapy or any other form of immunosuppressive therapy within 7 days prior to Cycle 1 Day 1.
17. History of hemolytic transfusion reaction.
18. History of non-infectious pneumonitis/interstitial lung disease that required steroids or has current pneumonitis/interstitial lung disease.
19. Has an autoimmune disease that has required systemic treatment in the past 2 years with use of disease modifying agents, corticosteroids, or immunosuppressive drugs. Replacement therapy (eg; thyroxine, insulin, physiologic corticosteroid replacement therapy for adrenal or pituitary insufficiency) is not considered a form of systemic treatment.
20. History of autoimmune hemolytic anemia or autoimmune thrombocytopenia

<u>Exclusion criteria based on organ function or medical history</u>:

21. Any major surgery within 28 days prior to enrollment (does not include pre-treatment biopsy).
22. The patient has clinically relevant coronary artery disease or history of myocardial infarction in the last 12 months or high risk of uncontrolled arrhythmia or uncontrolled cardiac insufficiency.
23. The patient has uncontrolled or poorly-controlled hypertension (>180 mmHg systolic or > 130 mmHg diastolic).
24. Life expectancy of < 12 weeks.
25. Has a history or current evidence of any condition, therapy, or laboratory abnormality that might confound the results of the study, interfere with the participant's participation for the full duration of the study, or is not in the best interest of the participant to participate, in the opinion of the treating investigator.
26. Has known psychiatric or substance abuse disorders that would interfere with cooperation with the requirements of the trial.
27. Pregnant or lactating or intending to become pregnant during the study.
28. AEs due to prior cancer therapies that have not returned to ≤Grade 1 or baseline. Participants with endocrine-related AEs Grade ≤2 that are now controlled with treatment/hormonal therapy are eligible.

<u>Exclusion criteria based on infectious diseases</u>:

29. Active infection requiring IV antibiotics at screening.

30. Patients with active hepatitis B (chronic or acute). Active hepatitis B infection is defined as having a positive hepatitis B surface antigen [HBsAg] test at screening. Patients with a cleared hepatitis B infection (as defined by the presence of hepatitis B core antibody [anti-HBc], absence of HBsAg, and negative HBV DNA) are eligible.
31. Patients with active hepatitis C. Patients positive for hepatitis C virus (HCV) antibody are eligible only if polymerase chain reaction (PCR) is negative for HCV RNA.
32. Known HIV infection.
33. Recent COVID-19 diagnosis (symptomatic or asymptomatic). To become eligible (following symptomatic infection), the patient must not have fever for 24 hours (without using medicine to reduce fever), other symptoms have improved, and at least 10 days have passed since onset of symptoms. To become eligible (following asymptomatic infection, ie positive test only), at least 10 days have passed since the positive test. In either case, a repeat COVID-19 test is not required. Likewise, a persistently positive test (if obtained) does not continue to exclude the patient should the other criteria be satisfied.
34. Influenza vaccination should be given during influenza season. Patients must not receive live, attenuated influenza vaccine (e.g., FluMist®) within 30 days prior to Cycle 1 Day 1 or at any time during the study and for at least 5 months after the last dose of study drug.

5.3 LIFESTYLE CONSIDERATIONS

There are no lifestyle considerations other than those pertaining to abstinence/contraception as outlined in section 5.1 (Inclusion Criteria).

5.4 SCREEN FAILURES

If in the determination of the PI there is reasonable chance that a patient may meet inclusion/exclusion criteria at a time following a screen failure, a patient may be re-screened for eligibility with documented approval from the medical monitor.

5.5 STRATEGIES FOR RECRUITMENT AND RETENTION

Potential subjects will be identified and recruited from the research study team's clinic. The consent process will be conducted in a private room. Potential subjects will be given a copy of the consent form to take home with them before making a final decision to participate. Advertisements approved by ethics committees and Investigator databases may be used as recruitment procedures. Patients will be recruited without regard to sex, race, or ethnicity.

In order to achieve a total sample size of 48 patients (including both Stage 1 and Stage 2), it is estimated that 56 patients will need to be screened (i.e., allowing for 4 patients who fail screening and 4 patients who do not complete the study). There will be approximately 5 sites (all

in the United States) involved, and it is estimated that the enrollment rate will be 1 patient/site/month. It will take approximately 12 months to fully enroll the study. The total study duration is anticipated to be approximately 24 months (enrollment/treatment) and 48 months (including survival follow-up).

6 STUDY INTERVENTION

6.1 STUDY INTERVENTION(S) ADMINISTRATION

6.1.1 STUDY INTERVENTION DESCRIPTION

The study agents for this study are evorpacept (ALX148), cetuximab, and pembrolizumab.

Evorpacept (ALX148), cetuximab, and pembrolizumab packaging will be overseen by ALX Oncology Ltd, Eli Lilly USA, LLC, and Merck & Co, Inc. clinical trial supplies department, respectively, and will bear labels in compliance with US Federal law. The packaging and labeling of the study drugs will be in accordance with the manufacturer's standards and Federal regulations.

Upon delivery of the investigational products to the site, site personnel should check for damage and verify proper identity, quality, integrity of seals and temperature conditions. Site personnel should report any deviations or product complaints to the AGICC and/or ALX Oncology Ltd, Merck & Co, Inc., and Eli Lilly USA, LLC (as appropriate) upon discovery.

Evorpacept (ALX148), cetuximab, and pembrolizumab will be stored at the clinical site under the required storage conditions as indicated on the study drug labels.

6.1.2 DOSING AND ADMINISTRATION

Evorpacept (ALX148), cetuximab, and pembrolizumab will all be given on an outpatient basis.

On administration days when dosing schedules coincide, evorpacept (ALX148) will be given first, followed by a 30-minute delay. Then, cetuximab and/or pembrolizumab should be given in an order determined by institutional standards.

Patients will be observed in the clinic for at least 2 hours after infusion of evorpacept (ALX148) on C1D1 and as clinically indicated thereafter.

Dose levels are provided in Section 12.1.2.

The maximum duration of pembrolizumab is 35 cycles. There is no maximum duration of evorpacept (ALX148) or cetuximab.

Dose, administration, and premedication

Evorpacept (ALX148) (per the Investigator's Brochure)

- Dose: 15 mg/kg day 1, 8, and 15 every 21 days
- Administration: Administer infusion solution intravenously over 60 minutes ± 10 minutes. The use of an infusion pump is the preferred method of administration to ensure accurate delivery of the investigational product, but gravity drips are allowed.
- Pre-medication: none required
- Route of administration: intravenous
- Starting dose and dose escalation: not applicable
- Dose adjustments/modifications/delays: see Appendix 1
- Duration of therapy: Patients in both the safety run-in and dose expansion stages will continue to receive study therapy until disease progression according to RECIST v1.1, unacceptable toxicity, death, patient or physician decision to withdraw, or pregnancy, whichever occurs first. Criteria in which patients in all cohorts may continue treatment after radiographic progression are detailed in Section 4.1.2.
- Tracking of dose: not applicable

Cetuximab (per the package insert)

- Dose: 400 mg/m^2 cycle 1 day 1 (loading dose), 250 mg/m^2 cycle 1 day 8 & 15, followed by 250 mg/m^2 day 1, 8, and 15 on cycle 2 and subsequent cycles
- Administration: Administer via IV infusion (infusion pump or syringe pump); loading dose over 2 hours, weekly maintenance dose over 1 hour. Do not administer as IV push or bolus. Do not exceed an infusion rate of 10 mg/min. Do not shake or dilute. Administer via infusion pump or syringe pump. Following the infusion, an observation period (1 hour) is recommended; longer observation time (following an infusion reaction) may be required. The maximum infusion rate is 10 mg/minute. Administer through a low protein-binding 0.22 micrometer in-line filter.
- Pre-medication: Premedicate with an H1 antagonist (eg, diphenhydramine) IV 30 to 60 minutes prior to the first dose; premedication for subsequent doses may be necessary based on clinical judgment.
- Route of administration: intravenous
- Starting dose and dose escalation: not applicable
- Dose adjustments/modifications/delays: see Appendix 1

- Duration of therapy: Patients in both the safety run-in and dose expansion stages will continue to receive study therapy until disease progression according to RECIST v1.1, unacceptable toxicity, death, patient or physician decision to withdraw, or pregnancy, whichever occurs first. Criteria in which patients in all cohorts may continue treatment after radiographic progression are detailed in Section 4.1.2.
- Tracking of dose: not applicable

Pembrolizumab (per the package insert)

- Dose: 200 mg IV day 1 every 21 days
- Administration: Pembrolizumab 200 mg will be administered as a 30 minute IV infusion every 3 weeks. Sites should make every effort to target infusion timing to be as close to 30 minutes as possible. However, given the variability of infusion pumps from site to site, a window of -5 minutes and +10 minutes is permitted (i.e., infusion time is 30 minutes: -5 min/+10 min). The Pharmacy Manual contains specific instructions for the preparation of the pembrolizumab infusion fluid and administration of infusion solution.
- Pre-medication: none required
- Route of administration: intravenous
- Starting dose and dose escalation: not applicable
- Dose adjustments/modifications/delays: see Appendix 1
- Duration of therapy: Patients in both the safety run-in and dose expansion stages will continue to receive study therapy until disease progression according to RECIST v1.1, unacceptable toxicity, death, patient or physician decision to withdraw, or pregnancy, whichever occurs first. Criteria in which patients in all cohorts may continue treatment after radiographic progression are detailed in Section 4.1.2.
- Tracking of dose: not applicable

Dose modifications, interruptions, and delays

Every effort should be made to administer study products on the planned dose and schedule. In the event of significant toxicity dosing may be delayed and/or reduced as described in Appendix I. In the event of multiple toxicities, dose modification should be based on the worst toxicity observed. Patients are to be instructed to notify Investigators at the first occurrence of any adverse symptom.

Medication Errors

Medication errors may occur in this study, from the administration of the wrong drug, to the wrong patient, at the wrong time, or at the wrong dosage strength. Such medication errors occurring to a study participant are to be captured on the medication error electronic case report form (eCRF) which is a specific version of the adverse event (AE) page, and on the SAE form

when appropriate. In the event of medication dosing error, AGICC and medical monitor should be notified immediately.

Medication errors are reportable irrespective of the presence of an associated AE/SAE, including;

- Medication errors involving patient exposure to evorpacept (ALX148), cetuximab, and/or pembrolizumab
- Potential medication errors or uses outside of what is foreseen in the protocol that do or do not involve the participating subject.

Whether or not the medication error is accompanied by an AE, as determined by the Investigator, the medication error should be captured on the medication error version of the adverse event (AE) page and, if applicable, any associated adverse event(s) is captured on an adverse event (AE) eCRF page.

6.2 STUDY DRUG PREPARATION/HANDLING/STORAGE/ACCOUNTABILITY

6.2.1 ACQUISITION AND ACCOUNTABILITY

Evorpacept (ALX148): ALX Oncology Ltd will provide evorpacept (ALX148) from investigational supply

Cetuximab: Eli Lilly USA, LLC will provide cetuximab from commercial supply

Pembrolizumab: Merck & Co, Inc. will provide pembrolizumab from commercial supply

6.2.2 FORMULATION, APPEARANCE, PACKAGING, AND LABELING

Evorpacept (ALX148)

The evorpacept (ALX148) Injection, 20 mg/mL, is packaged in 20 mL (400 mg) and 50 mL (1000 mg) USP Type I clear glass vials with a 20 mm Teflon coated rubber serum stopper and tamper-evident aluminum seal. The formulation is composed of evorpacept (ALX148) at 20 mg/mL, in 20 mM citrate, 150 mM NaCl, 0.02% (w/v) Polysorbate 80, pH 6.0. Each 20 mL vial (20 mg/mL) contains 400 mg of evorpacept (ALX148). Each 50 mL vial (20 mg/mL) contains 1000 mg of evorpacept (ALX148).

Cetuximab

Cetuximab injection is a sterile, preservative-free, clear, colorless solution in a 2 mg/mL single-dose vial supplied as follows:

- 100 mg/50 mL individually packaged in a carton (NDC 66733-948-23)

- 200 mg/100 mL individually packaged in a carton (NDC 66733-958-23)

Pembrolizumab

- Pembrolizumab (MK-3475) Solution for Infusion is a sterile, non-pyrogenic aqueous solution supplied in single-use Type I glass vial containing 100 mg/4 mL of pembrolizumab (MK-3475). The product is preservative-free, latex free solution which is essentially free of extraneous particulates.
- Pembrolizumab (MK-3475) Solution for Infusion vials are filled to a target of 4.25mL (106.25mg) to ensure recovery of 4.0mL (100mg).

6.2.3 PRODUCT STORAGE AND STABILITY

Evorpacept (ALX148)
Specific storage and stability instructions for evorpacept (ALX148) are provided in the Pharmacy Manual.

Cetuximab

- Store vials under refrigeration at 2° C to 8° C (36° F to 46° F).
- Do not freeze or shake.
- Increased particulate formation may occur at temperatures at or below 0° C (32° F).
- Discard any remaining solution in the infusion container after 8 hours at controlled room temperature or after 12 hours at 2° C to 8° C.
- Discard any unused portion of the vial.

Pembrolizumab
Pembrolizumab (MK-3475) Solution for Infusion, 100 mg/ 4 mL vial: pembrolizumab (MK-3475) Solution for Infusion vials should be stored at refrigerated conditions 2 – 8 °C (36 - 46 °F) and protected from light. Do not shake and do not freeze. Vials should be stored in the original box to ensure the drug product is protected from light.

6.2.4 PREPARATION

Evorpacept (ALX148)
Specific preparation and dispensing instructions for evorpacept (ALX148) are provided in the Pharmacy Manual.

Cetuximab
Specific preparation and dispensing instructions for cetuximab are provided in the Pharmacy Manual.

Pembrolizumab
Specific preparation and dispensing instructions for pembrolizumab are provided in the Pharmacy Manual.

6.3 MEASURES TO MINIMIZE BIAS: RANDOMIZATION AND BLINDING

There is no randomization or blinding in this study.

6.4 STUDY INTERVENTION COMPLIANCE

All study drugs are given intravenously, therefore a drug diary is not needed. All patients will be given a study calendar detailing study activities.

Subject retention and compliance with the protocol will be reported using:

- A CONSORT diagram illustrating the subject flow through the trial
- Tables summarizing retention and compliance
- Listings showing retention and compliance results at the individual subject level

6.5 PROHIBITED THERAPY

Any concomitant therapy intended for the treatment of cancer, whether health authority approved or experimental, is prohibited for various time periods prior to starting study treatment (depending on the anti-cancer agent, see Section 5.2), and during study treatment until disease progression is documented and the patient has discontinued study treatment. This includes but is not limited to chemotherapy, hormonal therapy, immunotherapy, radiotherapy (see exception below), investigational agents, or herbal therapy.

The following medications are prohibited while receiving study treatment, unless otherwise noted:

- Traditional herbal medicines, as their use may result in unanticipated drug-drug interactions that may cause or confound assessment of toxicity. The concurrent use of vitamins is permitted.
- Any live, attenuated vaccine (e.g., FluMist) within 30 days prior to Cycle 1 Day 1 or at any time during the study and for at least 5 months after the last dose of study drug.
- Use of steroids to premedicate patients for whom CT scans with contrast are contraindicated (i.e., patients with contrast allergy or impaired renal clearance). Such patients are allowed on study and MRIs of the chest, abdomen, and pelvis with a non-

contrast CT scan of the anatomical region of interest will be performed for tumor assessment.

- Immunomodulatory agents, including but not limited to interferons or interleukin-2, during the entire study; these agents could potentially increase the risk for autoimmune conditions.
- Immunosuppressive medications, including but not limited to cyclophosphamide, azathioprine, methotrexate, and thalidomide; these agents could potentially alter the activity and the safety of immunotherapy.
- Systemic corticosteroids and tumor necrosis factor-α (TNF-α) inhibitors may attenuate potential beneficial immunologic effects of treatment. Therefore, in situations where systemic corticosteroids or TNF-α inhibitors would be routinely administered, alternatives, including antihistamines, should be considered first by the treating physician.
- Systemic glucocorticoids for any purpose other than to modulate symptoms from an event of clinical interest of suspected immunologic etiology. The use of physiologic doses of corticosteroids may be approved after consultation with the Sponsor.
- Acute emergency administration, topical applications, inhaled sprays, eye drops, or local injections of corticosteroids are allowed. As noted in Section 5.2 (Exclusion Criteria), the use of systemic steroid therapy or any other form of immunosuppressive therapy is an exclusion criterion for study entry.
- Hematopoietic growth factors should not be administered prophylactically before initial treatment with study drugs. Hematopoietic growth factors and transfusions may be administered according to local guidelines if indicated during the study.

The above lists of medications are not necessarily comprehensive. Thus, the investigator should consult the prescribing information for any concomitant medication as well as online drug interaction resources when determining whether a certain medication is metabolized by or strongly inhibits or induces CYP. In addition, the investigator should contact the Medical Monitor if questions arise regarding medications not listed above.

There are no prohibited therapies during the Post-Treatment Follow-up Phase.

Surgery

Caution is advised on theoretical grounds for any surgical procedures during the study. The appropriate interval of time between surgery and evorpacept (ALX148) required to minimize the risk of impaired wound healing and bleeding has not been determined. Stopping evorpacept (ALX148) is recommended at least 7 days prior to surgery. Postoperatively, the decision to reinitiate evorpacept (ALX148) treatment should be based on a clinical assessment of

satisfactory wound healing and recovery from surgery. The Investigator should refer to the US or local Package inserts for instructions on stopping/re-initiation of cetuximab and pembrolizumab.

6.6 CONCOMITANT THERAPY

All concomitant medications taken during study participation will be recorded continuously on the electronic case report forms (eCRFs), starting at the screening visit and ending 30 days from treatment discontinuation. For this protocol, a prescription medication is defined as a medication that can be prescribed only by a properly authorized/licensed clinician. Medications reported in the eCRF are concomitant prescription medications, over-the-counter medications, and non-prescription medications. Concomitant treatment considered necessary for the patient's well-being may be given at the discretion of the treating physician.

Examples of permitted concomitant therapies include but are not limited to:

- Oral contraceptives.
- Hormone-replacement therapy.
- Prophylactic or therapeutic anticoagulation therapy (such as low-molecular weight heparin or warfarin at a stable dose level).
- Inactive influenza vaccinations during influenza season.
- Megestrol administered as an appetite stimulant.
- Inhaled corticosteroids for chronic obstructive pulmonary disease.
- Mineralocorticoids (e.g., fludrocortisone).

Radiation

Palliative radiotherapy on study is permitted for the treatment of painful bony lesions providing the lesions were known at the time of study entry and the Investigator clearly indicates that the need for palliative radiotherapy is not indicative of disease progression. Study treatment should be interrupted during palliative radiotherapy–stopping 7 days before and resuming treatment 7 days after. Irradiated lesions will be considered not evaluable for response but still can be used to assess disease progression. The intensities, number, and dates of doses received for allowed palliative radiotherapy should be recorded on the appropriate eCRFs.

6.6.1 RESCUE MEDICINE

Anti-emetics and anti-diarrheal medications should not be administered prophylactically before initial treatment with study drugs. At the discretion of the investigator, prophylactic anti-emetic and anti-diarrheal medication(s) may be used per standard clinical practice before subsequent doses of study drugs.

Hematopoietic growth factors should not be administered prophylactically before initial treatment with study drugs. Hematopoietic growth factors and transfusions may be administered according to local guidelines if indicated during the study.

In general, investigators should manage a patient's care with supportive therapies as clinically indicated, as per local standards. Patients who experience infusion-associated symptoms may be treated symptomatically with acetaminophen, ibuprofen, diphenhydramine, and/or famotidine or another H2 receptor antagonist as per standard practice. Serious infusion−associated events manifested by dyspnea, hypotension, wheezing, bronchospasm, tachycardia, reduced oxygen saturation, or respiratory distress should be managed with supportive therapies as clinically indicated (e.g., supplemental oxygen and β2-adrenergic agonists).

Guidelines for known toxicities of study agents are provided in the Appendix 1. All medications must be recorded on the Concomitant Medications eCRF.

7 STUDY INTERVENTION DISCONTINUATION AND PARTICIPANT DISCONTINUATION/WITHDRAWAL

7.1 DISCONTINUATION OF STUDY INTERVENTION (STOPPING RULES)

AGICC has the right to terminate the study intervention at any time. Reasons for terminating the study intervention may include, but are not limited to, the following:

- The incidence or severity of adverse events in this or other studies indicates a potential health hazard to patients
- Patient enrollment is unsatisfactory
- Poor protocol adherence
- Inaccurate or incomplete data recording
- Non-compliance with the International Conference on Harmonization (ICH) guideline for Good Clinical Practice
- No study activity (i.e., all patients have completed the study and all obligations have been fulfilled)

AGICC will notify ALX Oncology Ltd, Merck & Co, Inc., & Eli Lilly USA, LLC if they decide to discontinue the study intervention.

Discontinuation from study intervention does not mean discontinuation from the study, and remaining study procedures should be completed as indicated by the study protocol and Schedule of Events (Section 1.3). If a clinically significant finding is identified (including, but not limited to changes from baseline) after enrollment, the investigator or qualified designee will determine

if any change in subject management is needed. Any new clinically relevant finding will be reported as an adverse event (AE).

7.2 PARTICIPANT DISCONTINUATION/WITHDRAWAL FROM STUDY

Participants are free to withdraw from participation in the study at any time upon request.

In addition, the investigator has the right to withdraw a patient from the study at any time. Reasons for withdrawal from the study may include, but are not limited to, the following:

- Patient withdrawal of consent at any time
- Any medical condition that AGICC or the medical monitor determines may jeopardize the patient's safety if he or she continues in the study
- AGICC or the medical monitor determines it is in the best interest of the patient
- Patient non-compliance

Patients must discontinue study treatment if they experience any of the following:

- Disease progression (Patients are allowed to receive study treatment beyond RECIST v1.1-defined-disease progression if certain conditions are met (see Section 4.1.2)).
- Symptomatic deterioration attributed to disease progression as determined by the investigator after integrated assessment of radiographic data, biopsy results, and clinical status
- Intolerable toxicity related to any study drug (see section 12.1 for additional guidance)
- Any medical condition that may jeopardize the patient's safety if he or she continues study treatment
- Use of another non−protocol anti-cancer therapy
- Pregnancy

Every effort should be made to obtain information on patients who withdraw from the study. The primary reason for withdrawal from the study or study drug discontinuation should be documented on the appropriate eCRF. However, patients will not be followed for any reason after consent has been withdrawn. Patients enrolled in Stage 1 (safety run-in) who withdraw consent before completing the first cycle for reasons other than adverse events will be replaced. Patients who withdraw from Stage 2 of the study will not be replaced.

7.3 LOST TO FOLLOW-UP

A subject will be considered lost to follow-up if he or she fails to return for 3 scheduled visits and is unable to be contacted by the study staff.

The following actions must be taken if a subject fails to return to the clinic for a required study visit:

- Site will attempt to contact the subject and reschedule the missed visit and advise subject on importance of maintaining assigned visit schedule
- Before a subject is deemed lost to follow-up, the investigator will make every effort to regain contact with the subject. These contact attempts should be documented in the subject's medical record or study file
- Should the subject continue to be unreachable, he or she will be considered to have withdrawn from the study

8 STUDY ASSESSMENTS AND PROCEDURES

Please see Section 1.3 (Schedule of Events) for the schedule of events to be performed in this study.

8.1 EFFICACY ASSESSMENTS

Baseline tumor assessments should be performed ≤ 28 days before Cycle 1 Day 1 and assessed according to RECIST v1.1 and iRECIST (see Appendix 2). The same procedure used to assess disease sites at baseline should be used throughout the study (e.g., the same contrast protocol for CT scans or MRI scans). CT or MRI scans should include chest, abdomen, and pelvic scans; and other areas as clinically indicated. At the investigator's discretion, imaging may be repeated at any time if progressive disease is suspected. Evaluation of tumor response conforming to RECIST v1.1 and iRECIST must be documented every 9 weeks ± 5 days (no matter where the patient is in the treatment cycle). Patients in both the safety run-in and dose expansion stages will continue to receive study therapy until disease progression according to RECIST v1.1 (Appendix 2), unacceptable toxicity, death, patient or physician decision to withdraw, or pregnancy, whichever occurs first.

In all cohorts, at the time of RECIST v1.1-defined progression (i.e. iUPD), treatment is permitted to continue until the next assessment (at least 4 weeks but no longer than 8 weeks later) if the following criteria are met:

- Patient is clinically stable (An assignment of clinical stability requires that no worsening of performance status has occurred, that no clinically relevant increases in disease-related symptoms such as pain or dyspnea occur that are thought to be associated with disease progression (these symptoms are generally understood to mean a requirement for increased palliative intervention), and that no requirement for intensified management of disease-related symptoms exists, including increased

analgesia, radiotherapy, or other palliative care.)

- Absence of tumor progression at critical anatomical sites (e.g., leptomeningeal disease) that cannot be managed by protocol-allowed medical interventions
- Patient and investigator agree to continue treatment
- Patient signs the Treatment Beyond Progression Informed Consent Form
- If the subsequent scan continues to show progression, all therapy will be discontinued and the patient will be taken off study.

If iUPD is noted, a follow-up scan should be obtained at least 4 weeks but no longer than 8 weeks later. Schedule of tumor assessments are independent of any changes to the study treatment administration schedule (e.g., dose delay) and may occur mid-cycle depending on length of cycle. If a tumor assessment must be performed early or late, subsequent assessments should be conducted according to the original schedule based on the date of first study drug administration (Cycle 1, Day 1). Confirmation of response (PR or complete response [CR]) will be done no earlier than 28 days from study entry. In the case of SD, measurements must have met the SD criteria at least once after study entry at a minimum interval not less than 6 weeks. Patients who discontinue study treatment for any reason other than disease progression will continue to undergo tumor response evaluations (approximately every 9 weeks) until progressive disease. Rising tumor markers (e.g., CEA) in the absence of radiological evidence of progression is not considered progressive disease.

8.2 SAFETY AND OTHER ASSESSMENTS

8.2.1 SCREENING

Screening is to be done within 28 days prior to study entry (i.e. prior to Cycle 1 Day 1 (C1D1)). Written informed consent must be obtained prior to undergoing study specific procedures.

The following assessments will be conducted to determine eligibility. All of these investigations must be completed, reviewed, and recorded as part of the screening process. The investigator will maintain a screening log to record details of all patients screened irrespective of eligibility. Site staff will collect the blood samples for these standard blood tests. Any pre-existing baseline abnormality must be graded and recorded as per NCI-CTCAE v5.0.

Screening Assessments to Determine Eligibility

Tumor history will be recorded: History of CRC should include stage, date of first diagnosis, site of primary disease, previous biopsies particularly date and site of biopsy, location of metastases, previous treatments administered including all surgeries and radiation therapy, previous clinical trials especially details of any previous use of immunotherapies,

ECOG performance status, MMR/MSI status, tumor mutational burden (TMB), and mutational testing particularly KRAS, NRAS, and BRAF.

Demographic/Medical History. Demographic data and medical history will be recorded: Includes age, sex, self-reported ethnicity, detailed description of history of non-CRC cancer, past medical and surgical history, list of medications including prescription, OTC, herbal, homeopathic, nutritional drugs, performance status, social history including alcohol, tobacco and recreational drug use, family history especially of malignancy and reproductive history.

Physical Exam/Vitals. A complete physical exam, height, weight, and vital signs (blood pressure (BP), heart rate (HR), and temperature to be recorded in the sitting position) will be conducted at screening.

Baseline signs and symptoms will be recorded: patients will be asked about any signs and symptoms experienced within the 14 days prior to study entry. Baseline signs and symptoms will be recorded on the eCRF.

Performance status will be recorded: use Eastern Cooperative Oncology Group (ECOG) performance status criteria (Appendix 3)

12-lead ECG: A single ECG will be collected on at least a 10 second strip at screening and after infusion of evorpacept (ALX148) on C1D1, C1D8, and C3D1. No further study-mandated ECGs are needed after C3D1, except an ECG will be done at the Final study / treatment discontinuation visit. If the QTcF is prolonged (value of >500 msec), the ECG should be re-evaluated by a qualified person at the institution for confirmation. Additional ECGs should be performed as clinically indicated.

Hematology: Complete blood count (CBC) to include hemoglobin, platelets, WBC, absolute neutrophils, lymphocytes, monocytes, eosinophils, and basophils. No need to repeat on C1D1 if screening assessment performed within 72 hours prior to that date. Additional testing as clinically needed. Blood type and a RBC antibody screen also known as a 'Type and Screen' are to be collected at screening.

Because evorpacept (ALX148) binds to CD47 expressed on red blood cells (RBC), the presence of evorpacept (ALX148), which contains a modified IgG1 Fc domain, in patient whole blood and plasma can interfere with cross-matching assays performed by blood banks in order to identify compatible or least incompatible units of RBC for transfusion. Both the direct antibody test (DAT) and the indirect antibody test (IAT) may appear to be positive for reactive antibodies (IgG) in samples from patients who are on treatment with evorpacept (ALX148), due to the binding of AHG reagent to the Fc portion of evorpacept (ALX148). This phenomenon also occurs in patients receiving any Fc containing antibody-based targeted therapy that is directed against antigens present on RBC, such as the anti-CD38 antibodies used for treatment of multiple myeloma, as well as other anti-CD47 directed therapeutic antibodies.[69,70] ALX148 does not interfere with binding to C3,

and the DAT C3 test is interpretable. ABO blood type and baseline allo-antibodies (the latter in patients with a history of prior RBC transfusion) can both be identified by performing a blood type and antibody screen at baseline prior to the administration of CD47-directed therapy. Additionally, genotyping of the patient's common and minor blood group antigens is suggested at baseline to provide guidance on which allo-antibodies they may develop following RBC transfusion. Additional ABO Rh blood typing and cross-matching testing may be performed on study as required by local regulations, but the results of these on-treatment tests may not be interpretable. Patients' complete blood counts should be monitored, and if unanticipated anemia develops, additional hemolysis laboratory assessments (e.g., CBC, reticulocyte count, peripheral blood smear, LDH, serum haptoglobin, indirect bilirubin, urinalysis and others, as indicated; see Section 12.1.3) should be considered. If a blood transfusion is needed during study, the type and screen (and baseline genotyping or extended phenotyping of minor red cell antigens, if obtained) obtained during study screening should be used to guide these transfusions, and the blood bank should be made aware of these considerations and the presence of evorpacept (ALX148).

Blood Chemistry: Should include sodium, potassium, chloride, bicarbonate or carbon dioxide, BUN (or urea), creatinine, non-fasting glucose, calcium, **magnesium**, **phosphorus**, albumin, total protein, AST/SGOT, ALT/SGPT, alkaline phosphatase, total, **lipase**, and **amylase**. No need to repeat on C1D1 if screening assessment performed within 72 hours prior to that date. Additional testing as clinically needed.

Coagulation: Should include International Normalized Ratio (INR) and Partial Thromboplastin Time (PTT). No need to repeat on C1D1 if screening assessment performed within 72 hours prior to that date.

Urinalysis: Dipstick is acceptable. Microscopic analyses if dipstick abnormal. No need to repeat on C1D1 if screening assessment performed within 72 hours prior to that date. If ≥2+ protein on urine dipstick, then collect spot urine sample to calculate urine protein to creatinine ratio (UPCR).

Serum Pregnancy Test: For female patients of childbearing potential, a serum pregnancy test, with sensitivity of at least 25 mIU/mL, and assayed in a certified laboratory, will be performed on two occasions prior to starting study therapy - once at screening and once at the C1D1visit, immediately before investigational product administration. The test must be repeated at the Final Study/Treatment Discontinuation Visit. Additional pregnancy tests may also be undertaken if requested by institutional review board/ethics committee (IRB/ECs) or if required by local regulations.

HBV, HCV and HIV: <u>HBV serology:</u> Hepatitis B surface antigen, hepatitis B surface antibody, and total hepatitis B core antibody. HBV DNA should be obtained prior to enrollment if patient has a negative serology for HbsAg and a positive serology for anti-HBcAb (see Section 5.2). <u>HCV serology</u>: HCV antibody (anti-HCV). HCV RNA should be

obtained prior to enrollment if patient tests positive for anti-HCV. HIV: antigen/antibody combination test.

Adverse Event (AE) Assessments: Adverse events will be documented and recorded continuously, starting at the screening visit, using the National Cancer Institute Common Terminology Criteria for Adverse Events (NCI CTCAE) version 5.0.

Registration: Site numbers are entered into the Electronic Data Capture (EDC) system when the EDC is created. Patient numbers (registration) are assigned by AGICC when the patient signs the Informed Consent Form (ICF) and the patient is entered into the EDC. Screening failures are captured in the EDC. Enrollment occurs when the Medical Monitor approves the screening documents with his/her signature.

Concomitant Treatments: All concomitant medications and Non-Drug Supportive Interventions should be recorded in the eCRF within 28 days prior to study entry (i.e., prior to C1D1). All concomitant medications taken during study participation will continue to be recorded continuously on the eCRF starting at the screening visit and ending 30 days after treatment discontinuation (See Section 6.6).

Tumor Assessment: Baseline tumor assessments should be performed ≤ 28 days before C1 D1 and assessed according to RECIST v1.1 and iRECIST (see Appendix 2), as described in Section 8.1.

Screening Assessments Performed After Confirming Eligibility

Prior to the pre-treatment biopsy (Cohort A only), patients must sign the Informed Consent Form and all other described screening evaluations (Section 8.2.1 – "Screening Assessments to Determine Eligibility") must be completed and reviewed by the treating clinician to confirm that all eligibility criteria are met.

Tumor Tissue Samples. Patients in Cohort A will provide a fresh pretreatment biopsy sample (before C1D1 but after screening) and an on-treatment biopsy sample at C3D1 (±3 days). While the acceptable window for the C3D1 biopsy is ±3 days, it is preferred for the biopsy to occur following all treatments on C3D1. The date and time of the biopsies will be documented. In Cohort A, archival tissue may not be used in place of a fresh pre-treatment biopsy. Patients in Cohort B will not undergo any study-related biopsies. When available, archival tissue will also be obtained for analysis for patients in both Cohort A and Cohort B. Other than biopsies, there are no differences between Cohort A and Cohort B (both receive the same treatment and study events). Details for handling of these samples including processing, storage, and shipment will be provided in the Laboratory Manual. See Section 4.1.4 for additional information regarding tumor biopsies.

8.2.2 TREATMENT PERIOD

On treatment study assessments and procedures include:

Physical Exam. A brief physical exam will be conducted at the time points indicated in Schedule of Events (Section 1.3)

Vital Signs. Vital signs (blood pressure (BP), heart rate (HR), and temperature to be recorded in the sitting position.) will be recorded at the time points indicated in Schedule of Events (Section 1.3). Vital signs at the first pembrolizumab infusion will be collected within 60 min prior to the infusion, every 15 (±10) min during the pembrolizumab infusion and 30 (±10) min after the infusion. For subsequent infusions, vital signs will be collected within 60 min prior to the infusion and should be collected during the infusion if clinically indicated or if symptoms occurred in the prior

Performance Status. Performance status will be recorded at the time points indicated in Schedule of Events (Section 1.3): use Eastern Cooperative Oncology Group (ECOG) performance status criteria (Appendix 3).

12-lead ECG: A single ECG will be collected on at least a 10 second strip at screening and post-infusion on C1D1, C1D8, and C3D1. No further study-mandated ECGs are needed after C3D1, except an ECG will be done at the Final study / treatment discontinuation visit. If the QTcF is prolonged (value of >500 msec), the ECG should be re-evaluated by a qualified person at the institution for confirmation. Additional ECGs should be performed as clinically indicated.

Blood/Urine Tests: The following blood/urine tests will be collected at the time points indicated in Schedule of Events (Section 1.3)

- **Hematology:** Complete blood count (CBC) to include hemoglobin, platelets, WBC, absolute neutrophils, lymphocytes, monocytes, eosinophils, and basophils.
- **Blood Chemistry:** Should include sodium, potassium, chloride, bicarbonate or carbon dioxide, BUN (or urea), creatinine, non-fasting glucose, calcium, magnesium, phosphorus, albumin, total protein, AST/SGOT, ALT/SGPT, alkaline phosphatase, total bilirubin, lipase, and amylase.
- **Coagulation:** Should include International Normalized Ratio (INR) and Partial Thromboplastin Time (PTT).
- **Urinalysis:** Dipstick is acceptable. Microscopic analyses if dipstick abnormal. If ≥2+ protein on urine dipstick, then collect spot urine sample to calculate urine protein to creatinine ratio (UPCR).
- **Serum Pregnancy Test:** For female patients of childbearing potential, a serum pregnancy test, with sensitivity of at least 25 mIU/mL, and assayed in a certified

laboratory, will be performed at C1D1, immediately before investigational product administration. Additional pregnancy tests may also be undertaken if requested by institutional review board/ethics committee (IRB/ECs) or if required by local regulations.
- **Carcinoembryonic antigen (CEA)**
- **TSH, free T4, and cortisol**

Research Blood Samples. Pharmacokinetic (PK), Pharmacodynamic (PD) biomarker and anti-drug antibody (ADA) blood samples will be drawn throughout this study. See the Laboratory Manual, Section 1.3, and Section 8.2.6 for additional information on the research blood samples.

- **PK** - Blood will be drawn for pharmacokinetic (PK) analysis prior to the start of evorpacept (ALX148) infusion and immediately following completion of ALX148 infusion (within 6 minutes after end of infusion) on Day 1 of Cycles 1-5. Starting with Cycle 6, the frequency of the pre- and post- evorpacept (ALX148) infusion PK draws will be decreased to every 3 cycles (i.e. C6D1, C9D1, C12D1, etc). A final blood draw for PK analysis will be performed at the Final Study Visit / Treatment Discontinuation Visit. In addition to samples collected at the scheduled times, an additional blood sample may be requested from patients experiencing unexpected and/or serious AE's with the date and time documented in the eCRF. Additional details can be found in the Laboratory Manual and Section 8.2.6.
- **ADA -** Blood will be drawn for anti-drug antibody (ADA) analysis prior to the start of evorpacept (ALX148) infusion on Day 1 of Cycles 1-5. Starting with Cycle 6, the frequency of the pre-evorpacept (ALX148) infusion ADA draws will be decreased to every 3 cycles (i.e. C6D1, C9D1, C12D1, etc). A final blood draw for ADA analysis will be performed at the Final Study Visit / Treatment Discontinuation Visit. In addition to samples collected at the scheduled times, an additional blood sample may be requested from patients experiencing unexpected and/or serious AE's with the date and time documented in the eCRF. Additional details can be found in the Laboratory Manual and Section 8.2.6.
- **PD/biomarker** – Blood will be drawn for pharmacodynamic (PD)/biomarker analyses prior to the start of evorpacept (ALX148) infusion at Day 1 of each cycle and at the Final Study Visit/Treatment Discontinuation Visit. Additional details can be found in the Laboratory Manual and Section 8.2.6.

Adverse Events. Adverse Event (AE) assessments will be done at the time points indicated in Schedule of Events (Section 1.3) and recorded continuously. Adverse events should be documented and recorded at each visit using the National Cancer Institute Common Terminology Criteria for Adverse Events (NCI CTCAE) version 5.0. Patients must be

followed for non-serious AEs from C1D1 up to 28 days after the last study treatment administration or until all drug-related toxicities have resolved, whichever is later; or earlier than 28 days should the patient commence another anticancer therapy in the meantime. For serious adverse events (SAEs), the active reporting period to AGICC or its designated representative begins from the time that the patient provides informed consent, which is obtained prior to the patient's participation in the study, ie, prior to undergoing any study-related procedure and/or receiving investigational product, through and including 28 calendar days after the last administration of the investigational product. SAEs experienced by a patient after the active reporting period has ended should be reported to AGICC if the Investigator becomes aware of them, unless the SAE is attributed by the Investigator to complications of either the underlying malignancy or any subsequent anti-cancer therapy or to the patient's participation in a subsequent clinical study; at a minimum, all SAEs that the Investigator believes have at least a reasonable possibility of being related to investigational product are to be reported to the AGICC. To collect late immune related adverse event information, all patients will be contacted by phone 90 days after EOT.

Concomitant Treatments. Concomitant Treatments assessments will be done at the time points indicated in Schedule of Events (Section 1.3) and recorded continuously. All concomitant medications and Non-Drug Supportive Interventions should be recorded in the eCRF until 30 days after treatment discontinuation (See Section 6.6).

Treatment with Study Drug. Patients will be observed in the clinic for at least 2 hours after infusion of evorpacept (ALX148) on C1D1 and as clinically indicated, thereafter.

Tumor Tissue Samples. Patients in Cohort A will provide a fresh pretreatment biopsy sample and an on-treatment biopsy sample at C3D1 (±3 days). Prior to the pre-treatment biopsy, patients must sign the Informed Consent Form and all other described screening evaluations (Section 8.2.1 – "Screening Assessments to Determine Eligibility") must be completed and reviewed by the treating clinician to confirm that all eligibility criteria are met. While the acceptable window for the C3D1 biopsy is ±3 days, it is preferred for the biopsy to occur following all treatments on C3D1. The date and time of the biopsies will be documented. In Cohort A, archival tissue may not be used in place of a fresh pre-treatment biopsy. Patients in Cohort B will not undergo any study-related biopsies. When available, archival tissue will also be obtained for analysis for patients in both Cohort A and Cohort B. Other than biopsies, there are no differences between Cohort A and Cohort B (both receive the same treatment and study events). Details for handling of these samples including processing, storage, and shipment will be provided in the Laboratory Manual. See Section 4.1.4 for additional information regarding tumor biopsies.

8.2.3 UNSCHEDULED VISIT

The Investigator will review the patient per standard of care and document all pertinent information as per “Treatment Period” above.

8.2.4 FINAL STUDY/TREATMENT DISCONTINUATION VISIT

A final study visit is required if patients come off study for any reason. At least 28 days and no more than 35 days after discontinuation of treatment, patients will return to undergo the activities listed in Schedule of Events (Section 1.3), unless the assessment(s) were already completed within the prior week, or the last 6 weeks for the tumor response assessment.

Patients continuing to experience toxicity at this point following discontinuation of treatment will continue to be followed at least every 4 weeks until resolution or determination, in the clinical judgment of the Investigator, that no further improvement is expected. If the patient has any concern and contacts the clinic, the patient will be seen in the clinic within 5 calendar days of the patient’s contact with the clinic (assessments will be the same as the assessments performed at the Final Study/Treatment Discontinuation Visit).

8.2.5 AFTER TREATMENT / SURVIVAL FOLLOW-UP

Overall survival information will be collected via telephone calls and/or clinic visits every 3 months ±14 days (first call and/or clinic visit to occur 3 months following the Final Study/Treatment Discontinuation Visit) until death, withdrawal of consent, the patient is lost to follow-up, study termination by AGICC, or a maximum of 2 years from the Final Study/Treatment Discontinuation Visit.

8.2.6 RESEARCH EVALUATIONS (AND SPECIMEN PREPARATION, HANDLING, STORAGE, AND SHIPMENT)

The University of Colorado Pathology Shared Resource will coordinate the sample collection of tissue and blood samples for research-related testing at central laboratories. A Laboratory Manual and supply kits will be provided for all central laboratory assessments including those listed in this section. For sampling procedures, storage conditions, and shipment instructions, please see the Laboratory Manual.

The expected analyses/assays are listed here. Additional assays may be performed depending on innovation and scientific progress in the field prior to sample analysis, and PK, ADA, or PD samples may be used for the analysis if appropriate.

Any remaining samples post analysis will be destroyed unless patients have given signed consent allowing optional banking of their remaining biological samples. See section 10.1.4 for additional information on the future use of stored data and specimens.

Pharmacokinetic (PK) assessments

Blood will be drawn for pharmacokinetic (PK) analysis prior to the start of evorpacept (ALX148) infusion and immediately following completion of evorpacept (ALX148) infusion (within 6 minutes after end of infusion) on Day 1 of Cycles 1-5. Starting with Cycle 6, the frequency of the pre- and post- evorpacept (ALX148) infusion PK draws will be decreased to every 3 cycles (i.e. C6D1, C9D1, C12D1, etc). A final blood draw for PK analysis will be performed at the Final Study Visit / Treatment Discontinuation Visit. In addition to samples collected at the scheduled times, an additional blood sample may be requested from patients experiencing unexpected and/or serious AEs with the date and time documented in the eCRF. Additional details can be found in the Laboratory Manual.

Anti-drug antibody (ADA) assessments

Blood will be drawn for anti-drug antibody (ADA) analysis prior to the start of evorpacept (ALX148) infusion on Day 1 of Cycles 1-5. Starting with Cycle 6, the frequency of the pre-evorpacept (ALX148) infusion ADA draws will be decreased to every 3 cycles (i.e. C6D1, C9D1, C12D1, etc). A final blood draw for ADA analysis will be performed at the Final Study Visit / Treatment Discontinuation Visit. In addition to samples collected at the scheduled times, an additional blood sample may be requested from patients experiencing unexpected and/or serious AEs with the date and time documented in the eCRF. Additional details can be found in the Laboratory Manual.

Peripheral blood pharmacodynamic (PD)/biomarker assessments

Blood will be drawn for pharmacodynamic (PD)/biomarker analyses prior to the start of evorpacept (ALX148) infusion at Day 1 of each cycle and at the Final Study Visit/Treatment Discontinuation Visit. In addition to samples collected at the scheduled times, an additional blood sample may be requested from patients experiencing unexpected and/or serious AEs with the date and time documented in the eCRF. Additional details can be found in the Laboratory Manual.

The phenotype and function (activation makers, signaling molecules, and cytokines) of peripheral immune cells will be compared before and after treatment in all patients using mass cytometry, through our collaboration with the Human Immune Monitoring Shared Resource (HIMSR) at the University of Colorado School of Medicine. Tumor biopsies are not always readily available and biomarkers of response to treatment in the peripheral blood may be more clinically impactful. Mass cytometry is a variant of flow cytometry that allows single cell analysis of up to 45 antibody specificities. After a brief stimulation in vitro, blood samples will be labeled with a unique barcode and stained with a large panel of antibodies (designed to detect the phenotype and activation of myeloid and lymphoid immune cells, as well as signaling molecules and cytokines) labeled with heavy metal ion tags rather than fluorochromes. The metal

barcodes allow up to 20 samples to be stained and analyzed simultaneously, increasing the high throughput capability of the instrument and decreasing technical variability. The signal intensities will be normalized across samples using polystyrene "normalizing" beads that are acquired simultaneously with every sample. Data will then be analyzed using the University of Colorado Denver's CytoBank software premium account, utilizing the unsupervised clustering tool ViSNE, the population visualization tool SPADE, and the two-sample comparison tool Citrus. We will measure the change in phenotype and function of immune cells post-treatment compared with pre-treatment, and correlate these parameters with ORR, PFS, and OS.

Peripheral blood will be analyzed using a circulating tumor DNA (ctDNA) platform, to assess oncogenic mutations and mutational loads (tumor mutational burden). Exploratory analyses may be performed correlating response to treatment with evorpacept (ALX148), cetuximab, and pembrolizumab with molecular findings, including the use of serial ctDNA levels to predict response at the time of pseudoprogression.

Tumor pharmacodynamic (PD)/biomarker assessments

Patients in Cohort A will provide a fresh pretreatment biopsy sample and an on-treatment biopsy sample at C3D1 (±3 days). Prior to the pre-treatment biopsy, patients must sign the Informed Consent Form and all other described screening evaluations (Section 8.2.1 – "Screening Assessments to Determine Eligibility") must be completed and reviewed by the treating clinician to confirm that all eligibility criteria are met. While the acceptable window for the C3D1 biopsy is ±3 days, it is preferred for the biopsy to occur following all treatments on C3D1. The date and time of the biopsies will be documented. In Cohort A, archival tissue may not be used in place of a fresh pre-treatment biopsy. Patients in Cohort B will not undergo any study-related biopsies. When available, archival tissue will also be obtained for analysis for patients in both Cohort A and Cohort B. Other than biopsies, there are no differences between Cohort A and Cohort B (both receive the same treatment and study events). Details for handling of these samples including processing, storage, and shipment will be provided in the Laboratory Manual. See Section 4.1.4 for additional information regarding tumor biopsies.

The University of Colorado Pathology Shared Resource will coordinate the sample collection of tissue for research-related testing at central laboratories. A Laboratory Manual and supply kits will be provided for all central laboratory assessments.

Tumor biopsy acquisition

- Fine-needle aspiration, brushing, cell pellet from pleural effusion, and lavage samples are not acceptable.
- For core needle biopsy specimens, at least two cores (maximum four cores) should be submitted for evaluation at each biopsy (pre-treatment and on-treatment). Details for

acquiring, processing, storing, and shipping these samples will be provided in the Laboratory Manual.

- At the University of Colorado only, two additional core biopsies should be obtained at the pretreatment biopsy. These should be immediately placed into a provided specimen cup with RPMI. The container should immediately be brought to the Pitts lab for injection into mice. This process has been approved by the Institutional Animal Research Committee.

Safely biopsied disease is defined as:

- cutaneous lesions (without evidence of active infection)
- peripherally accessible lymph nodes (cervical, axillary, inguinal, extremities)
- liver metastases (not immediately adjacent to major vessels)
- lung metastases easily accessible by percutaneous biopsy or endobronchial ultrasound
- rectal lesions

Lesions not allowed for biopsy include brain, mediastinum, pelvis, or any lesion deemed by the patient's primary oncologist, study investigator, or radiologist to represent greater risk to the subject.

If non-evaluable tumor tissue is collected from the on-study biopsy procedures, (e.g., necrotic or fibrotic tissue, or the procedure is interrupted because of a complication), the patient will still be eligible to continue treatment.

Tumor biopsy assessments

Tumor tissue will be assessed using 9-color multispectral imaging and mRNA expression.

Nine-color multispectral imaging

The frequency and activation status of infiltrating immune cells in the tumor microenvironment will be compared before and after treatment using 9-color multispectral imaging. Through our collaboration with the Human Immune Monitoring Shared Resource (HIMSR) at the University of Colorado School of Medicine we will phenotype, quantify, and determine the spatial relationship of immune infiltrate in formalin-fixed paraffin-embedded biopsies. We will determine the presence and activation status of myeloid and lymphoid cells in the tumor microenvironment, determine the level of inhibitory markers (CD47, PD-1, and PD-L1), and measure EGFR expression on tumors by staining slides from untreated and treated biopsy specimens with antibodies. Mismatch repair status will also be assessed at baseline.

After staining, whole slide scans will be collected using the 10x objective and approximately 20 regions of interest covering the entire tissue biopsy will be selected. Multispectral images of each region of interest will then be collected using the 20x objective with a 0.5-micron resolution. The 9-color images will be analyzed with inForm software to segment the tissue and

compare the frequency and location of immune cell infiltrate in tumor and stromal areas, to phenotype the cells according to morphology and cell marker expression, and to score each cell for expression of activation and inhibitory molecules. The frequency of each immune cell subset, the activation status of those subsets, the expression of inhibitory molecules, and their location relative to tumor regions in post-treatment biopsies will be compared to the baseline biopsy in each patient to determine whether treatment with evorpacept (ALX148), cetuximab, and pembrolizumab reprograms the tumor microenvironment. The pre- and post-treatment biopsies will also be compared between patients that respond to treatment and patients that are refractory to treatment.

mRNA expression

Pre-treatment and post-treatment mRNA expression will be measured using the NanoString PanCancer IO 360 Gene Expression Panel. This panel analyzes 770 genes and will be used to characterize mRNA expression in the tumor, tumor microenvironment, and immune response. Changes in gene expression signatures will be compared to the baseline biopsy in each patient to determine whether treatment with evorpacept (ALX148), cetuximab, and pembrolizumab reprograms the tumor microenvironment. The pre- and post-treatment biopsies will also be compared between patients that respond to treatment and patients that are refractory to treatment. Finally, baseline gene expression signatures will be correlated with response to treatment as a possible predictive biomarker.

8.3 ADVERSE EVENTS AND SERIOUS ADVERSE EVENTS

8.3.1 DEFINITION OF ADVERSE EVENTS (AE)

Adverse event means any untoward medical occurrence associated with the use of an intervention in humans, whether or not considered intervention-related (21 CFR 312.32 (a)).

8.3.2 DEFINITION OF SERIOUS ADVERSE EVENTS (SAE)

Serious adverse event or **serious suspected adverse reaction**. An AE or suspected adverse reaction is considered “serious” if, in the view of either the investigator or AGICC, it results in any of the following outcomes: death, a life-threatening adverse event, inpatient hospitalization or prolongation of existing hospitalization, a persistent or significant incapacity or substantial disruption of the ability to conduct normal life functions, a congenital anomaly/ birth defect, or other serious events that may jeopardize the patient and may require medical or surgical intervention to prevent one of the other listed outcomes. Important medical events that may not result in death, be life-threatening, or require hospitalization may be considered serious when, based upon appropriate medical judgment, they may jeopardize the patient or subject and may require medical or surgical intervention to prevent one of the outcomes listed in this definition. Examples of such medical events include allergic bronchospasm requiring intensive treatment in

an emergency room or at home, blood dyscrasias or convulsions that do not result in inpatient hospitalization, or the development of drug dependency or drug abuse.

In addition to the above criteria, AEs meeting either of the below criteria, although not serious per International Conference on Harmonization definition, are reportable to Merck in the same time frame as SAEs to meet certain local requirements. Therefore, these events are considered serious by Merck for collection purposes.

- Is a new cancer (that is not a condition of the study)
- Is associated with an overdose of pembrolizumab

8.3.3 CLASSIFICATION OF AN ADVERSE EVENT

8.3.3.1 SEVERITY OF EVENT

The adverse event severity grading scale for the NCI CTCAE (v5.0) will be used for assessing adverse event severity. The following table will be used for assessing severity for adverse events that are not specifically listed in the NCI CTCAE (v5.0).

Grade	Severity
1	Mild; asymptomatic or mild symptoms; clinical or diagnostic observations only; or intervention not indicated
2	Moderate; minimal, local, or non-invasive intervention indicated; or limiting age-appropriate instrumental activities of daily living [a]
3	Severe or medically significant, but not immediately life-threatening; hospitalization or prolongation of hospitalization indicated; disabling; or limiting self-care activities of daily living [b,c]
4	Life-threatening consequences or urgent intervention indicated
5	Death related to adverse event

NCI CTCAE: National Cancer Institute Common Terminology Criteria for Adverse Events. Note: Based on the most recent version of NCI CTCAE (v5.0), which can be found at: *http://ctep.cancer.gov/protocolDevelopment/electronic_applications/ctc.htm*

[a] Instrumental activities of daily living refer to preparing meals, shopping for groceries or clothes, using the telephone, managing money, etc.

[b] Examples of self-care activities of daily living include bathing, dressing and undressing, feeding oneself, using the toilet, and taking medications, as performed by patients who are not bedridden.

[c] If an event is assessed as a "significant medical event," it must be reported as a serious adverse event.

8.3.3.2 RELATIONSHIP TO STUDY INTERVENTION

The clinician's assessment of an AE's relationship to study agent (drug, biologic, device) is part of the documentation process, but it is not a factor in determining what is or is not

reported in the study. If there is any doubt as to whether a clinical observation is an AE, the event should be reported. All AEs must have their relationship to the study agents assessed. In a clinical trial, the study product must always be suspect.

For all collected AEs, the clinician who examines and evaluates the participant will determine the AE's causality based on temporal relationship and his/her clinical judgment. The degree of certainty about causality will be graded using the categories below.

- **Definitely Related** – There is clear evidence to suggest a causal relationship, and other possible contributing factors can be ruled out. The clinical event, including an abnormal laboratory test result, occurs in a plausible time relationship to drug administration and cannot be explained by concurrent disease or other drugs or chemicals. The response to withdrawal of the drug (dechallenge) should be clinically plausible. The event must be pharmacologically or phenomenologically definitive, with use of a satisfactory rechallenge procedure if necessary.
- **Probably Related** – There is evidence to suggest a causal relationship, and the influence of other factors is unlikely. The clinical event, including an abnormal laboratory test result, occurs within a reasonable time after administration of the drug, is unlikely to be attributed to concurrent disease or other drugs or chemicals, and follows a clinically reasonable response on withdrawal (dechallenge). Rechallenge information is not required to fulfill this definition.
- **Possibly Related** – There is some evidence to suggest a causal relationship (e.g., the event occurred within a reasonable time after administration of the trial medication). However, other factors may have contributed to the event (e.g., the participant's clinical condition, other concomitant events). Although an AE may rate only as "possibly related" soon after discovery, it can be flagged as requiring more information and later be upgraded to "probably related" or "definitely related", as appropriate.
- **Unlikely to be Related** – A clinical event, including an abnormal laboratory test result, whose temporal relationship to drug administration makes a causal relationship improbable (e.g., the event did not occur within a reasonable time after administration of the trial medication) and in which other drugs or chemicals or underlying disease provides plausible explanations (e.g., the participant's clinical condition, other concomitant treatments).
- **Not Related** – The AE is completely independent of study drug administration, and/or evidence exists that the event is definitely related to another etiology. There must be an alternative, definitive etiology documented by the clinician.

8.3.3.3 EXPECTEDNESS

Expectedness will only be documented for SAEs. The medical monitor (in discussion with the study investigators) will be responsible for determining whether an SAE is expected or unexpected. An SAE will be considered unexpected if the nature, severity, or frequency of the event is not consistent with the risk information previously described for the study agent.

8.3.4 TIME PERIOD AND FREQUENCY FOR EVENT ASSESSMENT AND FOLLOW-UP

The occurrence of an AE or SAE may come to the attention of study personnel during study visits and interviews of a study participant presenting for medical care, or upon review by a study monitor. All AEs including local and systemic reactions not meeting the criteria for SAEs will be captured on the appropriate eCRF. Information to be collected includes event description, time of onset, clinician's assessment of severity, relationship to study product (assessed only by those with the training and authority to make a diagnosis), and time of resolution/ stabilization of the event. All AEs occurring while on study must be documented appropriately regardless of relationship. All AEs will be followed to adequate resolution.

Any medical condition that is present at the time that the participant is screened will be considered as baseline and not reported as an AE. However, if the study participant's condition deteriorates at any time during the study, it will be recorded as an AE. UAPs will be recorded in the data collection system throughout the study.

Changes in the severity of an AE will be documented to allow an assessment of the duration of the event at each level of severity to be performed. AEs characterized as intermittent require documentation of onset and duration of each episode.

The PI will record all reportable events with start dates occurring any time after informed consent is obtained until 90 days after the last day of study treatment or until initiation of new treatment, whichever comes first. SAEs will be followed until resolution or stabilization. At each study visit, the investigator will inquire about the occurrence of AE/ SAEs since the last visit.

8.3.5 ADVERSE EVENT REPORTING

The adverse event severity grading scale for the NCI CTCAE (v5.0) will be used for assessing adverse event severity as noted in Section 8.3.3.1. For each adverse event recorded on the Adverse Event eCRF, the investigator will make an assessment of seriousness, severity, and causality. Investigators will seek information on adverse events at each patient contact. All adverse events, whether reported by the patient or noted by study personnel, will be recorded in the patient's medical record and on the Adverse Event eCRF. Investigators should use correct medical terminology/concepts when recording adverse events on the Adverse Event eCRF.

Avoid colloquialisms and abbreviations. Only one adverse event term should be recorded in the event field on the Adverse Event eCRF.

After informed consent has been obtained but prior to initiation of study drug, only serious adverse events caused by a protocol-mandated intervention (e.g., invasive procedures such as biopsies, discontinuation of medications) should be reported. After initiation of study drug, all adverse events will be reported until 90 days after the last dose of study drug.

Adverse Events that are secondary to other events

In general, adverse events that are secondary to other events (e.g., cascade events or clinical sequelae) should be identified by their primary cause, with the exception of severe or serious secondary events. A medically significant secondary adverse event that is separated in time from the initiating event should be recorded as an independent event on the Adverse Event eCRF. For example:

- If vomiting results in mild dehydration with no additional treatment in a healthy adult, only vomiting should be reported on the eCRF.
- If vomiting results in severe dehydration, both events should be reported separately on the eCRF.
- If a severe gastrointestinal hemorrhage leads to renal failure, both events should be reported separately on the eCRF.
- If dizziness leads to a fall and consequent fracture, all three events should be reported separately on the eCRF.
- If neutropenia is accompanied by an infection, both events should be reported separately on the eCRF.

All adverse events should be recorded separately on the Adverse Event eCRF if it is unclear as to whether the events are associated.

Persistent or Recurrent Adverse Events

A persistent adverse event is one that extends continuously, without resolution, between patient evaluation time-points. Such events should only be recorded once on the Adverse Event eCRF. The initial severity (intensity or grade) of the event will be recorded at the time the event is first reported. If a persistent adverse event becomes more severe, the most extreme severity should also be recorded on the Adverse Event eCRF. If the event becomes serious, it should be reported to AGICC (i.e., no more than 24 hours after learning that the event became serious). The Adverse Event eCRF should be updated by changing the event from "non-serious" to "serious," providing the date that the event became serious, and completing all data fields related to serious adverse events.

A recurrent adverse event is one that resolves between patient evaluation time-points and subsequently recurs. Each recurrence of an adverse event should be recorded as a separate event on the Adverse Event eCRF

Abnormal Laboratory Values

- Not every laboratory abnormality qualifies as an adverse event. A laboratory test result must be reported as an adverse event if it meets any of the following criteria:
- Is accompanied by clinical symptoms
- Results in a change in study treatment (e.g., dosage modification, treatment interruption, or treatment discontinuation)
- Results in a medical intervention (e.g., potassium supplementation for hypokalemia) or a change in concomitant therapy
- Is clinically significant in the investigator's judgment

It is the investigator's responsibility to review all laboratory findings. Medical and scientific judgment should be exercised in deciding whether an isolated laboratory abnormality should be classified as an adverse event.

If a clinically significant laboratory abnormality is a sign of a disease or syndrome (e.g., alkaline phosphatase and bilirubin 5x ULN associated with cholestasis), only the diagnosis (i.e. bile duct stenosis) should be recorded on the Adverse Event eCRF.

If a clinically significant laboratory abnormality is not a sign of a disease or syndrome, the abnormality itself should be recorded on the Adverse Event eCRF, along with a descriptor indicating whether the test result is above or below the normal range (e.g., "elevated potassium," as opposed to "abnormal potassium"). If the laboratory abnormality can be characterized by a precise clinical term per standard definitions, the clinical term should be recorded as the adverse event. For example, an elevated serum potassium level of 7.0 mEq/L should be recorded as "hyperkalemia."

Observations of the same clinically significant laboratory abnormality from visit to visit should only be recorded once on the Adverse Event eCRF.

Abnormal Vital Sign Values

Not every vital sign abnormality qualifies as an adverse event. A vital sign result must be reported as an adverse event if it meets any of the following criteria:

- Is accompanied by clinical symptoms
- Results in a change in study treatment (e.g., dosage modification, treatment interruption, or treatment discontinuation)

- Results in a medical intervention or a change in concomitant therapy
- Is clinically significant in the investigator's judgment

It is the investigator's responsibility to review all vital sign findings. Medical and scientific judgment should be exercised in deciding whether an isolated vital sign abnormality should be classified as an adverse event.

If a clinically significant vital sign abnormality is a sign of a disease or syndrome (e.g., high blood pressure), only the diagnosis (i.e., hypertension) should be recorded on the Adverse Event eCRF.

Preexisting Medical Conditions

A preexisting medical condition should be recorded as an adverse event only if the frequency, severity, or character of the condition worsens during the study. When recording such events on the Adverse Event eCRF, it is important to convey the concept that the preexisting condition has changed by including applicable descriptors (e.g., "more frequent headaches").

Hospitalization or Prolonged Hospitalization

Any adverse event that results in hospitalization (i.e., inpatient admission to a hospital) or prolonged hospitalization should be documented and reported as a serious adverse event (per the definition of serious adverse event in Section 8.3.2), except as outlined below.

The following hospitalization scenarios are not considered to be adverse events:

- Hospitalization for respite care.
- Planned hospitalization required by the protocol (e.g., for study drug administration or insertion of access device for study drug administration).
- Hospitalization for a preexisting condition, provided that all of the following criteria are met:
- The hospitalization was planned prior to the study or was scheduled during the study when elective surgery became necessary because of the expected normal progression of the disease.
- The patient has not experienced an adverse event.
- Hospitalization due solely to progression of the underlying cancer.

The following hospitalization scenarios are not considered to be serious adverse events, but should be reported as adverse events instead:

- Hospitalization that was necessary because of patient requirement for outpatient care outside of normal outpatient clinic operating hours.

Overdose or Error in Drug Administration

An overdose is the accidental or intentional use of a drug in an amount higher than the dose being studied. An overdose or incorrect administration of study treatment is not itself an adverse event, but it may result in an adverse event. All adverse events associated with an overdose or incorrect administration of study drug should be recorded on the Adverse Event eCRF. If the associated adverse event fulfills seriousness criteria, the event should be reported to AGICC immediately.

Deaths
Deaths that occur during the protocol- specified adverse event reporting period should be entered in the Adverse Event eCRF. All on-study deaths, regardless of relationship to study drug, must be recorded on the Adverse Event eCRF and immediately reported to AGICC.

Death should be considered an outcome and not a distinct event. The event or condition that caused or contributed to the fatal outcome should be recorded as the single medical concept on the Adverse Event eCRF. Generally, only one such event should be reported. The term "sudden death" should be used only for the occurrence of an abrupt and unexpected death due to presumed cardiac causes in a patient with or without preexisting heart disease, within 1 hour after the onset of acute symptoms or, in the case of an unwitnessed death, within 24 hours after the patient was last seen alive and stable. If the cause of death is unknown and cannot be ascertained at the time of reporting, "unexplained death" should be recorded on the Adverse Event eCRF. If the cause of death later becomes available (e.g., after autopsy), "unexplained death" should be replaced by the established cause of death.

During survival follow-up, deaths should be entered in the Adverse Event eCRF.

Adverse Events After the Reporting Period
After the end of the adverse event reporting period, all deaths, regardless of cause, should be reported through use of the Long-Term Survival Follow-Up eCRF. In addition, if the investigator becomes aware of a serious adverse event that is believed to be related to prior study drug treatment, the event should be reported through use of the Adverse Event eCRF.

8.3.6 SERIOUS ADVERSE EVENT REPORTING

Investigators and Institutions will comply with applicable laws, regulations and standards regarding Investigator’s and Institution’s obligations.

The investigator must record all serious adverse events, complete an SAE Form, and report to DSMC and IRB according to the following timelines:

- All deaths and immediately life-threatening events, whether related or unrelated, will be recorded on the SAE Form and submitted per the instructions below within 24 hours of site awareness.
- Other SAEs, regardless of relationship, will be submitted per the instructions below within 24 hours of site awareness.

SAEs will be submitted to the following email addresses within 24 hours of becoming aware of the event:

To:	*robert.lentz@CUAnschutz.edu*
	ALX148SAE@criteriuminc.com
Subject:	AGICC-ALX148 21CRC01 SAE Report Form
Attach:	SAE form completed and signed by the Investigator

After receiving notification of an SAE, AGICC, as the sponsor of the Study, will collect and report SAEs to Investigators, regulatory authorities (with the exception of local IRBs, who local investigators will report to in accordance with applicable requirements), Ethics Committees, or other third parties. AGICC will report to regulatory authorities within 7 calendar days for events that are life-threatening or result in death, and within 15 calendar days for all other events. In addition to the obligations set forth below, AGICC agrees to provide ALX Oncology Ltd, Merck & Co, Inc., & Eli Lilly USA, LLC with a copy of all information AGICC submits to regulators related to any SAEs for the Study Drug(s) that occur during the Study that AGICC has not otherwise provided ALX Oncology Ltd, Merck & Co, Inc., & Eli Lilly USA, LLC.

AGICC will notify Investigators, ALX Oncology Ltd, Merck & Co, Inc., & Eli Lilly USA, LLC of any problems involving risk to Study patients. Investigators will report new safety information to local IRBs in accordance with applicable requirements.

AGICC will notify ALX Oncology Ltd, Merck & Co, Inc., & Eli Lilly USA, LLC within 2 business days but no longer than 3 calendar days of AGICC receiving notification of any SAE experienced by a patient participating in the Study and receiving Study Drug(s) that is possibly related, based on Investigator's assessment, to the Study Drug(s). Serious adverse events should be reported to ALX Oncology Ltd, Merck & Co, Inc., & Eli Lilly USA, LLC using a CIOMS Form or other form acceptable to ALX Oncology Ltd, Merck & Co, Inc., & Eli Lilly USA, LLC. AGICC further agrees to make available promptly to ALX Oncology Ltd, Merck & Co, Inc., & Eli Lilly USA, LLC such records as may be necessary and pertinent for ALX Oncology Ltd,

Merck & Co, Inc., & Eli Lilly USA, LLC to further investigate an adverse event in the Study that is possibly associated with the Study Drug(s).

SAE reports and any other relevant safety information are to be forwarded to the MSD Global Safety facsimile number: +1-215-661-6229. A copy of all 15 Day Reports and Annual Progress Reports is submitted as required by FDA, European Union (EU), Pharmaceutical and Medical Devices agency (PMDA) or other local regulators. Investigators will cross reference this submission according to local regulations to the MSD Investigational Compound Number (IND, CSA, etc.) at the time of submission. Additionally, investigators will submit a copy of these reports to MSD (Attn: Worldwide Product Safety; FAX 215-661-6229) at the time of submission to FDA.

Follow-up of unresolved serious adverse events

Any SAEs that are unresolved at the time of the initial report submission should be followed up by the investigator for as long as medically indicated, and an updated SAE report submitted at the time new information regarding the event becomes available.

See Section 8.4.2 for Reporting of Unanticipated Problems.

8.3.7 REPORTING OF PREGNANCY

Pregnancies in Female Patients

Female patients of childbearing potential will be instructed to immediately inform the investigator if they become pregnant during the study or within 180 days after the last dose of study drug. A pregnancy report should be completed and submitted to AGICC or its designee immediately (i.e., no more than 24 hours after learning of the pregnancy), either by faxing or by scanning and emailing the form using the fax number or email address provided to investigators. Pregnancy should not be recorded on the Adverse Event eCRF. The investigator should discontinue study drug and counsel the patient, discussing the risks of the pregnancy and the possible effects on the fetus. Monitoring of the patient should continue until conclusion of the pregnancy. Any serious adverse events associated with the pregnancy (e.g., an event in the fetus, an event in the mother during or after the pregnancy, or a congenital anomaly/birth defect in the child) should be reported on the Adverse Event eCRF. In addition, the investigator will submit a Clinical Trial Pregnancy Reporting Form when updated information on the course and outcome of the pregnancy becomes available. AGICC will notify ALX Oncology Ltd, Merck & Co, Inc., & Eli Lilly USA, LLC LLC within 2 business days but no longer than 3 calendar days of AGICC receiving notification of pregnancy or lactation exposure.

Pregnancies in Female Partners of Male Patients

Male patients will be instructed through the Informed Consent Form to immediately inform the investigator if their partner becomes pregnant during the study or within 180 days after the last dose of study drug. A Clinical Trial Pregnancy Reporting Form should be completed and submitted to AGICC or its designee immediately (i.e., no more than 24 hours after learning of the pregnancy), either by faxing or by scanning and emailing the form using the fax number or email address provided to investigators. Attempts should be made to collect and report details of the course and outcome of any pregnancy in the partner of a male patient exposed to study drug. An investigator who is contacted by the male patient or his pregnant partner may provide information on the risks of the pregnancy and the possible effects on the fetus, to support an informed decision in cooperation with the treating physician and/or obstetrician. AGICC will notify ALX Oncology Ltd, Merck & Co, Inc., & Eli Lilly USA, LLC LLC within 2 business days but no longer than 3 calendar days of AGICC receiving notification of pregnancy or lactation exposure.

Abortions
Any abortion should be classified as a serious adverse event, recorded on the Adverse Event eCRF, and reported to AGICC immediately (i.e., no more than 24 hours after learning of the event).

Congenital Anomalies/Birth Defects
Any congenital anomaly/birth defect in a child born to a female patient exposed to study drug or the female partner of a male patient exposed to study drug should be classified as a serious adverse event, recorded on the Adverse Event eCRF, and reported to AGICC immediately (i.e., no more than 24 hours after learning of the event).

8.4 UNANTICIPATED PROBLEMS

8.4.1 DEFINITION OF UNANTICIPATED PROBLEMS (UAP)

The Office of Human Research Protection (OHRP) considers unanticipated problems involving risks to participants or others to include, in general, any incident, experience, or outcome that meets **all** of the following criteria:

- Unexpected in terms of nature, severity, or frequency given (a) the research procedures that are described in the protocol-related documents, such as the IRB-approved research protocol and informed consent document; and (b) the characteristics of the participant population being studied;
- Related or possibly related to participation in the research ("possibly related" means there is a reasonable possibility that the incident, experience, or outcome may have been caused by the procedures involved in the research); and

- Suggests that the research places participants or others at a greater risk of harm (including physical, psychological, economic, or social harm) than was previously known or recognized.

This study will use the OHRP definition of UAP.

8.4.2 REPORTING OF UNANTICIPATED PROBLEMS

Incidents or events that meet the OHRP criteria for UAPs require the creation and completion of a UAP report. It is the Site PI's responsibility to report UAPs to their IRB per institutional policies. The Lead PI is responsible for reporting the UAP to the IRB and the DSMC. The UAP report will include the following information:

- Protocol-identifying information: protocol title and number, PI's name, and the IRB project number;
- A detailed description of the event, incident, experience, or outcome;
- An explanation of the basis for determining that the event, incident, experience, or outcome represents a UAP;
- A description of any changes to the protocol or other corrective actions that have been taken or are proposed in response to the UAP.

The investigator must record all UAPs, complete a UAP Form, and report to DSMC and IRB within 24 hours of site awareness.

UAPs will be submitted to the following email addresses within 24 hours of becoming aware of the event:

To:	*robert.lentz@CUAnschutz.edu*<br>*ALX148SAE@criteriuminc.com*
Subject:	AGICC-ALX148 21CRC01 UAP Report Form
Attach:	UAP form completed and signed by the Investigator

After receiving notification of an UAP, AGICC, as the sponsor of the Study, will collect and report UAPs to Investigators, regulatory authorities (with the exception of local IRBs, who local investigators will report to in accordance with applicable requirements), Ethics Committees, or other third parties. AGICC will report to regulatory authorities within 7 calendar days for events that are life-threatening or result in death, and within 15 calendar days for all other events. In addition to the obligations set forth below, AGICC agrees to provide ALX Oncology Ltd, Merck & Co, Inc., & Eli Lilly USA, LLC with a copy of all information AGICC submits to regulators related to any UAPs for the Study Drug(s) that occur during the Study that AGICC has not otherwise provided ALX Oncology Ltd, Merck & Co, Inc., & Eli Lilly USA, LLC.

AGICC will notify Investigators, ALX Oncology Ltd, Merck & Co, Inc., & Eli Lilly USA, LLC of any problems involving risk to Study patients. Investigators will report new safety information to local IRBs in accordance with applicable requirements.

AGICC will notify ALX Oncology Ltd, Merck & Co, Inc., & Eli Lilly USA, LLC LLC within 2 business days but no longer than 3 calendar days of AGICC receiving notification of any UAP experienced by a patient participating in the Study and receiving Study Drug(s) that is possibly related, based on Investigator's assessment, to the Study Drug(s). UAPs should be reported to ALX Oncology Ltd, Merck & Co, Inc., & Eli Lilly USA, LLC using a CIOMS Form or other form acceptable to ALX Oncology Ltd, Merck & Co, Inc., & Eli Lilly USA, LLC. AGICC further agrees to make available promptly to ALX Oncology Ltd, Merck & Co, Inc., & Eli Lilly USA, LLC such records as may be necessary and pertinent for ALX Oncology Ltd, Merck & Co, Inc., & Eli Lilly USA, LLC to further investigate an adverse event in the Study that is possibly associated with the Study Drug(s).

8.5 EVENTS OF CLINICAL INTEREST

Selected nonserious and SAEs are also known as events of clinical interest (ECIs) and must be reported to Merck by AGICC.

Events of clinical interest for this study include:

- An overdose of pembrolizumab that is not associated with clinical symptoms or abnormal laboratory results. For purposes of this study, an overdose of pembrolizumab will be defined as any dose of 1,000 mg or greater (≥5 times the indicated dose). No specific information is available on the treatment of overdose of pembrolizumab. In the event of overdose, the participant should be observed closely for signs of toxicity. Appropriate supportive treatment should be provided if clinically indicated. If an adverse event(s) is associated with ("results from") the overdose of a Merck product, the adverse event(s) is reported as a serious adverse event, even if no other seriousness criteria are met.
- An elevated AST or ALT lab value that is greater than or equal to 3X the upper limit of normal and an elevated total bilirubin lab value that is greater than or equal to 2X the upper limit of normal and, at the same time, an alkaline phosphatase lab value that is less than 2X the upper limit of normal, as determined by way of protocol-specified laboratory testing or unscheduled laboratory testing.*

*Note: These criteria are based upon available regulatory guidance documents. The purpose of the criteria is to specify a threshold of abnormal hepatic tests that may require an additional evaluation for an underlying etiology.

9 STATISTICAL CONSIDERATIONS

9.1 STATISTICAL HYPOTHESES

Primary Hypothesis:

1. Evorpacept (ALX148), cetuximab, and pembrolizumab will demonstrate improved objective response rate (ORR, per RECIST v1.1) compared to historical controls

Secondary Hypotheses:

1. Evorpacept (ALX148), cetuximab, and pembrolizumab will demonstrate improved disease control rate (DCR, per RECIST v1.1) compared to historical controls
2. Evorpacept (ALX148), cetuximab, and pembrolizumab will demonstrate improved duration of response (DOR, per RECIST v1.1) compared to historical controls
3. Evorpacept (ALX148), cetuximab, and pembrolizumab will demonstrate improved progression-free survival (PFS, per RECIST v1.1) compared to historical controls
4. Evorpacept (ALX148), cetuximab, and pembrolizumab will demonstrate improved overall survival (OS) compared to historical controls
5. Evorpacept (ALX148), cetuximab, and pembrolizumab will be safe and tolerable, defined and graded according to the NCI CTCAE v5.0

Exploratory Hypotheses:

1. Evorpacept (ALX148), cetuximab, and pembrolizumab will demonstrate improved objective response rate (ORR), disease-control rate (DCR), duration of response (DOR, and progression-free survival (PFS) per iRECIST compared to historical controls
2. After treatment with evorpacept (ALX148), cetuximab, and pembrolizumab, there will be an enhanced immune response in the peripheral blood and tumor tissue compared to pre-treatment
3. Immune profiles in patients with an objective response to evorpacept (ALX148), cetuximab, and pembrolizumab will show enhanced immune response in the tumor and peripheral blood compared to non-responders (by RECIST v1.1 and iRECIST)
4. Objective response rate will not correlate with pre-treatment PD-L1 tumor expression by immunohistochemistry (by RECIST v1.1 and iRECIST)
5. Objective response rate will not correlate with pre-treatment EGFR tumor expression by immunohistochemistry (by RECIST v1.1 and iRECIST)
6. Objective response rate will not correlate with pre-treatment CD47 tumor expression by immunohistochemistry (by RECIST v1.1 and iRECIST)
7. Objective response rate will be higher for patients with high tumor mutational burden (TMB, >10 mut/Mb) than for patients with low TMB (<10 mut/Mb) (by RECIST v1.1 and iRECIST)

9.2 SAMPLE SIZE DETERMINATION

The sample size for stage 1 is not based on formal statistical calculations. Rather, it is based on a desire to obtain sufficient safety and tolerability information while exposing as few patients as possible to the investigational treatments.

The sample size for stage 2 has been determined as follows. The currently available third-line treatment options for MSS mCRC are regorafenib and trifluridine-tipiracil, however ORR is <5% and improvements in PFS and OS minimal, if any (Appendix 4. As previously discussed, expected response to single agent treatment with either anti-PD-1 or EGFR inhibitor in this patient population is extremely poor (Appendix 12, including linked references). Efficacy data from currently available third-line treatment options for MSS mCRC will comprise an estimation of the historical control (null hypothesis): ORR 3%, DCR 45%, and 6-month PFS probability 20%. In this study, we estimate ORR 15%, DCR 65%, and 6-month PFS probability 40%.

In paired pre- and post-treatment biopsies in patients with lung cancer and head/neck cancer treated with evorpacept (ALX148) and pembrolizumab, mean intra-tumoral pre- and post-treatment staining with CD68, CD163, and CD8 was 9 to 13%, 11 to 17%, and 8 to 10%, respectively.[59] Based on this data, in this study, we will assume an expected mean of the paired differences of 5% with an expected standard deviation of the paired differences of 7%.

9.3 POPULATION FOR ANALYSES

All analyses will be conducted using the safety-evaluable population, defined as all patients who receive any amount of study drug. Exploratory efficacy analyses will also be conducted using the response-evaluable population (see Section 12.2 for definition).

9.4 STATISTICAL ANALYSES

9.4.1 GENERAL APPROACH

The primary analysis will be based on patient data collected through study discontinuation or at the end of study. All analyses will be conducted using the safety-evaluable population, defined as all patients who receive any amount of study drug. Exploratory efficacy analyses will also be conducted using the response-evaluable population (see Section 12.2 for definition).

Summary descriptive statistics will be calculated and presented for all primary, secondary, safety, and exploratory outcomes for all participants, and for stratifications defined by participant demographic characteristics. The demographic characteristics of the participants will be presented with descriptive statistics also. The means, medians, and 95% confidence intervals will

be calculated for continuous variables; the frequencies, estimated rates, and the exact 95% confidence intervals will be calculated for binary and categorical variables.

The safety, tolerability, and clinical activity of evorpacept (ALX148), cetuximab, and pembrolizumab will be described and summarized, as warranted by sample size. That is, listings may be used in lieu of tables in the event of small sample size. Analysis of the primary efficacy, secondary efficacy, and exploratory endpoints will be performed as described in sections 9.4.2, 9.4.3, and 9.4.8, respectively. Safety analyses will be performed as described in section 9.4.4.

9.4.2 ANALYSIS OF THE PRIMARY EFFICACY HYPOTHESIS & STOPPING CRITERIA

The one-sided proportion test will be conducted for ORR against the null hypothesis that the ORR is less than or equal to 3%, with significance level of 0.05. The cumulative frequency, estimated proportion, and the exact 95% confidence intervals will be reported. A sample size of 48 achieves 87% power if the ORR is 15% using a one-sided exact test with a target significance level of 0.05. These results assume that the ORR of the historical control is 3%.

For alpha = 0.025 (1-sided) and power of 87%, MinMax design supports stopping enrollment for futility as noted if there are no responses (partial response or complete response as defined in the protocol) in first 24 subjects. Otherwise, full study enrollment will proceed as planned. Using a Simon-like 2-stage design, ORR will be determined based on the first 24 evaluable patients (including patients in both Stage 1 and Stage 2). If there are no responses (partial response or complete response as defined in the protocol) in the first 24 evaluable patients, then study enrollment will be stopped for futility. If there is at least one response (partial response or complete response as defined in the protocol) in the first 24 evaluable patients then full study enrollment will proceed as planned. If there are no responses (partial response or complete response as defined in the protocol) in the first 23 evaluable patients then enrollment will be temporarily paused to allow response evaluation of the 24th evaluable patient after up to 15 total weeks (ie 5 total cycles) of study therapy. If the 24th patient has response (partial response or complete response as defined in the protocol) then then full study enrollment will proceed as planned. If the 24th patient does not have a response (partial response or complete response as defined in the protocol) then the study will be stopped for futility. See Section 9.4.2 for statistical considerations.

9.4.3 ANALYSIS OF THE SECONDARY EFFICACY HYPOTHESES

The one-sided proportion test will be conducted for DCR against the null hypothesis that the DCR is less than or equal to 45%, with significance level of 0.05. The cumulative frequency, estimated proportion, and the exact 95% confidence intervals will be reported. A sample size of 48 achieves 87% power if the DCR is 65% using a one-sided exact test with a target significance level of 0.05. These results assume that the DCR of the historical control is 45%.

The one-sided, one sample log rank test will be conducted for PFS against the null hypothesis assuming that the hazard ratio of progression to the historical control is 1 while assuming the progression free survival probability at 6 months of the historical control is 20%. The survival probability (Kaplan-Meier survival curve) at 6 months will be calculated by the Kaplan-Meier method; the survival curve and the median survival time will be reported with the corresponding 2-sided 95% Brookmeyer-Crowley's Confidence Interval if feasible. A one-sided, one-sample log-rank test calculated from a sample of 48 subjects achieves 95.6% power at a 0.05 significance level to detect a 6-month progression free survival probability of 40% in the treatment group when the 6-month progression free probability in the historic control group is 20%. Assuming that subjects are accrued for 1 year and follow-up continues for 6 months after the last subject is added, the probability that a subject progress during the study is 0.82. The expected number of progression events during the study is 39. It is assumed that the survival time distributions of both groups are approximated reasonably well by the Weibull distribution with a shape parameter of 1.

DOR power calculations were not performed and DOR was not used to determine sample size. DOR will be compared to historical controls if sample size allows.

OS power calculations were not performed and OS was not used to determine sample size. OS will be compared to historical controls if sample size allows.

9.4.4 SAFETY ANALYSES

Safety will be assessed through summaries of adverse events. Adverse events will include changes in symptoms, changes in medical conditions, changes in vital signs, and changes in laboratory values. Additionally, separate summaries will be created which will include only serious adverse events (SAEs). When subjects experience multiple events of varying severity, the highest grade will be used in the subject-level summaries. Toxicity will be summarized and tabulated based on organ and severity.

9.4.5 BASELINE DESCRIPTIVE STATISTICS

The demographic characteristics of the participants will be presented with descriptive statistics. The means, medians, and 95% confidence intervals will be calculated for continuous variables; the frequencies, estimated rates, and the exact 95% confidence intervals will be calculated for binary and categorical variables.

9.4.6 PLANNED INTERIM ANALYSES

An interim efficacy analysis will be performed based on the first 24 evaluable patients as detailed in Section 9.4.2.

9.4.7 TABULATION OF INDIVIDUAL PARTICIPANT DATA

Tabulation data sets will be created which provide a tabular listing of subject data for all domains (e.g. demographics, outcomes, adverse events, etc).

9.4.8 EXPLORATORY ANALYSES

Analyses of ORR, DCR, DOR, and PFS per iRECIST compared to historical controls will be performed in a similar manner as described in Sections 9.4.2 and 9.4.3.

The change in pre- and post-treatment immune cells in the tumor microenvironment as measured by nine-color multispectral imaging will be evaluated using a paired t-test. The pre-treatment means, post-treatment means, mean difference between the pre- and post-treatment tumor infiltrating scores, and the 95% confidence intervals will be reported. A sample size of 18 achieves 81% power to detect a mean of paired differences of 5% with an estimated standard deviation of differences of 7% with a significance level (alpha) of 0.05 using a two-sided paired t-test. To account for non-evaluable biopsy specimens or patient dropout, the size of the Cohort A will be 23.

Additional exploratory analyses will be performed as sample size allows.

10 SUPPORTING DOCUMENTATION AND OPERATIONAL CONSIDERATIONS

10.1 REGULATORY, ETHICAL, AND STUDY OVERSIGHT CONSIDERATIONS

10.1.1 INFORMED CONSENT PROCESS

The Consent Forms must be signed and dated by the patient or the patient's legally authorized representative before his or her participation in the study. The case history or clinical records for each patient shall document the informed consent process and that written informed consent was obtained prior to participation in the study.

The Consent Forms should be revised whenever there are changes to study procedures or when new information becomes available that may affect the willingness of the patient to participate. The final revised IRB/EC-approved Consent Forms must be provided to AGICC for review.

Patients must be re-consented to the most current version of the Consent Forms (or to a significant new information/findings addendum in accordance with applicable laws and IRB/EC policy) during their participation in the study. For any updated or revised Consent Forms, the case history or clinical records for each patient shall document the informed consent process and that written informed consent was obtained using the updated/revised Consent Forms for continued participation in the study.

A copy of each signed Consent Form must be provided to the patient or the patient's legally authorized representative. All signed and dated Consent Forms must remain in each patient's study file or in the site file and must be available for verification by study monitors at any time.

Consent Form may also include patient authorization to allow use and disclosure of personal health information in compliance with the U.S. Health Insurance Portability and Accountability Act (HIPAA) of 1996. If the site utilizes a separate Authorization Form for patient authorization for use and disclosure of personal health information under the HIPAA regulations, the review, approval, and other processes outlined above apply except that IRB review and approval may not be required per study site policies.

10.1.1.1 CONSENT/ASSENT AND OTHER INFORMATIONAL DOCUMENTS PROVIDED TO PARTICIPANTS

Consent forms describing in detail the study agent, study procedures, and risks are given to the participant and written documentation of informed consent is required prior to starting intervention/ administering study product.

10.1.1.2 CONSENT PROCEDURES AND DOCUMENTATION

Informed consent process will be initiated prior to the individual's agreeing to participate in the study and continues throughout the individual's study participation. Extensive discussion of risks and possible benefits of participation will be provided to the participants and their families.

Consent forms will be IRB-approved and the participant will be asked to read and review the document. The investigator will explain the research study to the participant and answer any questions that may arise. All participants will receive a verbal explanation in terms suited to their comprehension of the purposes, procedures, and potential risks of the study and of their rights as research participants. Participants will have the opportunity to carefully review the written consent form and ask questions prior to signing. The participants will have the opportunity to discuss the study with their surrogates or think about it prior to agreeing to

participate. The participant will sign the informed consent document prior to any procedures being done specifically for the study.

The participants may withdraw consent at any time throughout the course of the trial. A copy of the informed consent document will be given to the participants for their records. The rights and welfare of the participants will be protected by emphasizing to them that the quality of their medical care will not be adversely affected if they decline to participate in this study.

The study allows the inclusion of non-English speaking and non-reading participants. Witnesses to these consent processes will be individuals not associated with the trial and will not have a conflict of interest.

10.1.2 STUDY DISCONTINUATION AND CLOSURE

This study may be temporarily suspended or prematurely terminated if there is sufficient reasonable cause. Written notification, documenting the reason for study suspension or termination, will be provided by the suspending, or terminating party to the study including the Lead PI, ALX Oncology Ltd, Merck & Co, Inc., & Eli Lilly USA, LLC, AGICC (the IND sponsor) and regulatory authorities. If the study is prematurely terminated or suspended, the study's Lead PI will promptly inform AGICC and the IRB providing the reason(s) for the termination or suspension. AGICC will update the IND with this information.

Circumstances that may warrant termination or suspension include, but are not limited to:

- Determination of unexpected, significant, or unacceptable risk to participants.
- Demonstration of efficacy that would warrant stopping.
- Insufficient compliance to protocol requirements.
- Data that are not sufficiently complete and/ or evaluable.
- Determination of futility.

The study may resume once concerns about safety, protocol compliance, and data quality are addressed and satisfy AGICC, the IRB, the DSMC, and/or the FDA.

10.1.3 CONFIDENTIALITY AND PRIVACY

Participant confidentiality is strictly held in trust by the participating investigators, their staff, and AGICC and their agents. This confidentiality is extended to cover testing of biological samples and genetic tests in addition to the clinical information relating to participants. Therefore, the study protocol, documentation, data, and all other information generated will be

held in strict confidence. No information concerning the study or the data will be released to any unauthorized third party without prior written approval of AGICC.

The study monitor, other authorized representatives of AGICC, representatives of the IRB or pharmaceutical companies supplying study product and health authority representatives may inspect all documents and records required to be maintained by the investigator, including but not limited to, medical records (office, clinic, or hospital) and pharmacy records for the participants in this study. The clinical study site will permit access to such records.

The study participant's contact information will be securely stored at each clinical site for internal use during the study. At the end of the study, all records will continue to be kept in a secure location for as long a period as dictated by local IRB and Institutional regulations.

Study participant research data and samples, which is for purposes of statistical analysis and scientific reporting, will be transmitted to and stored at the University of Colorado Cancer Center (samples) and AGICC (data). This will not include the participant's contact or identifying information. Rather, individual participants and their research data will be identified by a unique study identification number. The study data entry and study management systems used by clinical sites, the University of Colorado Cancer Center research staff, and AGICC will be secured and password protected. Data collected for this study will be analyzed by the study team including the Lead PI and a biostatistician(s).

During the study, these criteria regarding specimens and data specimens will be followed:

- **Intended Use:** Samples and data collected under this protocol may be used to study tumor-associated immune cells, peripheral blood immune cells, ctDNA, and biomarkers related to CRC to study the mechanism of action of study drugs. No germline genetic testing will be performed.
- **Storage:** During the study, blood and tissue samples will be stored at the University of Colorado Cancer Center. Access to stored samples will be limited to research personnel only, including ALX Oncology Ltd (to perform PK, ADA, and/or PD analyses). Samples and data will be stored using codes assigned by the investigators. Data will be kept in password-protected computers. Only investigators will have access to the samples and data.

At the end of the study, all study databases will be de-identified and archived at AGICC. Any remaining samples after analysis will be destroyed unless there is optional participant consent for banking. The future use of stored/banked specimens or data is described in Section 10.1.4.

This study will ensure that the public has access to the results of this research, via presentations at meetings, publication, and ClinicalTrials.gov.

10.1.4 FUTURE USE OF STORED SPECIMENS OR DATA

Any remaining samples or data after analysis will be destroyed unless there is optional participant consent for banking (specimens and data). During the conduct of the study, an individual participant can choose to withdraw consent to have de-identified biological specimens and data stored for future research. In such instances, the participant will be notified of compliance with such request and all supporting details will be maintained for tracking. However, withdrawal of consent with regard to biosample storage will not be possible after the study is completed.

After the study is complete, if study participants provide optional consent for banking of specimens and data, de-identified archived data and specimens will be stored at the University of Colorado Biorepository, under the supervision of the Director. These data and specimens may be used by other researchers including those outside of the study, with the oversight of appropriate regulatory processes (such as the local IRB), for the study of cancer, its complications, and other conditions for which individuals with cancer are at increased risk, and to improve treatment. Access to data and/or samples under these guidelines will be provided through the University of Colorado Biorepository.

10.1.5 SAFETY OVERSIGHT

PHASE II Monitoring and Oversight

AGICC will be responsible for monitoring the trial per the trial monitoring plan, in addition to overseeing the safety and efficacy of the trial including any specimens collected, executing the data and safety monitoring (DSM) plan, and complying with all reporting requirements to local and federal authorities. This oversight will be accomplished through additional oversight from the AGICC Data and Safety Monitoring Committee (DSMC) according to the DMC Charter. The DMC Charter describes roles and responsibilities of the DSMC for this study protocol and outlines the plan for implementing these roles and for disseminating study data.

The DSMC is primarily responsible for ensuring data quality and study participant safety for the clinical studies to which they provide such oversight. The DSMC shares responsibility with AGICC for regularly monitoring the overall safety of the subjects in this clinical study.

A summary of the DSMC’s activities is as follows:

- Ongoing review of all serious adverse events (SAEs) and unanticipated problems (UAPs)
- Has the authority to suspend trials for safety or trial conduct issues
- May submit recommendations for corrective actions

Per the study's DSM Plan, SAEs and UAPs are reported to the DSMC, IRB and AGICC per this protocol. All SAEs and UAPs are to be reported to the DSMC within 7 (for fatal or life-threatening events) or 15 (non-life-threatening events) calendar days of AGICC receiving notification of the occurrence.

Data regarding number of subjects, significant toxicities, dose modifications, and treatment responses will be discussed and documented in the meeting's minutes.

AGICC is responsible for organizing and conducting regularly scheduled teleconferences with all participating sites. AGICC will also be responsible for including data from all the participating sites to include the minutes from these regularly scheduled teleconferences between AGICC and the sites within the overall trial's DSM progress report.

10.1.6 CLINICAL MONITORING

Clinical site monitoring will be conducted to ensure that the rights and well-being of human participants are protected, that the reported trial data are accurate, complete, and verifiable, and that the conduct of the trial is in compliance with the currently approved protocol/ amendment(s), with GCP, and with applicable regulatory requirement(s).

Monitoring for this study will be performed by a AGICC or authorized representative in accordance with the clinical study monitoring plan (AGICC MP), incorporated herein by reference. The AGICC MP describes in detail who will conduct the monitoring, at what frequency monitoring will be done, at what level of detail monitoring will be performed, and the distribution of the monitoring reports.

10.1.7 QUALITY ASSURANCE AND QUALITY CONTROL

Quality Control (QC) procedures will be implemented beginning with the data entry system and data QC checks that will be run on the database will be generated. Any missing data or data anomalies will be communicated to the site(s) for clarification/ resolution.

Following written standard operating procedures (SOPs), the study monitor will verify that the clinical trial is conducted and data are generated, documented (recorded), and reported in compliance with the protocol, GCP, and the applicable regulatory requirements (e.g., Good Laboratory Practices (GLP), Good Manufacturing Practices (GMP)).

The investigational site will provide direct or remote access to all trial-related sites, source data/ documents, and reports for the purpose of monitoring and auditing by AGICC's authorized audit team, and inspection by local and regulatory authorities.

Site monitoring visits will be performed by AGICC's authorized representative on a regular basis, pursuant to the AGICC MP. During these visits, information recorded on the eCRFs will be verified against source documents. Additional computer programs that identify selected protocol deviations, out-of-range data, and other data errors within the electronic data entry may also be used to help monitor the study. As necessary, requests for data clarification or correction will be sent to the appropriate site PI. Independent auditors from AGICC's authorized representatives will be allowed by the site's PI to audit. In addition, audits may be conducted at any time by appropriate regulatory authorities and/or the IRB.

10.1.8 DATA HANDLING AND RECORD KEEPING

10.1.8.1 DATA COLLECTION AND MANAGEMENT RESPONSIBILITIES

Data collection is the responsibility of the clinical trial staff at the site under the supervision of the site PI. The PI is responsible for ensuring the accuracy, completeness, legibility, and timeliness of the data reported.

All source documents should be completed in a neat, legible manner to ensure accurate interpretation of data. When making changes or corrections, cross out the original entry with a single line, and initial and date the change. DO NOT ERASE, OVERWRITE, OR USE CORRECTION FLUID OR TAPE ON THE ORIGINAL.

Data for each participant enrolled in the study will be recorded on the CRFs within the electronic data capture system (EDC). Data reported in the eCRF derived from source documents should be consistent with the source documents or the discrepancies should be explained and captured in a progress note and maintained in the participant's official electronic study record.

Clinical data (including AEs, concomitant medications, and expected adverse reactions data) and clinical laboratory data will be entered into a 21 CFR Part 11-compliant electronic data capture system provided by AGICC. The data system includes password protection and internal quality checks, such as automatic range checks, to identify data that appear inconsistent, incomplete, or inaccurate. Clinical data will be entered directly from the source documents.

10.1.8.2 STUDY RECORDS RETENTION

Study documents should be retained for a minimum of 2 years after the last approval of an investigational marketing application and until there are no pending or contemplated marketing applications or until at least 2 years have elapsed since the formal discontinuation of clinical development of the investigational product. These documents should be retained for a longer period, however, if required by local regulations or institutional policies. No records will be destroyed without the written consent of AGICC, if applicable. It is the responsibility of AGICC to inform the PI when these documents no longer need to be retained.

10.1.9 PROTOCOL DEVIATIONS

A protocol deviation is any noncompliance with the clinical trial protocol, GCP, or SOP requirements. The noncompliance may be either on the part of the participant, the investigator, or the study site staff. As a result of deviations, corrective actions are to be developed by the site and implemented promptly. These practices are consistent with ICH E6, sections:

- 4.5 Compliance with Protocol, sections 4.5.1, 4.5.2, and 4.5.3.
- 5.1 Quality Assurance and Quality Control, section 5.1.1.
- 5.20 Noncompliance, sections 5.20.1 and 5.20.2.

It is the responsibility of the study team to use continuous vigilance to identify and report deviations. All deviations must be addressed in study source documents and reported appropriately as required by AGICC, the DSMC, the study agent manufacturers, and the site's local policies. Protocol deviations must be sent to the local IRB per institutional guidelines. The site PI/study staff is responsible for knowing and adhering to their IRB requirements. Further details about the handling of protocol deviations will be included in the SOP.

Protocol deviations due to COVID-19 should be identified and reported in the same manner. Decisions regarding study events, treatments, and patient safety should be discussed with the medical monitor.

10.1.10 PUBLICATION AND DATA SHARING POLICY

This study will ensure that the public has access to the published results of this research.

As required, either for publication (the ICMJE or other publication policy) or according to U.S. regulations (Section 801 of the Food and Drug Administration Amendments Act of 2007) this clinical trial will be registered in a public trials registry including ClinicalTrials.gov, which is

sponsored by the National Library of Medicine, and the NCI CTRP Registry for cancer clinical trials.

10.1.11 CONFLICT OF INTEREST POLICY

Independence of this study from any actual or perceived influence, such as by the pharmaceutical industry, is critical. Any actual conflict of interest of persons who have a role in the design, conduct, analysis, publication, or any aspect of this trial will be disclosed to AGICC and managed by the participating site's institutional program or department that manages conflict of interest matters. Persons with a perceived conflict of interest will have such conflicts managed in a way that is appropriate to their participation in the trial. Conflict of Interest management plans are project-specific and should be reviewed at least annually.

11 REFERENCES

1. Bray F, Ferlay J, Soerjomataram I, Siegel RL, Torre LA, Jemal A. Global cancer statistics 2018: GLOBOCAN estimates of incidence and mortality worldwide for 36 cancers in 185 countries. *CA Cancer J Clin.* 2018;68(6):394-424.

2. Siegel RL, Miller KD, Jemal A. Cancer statistics, 2020. *CA Cancer J Clin.* 2020;70(1):7-30.

3. Dekker E, Tanis PJ, Vleugels JLA, Kasi PM, Wallace MB. Colorectal cancer. *Lancet.* 2019;394(10207):1467-1480.

4. Karapetis CS, Khambata-Ford S, Jonker DJ, et al. K-ras mutations and benefit from cetuximab in advanced colorectal cancer. *N Engl J Med.* 2008;359(17):1757-1765.

5. Amado RG, Wolf M, Peeters M, et al. Wild-type KRAS is required for panitumumab efficacy in patients with metastatic colorectal cancer. *J Clin Oncol.* 2008;26(10):1626-1634.

6. Douillard JY, Oliner KS, Siena S, et al. Panitumumab-FOLFOX4 treatment and RAS mutations in colorectal cancer. *N Engl J Med.* 2013;369(11):1023-1034.

7. Pietrantonio F, Petrelli F, Coinu A, et al. Predictive role of BRAF mutations in patients with advanced colorectal cancer receiving cetuximab and panitumumab: a meta-analysis. *Eur J Cancer.* 2015;51(5):587-594.

8. Tejpar S, Stintzing S, Ciardiello F, et al. Prognostic and Predictive Relevance of Primary Tumor Location in Patients With RAS Wild-Type Metastatic Colorectal Cancer: Retrospective Analyses of the CRYSTAL and FIRE-3 Trials. *JAMA Oncol.* 2017;3(2):194-201.

9. Holch JW, Ricard I, Stintzing S, Modest DP, Heinemann V. The relevance of primary tumour location in patients with metastatic colorectal cancer: A meta-analysis of first-line clinical trials. *Eur J Cancer.* 2017;70:87-98.

10. Grothey A, Van Cutsem E, Sobrero A, et al. Regorafenib monotherapy for previously treated metastatic colorectal cancer (CORRECT): an international, multicentre, randomised, placebo-controlled, phase 3 trial. *Lancet.* 2013;381(9863):303-312.

11. Li J, Qin S, Xu R, et al. Regorafenib plus best supportive care versus placebo plus best supportive care in Asian patients with previously treated metastatic colorectal cancer (CONCUR): a randomised, double-blind, placebo-controlled, phase 3 trial. *Lancet Oncol.* 2015;16(6):619-629.

12. Mayer RJ, Van Cutsem E, Falcone A, et al. Randomized trial of TAS-102 for refractory metastatic colorectal cancer. *N Engl J Med.* 2015;372(20):1909-1919.

13. Xu J, Kim TW, Shen L, et al. Results of a Randomized, Double-Blind, Placebo-Controlled, Phase III Trial of Trifluridine/Tipiracil (TAS-102) Monotherapy in Asian Patients With Previously Treated Metastatic Colorectal Cancer: The TERRA Study. *J Clin Oncol.* 2018;36(4):350-358.

14. Le DT, Uram JN, Wang H, et al. PD-1 Blockade in Tumors with Mismatch-Repair Deficiency. *N Engl J Med.* 2015;372(26):2509-2520.

15. Overman MJ, McDermott R, Leach JL, et al. Nivolumab in patients with metastatic DNA mismatch repair-deficient or microsatellite instability-high colorectal cancer (CheckMate 142): an open-label, multicentre, phase 2 study. *Lancet Oncol.* 2017;18(9):1182-1191.

16. Oh DY, Venook AP, Fong L. On the Verge: Immunotherapy for Colorectal Carcinoma. *J Natl Compr Canc Netw.* 2015;13(8):970-978.

17. Togashi Y, Shitara K, Nishikawa H. Regulatory T cells in cancer immunosuppression - implications for anticancer therapy. *Nat Rev Clin Oncol.* 2019;16(6):356-371.

18. Kamada T, Togashi Y, Tay C, et al. PD-1(+) regulatory T cells amplified by PD-1 blockade promote hyperprogression of cancer. *Proc Natl Acad Sci U S A.* 2019;116(20):9999-10008.

19. Wei SC, Duffy CR, Allison JP. Fundamental Mechanisms of Immune Checkpoint Blockade Therapy. *Cancer Discov.* 2018;8(9):1069-1086.

20. Xie YH, Chen YX, Fang JY. Comprehensive review of targeted therapy for colorectal cancer. *Signal Transduct Target Ther.* 2020;5(1):22.

21. Chen DS, Mellman I. Oncology meets immunology: the cancer-immunity cycle. *Immunity.* 2013;39(1):1-10.

22. Wilky BA. Immune checkpoint inhibitors: The linchpins of modern immunotherapy. *Immunol Rev.* 2019;290(1):6-23.

23. Iwai Y, Ishida M, Tanaka Y, Okazaki T, Honjo T, Minato N. Involvement of PD-L1 on tumor cells in the escape from host immune system and tumor immunotherapy by PD-L1 blockade. *Proc Natl Acad Sci U S A.* 2002;99(19):12293-12297.

24. Wainberg ZA, Diamond JR, Curigliano G, et al. First-line durvalumab + monalizumab, mFOLFOX6, and bevacizumab or cetuximab for metastatic microsatellite-stable colorectal cancer (MSS-CRC). *Journal of Clinical Oncology.* 2020;38(4_suppl):128-128.

25. McDermott DF, Atkins MB. PD-1 as a potential target in cancer therapy. *Cancer Med.* 2013;2(5):662-673.

26. Hutchinson RA, Adams RA, McArt DG, Salto-Tellez M, Jasani B, Hamilton PW. Epidermal growth factor receptor immunohistochemistry: new opportunities in metastatic colorectal cancer. *J Transl Med.* 2015;13:217.

27. Vecchione L, Jacobs B, Normanno N, Ciardiello F, Tejpar S. EGFR-targeted therapy. *Exp Cell Res.* 2011;317(19):2765-2771.

28. Mendelsohn J, Prewett M, Rockwell P, Goldstein NI. CCR 20th anniversary commentary: a chimeric antibody, C225, inhibits EGFR activation and tumor growth. *Clin Cancer Res.* 2015;21(2):227-229.

29. Cunningham D, Humblet Y, Siena S, et al. Cetuximab monotherapy and cetuximab plus irinotecan in irinotecan-refractory metastatic colorectal cancer. *N Engl J Med.* 2004;351(4):337-345.

30. Jonker DJ, O'Callaghan CJ, Karapetis CS, et al. Cetuximab for the treatment of colorectal cancer. *N Engl J Med.* 2007;357(20):2040-2048.

31. Sobrero AF, Maurel J, Fehrenbacher L, et al. EPIC: phase III trial of cetuximab plus irinotecan after fluoropyrimidine and oxaliplatin failure in patients with metastatic colorectal cancer. *J Clin Oncol.* 2008;26(14):2311-2319.

32. Van Cutsem E, Kohne CH, Hitre E, et al. Cetuximab and chemotherapy as initial treatment for metastatic colorectal cancer. *N Engl J Med.* 2009;360(14):1408-1417.

33. Bokemeyer C, Bondarenko I, Hartmann JT, et al. Efficacy according to biomarker status of cetuximab plus FOLFOX-4 as first-line treatment for metastatic colorectal cancer: the OPUS study. *Ann Oncol.* 2011;22(7):1535-1546.

34. Qin S, Li J, Wang L, et al. Efficacy and Tolerability of First-Line Cetuximab Plus Leucovorin, Fluorouracil, and Oxaliplatin (FOLFOX-4) Versus FOLFOX-4 in Patients With RAS Wild-Type Metastatic Colorectal Cancer: The Open-Label, Randomized, Phase III TAILOR Trial. *J Clin Oncol.* 2018;36(30):3031-3039.

35. Peeters M, Kafatos G, Taylor A, et al. Prevalence of RAS mutations and individual variation patterns among patients with metastatic colorectal cancer: A pooled analysis of randomised controlled trials. *Eur J Cancer.* 2015;51(13):1704-1713.

36. Venderbosch S, Nagtegaal ID, Maughan TS, et al. Mismatch repair status and BRAF mutation status in metastatic colorectal cancer patients: a pooled analysis of the CAIRO, CAIRO2, COIN, and FOCUS studies. *Clin Cancer Res.* 2014;20(20):5322-5330.

37. Chu JE, Johnson B, Kugathasan L, et al. Population-based Screening for BRAF (V600E) in Metastatic Colorectal Cancer Reveals Increased Prevalence and Poor Prognosis. *Clin Cancer Res.* 2020;26(17):4599-4605.

38. Arnold D, Lueza B, Douillard JY, et al. Prognostic and predictive value of primary tumour side in patients with RAS wild-type metastatic colorectal cancer treated with chemotherapy and EGFR directed antibodies in six randomized trials. *Ann Oncol.* 2017;28(8):1713-1729.

39. Vidarsson G, Dekkers G, Rispens T. IgG subclasses and allotypes: from structure to effector functions. *Front Immunol.* 2014;5:520.

40. Gul N, van Egmond M. Antibody-Dependent Phagocytosis of Tumor Cells by Macrophages: A Potent Effector Mechanism of Monoclonal Antibody Therapy of Cancer. *Cancer Res.* 2015;75(23):5008-5013.

41. Ashraf SQ, Nicholls AM, Wilding JL, Ntouroupi TG, Mortensen NJ, Bodmer WF. Direct and immune mediated antibody targeting of ERBB receptors in a colorectal cancer cell-line panel. *Proc Natl Acad Sci U S A.* 2012;109(51):21046-21051.

42. Cohen RB. Epidermal growth factor receptor as a therapeutic target in colorectal cancer. *Clin Colorectal Cancer.* 2003;2(4):246-251.

43. Maughan TS, Adams RA, Smith CG, et al. Addition of cetuximab to oxaliplatin-based first-line combination chemotherapy for treatment of advanced colorectal cancer: results of the randomised phase 3 MRC COIN trial. *Lancet.* 2011;377(9783):2103-2114.

44. Chung KY, Shia J, Kemeny NE, et al. Cetuximab shows activity in colorectal cancer patients with tumors that do not express the epidermal growth factor receptor by immunohistochemistry. *J Clin Oncol.* 2005;23(9):1803-1810.

45. Benson AB, Venook AP, Al-Haway MM, et al. NCCN Guidelines Insights: Colon Cancer, Version 2.2018. *J Natl Compr Canc Netw.* 2018;16(4):359-369.

46. Weiskopf K. Cancer immunotherapy targeting the CD47/SIRPalpha axis. *Eur J Cancer.* 2017;76:100-109.

47. Demaria O, Cornen S, Daeron M, Morel Y, Medzhitov R, Vivier E. Harnessing innate immunity in cancer therapy. *Nature.* 2019;574(7776):45-56.

48. Feng M, Jiang W, Kim BYS, Zhang CC, Fu YX, Weissman IL. Phagocytosis checkpoints as new targets for cancer immunotherapy. *Nat Rev Cancer.* 2019;19(10):568-586.

49. Willingham SB, Volkmer JP, Gentles AJ, et al. The CD47-signal regulatory protein alpha (SIRPa) interaction is a therapeutic target for human solid tumors. *Proc Natl Acad Sci U S A.* 2012;109(17):6662-6667.

50. Advani R, Flinn I, Popplewell L, et al. CD47 Blockade by Hu5F9-G4 and Rituximab in Non-Hodgkin's Lymphoma. *N Engl J Med.* 2018;379(18):1711-1721.

51. Sikic BI, Lakhani N, Patnaik A, et al. First-in-Human, First-in-Class Phase I Trial of the Anti-CD47 Antibody Hu5F9-G4 in Patients With Advanced Cancers. *J Clin Oncol.* 2019;37(12):946-953.

52. Kauder SE, Kuo TC, Harrabi O, et al. ALX148 blocks CD47 and enhances innate and adaptive antitumor immunity with a favorable safety profile. *PLoS One.* 2018;13(8):e0201832.

53. Chow LQM, Gainor JF, Lakhani NJ, et al. A phase I study of ALX148, a CD47 blocker, in combination with established anticancer antibodies in patients with advanced malignancy. *Journal of Clinical Oncology.* 2019;37(15_suppl):2514-2514.

54. Chow LQM, Gainor JF, Lakhani NJ, et al. A phase I study of ALX148, a CD47 blocker, in combination with standard anticancer antibodies and chemotherapy regimens in patients with advanced malignancy. *Journal of Clinical Oncology.* 2020;38(15_suppl):3056-3056.

55. Nehal JL, Patricia L, Navid H, et al. A phase 1 study of ALX148, a CD47 blocker, alone and in combination with established anticancer antibodies in patients with advanced malignancy and non-Hodgkin lymphoma. *Journal of Clinical Oncology.* 2018;36(15_suppl):3068-3068.

56. Chow LQM, Gainor JF, Lakhani NJ, et al. A phase I study of ALX148, a CD47 blocker, in combination with established anticancer antibodies in patients with advanced malignancy. *Journal of Clinical Oncology.* 2019;37(15_suppl):2514-2514.

57. Pembrolizumab FDA Prescribing Information. *https://www.accessdata.fda.gov/drugsatfda_docs/label/2020/125514s066lbl.pdf*. Accessed Oct 26, 2020.

58. Saltz LB, Meropol NJ, Loehrer PJ, Sr., Needle MN, Kopit J, Mayer RJ. Phase II trial of cetuximab in patients with refractory colorectal cancer that expresses the epidermal growth factor receptor. *J Clin Oncol.* 2004;22(7):1201-1208.

59. Wan H, Chow, L., Gainor, J., Lakhani, N., Chung, H., Lee, K., Lee, J., LoRusso, P., Bang, Y., Hodi, S., Messersmith, W., Fanning, P., Squifflet, P., Jin, F., Kuo, T., Bollini, S., Pons, J., Randolph, S. Pharmacodynamic biomarker characterization of ALX148, a CD47 blocker, in combination with established anticancer antibodies in patients with advanced malignancy. *Poster presented at Society for Immunotherpay of Cancer Annual Meeting.* 2019.

60. Tentler JJ, Tan AC, Weekes CD, et al. Patient-derived tumour xenografts as models for oncology drug development. *Nat Rev Clin Oncol.* 2012;9(6):338-350.

61. Cetuximab FDA Prescribing Information. *https://www.accessdata.fda.gov/drugsatfda_docs/label/2019/125084s273lbl.pdf*. Accessed Nov 19, 2020.

62. ALX Oncology. ALX148 - Investigator's Brochure. 2022.

63. Van Cutsem E, Peeters M, Siena S, et al. Open-label phase III trial of panitumumab plus best supportive care compared with best supportive care alone in patients with chemotherapy-refractory metastatic colorectal cancer. *J Clin Oncol.* 2007;25(13):1658-1664.

64. Peeters M, Price TJ, Cervantes A, et al. Final results from a randomized phase 3 study of FOLFIRI {+/-} panitumumab for second-line treatment of metastatic colorectal cancer. *Ann Oncol.* 2014;25(1):107-116.

65. Douillard JY, Siena S, Cassidy J, et al. Randomized, phase III trial of panitumumab with infusional fluorouracil, leucovorin, and oxaliplatin (FOLFOX4) versus FOLFOX4 alone as first-line treatment in patients with previously untreated metastatic colorectal cancer: the PRIME study. *J Clin Oncol.* 2010;28(31):4697-4705.

66. Moretto R, Cremolini C, Rossini D, et al. Location of Primary Tumor and Benefit From Anti-Epidermal Growth Factor Receptor Monoclonal Antibodies in Patients With RAS and BRAF Wild-Type Metastatic Colorectal Cancer. *Oncologist.* 2016;21(8):988-994.

67. Kim TM, Lakhani N, Gainor J, et al. ALX148, a CD47 Blocker, in Combination with Rituximab in Patients with Relapsed/Refractory (R/R) Non-Hodgkin Lymphoma. *Poster presented at European Hematology Association Annual Congress.* 2020.

68. Martinelli E, Martini G, Troiani T, et al. 397O Avelumab plus cetuximab in pre-treated RAS wild type metastatic colorectal cancer patients as a rechallenge strategy: The phase II CAVE (cetuximab-avelumab) mCRC study. *Annals of Oncology.* 2020;31:S409-S410.

69. Chapuy CI, Nicholson RT, Aguad MD, et al. Resolving the daratumumab interference with blood compatibility testing. *Transfusion.* 2015;55(6 Pt 2):1545-1554.

70. Velliquette RW, Aeschlimann J, Kirkegaard J, Shakarian G, Lomas-Francis C, Westhoff CM. Monoclonal anti-CD47 interference in red cell and platelet testing. *Transfusion.* 2019;59(2):730-737.

71. Brahmer JR, Lacchetti C, Schneider BJ, et al. Management of Immune-Related Adverse Events in Patients Treated With Immune Checkpoint Inhibitor Therapy: American Society of Clinical Oncology Clinical Practice Guideline. *J Clin Oncol.* 2018;36(17):1714-1768.

72. Lacouture ME, Mitchell EP, Piperdi B, et al. Skin toxicity evaluation protocol with panitumumab (STEPP), a phase II, open-label, randomized trial evaluating the impact of a pre-Emptive Skin treatment regimen on skin toxicities and quality of life in patients with metastatic colorectal cancer. *J Clin Oncol.* 2010;28(8):1351-1357.

73. Bergsten E, Horne A, Arico M, et al. Confirmed efficacy of etoposide and dexamethasone in HLH treatment: long-term results of the cooperative HLH-2004 study. *Blood.* 2017;130(25):2728-2738.

74. Trottestam H, Horne A, Arico M, et al. Chemoimmunotherapy for hemophagocytic lymphohistiocytosis: long-term results of the HLH-94 treatment protocol. *Blood.* 2011;118(17):4577-4584.

75. Masood A, Wahab A, Clifford T, Weaver EJ, Ehsan H, El Ayass W. Secondary hemophagocytic lymphohistiocytosis due to nivolumab/ipilimumab in a renal cell cancer patient-A case report. *Clin Case Rep.* 2021;9(12):e05184.

76. Rubio-Perez J, Rodriguez-Perez AR, Diaz-Blazquez M, Moreno-Garcia V, Domine-Gomez M. Treatment-related hemophagocytic lymphohistiocytosis due to atezolizumab: a case report and review of the literature. *J Med Case Rep.* 2022;16(1):365.

77. Eisenhauer EA, Therasse P, Bogaerts J, et al. New response evaluation criteria in solid tumours: revised RECIST guideline (version 1.1). *Eur J Cancer.* 2009;45(2):228-247.

78. Seymour L, Bogaerts J, Perrone A, et al. iRECIST: guidelines for response criteria for use in trials testing immunotherapeutics. *Lancet Oncol.* 2017;18(3):e143-e152.

79. Oken MM, Creech RH, Tormey DC, et al. Toxicity and response criteria of the Eastern Cooperative Oncology Group. *Am J Clin Oncol.* 1982;5(6):649-655.

12 APPENDICES

12.1 APPENDIX 1: GUIDELINES FOR TOXICITY MANAGEMENT, INCLUDING DOSE MODIFICATIONS, DOSE INTERRUPTIONS, DOSE DELAYS, & DISCONTINUATION

12.1.1 GENERAL GUIDANCE

The adverse event severity grading scale for the NCI CTCAE (v5.0) will be used for assessing adverse event severity. The following table will be used for assessing severity for adverse events that are not specifically listed in the NCI CTCAE (v5.0) (also listed in Section 8.3.3.1).

Grade	Severity
1	Mild; asymptomatic or mild symptoms; clinical or diagnostic observations only; or intervention not indicated
2	Moderate; minimal, local, or non-invasive intervention indicated; or limiting age-appropriate instrumental activities of daily living [a]
3	Severe or medically significant, but not immediately life-threatening; hospitalization or prolongation of hospitalization indicated; disabling; or limiting self-care activities of daily living [b,c]
4	Life-threatening consequences or urgent intervention indicated
5	Death related to adverse event

NCI CTCAE: National Cancer Institute Common Terminology Criteria for Adverse Events. Note: Based on the most recent version of NCI CTCAE (v5.0), which can be found at: http://ctep.cancer.gov/protocolDevelopment/electronic_applications/ctc.htm

a. Instrumental activities of daily living refer to preparing meals, shopping for groceries or clothes, using the telephone, managing money, etc.

b. Examples of self-care activities of daily living include bathing, dressing and undressing, feeding oneself, using the toilet, and taking medications, as performed by patients who are not bedridden.

c. If an event is assessed as a "significant medical event," it must be reported as a serious adverse event.

AEs associated with pembrolizumab exposure, including coadministration with additional compounds, may represent an immunologic etiology. These immune-related AEs (irAEs) may occur shortly after the first dose or several months after the last dose of pembrolizumab/combination treatment and may affect more than one body system simultaneously. Therefore, early recognition and initiation of treatment is critical to reduce complications. Based on existing clinical study data, most irAEs were reversible and could be managed with interruptions of pembrolizumab/combination treatment, administration of corticosteroids and/or other supportive care. For suspected irAEs, ensure adequate evaluation to confirm etiology or exclude other causes. Additional procedures or tests such as bronchoscopy,

endoscopy, skin biopsy may be included as part of the evaluation. Dose modification and toxicity management guidelines for irAEs are provided in Section 12.1 subsections.

AEs should be managed with maximal supportive care per Investigator discretion and based on published guidelines, taking the most likely causative drug into account. In general, no specific dose adjustments or changes to the dose schedule are recommended for Grade 1/2 treatment-related toxicity with several exceptions noted in Sections 12.1.3 through 12.1.9. However, Investigators should always manage their patients according to their medical judgment based on the particular clinical circumstances and utilize the following guidelines.

When study interventions are administered in combination, attribution of an adverse event to a single or multiple compound(s) is likely to be difficult. Therefore, evorpacept (ALX148), cetuximab, **and** pembrolizumab should be interrupted for Grade ≥3 treatment-related toxicity, unless as otherwise described in Sections 12.1.3 through 12.1.11. Retreatment following treatment interruption for treatment-related toxicity or the start of a new cycle of treatment may begin only if (and as detailed in Sections 12.1.3 through 12.1.11):

- ANC ≥1,000/mm^3
- Platelet count ≥50,000/mm^3
- Evidence for Grade ≥2 hemolysis has returned to baseline or Grade ≤1 severity (or, at the Investigator discretion, Grade ≤2 if not considered a safety risk for the patient)
- Non-hematologic toxicities have returned to baseline or Grade ≤1 severity (or, at the Investigator discretion, Grade ≤2 if not considered a safety risk for the patient)

Sections 12.1.3 through 12.1.11 detail specific guidelines for the management of common AEs. Other AEs should be managed per published guidelines and Investigator discretion if clearly due to cetuximab or pembrolizumab.

General instructions for dose modification and toxicity management guidelines for immune-related AEs (additional details are provided in Sections 12.1.3 through 12.1.11):

- Severe and life-threatening irAEs should be treated with IV corticosteroids followed by oral steroids. Other immunosuppressive treatment should begin if the irAEs are not controlled by corticosteroids.
- Study intervention must be permanently discontinued if the irAE does not resolve or the corticosteroid dose is not ≤10 mg/day within 12 weeks of the last study intervention treatment.
- The corticosteroid taper should begin when the irAE is ≤ Grade 1 and continue at least 4 weeks.

- If pembrolizumab has been withheld, pembrolizumab may resume after the irAE decreases to ≤ Grade 1 and the patient is receiving ≤10 mg/day oral prednisone or equivalent
- Pembrolizumab must be permanently discontinued for ≥Grade 3 neurologic toxicity, ≥Grade 2 myocarditis, confirmed Stevens-Johnson Syndrome/Toxic Epidermal Necrolysis/Drug Rash with Eosinophilia and Systemic Symptoms, and other grade 4 or recurrent grade 3 immune-related adverse events.

Patients with grade 4 hypertension must not receive further treatment with cetuximab.

If the toxicity is unlikely due to cetuximab or pembrolizumab, there should be a low threshold to attribute it to evorpacept (ALX148) and the following guidelines should be used:

- Patients experiencing Grade 3 or 4 potentially treatment related toxicity or intolerable Grade 2 toxicity despite maximal supportive care should have their treatment (evorpacept (ALX148), cetuximab, **and** pembrolizumab) interrupted. Appropriate follow-up assessments should be performed until adequate recovery occurs as assessed by the Investigator.
- Treatment may be resumed when toxicities have returned to baseline or Grade ≤1 severity (or, at the Investigator discretion, Grade ≤2 if not considered a safety risk for the patient). A treatment delay of more than 14 days (beyond the next scheduled study drug infusion) due to lack of recovery will result in discontinuation of the patient from the treatment unless otherwise agreed between the Investigator AGICC.
- Upon resuming:
    - Evorpacept (ALX148) should be reduced by one dose level (Section 12.1.2)
    - Cetuximab and pembrolizumab can be continued at the same dose level (Section 12.1.2). Cetuximab dose may be reduced (Section 12.1.2) per Investigator discretion
    - If a pembrolizumab immune-mediated toxicity is suspected, guidelines for workup and management should be followed. If severe, pembrolizumab can be discontinued per investigator discretion (must discontinue if grade 4 or recurrent grade 3 and likely discontinue if grade 3)

If the AE that led to treatment interruption improves to allow treatment resumption/resolves, then re-dosing in that cycle is allowed. Doses omitted for toxicity are not replaced within the same cycle. If a treatment interruption continues beyond Day 21 of the current cycle, then the day when treatment is restarted will be counted as Day 1 of the next cycle. A treatment delay of more than 14 days (beyond the next scheduled study drug infusion) due to lack of recovery will result in discontinuation of the patient from the treatment unless otherwise agreed between the Investigator and AGICC.

Evorpacept (ALX148), cetuximab, and pembrolizumab may be interrupted for situations other than treatment-related AEs such as medical / surgical events and/or unforeseen circumstances not related to study intervention. However, intervention is to be restarted within 3 weeks of the originally scheduled dose, unless otherwise discussed with AGICC. The reason for study intervention interruption is to be documented in the patient's study record.

12.1.2 DOSE LEVELS

Evorpacept (ALX148)

- Dose level 1: 15 mg/kg weekly
- Dose level -1: 10 mg/kg weekly
- Dose level -2: 5 mg/kg weekly

Cetuximab

- Dose level 1: 250 mg/m^2 weekly (following 400 mg/m^2 loading dose)
- Dose level -1: 200 mg/m^2 weekly
- Dose level -2: 150 mg/m^2 weekly

Pembrolizumab

- Dose level 1: 200 mg every 3 weeks
- There are no additional dose levels

Once a dose has been reduced for a given patient, all subsequent cycles should be administered at that dose level, unless further dose reduction is required. Dose re-escalation is not allowed.

12.1.3 MANAGEMENT OF HEMATOLOGIC TOXICITY

Evorpacept (ALX148) may cause hematologic toxicity. Hematologic toxicity is uncommon with pembrolizumab and cetuximab.

Management of neutropenia and thrombocytopenia

Toxicity[a]	Hold treatment (evorpacept [ALX148], cetuximab, and pembrolizumab) until:[b]	Restart evorpacept (ALX148) treatment at:[c]	Restart cetuximab and pembrolizumab treatment at:

Uncomplicated Grade 3 neutropenia (ANC<1000/mm^3)	ANC ≥1,000/mm^3	Same dose level	Same dose levels (2nd occurrence: reduce cetuximab 1 dose level)
Grade 3 neutropenia (ANC<1000/mm^3) associated with a documented infection or fever > 38.55°C	ANC ≥1,000/mm^3 and infection is adequately treated	Decrease 1 dose level	Same dose levels (2nd occurrence: reduce cetuximab 1 dose level)
Grade 4 neutropenia (ANC<500/mm^3)	ANC ≥1,000/mm^3	Decrease 1 dose level	Same dose levels (2nd occurrence: reduce cetuximab 1 dose level)
Grade 4 thrombocytopenia (Platelet count <25,000/mm^3)	Platelets count ≥50,000/mm^3	Decrease 1 dose level	Same dose levels (2nd occurrence: reduce cetuximab 1 dose level)

a. Hematologic toxicities should be managed via supportive care (including transfusions) by Investigators' medical judgement.
b. A treatment delay of more than 14 days (beyond the next scheduled study drug infusion) due to lack of recovery will result in discontinuation of the patient from the treatment unless otherwise agreed between the Investigator and AGICC.
c. Dose reduction of evorpacept (ALX148) by 1 and, if needed, 2 dose levels will be allowed depending on the type and severity of toxicity encountered. Patients requiring more than 2 dose reductions will be discontinued from the treatment and entered into the follow-up phase, unless otherwise agreed between the Investigator and AGICC.

Management of hemolytic anemia (hemolysis)[e]

Event	Guidance
Grade 1: Laboratory evidence of hemolysis only	• Workup and evaluation: 1) Consider new medications and infectious causes; 2) CBC, CMP (including total, direct, and indirect bilirubin), LDH, haptoglobin, reticulocyte count, folate, B12, iron studies, peripheral smear, urinalysis, PT/INR, PTT, autoimmune serology, direct agglutinin test[d]<br>• Continue evorpacept (ALX148), cetuximab, and pembrolizumab at the same doses<br>• Continue with standard monitoring guidelines
Grade 2: Evidence of hemolysis and >=2 g decrease in hemoglobin	• Workup and evaluation as listed in Grade 1<br>• Hold evorpacept (ALX148), cetuximab, and pembrolizumab<br>• If autoimmune hemolytic anemia is suspected, administer 0.5-1 mg/kg/d prednisone equivalents, followed by ≥ 1-month taper. Monitor Hgb levels at least weekly until the steroid tapering process is complete.<br>• If event resolves to Grade 1 or better within 2 weeks, may consider resuming evorpacept (ALX148), cetuximab, and pembrolizumab at same doses, if approved by the medical monitor[a,b,c]
Grade 3: Transfusion or medical intervention indicated	• Workup and evaluation as listed in Grade 1<br>• Permanently discontinue study treatments

	• Consider admitting patient<br>• Hematology consult<br>• If autoimmune hemolytic anemia is suspected, administer prednisone 1-2 mg/kg/d (oral or IV equivalent depending on symptoms or speed of development), followed by ≥ 1-month taper. Monitor Hgb levels at least weekly until the steroid tapering process is complete.<br>• Consider RBC transfusion. Do not transfuse more than the minimum number of RBC units necessary to relieve symptoms of anemia or to return a patient to a safe hemoglobin range (7-8 g/dL in stable, noncardiac inpatients).[d]<br>• Begin folic acid 1 mg daily.
Grade 4: Life-threatening consequences; urgent intervention indicated	• Workup and evaluation as listed in Grade 1<br>• Permanently discontinue study treatments<br>• Admit patient<br>• Hematology consult<br>• IV corticosteroid (prednisone equivalent 1-2 mg/kg/d), followed by ≥ 1-month taper. Monitor Hgb levels at least weekly until the steroid tapering process is complete.<br>• If no improvement or worsening on corticosteroids or severe symptoms on presentation, initiate other immunosuppressive drugs, such as rituximab, IVIG, cyclosporine, infliximab, MMF, or ATG.<br>• Consider RBC transfusion. Do not transfuse more than the minimum number of RBC units necessary to relieve symptoms of anemia or to return a patient to a safe hemoglobin range (7-8 g/dL in stable, noncardiac inpatients).[d]<br>• Begin folic acid 1 mg daily.

a. If corticosteroids have been initiated, they must be tapered over ≥1 month to ≤10 mg/day oral prednisone or equivalent before study treatments can be resumed. Add prophylactic antibiotics for opportunistic infections.
b. The acceptable length of the extended period of time for which study treatments can be held to allow for corticosteroids to be reduced to ≤10 mg/day oral prednisone or equivalent must be agreed upon by the investigator and the Medical Monitor.
c. Resumption of study treatments may be considered in patients who are deriving benefit and have recovered from the immune-related event (baseline or Grade ≤1). Patients can be rechallenged with study treatments only after approval has been documented by both the investigator (or an appropriate delegate) and the Medical Monitor.
d. Because evorpacept (ALX148) binds to CD47 expressed on red blood cells (RBC), the presence of evorpacept (ALX148), which contains a modified IgG1 Fc domain, in patient whole blood and plasma can interfere with cross-matching assays performed by blood banks in order to identify compatible or least incompatible units of RBC for transfusion. Both the direct antibody test (DAT) and the indirect antibody test (IAT) may appear to be positive for reactive antibodies (IgG) in samples from patients who are on treatment with evorpacept (ALX148), due to the binding of AHG reagent to the Fc portion of evorpacept (ALX148). This phenomenon also occurs in patients receiving any Fc containing antibody-based targeted therapy that is directed against antigens present on RBC, such as the anti-CD38 antibodies used for treatment of multiple myeloma, as well as other anti-CD47 directed therapeutic antibodies. ALX148 does not interfere with binding to C3, and the DAT C3 test is interpretable. ABO blood type and baseline allo-antibodies (the latter in patients with a history of prior RBC

transfusion) can both be identified by performing a blood type and antibody screen at baseline prior to the administration of CD47-directed therapy. Additionally, genotyping of the patient's common and minor blood group antigens is suggested at baseline to provide guidance on which allo-antibodies they may develop following RBC transfusion. Additional ABO Rh blood typing and cross-matching testing may be performed on study as required by local regulations, but the results of these on-treatment tests may not be interpretable. If a blood transfusion is needed during study, the type and screen (and baseline genotyping of minor red cell antigens, if obtained) obtained during study screening should be used to guide these transfusions, and the blood bank should be made aware of these considerations and the presence of evorpacept (ALX148).
e. If hemolytic anemia is suspected to be due to hemophagocytic lymphohistiocytosis (HLH), follow management guidelines in Section 12.1.12.

12.1.4 MANAGEMENT OF DERMATOLOGIC TOXICITY (ACNEIFORM RASH)

Acneiform rash is usually due to cetuximab.

Event	Guidance
Grade 1-2<br><br>Grade 1: Papules and/or pustules covering < 10% BSA, which may or may not be associated with symptoms of pruritus or tenderness<br><br>Grade 2: Papules and/or pustules covering 10 - 30% BSA, which may or may not be associated with symptoms of pruritus or tenderness; associated with psychosocial impact; limiting instrumental ADL; papules and/or pustules covering > 30% BSA with or without mild symptoms	• Skin moisturizer applied to face, hands, feet, neck, back, and chest daily in the morning on rising; sunscreen (PABA free, SPF ≥ 15, UVA and UVB protection) applied to exposed skin areas before going outdoors; topical steroid (1% hydrocortisone cream) applied to face, hands, feet, neck, back, and chest at bedtime; and doxycycline 100 mg twice per day[72]<br>• Continue cetuximab at the same dose<br>• Continue evorpacept (ALX148) and pembrolizumab at the same doses
Grade 3-4, first occurrence<br><br>Grade 3: Papules and/or pustules covering >30% BSA with moderate or severe symptoms; limiting self-care ADL; associated with local superinfection with oral antibiotics indicated<br><br>Grade 4: Life-threatening consequences; papules and/or pustules covering any % BSA, which may or may not be associated with symptoms of pruritus or tenderness and are associated with extensive superinfection with IV antibiotics indicated	• Skin moisturizer applied to face, hands, feet, neck, back, and chest daily in the morning on rising; sunscreen (PABA free, SPF ≥ 15, UVA and UVB protection) applied to exposed skin areas before going outdoors; topical steroid (1% hydrocortisone cream) applied to face, hands, feet, neck, back, and chest at bedtime; and doxycycline 100 mg twice per day[72]<br>• Delay cetuximab infusion up to 2 weeks until improved to grade ≤2, then resume cetuximab with dose determined by institutional guidelines. If does not improve to grade ≤2 within 2 weeks, discontinue cetuximab<br>• Continue evorpacept (ALX148) and pembrolizumab at the same doses
Grade 3-4, second occurrence	• Skin moisturizer applied to face, hands, feet, neck, back, and chest daily in the morning on rising; sunscreen (PABA free, SPF ≥ 15, UVA and UVB protection) applied to exposed skin areas before going outdoors; topical steroid (1% hydrocortisone cream) applied to face, hands, feet, neck, back, and chest at

	bedtime; and doxycycline 100 mg twice per day[72]<br>• Delay cetuximab infusion up to 2 weeks until improved to grade ≤2, then resume cetuximab with dose determined by institutional guidelines. If does not improve to grade ≤2 within 2 weeks, discontinue cetuximab<br>• Continue evorpacept (ALX148) and pembrolizumab at the same doses
Grade 3-4, third occurrence	• Skin moisturizer applied to face, hands, feet, neck, back, and chest daily in the morning on rising; sunscreen (PABA free, SPF ≥ 15, UVA and UVB protection) applied to exposed skin areas before going outdoors; topical steroid (1% hydrocortisone cream) applied to face, hands, feet, neck, back, and chest at bedtime; and doxycycline 100 mg twice per day[72]<br>• Delay cetuximab infusion up to 2 weeks until improved to grade ≤2, then resume cetuximab with dose determined by institutional guidelines.<br>• If does not improve to grade ≤2 within 2 weeks, discontinue cetuximab<br>• Continue evorpacept (ALX148) and pembrolizumab at the same doses
Grade 3-4, fourth occurrence	• Skin moisturizer applied to face, hands, feet, neck, back, and chest daily in the morning on rising; sunscreen (PABA free, SPF ≥ 15, UVA and UVB protection) applied to exposed skin areas before going outdoors; topical steroid (1% hydrocortisone cream) applied to face, hands, feet, neck, back, and chest at bedtime; and doxycycline 100 mg twice per day[72]<br>• Discontinue cetuximab<br>• Continue evorpacept (ALX148) and pembrolizumab at the same doses

12.1.5 MANAGEMENT OF DERMATOLOGIC TOXICITY (OTHER THAN ACNEIFORM RASH)

Dermatologic toxicity (other than acneiform rash) may be due to evorpacept (ALX148), cetuximab, or pembrolizumab.

Event	Guidance
Grade 1-2<br><br>Grade 1: Macules/papules covering < 10% BSA with or without symptoms (e.g., pruritus, burning, tightness)<br><br>Grade 2: Macules/papules covering 10 - 30% BSA with or without symptoms (e.g., pruritus, burning, tightness); limiting instrumental ADL; rash covering > 30% BSA with or without mild symptoms	• Continue evorpacept (ALX148), cetuximab, and pembrolizumab.<br>• Initiate supportive care (e.g., antihistamines, topical corticosteroids). If event does not improve consider treatment with higher-potency topical corticosteroids.<br>• For Grade 2 rash, consider referral to dermatologist.

Grade 3 (Macules/papules covering >30% BSA with moderate or severe symptoms; limiting self care ADL)	• Withhold evorpacept (ALX148), cetuximab, and pembrolizumab<br>• Refer patient to dermatologist. A biopsy should be performed if appropriate.<br>• Consider initiating treatment with 10 mg/day oral prednisone or equivalent, increasing dose to 1-2 mg/kg/day if event does not improve within 48-72 hours.<br>• If event resolves to Grade 1 or better within 2 weeks, resume evorpacept (ALX148) (at one lower dose level) and resume cetuximab (with dose determined by institutional guidelines). May consider resuming pembrolizumab at fixed dose at discretion of investigator and medical monitor.[a,b]<br>• Permanently discontinue study treatments and contact Medical Monitor if event does not resolve to Grade 1 or better within 2 weeks (unless otherwise agreed between the Investigator and AGICC).[a,b,c]<br>• Permanently discontinue study treatments for confirmed Stevens-Johnson Syndrome, Toxic Epidermal Necrolysis, or Drug Rash with Eosinophilia and Systemic Symptoms and contact Medical Monitor.
Grade 4 (Life-threatening consequences)	• Permanently discontinue study treatments and contact Medical Monitor.

BID: twice daily; BSA: body surface area; PRN: as needed.

a. If corticosteroids have been initiated, they must be tapered over ≥1 month to ≤10 mg/day oral prednisone or equivalent before pembrolizumab can be resumed. Add prophylactic antibiotics for opportunistic infections.
b. The acceptable length of the extended period of time for which pembrolizumab can be held to allow for corticosteroids to be reduced to ≤10 mg/day oral prednisone or equivalent must be agreed upon by the investigator and the Medical Monitor.
c. Resumption of pembrolizumab may be considered in patients who are deriving benefit and have recovered from the immune-related event (baseline or Grade ≤1). Patients can be rechallenged with pembrolizumab only after approval has been documented by both the investigator (or an appropriate delegate) and the Medical Monitor.

12.1.6 MANAGEMENT OF HEPATOTOXICITY

Hepatotoxicity may be due to evorpacept (ALX148), cetuximab, or pembrolizumab, and may be immune-mediated.

Event	Guidance
Grade 1:<br>AST or ALT >ULN - 3.0 x ULN if baseline was normal; 1.5 - 3.0 x baseline if baseline was abnormal<br>and/or	• Continue evorpacept (ALX148), cetuximab, and pembrolizumab<br>• Continue with the standard monitoring plan

Total bilirubin >ULN - 1.5 x ULN if baseline was normal; > 1.0 - 1.5 x baseline if baseline was abnormal	
Grade 2:<br>AST or ALT >3.0 - 5.0 x ULN if baseline was normal; >3.0 - 5.0 x baseline if baseline was abnormal<br>and/or<br>Total bilirubin >1.5 - 3.0 x ULN if baseline was normal; >1.5 - 3.0 x baseline if baseline was abnormal	• Permanently discontinue study treatments if AST or ALT > 3x upper limit of normal (ULN) AND total bilirubin >2 x ULN, unless clear evidence of biliary obstruction exists<br>• Otherwise, continue evorpacept (ALX148) and cetuximab (may hold at Investigator discretion) and HOLD pembrolizumab<br>• Consider administering 0.5-1 mg/kg/day oral prednisone or equivalent followed by ≥ 1-month taper<br>• If event resolves to Grade 1 or better within 2 weeks, may consider resuming pembrolizumab at fixed dose at discretion of investigator and medical monitor.[a,b,c]<br>• Permanently discontinue pembrolizumab if event does not resolve to Grade 1 or baseline or better within 2 weeks (unless otherwise agreed between the Investigator and AGICC)[a,b,c]<br>• Monitor LFTs at least weekly.
Grade 3:<br>AST or ALT >5.0 - 20.0 x ULN if baseline was normal; >5.0 - 20.0 x baseline if baseline was abnormal<br>and/or<br>Total bilirubin >3.0 - 10.0 x ULN if baseline was normal; >3.0 - 10.0 x baseline if baseline was abnormal	• Permanently discontinue study treatments if AST or ALT > 3x upper limit of normal (ULN) AND total bilirubin >2 x ULN, unless clear evidence of biliary obstruction exists<br>• Otherwise, hold evorpacept (ALX148), cetuximab, and pembrolizumab<br>• Refer to a hepatologist and consider liver biopsy.<br>• Consider administering 1-2 mg/kg/day oral prednisone or equivalent followed by ≥ 1 month taper (for possible autoimmune hepatitis).<br>• If LFTs do not decrease within 48 hr after initiation of systemic steroids, consider adding an immunosuppressive agent (e.g., mycophenolate or TNF-antagonist). Infliximab might not be the most appropriate treatment option for immune mediated hepatitis given the risk for liver failure<br>• Monitor LFTs every 48-72 hours until decreasing and then follow weekly.<br>• Permanently discontinue study treatments for life-threatening hepatic events and contact the Medical Monitor.<br>• Permanently discontinue pembrolizumab. Restart evorpacept (ALX148, at one dose level reduction) and cetuximab (dose determined by institutional guidelines) once resolved to grade 1 or baseline or better, per Investigator discretion after discussion with the medical monitor
Grade 4<br>AST or ALT >20.0 x ULN if baseline was normal; >20.0 x baseline if baseline was abnormal<br>And/or<br>Total bilirubin >10.0 x ULN if baseline was normal; >10.0 x baseline if baseline was abnormal	• Permanently discontinue study treatments.<br>• Consult hepatologist and consider liver biopsy.<br>• Consider administering 1-2 mg/kg/day oral prednisone or equivalent (for possible autoimmune hepatitis). If event resolves to Grade 1 or better, taper corticosteroids over ≥1 month.<br>• If LFTs do not decrease within 48 hours after initiation of systemic steroids, addition of an alternative immunosuppressive agent (e.g., mycophenolate or TNF-α antagonist) or dose escalation of corticosteroids may be considered. Infliximab might

	not be the most appropriate treatment option for immune mediated hepatitis given the risk for liver failure<br>• Monitor LFTs every 48-72 hours until decreasing and then follow weekly.

IV intravenous; LFT:liver function test; q4w: every 4 weeks; TNF: tumor necrosis factor; ULN: upper limit of normal.

a. If corticosteroids have been initiated, they must be tapered over ≥1 month to ≤10 mg/day oral prednisone or equivalent before pembrolizumab can be resumed. Add prophylactic antibiotics for opportunistic infections.
b. The acceptable length of the extended period of time for which pembrolizumab can be held to allow for corticosteroids to be reduced to ≤10 mg/day oral prednisone or equivalent must be agreed upon by the investigator and the Medical Monitor.
c. Resumption of pembrolizumab may be considered in patients who are deriving benefit and have recovered from the immune-related event (baseline or Grade ≤1). Patients can be rechallenged with pembrolizumab only after approval has been documented by both the investigator (or an appropriate delegate) and the Medical Monitor.

12.1.7 MANAGEMENT OF PNEUMONITIS

Events of pneumonitis have been reported with pembrolizumab and cetuximab. All pulmonary events should be thoroughly evaluated for other commonly reported etiologies such as pneumonia/infection, lymphangitic carcinomatosis, pulmonary embolism, heart failure, chronic obstructive pulmonary disease, or pulmonary hypertension. Pneumonitis has not been reported with evorpacept (ALX148).

Event	Guidance
Grade 1 (Asymptomatic; clinical or diagnostic observations only; intervention not indicated)	• Continue evorpacept (ALX148), cetuximab, and pembrolizumab.<br>• Re-evaluate on serial imaging.<br>• Consider patient referral to pulmonary specialist<br>• For recurrent pneumonitis, treat as Grade 3 or 4 event.
Grade 2 (Symptomatic; medical intervention indicated; limiting instrumental ADL)	• Evorpacept (ALX148) may continue at the discretion of the investigator per medical judgment.<br>• Withhold pembrolizumab and cetuximab.<br>• Refer patient to pulmonary and infectious disease specialists and consider bronchoscopy or BAL.<br>• If bronchoscopy is consistent with immune-related etiology, initiate treatment with 1−2 mg/kg/day oral prednisone or equivalent.<br>• If event improves to Grade 1 or better within 2 weeks, resume cetuximab (dose determined by institutional guidelines). May consider resuming pembrolizumab at fixed dose at discretion of investigator and medical monitor.[a,b,c]<br>• Permanently discontinue study treatments and contact Medical Monitor if event does not resolve to Grade 1 or better within 2 weeks (unless otherwise agreed between the Investigator and AGICC).[a,b,c]<br>• For recurrent events, treat as a Grade 3 or 4 event.
Grade 3 (Severe symptoms; limiting self care ADL; oxygen indicated)	• Hold evorpacept (ALX148)<br>• Permanently discontinue pembrolizumab and cetuximab.

OR<br><br>Grade 4 (Life-threatening respiratory compromise; urgent intervention indicated (e.g., tracheotomy or intubation))	• Refer patient to pulmonary and infectious disease specialists and consider bronchoscopy or BAL.<br>• If bronchoscopy is consistent with immune-related etiology, initiate treatment with 1−2 mg/kg/day oral prednisone or equivalent.<br>• If pulmonary event does not improve within 48 hr or worsens, consider adding an immunosuppressive agent (e.g., infliximab, cyclophosphamide, IV Ig, or mycophenolate mofetil).<br>• If event resolves to Grade 1 or better, taper corticosteroids over ≥ 1 month.<br>• If event resolves to Grade 1 or better, evorpacept (ALX148) may be resumed at 1 dose level lower if deemed appropriate by the Investigator and medical monitor.

IV: intravenous; BAL: bronchoalveolar lavage.

a. If corticosteroids have been initiated, they must be tapered over ≥1 month to ≤10 mg/day oral prednisone or equivalent before pembrolizumab can be resumed. Add prophylactic antibiotics for opportunistic infections.
b. The acceptable length of the extended period of time for which pembrolizumab can be held to allow for corticosteroids to be reduced to ≤10 mg/day oral prednisone or equivalent must be agreed upon by the investigator and the Medical Monitor.
c. Resumption of pembrolizumab may be considered in patients who are deriving benefit and have recovered from the immune-related event (baseline or Grade ≤1). Patients can be rechallenged with pembrolizumab only after approval has been documented by both the investigator (or an appropriate delegate) and the Medical Monitor.

12.1.8 MANAGEMENT OF DIARRHEA & COLITIS

Diarrhea can frequently be managed with anti-diarrheal agents but can also progress to clinically significant dehydration and/or electrolyte imbalances with effects on other organs, possibly resulting in renal, hepatic, and/or cardiac failure. Patients should be instructed to promptly contact the investigators if they develop diarrhea. Investigators should treat diarrhea and intervene promptly for patients who appear to be at increased risk of developing significant dehydration, electrolyte imbalances, and/or multi-organ failure. Patients should receive maximum supportive care per institutional guidelines.

Immune-related colitis has been associated with the administration of pembrolizumab. For events of significant duration or severity or associated with signs of systemic inflammation or acute phase reactants, check for immune-related colitis.

General guidance

- All events of diarrhea or colitis should be thoroughly evaluated for more common etiologies other than drug-induced effects.
- For events of significant duration or severity or associated with signs of systemic inflammation or acute phase reactants, consider & investigate immune-related colitis.
- Administer anti-diarrheal agents and other maximal supportive care per institutional

guidelines such as: at the first report of watery diarrhea or loose stool, initiate maximal anti-diarrheal supportive care (Lomotil and loperamide).

- Suggested regimen:
  - Loperamide: Initiate dose with 4 mg, then 4 mg every 6 hours around the clock, alternating with Lomotil.
  - Lomotil (diphenoxylate and atropine): 2 tablets (diphenoxylate 5 mg, atropine 0.05 mg) every 6 hours around the clock
  - Continue Lomotil and loperamide until no loose stools for 24 hours.
  - If Grade ≤2 diarrhea persists after 48 hours total treatment with Lomotil and loperamide, consider second-line agents (e.g., octreotide, budesonide, tincture of opium).
- Oral supplementation:
  - Initiate oral supplementation of potassium and/or magnesium if serum levels are <LLN.
  - Consider oral rehydration therapy (e.g., Pedialyte) for Grade ≥1 diarrhea or vomiting.
  - If sufficient oral fluid intake is not feasible, fluid and electrolytes should be substituted via IV infusion
- Dietary modifications:
  - Stop all lactose-containing products and eat small meals.
  - The BRAT (banana, rice, apples, toast) diet, without fiber (other vegetables and fruits), may be helpful.
- Encourage adequate hydration with salt-containing liquids, such as broth or Gatorade.

Event	Guidance
Diarrhea, grade 1-2 (tolerable)<br><br>Diarrhea, grade 1: Increase of < 4 stools per day over baseline; mild increase in ostomy output compared to baseline<br><br>Diarrhea, grade 2: Increase of 4 - 6 stools per day over baseline; moderate increase in ostomy output compared to baseline; limiting instrumental ADL	• Continue evorpacept (ALX148), cetuximab, and pembrolizumab.<br>• Initiate supportive care and monitor patient closely.<br>• Investigate etiology, referring patient to GI specialist for evaluation of possible colitis if appropriate.<br>• Monitor participants for signs and symptoms of enterocolitis (ie, diarrhea, abdominal pain, blood or mucus in stool with or without fever) and of bowel perforation (ie, peritoneal signs and ileus)
Diarrhea, grade 2 (intolerable) or grade 3<br><br>Diarrhea, grade 3: Increase of >=7 stools per day over baseline; hospitalization indicated; severe increase in ostomy output compared to baseline; limiting self care ADL	• Hold evorpacept (ALX148), cetuximab, and pembrolizumab<br>• Initiate supportive care and monitor patient closely.<br>• Discontinue medications that may exacerbate colitis (e.g., NSAIDS) while investigating etiology.<br>• Investigate etiology, referring patient to GI specialist for evaluation of possible colitis, including biopsy if appropriate.

	• Initiate treatment with 1−2 mg/kg/day prednisone or equivalent[a]<br>• Monitor participants for signs and symptoms of enterocolitis (ie, diarrhea, abdominal pain, blood or mucus in stool with or without fever) and of bowel perforation (ie, peritoneal signs and ileus)<br>• If event resolves to Grade 1 or better within 2 weeks, resume evorpacept (ALX148, reduced by one level), cetuximab (dose determined by institutional guidelines), and pembrolizumab (fixed dose), unless otherwise agreed between the Investigator and medical monitor. If not, permanently discontinue study treatments.[a- c]
Diarrhea, grade 3 (recurrent) or grade 4:<br><br>Diarrhea, grade 4: Life-threatening consequences; urgent intervention indicated	• Permanently discontinue study treatments and contact Medical Monitor.<br>• Initiate supportive care and monitor patient closely.<br>• Discontinue medications that may exacerbate colitis (e.g., NSAIDS) while investigating etiology.<br>• Investigate etiology, referring patient to GI specialist for evaluation of possible colitis, including biopsy if appropriate.<br>• Initiate treatment with 1−2 mg/kg/day prednisone or equivalent[a]<br>• Monitor participants for signs and symptoms of enterocolitis (ie, diarrhea, abdominal pain, blood or mucus in stool with or without fever) and of bowel perforation (ie, peritoneal signs and ileus)
Colitis, grade 1: Asymptomatic; clinical or diagnostic observations only; intervention not indicated	• Continue evorpacept (ALX148), cetuximab, and pembrolizumab<br>• Initiate supportive care and monitor patient closely.<br>• Discontinue medications that may exacerbate colitis (e.g., NSAIDS).<br>• Refer patient to gastrointestinal specialist for evaluation and confirmatory biopsy if symptoms persist for > 7 days.<br>• Monitor participants for signs and symptoms of enterocolitis (ie, diarrhea, abdominal pain, blood or mucus in stool with or without fever) and of bowel perforation (ie, peritoneal signs and ileus)
Colitis, grade 2: Abdominal pain; mucus or blood in stool	• Evorpacept (ALX148) and cetuximab may continue at discretion of the investigator<br>• Withhold pembrolizumab<br>• Initiate supportive care and monitor patient closely.<br>• Discontinue medications that may exacerbate colitis (e.g., NSAIDS).<br>• Refer patient to gastrointestinal specialist for evaluation and confirmatory biopsy.<br>• Monitor participants for signs and symptoms of enterocolitis (ie, diarrhea, abdominal pain, blood or mucus in stool with or without fever) and of bowel perforation (ie, peritoneal signs and ileus)<br>• For recurrent events or events that persist ≥5 days,

	initiate treatment with 1-2 mg/kg/day oral prednisone or equivalent.<br>• If event resolves to Grade 1 or better within 2 weeks, resume pembrolizumab at fixed dose. If not, permanently discontinue pembrolizumab, unless otherwise agreed between the Investigator and medical monitor.[a-c]
Colitis, grade 3: Severe abdominal pain; peritoneal signs	• Hold evorpacept (ALX148), cetuximab, and pembrolizumab<br>• Initiate supportive care and monitor patient closely.<br>• Discontinue medications that may exacerbate colitis (e.g., NSAIDS).<br>• Refer patient to gastrointestinal specialist for evaluation and confirmatory biopsy.<br>• Monitor participants for signs and symptoms of enterocolitis (ie, diarrhea, abdominal pain, blood or mucus in stool with or without fever) and of bowel perforation (ie, peritoneal signs and ileus)<br>• Initiate treatment with 1-2 mg/kg/day IV methylprednisolone or equivalent and convert to 1-2 mg/kg/day oral prednisone or equivalent upon improvement.<br>• If event resolves to Grade 1 or better within 2 weeks (unless otherwise agreed between the Investigator and medical monitor), resume evorpacept (ALX148, reduced by one dose level) and cetuximab (dose determined by institutional guidelines). Pembrolizumab may be resumed at fixed dose after discussion with the medical monitor.[a-c]
Colitis, grade 3 (recurrent) or grade 4:<br><br>Colitis, grade 4: Life-threatening consequences; urgent intervention indicated	• Permanently discontinue study treatments and contact Medical Monitor.<br>• Initiate supportive care and monitor patient closely.<br>• Discontinue medications that may exacerbate colitis (e.g., NSAIDS).<br>• Refer patient to GI specialist for evaluation and confirmatory biopsy.<br>• Monitor participants for signs and symptoms of enterocolitis (ie, diarrhea, abdominal pain, blood or mucus in stool with or without fever) and of bowel perforation (ie, peritoneal signs and ileus)<br>• Initiate treatment with 1-2 mg/kg/day IV methylprednisolone or equivalent and convert to 1-2 mg/kg/day oral prednisone or equivalent upon improvement.<br>• If event does not improve within 48 hours after initiating corticosteroids, consider adding an immunosuppressive agent.<br>• If event resolves to Grade 1 or better, taper corticosteroids over ≥1 month.

GI: gastrointestinal; IV: intravenous; LLN: lower limit of normal; NSAID: non-steroidal anti-inflammatory drug.

a. If corticosteroids have been initiated, they must be tapered over ≥1 month to ≤10 mg/day oral

prednisone or equivalent before pembrolizumab can be resumed. Add prophylactic antibiotics for opportunistic infections.
b. The acceptable length of the extended period of time for which pembrolizumab can be held to allow for corticosteroids to be reduced to ≤10 mg/day oral prednisone or equivalent must be agreed upon by the investigator and the Medical Monitor.
c. Resumption of pembrolizumab may be considered in patients who are deriving benefit and have recovered from the immune-related event (baseline or Grade ≤1). Patients can be rechallenged with pembrolizumab only after approval has been documented by both the investigator (or an appropriate delegate) and the Medical Monitor.

12.1.9 MANAGEMENT OF INFUSION REACTION

Guidelines for infusion reactions to study drugs are provided below. See section 6.1.2 for recommended pre-medications in all patients. In general, if an infusion reaction occurs the infusion should be slowed (if mild reaction) or interrupted (if severe infection) and medical therapies should be administered. Medical therapies are at discretion of the investigator per medical judgment and may include antihistamines, antipyretics, glucocorticoids, epinephrine, bronchodilators, and oxygen. In the event of a grade 3-4 event, the drug should be permanently discontinued.

Grading of infusion related reaction (NCI CTCAE v5.0)

- Grade 1: Mild transient reaction; infusion interruption not indicated; intervention not indicated
- Grade 2: Therapy or infusion interruption indicated but responds promptly to symptomatic treatment (e.g., antihistamines, NSAIDS, narcotics, IV fluids); prophylactic medications indicated for < =24 hrs
- Grade 3: Prolonged (e.g., not rapidly responsive to symptomatic medication and/or brief interruption of infusion); recurrence of symptoms following initial improvement; hospitalization indicated for clinical sequelae
- Grade 4: Life-threatening consequences; urgent intervention indicated

Evorpacept (ALX148)

The infusion rate should be reduced or interrupted in the case of symptoms of infusion reaction, and symptomatic treatment administered. The infusion may be continued at one-half the previous rate upon improvement of symptoms. If symptoms persist or worsen, the infusion should be discontinued. In the event of an evorpacept (ALX148) infusion related reaction characterized by fever and chills, and less commonly hypotension, either experienced by a particular patient or if seen in other patients, pre-treatment medication should be administered to reduce the incidence and severity. In this scenario, patients should be pre-treated with acetaminophen and

diphenhydramine (or other antihistamine) approximately 0.5 to 2 hours before each evorpacept (ALX148) administration. Suggested starting doses are 650 to 1000 mg acetaminophen and 50 mg diphenhydramine (or equivalent of other antihistamine) IV or oral. Two (2) additional doses of acetaminophen may be administered approximately every 4 hours after the initial pre-treatment or as needed. Evorpacept (ALX148) should be permanently discontinued for a grade 3 or 4 infusion reaction.

Cetuximab

Grade 1 or 2: Reduce the cetuximab infusion rate by 50%.

Grade 3 or 4: Immediately and permanently discontinue cetuximab.

Pembrolizumab

Grade 1 or 2: Interrupt infusion or slow the infusion rate.

Grade 3 or 4: Permanently discontinue pembrolizumab.

12.1.10 MANAGEMENT OF ENDOCRINE TOXICITY

Event	Guidance
New-onset type I diabetes mellitus or grade 3 or 4 hyperglycemia associated with evidence of β-cell failure<br><br>Grade 3 hyperglycemia: Insulin therapy initiated; hospitalization indicated<br><br>Grade 4 hyperglycemia: Life-threatening consequences; urgent intervention indicated	• Withhold evorpacept (ALX148), cetuximab, and pembrolizumab<br>• Initiate insulin replacement therapy for participants with type I diabetes mellitus<br>• Administer antihyperglycemic in participants with hyperglycemia<br>• Monitor participants for hyperglycemia or other signs and symptoms of diabetes<br>• Evorpacept (ALX148), cetuximab, and pembrolizumab can be resumed when the event improves to ≤Grade 1 or baseline
Hypophysitis grade 2: Moderate; minimal, local or noninvasive intervention indicated; limiting age-appropriate instrumental ADL	• Hold pembrolizumab<br>• Continue or hold evorpacept (ALX148) and cetuximab at Investigator's discretion<br>• Administer corticosteroids and initiate hormonal replacements as clinically indicated<br>• Monitor for signs and symptoms of hypophysitis (including hypopituitarism and adrenal insufficiency)
Hypophysitis, grade 3 or 4<br><br>Hypophysitis, grade 3: Severe or medically significant but not immediately life-threatening; hospitalization or	• Hold evorpacept (ALX148), cetuximab, and pembrolizumab<br>• Administer corticosteroids and initiate hormonal replacements as clinically indicated<br>• Monitor for signs and symptoms of hypophysitis

prolongation of existing hospitalization indicated; limiting self care ADL<br><br>Hypophysitis, grade 4: Life-threatening consequences; urgent intervention indicated	(including hypopituitarism and adrenal insufficiency)<br>• If event improves to ≤Grade 1 or baseline, resume evorpacept (ALX148), cetuximab, and pembrolizumab after discussion with the medical monitor. Pembrolizumab can be permanently discontinued at the discretion of the investigator and medical monitor.
Hyperthyroidism, grade 2: Symptomatic; thyroid suppression therapy indicated; limiting instrumental ADL	• Continue evorpacept (ALX148), cetuximab, and pembrolizumab<br>• Treat with nonselective beta-blockers (eg, propranolol) or thionamides as appropriate<br>• Monitor for signs and symptoms of thyroid disorders
Hyperthyroidism, grade 3 or 4<br><br>Hyperthyroidism, grade 3: Severe symptoms; limiting self care ADL; hospitalization indicated<br><br>Hyperthyroidism, grade 4: Life-threatening consequences; urgent intervention indicated	• Withhold evorpacept (ALX148), cetuximab, and pembrolizumab<br>• Treat with nonselective beta-blockers (eg, propranolol) or thionamides as appropriate<br>• Monitor for signs and symptoms of thyroid disorders<br>• If event improves to ≤Grade 2 or baseline, resume evorpacept (ALX148), cetuximab, and pembrolizumab after discussion with the medical monitor. Pembrolizumab can be permanently discontinued at the discretion of the investigator and medical monitor.
Hypothyroidism, grade 2<br><br>Hypothyroidism, grade 2: Symptomatic; thyroid replacement indicated; limiting instrumental ADL	• Continue evorpacept (ALX148), cetuximab, and pembrolizumab<br>• Initiate thyroid replacement hormones (eg, levothyroxine or liothyronine) per standard of care<br>• Monitor for signs and symptoms of thyroid disorders
Hypothyroidism, grade 3 or 4<br><br>Hypothyroidism, grade 3: Severe symptoms; limiting self care ADL; hospitalization indicated<br><br>Hypothyroidism, grade 4: Life-threatening consequences; urgent intervention indicated	• Permanently discontinue study treatments<br>• Initiate thyroid replacement hormones (eg, levothyroxine or liothyronine) per standard of care<br>• Consider endocrine consultation<br>• Monitor for signs and symptoms of thyroid disorders

Management of additional endocrine immune-related adverse events is as per standard protocols.

12.1.11 MANAGEMENT OF RENAL TOXICITY

Nephritis (graded according to creatinine increased), grade 2: creatinine >1.5 - 3.0 x baseline or >1.5 - 3.0 x ULN	• Withhold pembrolizumab<br>• Continue evorpacept (ALX148) and cetuximab, or may hold at investigator's discretion<br>• Administer corticosteroids (prednisone 1 to 2 mg/kg or equivalent) followed by taper[a-c]<br>• Monitor changes of renal function<br>• If event improves to ≤Grade 1 or baseline, resume evorpacept (ALX148), cetuximab, and pembrolizumab. Pembrolizumab is resumed at fixed dose. Dose reductions of evorpacept (ALX148) and

	cetuximab are not required but can be considered upon discussion with the medical monitor. [a-c]
Nephritis (graded according to creatinine increased), grade 3 or 4<br><br>Nephritis, grade 3: creatinine >3.0 x baseline or >3.0 - 6.0 x ULN<br><br>Nephritis, grade 4: creatinine >6.0 x ULN	• Permanently discontinue evorpacept (ALX148), cetuximab, and pembrolizumab. Permanently discontinue study treatments<br>• Administer corticosteroids (prednisone 1 to 2 mg/kg or equivalent) followed by taper[a]<br>• Monitor changes of renal function

a. If corticosteroids have been initiated, they must be tapered over ≥1 month to ≤10 mg/day oral prednisone or equivalent before pembrolizumab can be resumed. Add prophylactic antibiotics for opportunistic infections.
b. The acceptable length of the extended period of time for which pembrolizumab can be held to allow for corticosteroids to be reduced to ≤10 mg/day oral prednisone or equivalent must be agreed upon by the investigator and the Medical Monitor.
c. Resumption of pembrolizumab may be considered in patients who are deriving benefit and have recovered from the immune-related event (baseline or Grade ≤1). Patients can be rechallenged with pembrolizumab only after approval has been documented by both the investigator (or an appropriate delegate) and the Medical Monitor.

12.1.12 MANAGEMENT OF HEMOPHAGOCYTIC LYMPHOHISTIOCYTOSIS

Hemophagocytic lymphohistiocytosis (HLH) is a syndrome of excessive immune activation that can result in life-threatening tissue destruction. Manifestations include the following diagnostic criteria (the presence of at least 5 of 8 criteria are suggested to satisfy the diagnosis):[73]

- Fever ≥38.5°C
- Splenomegaly
- Peripheral blood cytopenia, with at least two of the following: hemoglobin <9 g/dL, platelets <100,000/microL; absolute neutrophil count <1000/microL
- Hypertriglyceridemia (fasting triglycerides >265 mg/dL) and/or hypofibrinogenemia (fibrinogen <150 mg/dL)
- Hemophagocytosis in bone marrow, spleen, lymph node, or liver
- Low or absent NK cell activity
- Ferritin >500 ng/mL
- Elevated soluble CD25 (soluble IL-2 receptor alpha [sIL-2R]) two standard deviations above age-adjusted laboratory-specific norms

Additional manifestations of HLH can include pulmonary abnormalities (such as hypoxia or consolidation or ground-glass opacities on imaging), neurologic abnormalities (such as ataxia, seizures, or mental status changes), liver function and coagulation abnormalities, hypotension, renal dysfunction, skin manifestations, and bleeding.

HLH has been reported to occur in patients receiving immune checkpoint inhibitors including in <1% of patients administered pembrolizumab (reference USPI Keytruda). Suggested treatment guidelines are based on these reports, as well as the HLH-94 protocol.[74-76]

HLH is not included in NCI CTCAE 5.0, so grading is based on the term "Immune system disorders - Other, specify":

Event	Guidance
Grade 1: Asymptomatic or mild symptoms; clinical or diagnostic observations only; intervention not indicated	• Obtain the following evaluation: CBC, CMP (including total, direct, and indirect bilirubin), LDH, haptoglobin, reticulocyte count, folate, B12, iron studies, peripheral smear, urinalysis, PT/INR, PTT, autoimmune serology, direct agglutinin test[a], fibrinogen, D-dimer, GGT, triglycerides, ferritin, NK cell activity, soluble IL-2 receptor alpha (soluble CD25), CXCL9, interferon gamma, TNF alpha, IL-6, IL-10, and IL-12[b]<br>• Obtain study tubes for PK, PD, and ADA (see Section 8.2.6)<br>• CT scan of the chest, abdomen, and pelvis<br>• Continue evorpacept (ALX148), cetuximab, and pembrolizumab at the same doses<br>• Continue with standard monitoring guidelines
Grade 2: Moderate; minimal, local or noninvasive intervention indicated; limiting age-appropriate instrumental ADL	• Permanently discontinue study treatments<br>• Obtain evaluation as listed in Grade 1, including laboratories, study tubes, and CT scan<br>• Obtain tissue biopsy of the bone marrow, lymph node, or liver if feasible<br>• Do not wait on diagnostic confirmation to start treatment. Administer 1 mg/kg/d prednisone equivalents, followed by ≥ 1-month taper. Monitor blood work at least weekly until the steroid tapering process is complete.
Grade 3: Severe or medically significant but not immediately life threatening; hospitalization or prolongation of existing hospitalization indicated; limiting self care ADL<br><br>Grade 4: Life-threatening consequences; urgent intervention indicated	• Permanently discontinue study treatments<br>• Admit the patient to the hospital<br>• Obtain evaluation as listed in Grade 1, including laboratories, study tubes, and CT scan<br>• Obtain tissue biopsy of the bone marrow, lymph node, or liver if feasible<br>• Do not wait on diagnostic confirmation to start treatment. Administer methylprednisolone 1 gram daily x3 days. Then, prednisone (or equivalent) minimum dose 1 mg/kg for at least one week, per investigator discretion. Then, gradual taper over at least 8 weeks total, per investigator discretion<br>• Monitor blood work at least weekly until the steroid tapering process is complete<br>• Steroids are the suggested primary treatment. Additional immunosuppressive medications (such as tocilizumab or the HLH-94 protocol[c]) may be added at the discretion of the investigator and in consultation with the medical monitor

a. Because evorpacept (ALX148) binds to CD47 expressed on red blood cells (RBC), the presence of evorpacept (ALX148), which contains a modified IgG1 Fc domain, in patient whole blood and plasma can interfere with cross-matching assays performed by blood banks in order to identify compatible or least incompatible units of RBC for transfusion. Both the direct antibody test (DAT) and the indirect antibody test (IAT) may appear to be positive for reactive antibodies (IgG) in samples from patients who are on treatment with evorpacept

(ALX148), due to the binding of AHG reagent to the Fc portion of evorpacept (ALX148). This phenomenon also occurs in patients receiving any Fc containing antibody-based targeted therapy that is directed against antigens present on RBC, such as the anti-CD38 antibodies used for treatment of multiple myeloma, as well as other anti-CD47 directed therapeutic antibodies. ALX148 does not interfere with binding to C3, and the DAT C3 test is interpretable. ABO blood type and baseline allo-antibodies (the latter in patients with a history of prior RBC transfusion) can both be identified by performing a blood type and antibody screen at baseline prior to the administration of CD47-directed therapy. Additionally, genotyping of the patient's common and minor blood group antigens is suggested at baseline to provide guidance on which allo-antibodies they may develop following RBC transfusion. Additional ABO Rh blood typing and cross-matching testing may be performed on study as required by local regulations, but the results of these on-treatment tests may not be interpretable. If a blood transfusion is needed during study, the type and screen (and baseline genotyping of minor red cell antigens, if obtained) obtained during study screening should be used to guide these transfusions, and the blood bank should be made aware of these considerations and the presence of evorpacept (ALX148).

b. NK cell activity, soluble IL-2 receptor alpha (soluble CD25), CXCL9, interferon gamma, TNF alpha, IL-6, IL-10, and IL-12 are usually send-out tests. Acceptable methodology to measure NK cell activity include NK cell cytotoxicity or flow cytometry for reduced/absent NK cell perforin and/or CD107alpha.

c. The HLH-94 protocol includes treatment with etoposide and dexamethasone.[74]

Cytokine release syndrome (CRS) is a similar acute systemic inflammatory syndrome characterized by fever and multiple organ dysfunction (such as tachypnea, tachycardia, hypotension, and hypoxia). CRS can present similarly to HLH, and most commonly occurs following CAR-T cell therapy and allogeneic bone marrow transplant. If a patient experiences a syndrome that is felt by the Investigator to be more consistent with CRS than HLH, then establish published or local CRS management guidelines should be followed.

12.2 APPENDIX 2: PROTOCOL CRITERIA FOR MEASUREMENT OF STUDY EFFICACY ENDPOINTS (RECIST V1.1 AND IRECIST)

1 Definitions

1.1 Evaluable for adverse events. All patients will be evaluable for adverse event evaluation from the time of their first treatment.

1.2 Evaluable for response. All patients who have received at least one cycle of therapy and have their disease re-evaluated will be considered evaluable for response (exceptions will be those who exhibit objective disease progression prior to the end of cycle 1 who will also be considered evaluable). Patients on therapy for at least this period and who meet the other listed criteria will have their response classified according to the definitions set out below.

Response and progression will be evaluated in this study using the revised international criteria (1.1) proposed by the RECIST (Response Evaluation Criteria in Solid Tumors)[77] committee as well as the modified iRECIST guidelines.[78] Investigators should note the different requirements for confirmatory scans as well as follow up for the two criteria.

See section 4.1.2 for criteria for continuing treatment past RECIST 1.1 disease progression.

2 RECIST 1.1 Response and Evaluation Endpoints

2.1 Measurable Disease. Measurable tumor lesions (nodal, subcutaneous, lung parenchyma, solid organ metastases) are defined as those that can be accurately measured in at least one dimension (longest diameter to be recorded) as ≥20 mm with chest x-ray and as ≥10 mm with CT scan or clinical examination. Bone lesions are considered measurable only if assessed by CT scan and have an identifiable soft tissue component that meets these requirements (soft tissue component ≥10 mm by CT scan). Malignant lymph nodes must be ≥15 mm in the short axis to be considered measurable; only the short axis will be measured and followed. All tumor measurements must be recorded in millimeters (or decimal fractions of centimeters). Previously irradiated lesions are not considered measurable unless progression has been documented in the lesion.

2.2 Non-measurable Disease. All other lesions (or sites of disease), including small lesions are considered nonmeasurable disease. Bone lesions without a measurable soft tissue component, leptomeningeal disease, ascites, pleural/pericardial effusions, lymphangitis cutis/pulmonis, inflammatory breast disease, lymphangitic involvement of lung or skin and abdominal masses followed by clinical examination are all non-measurable. Lesions in previously irradiated areas are non-measurable, unless progression has been demonstrated.

2.3 Target Lesions. When more than one measurable tumor lesion is present at baseline all lesions up to a maximum of 5 lesions total (and a maximum of 2 lesions per organ) representative of all involved organs should be identified as target lesions and will be recorded and measured at baseline. Target lesions should be selected on the basis of their size (lesions with the longest diameter), be representative of all involved organs, but in addition should be those that lend themselves to reproducible repeated measurements. Note that pathological nodes must meet the criterion of a short axis of ≥15 mm by CT scan and only the short axis of these nodes will contribute to the baseline sum. All other pathological nodes (those with short axis ≥10 mm but <15 mm) should be considered non-target lesions. Nodes that have a short axis <10 mm are considered non-pathological and should not be recorded or followed (see 8.2.4). At baseline, the sum of the target lesions (longest diameter of tumor lesions plus short axis of lymph nodes: overall maximum of 5) is to be recorded.

After baseline, a value should be provided on the eCRF for all identified target lesions for each assessment, even if very small. If extremely small and faint lesions cannot be accurately measured but are deemed to be present, a default value of 5 mm may be used. If lesions are too small to measure and indeed are believed to be absent, a default value of 0 mm may be used.

2.4 Non-target Lesions. All non-measurable lesions (or sites of disease) plus any measurable lesions over and above those listed as target lesions are considered non-target lesions. Measurements are not required but these lesions should be noted at baseline and should be followed as “present” or “absent”.

2.5 Response.

All patients will have their BEST RESPONSE from the start of study treatment until the end of treatment classified as outlined below:

Complete Response (CR): disappearance of target and non-target lesions and normalization of tumor markers. Pathological lymph nodes must have short axis measures <10 mm (Note: continue to record the measurement even if <10 mm and considered CR). Residual lesions (other than nodes <10 mm) thought to be non-malignant should be further investigated (by cytology specialized imaging or other techniques as appropriate for individual cases before CR can be accepted.

Partial Response (PR): at least a 30% decrease in the sum of measures (longest diameter for tumor lesions and short axis measure for nodes) of target lesions, taking as reference the baseline sum of diameters. Non target lesions must be non-PD.

Stable Disease (SD): Neither sufficient shrinkage to qualify for PR nor sufficient increase to qualify for PD taking as reference the smallest sum of diameters on study.

Progressive Disease (PD): at least a 20% increase in the sum of diameters of measured lesions taking as references the smallest sum of diameters recorded on study (including baseline) AND an absolute increase of ≥5 mm. Appearance of new lesions will also constitute progressive disease (including lesions in previously unassessed areas). In exceptional circumstances, unequivocal progression of non-target disease may be accepted as evidence of disease progression, where the overall tumor burden has increased sufficiently to merit discontinuation of treatment or where the tumor burden appears to have increased by at least 73% in volume. Modest increases in the size of one or more non-target lesions are NOT considered unequivocal progression. If the evidence of PD is equivocal (target or non-target), treatment may continue until the next assessment, but if confirmed, the earlier date must be used.

Table 4: Integration of target, non-target, and new lesions into response assessment

Target Lesions	Non-Target Lesions	New Lesions	Overall Response	Best Response for this Category also Requires
Target lesions ± non target lesions				
CR	CR	No	CR	Normalization of tumour markers, tumour nodes <10 mm
CR	Non-CR/Non-PD	No	PR	
CR	Not all evaluated	No	PR	
PR	Non-PD/ not all evaluated	No	PR	
SD	Non-PD/ not all evaluated	No	SD	Documented at least once ≥4 wks. from baseline
Not all evaluated	Non-PD	No	NE	
PD	Any	Any	PD	
Any	PD	Any	PD	
Any	Any	Yes	PD	
Non target lesions ONLY				
No Target	CR	No	CR	Normalization of tumour markers, tumour nodes <10 mm
No Target	Non-CR/non-PD	No	Non-CR/non-PD	
No Target	Not all evaluated	No	NE	
No Target	Unequivocal PD	Any	PD	
No Target	Any	Yes*	PD	

Note: Patients with a global deterioration of health status requiring discontinuation of treatment without objective evidence of disease progression at that time should be reported as "symptomatic deterioration". This is a reason for stopping therapy, but is NOT objective PD. Every effort should be made to document the objective progression even after discontinuation of treatment.

*Investigators should record all new lesions; if the new lesion is felt to be equivocal, treatment may be continued pending further assessments – see table 2.

3 iRECIST Response Assessment

Overall response will also be assessed using iRECIST. Immunotherapeutics may result in infiltration of immune cells leading to transient increase in the size in malignant lesions, or undetectable lesions becoming detectable. The criteria are identical to those of RECIST 1.1 in many respects but have been adapted to account for instances where an increase in tumor burden, or the appearance of new lesions, does not reflect true tumor progression.

Key differences are described below. All responses defined using iRECIST criteria are designated with a prefix. iRECIST time-point and best overall responses will be recorded separately.

3.1 Confirming Progression

Unlike RECIST 1.1, iRECIST requires the confirmation of progression and uses the terms iUPD (unconfirmed progression) and iCPD (confirmed progression). Confirmatory scans should be performed at least 4 weeks, but no longer than 8 weeks after iUPD.

iCPD is confirmed if further increase in tumor burden, compared to the last assessment, is seen as evidenced by one or more of the following:

- Continued increase in tumor burden (from iUPD) where RECIST 1.1 definitions of progression had been met (from nadir) in target, non-target disease or new lesions
    - Progression in target disease worsens with an increase of at least 5 mm in the absolute value of the sum
    - Continued unequivocal progression in non-target disease with an increase in tumor burden
    - Increase in size of previously identified new lesion (s) (an increase of at least 5 mm in the absolute value of the sum of those considered to be target new lesions) or additional new lesions.
- RECIST 1.1 criteria are met in lesions types (target or non-target or new lesions) where progression was not previously identified, including the appearance of additional new lesions.

If iUPD is not confirmed at the next assessment, then the appropriate response will be assigned (iUPD if the criteria are still met, but no worsening, or iSD, iPR or iCR if those criteria are met compared to baseline). As can be seen in Table 5, the prior documentation of iUPD does not preclude assigning iCR, iPR, or iSD in subsequent time-point assessments or as best overall response (BOR) providing that iCPD is not documented at the next assessment after iUPD.

3.2 New lesions

New lesions should be assessed and measured as they appear using RECIST 1.1 criteria (maximum of 5 lesions, no more than 2 per site, at least 10 mm in long axis (or 15 mm in short axis for nodal lesions), and recorded as New Lesions-Target (NLT) and New Lesion-Non-Target (NLNT) to allow clear differentiation from baseline target and non-target lesions.

New lesions may either meet the criteria of NLT or NLNT to drive iUPD (or iCPD). However, the measurements of target lesions should NOT be included in the sum of measures of original target lesions identified at baseline. Rather, these measurements will be collected on a separate table in the case record form.

PD is confirmed in the New Lesion category if the next imaging assessment, conducted at least 4 weeks (but not more than 8 weeks) after iUPD confirms further progression from iUPD with either an increase of at least 5 mm in the absolute value of the sum of NLT OR an increase (but not necessarily unequivocal increase) in the size of NLNT lesions OR the appearance of additional new lesions.

Table 5: Time-point (TP) iResponse

Target Lesions*	Non-Target Lesions*	New Lesions*	Time Point Response	
			No prior iUPD**	Prior iUPD**; ***
iCR	iCR	No	iCR	iCR
iCR	Non-iCR/Non-iUPD	No	iPR	iPR
iPR	Non-iCR/Non-iUPD	No	iPR	iPR
iSD	Non-iCR/Non-iUPD	No	iSD	iSD
iUPD with no change OR decrease from last TP	iUPD with no change OR decrease from last TP	Yes	NA	NLs confirms iCPD if NLs were previously identified and increase in size (≥5 mm in SOM for NLT or any increase for NLNT) or number. If no change in NLs (size or number) from last TP, remains iUPD
iSD	iUPD	No	iUPD	Remains iUPD unless iCPD confirmed based in further increase in size of NT disease (need not meet RECIST 1.1 criteria for unequivocal PD)
iUPD	Non-iCR/Non-iUPD	No	iUPD	Remains iUPD unless iCPD confirmed based on:<br>o further increase in SOM of at least 5 mm, otherwise remains iUPD
iUPD	iUPD	No	iUPD	Remains iUPD unless iCPD confirmed based on further increase in:<br>o previously identified T lesion iUPD SOM ≥5 mm and / or<br>o NT lesion iUPD (prior assessment - need not be unequivocal PD)
iUPD	iUPD	Yes	iUPD	Remains iUPD unless iCPD confirmed based on further increase in:<br>o previously identified T lesion iUPD ≥5 mm and / or<br>o previously identified NT lesion iUPD (need not be unequivocal) and /or<br>o size or number of new lesions previously identified
Non-iUPD/PD	Non-iUPD/PD	Yes	iUPD	Remains iUPD unless iCPD confirmed based on<br>o increase in size or number of new lesions previously identified
* Using RECIST 1.1 principles. If no PSPD occurs, RECIST 1.1 and iRECIST categories for CR, PR and SD would be the same. ** in any lesion category. *** previously identified in assessment immediately prior to this TP.				

All patients will have their iBOR from the start of study treatment until the end of treatment classified as outlined below (Table 6).

Table 6: iRECIST Best Overall Response (iBOR)

TPR1	TPR2	TPR3	TPR4	TPR5	iBOR
iCR	iCR, iPR, iUPD, NE	iCR, iPR, iUPD, NE	iUPD	iCPD	iCR
iUPD	iPR, iSD, NE	iCR	iCR, iPR, iSD, iUPD, NE	iCR, iPR, iSD, iUPD, iCPD, NE	iCR
iUPD	iPR	iPR, iSD, iUPD, NE	iPR, iSD, iUPD, NE, iCPD	iPR, iSD, iUPD, NE, iCPD	iPR
iUPD	iSD, NE	PR	iPR, iSD, iUPD, NE	iPR, iSD, iUPD, iCPD, NE	iPR
iUPD	iSD	iSD, iUPD, NE	iSD, iUPD, iCPD, NE	iSD, iUPD, ICPD, NE	iSD
iUPD	iCPD	Anything	Anything	Anything	iCPD
iUPD	iUPD	iCPD	Anything	Anything	iCPD
iUPD	NE	NE	NE	NE	iUPD

- Table assumes a randomised study where confirmation of CR or PR is not required.
- NE = not evaluable that cycle.
- Designation "I" for BOR can be used to indicate prior iUPD to aid in data interpretation.
- For patients with non-target disease only at baseline, only CR or non-CR/non-PD can be assigned at each TPR but is not shown in the table for ease of presentation.

5 Response and Stable Disease Duration (RECIST 1.1 and iRECIST)

Response duration will be measured from the time measurement criteria for CR/PR or iCR/iPR (whichever is first recorded) are first met until the first date that recurrent or progressive disease is objectively documented, taking as reference the smallest measurements recorded on study (including baseline).

Stable disease duration will be measured from the time of start of treatment until the criteria for progression are met, taking as reference the smallest sum on study (including baseline).

6 Methods of Measurement

The same method of assessment and the same technique should be used to characterize each identified and reported lesion at baseline and during follow-up. Assessments should be identified on a calendar schedule and should not be affected by delays in therapy. While on study, all lesions recorded at baseline should have their actual measurements recorded at each subsequent evaluation, even when very small (e.g. 2 mm). If it is the opinion of the radiologist that the lesion has likely disappeared, the measurement should be recorded as 0 mm. If the lesion is believed to be present and is faintly seen but too small to measure, a default value of 5 mm should be assigned. For lesions which fragment/split add together the longest diameters of the fragmented portions; for lesions which coalesce, measure the maximal longest diameter for the "merged lesion".

6.1 Clinical Lesions. Clinical lesions will only be considered measurable when they are superficial and ≥10 mm as assessed using calipers (e.g. skin nodules). For the case of skin lesions, documentation by color photography including a ruler to estimate the size of the lesion is recommended. If feasible, imaging is preferred.

6.2 Chest X-ray. Chest CT is preferred over chest X-ray, particularly when progression is an important endpoint, since CT is more sensitive than X-ray, particularly in identifying new

lesions. However, lesions ≥20 mm on chest X-ray may be considered measurable if they are clearly defined and surrounded by aerated lung.

6.3 CT, MRI. CT is the best currently available and reproducible method to measure lesions selected for response assessment. This guideline has defined measurability of lesions on CT scan based on the assumption that CT slice thickness is 5 mm or less. When CT scans have slice thickness greater than 5 mm, the minimum size for a measurable lesion should be twice the slice thickness. MRI is also acceptable in certain situations (e.g. for body scans). Other specialized imaging or other techniques may also be appropriate for individual case. For example, while PET scans are not considered adequate to measure lesions, PET-CT scans may be used providing that the measures are obtained from the CT scan and the CT scan is of identical diagnostic quality to a diagnostic CT (with IV and oral contrast).

6.4 Ultrasound. Ultrasound is not useful in assessment of lesion size and should not be used as a method of measurement. If new lesions are identified by ultrasound in the course of the study, confirmation by CT is advised.

6.5 Endoscopy, Laparoscopy. The utilization of these techniques for objective tumor evaluation is not advised. However, they can be useful to confirm complete pathological response when biopsies are obtained or to determine relapse in trials where recurrence following complete response or surgical resection is an endpoint.

6.6 Tumor Markers. Tumor markers alone cannot be used to assess objective tumor response. If markers are initially above the upper normal limit, however, they must normalize for a patient to be considered in complete response.

6.7 Cytology, Histology. These techniques can be used to differentiate between PR and CR in rare cases if required by protocol (for example, residual lesions in tumor types such as germ cell tumors, where known residual benign tumors can remain). When effusions are known to be a potential adverse effect of treatment (e.g. with certain taxane compounds or angiogenesis inhibitors), the cytological confirmation of the neoplastic origin of any effusion that appears or worsens during treatment when the measurable tumor has met criteria for response or stable disease is advised to differentiate between response or stable disease and progressive disease

12.3 APPENDIX 3: ECOG PERFORMANCE STATUS

Grade	ECOG Performance Status[79]
0	Fully active, able to carry on all pre-disease performance without restriction.
1	Restricted in physically strenuous activity but ambulatory and able to carry out work of a light or sedentary nature, eg, light house work, office work.
2	Ambulatory and capable of all self-care but unable to carry out any work activities. Up and about more than 50% of waking hours.
3	Capable of only limited self-care, confined to bed or chair more than 50% of waking hours.
4	Completely disabled. Cannot carry on any self-care. Totally confined to bed or chair.
5	Dead.

12.4 APPENDIX 4: EFFICACY OF MCRC TREATMENT OPTIONS, INCLUDING THIRD LINE DRUGS, EGFR INHIBITORS, AND A PD-1 INHIBITOR

Drug (trial name)	Population	ORR (%)	DCR (%)	mPFS (mo)	mOS (mo)
Regorafenib vs placebo (CORRECT)[10]	mCRC without standard treatment options	1.0 vs 0.4	41.0 vs 15.0	1.9 vs 1.7	6.4 vs 5.0
Regorafenib vs placebo (CONCUR)[11]	mCRC >2L	4.9 vs 0	51.0 vs 7.0	3.2 vs 1.7	8.8 vs 6.3
Trifluridine-tipiracil vs placebo (RECOURSE)[12]	mCRC >2L	1.6 vs 0.4	44.0 vs 16.0	2.0 vs 1.7	7.1 vs 5.3
Trifluridine-tipiracil vs placebo (TERRA)[13]	mCRC >2L	1.1 vs 0	44.1 vs 14.6	2.0 vs 1.8	7.8 vs 7.1
Panitumumab/BSC vs BSC[5]	KRAS mutant mCRC >2L, retrospective analysis	0 vs NR	12 vs 8	1.7 vs 1.7	4.9 vs 4.4
Cetuximab/BSC vs BSC (CO.17)[4]	KRAS mutant mCRC without standard treatment options, retrospective analysis	1.2 vs 0	NR	1.8 vs 1.8	4.5 vs 4.6
EGFR inhibitor[#] + chemotherapy vs chemotherapy ± bevacizumab[38]	Right-sided RAS WT mCRC 1L^, retrospective analysis of 6 clinical trials	Hazard ratio 1.47 (NS)	NR	Hazard ratio 1.12 (NS)	Hazard ratio 1.12 (NS)
Pembrolizumab (single-arm)[14]	mCRC >2L, MSS	0	11.0	2.2	5.0

#: cetuximab or panitumumab
^: 1L in 5/6 trials, 2L in 1/6 trials
Abbreviations: BSC, best supportive care; DC, disease control rate; L, line of therapy; mCRC, metastatic colorectal cancer; mOS, median overall survival; mPFS, median progression free survival; NR, not reported; NS, not statistically significant; ORR, objective response rate; WT, wild-type.
